# Insights into the Ligand Binding to Bromodomain-Containing Protein 9 (BRD9): A Guide to the Selection of Potential Binders by Computational Methods

**DOI:** 10.3390/molecules26237192

**Published:** 2021-11-27

**Authors:** Simona De Vita, Maria Giovanna Chini, Giuseppe Bifulco, Gianluigi Lauro

**Affiliations:** 1 Department of Pharmacy, University of Salerno, Via Giovanni Paolo II 132, 84084 Fisciano, Italy; sdevita@unisa.it (S.D.V.); bifulco@unisa.it (G.B.); 2 Department of Biosciences and Territory, University of Molise, Contrada Fonte Lappone, Pesche, 86090 Isernia, Italy; mariagiovanna.chini@unimol.it

**Keywords:** molecular dynamics, BRD9, binding predictions, computational studies, molecular docking

## Abstract

The estimation of the binding of a set of molecules against BRD9 protein was carried out through an in silico molecular dynamics-driven exhaustive analysis to guide the identification of potential novel ligands. Starting from eight crystal structures of this protein co-complexed with known binders and one *apo* form, we conducted an exhaustive molecular docking/molecular dynamics (MD) investigation. To balance accuracy and an affordable calculation time, the systems were simulated for 100 ns in explicit solvent. Moreover, one complex was simulated for 1 µs to assess the influence of simulation time on the results. A set of MD-derived parameters was computed and compared with molecular docking-derived and experimental data. MM-GBSA and the per-residue interaction energy emerged as the main indicators for the good interaction between the specific binder and the protein counterpart. To assess the performance of the proposed analysis workflow, we tested six molecules featuring different binding affinities for BRD9, obtaining promising outcomes. Further insights were reported to highlight the influence of the starting structure on the molecular dynamics simulations evolution. The data confirmed that a ranking of BRD9 binders using key parameters arising from molecular dynamics is advisable to discard poor ligands before moving on with the synthesis and the biological tests.

## 1. Introduction

The highly regulated post-translational modifications (PTMs) on histone proteins ultimately determine the overall state of chromatin and the consequent gene expression, generating what is called “histone code”, a series of signals that can be interpreted by the recruitment of specific proteins that introduce (writers), remove (erasers), or recognize (readers) them [1,2,3]. Lysine acetylation (LysAc) is one of the most frequent, and it is a reversible PTM that adds an acetyl group on the *ε*-primary amine of lysine side chains. In this way, the positive charges of the amino acid are neutralized, disrupting the interactions between histones and the negatively charged DNA backbone. This leads to the detachment of DNA from the histones and generates a more relaxed chromatin state (euchromatin), promoting DNA transcription of the genes contained in that particular sequence [4].

**Figure 1:**
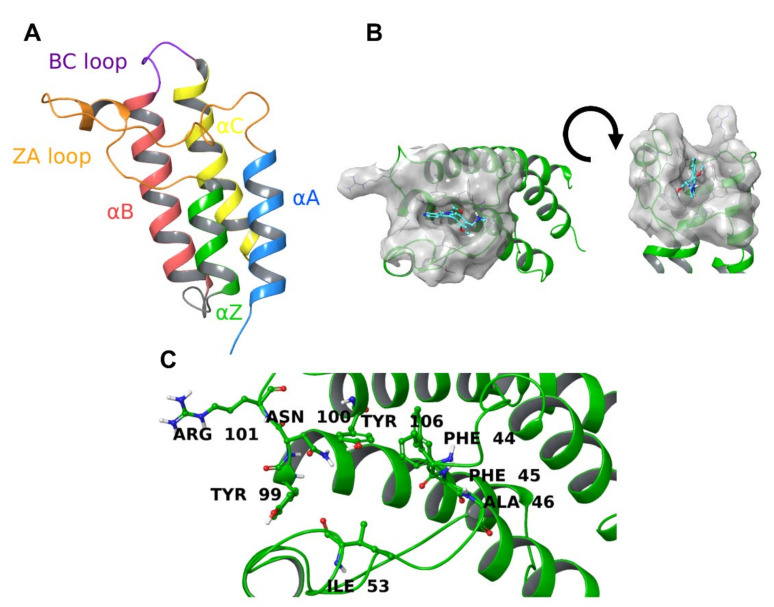
(**A**) Structure of BRD9 protein. Chains and loops are colored in different colors and named accordingly. (**B**) Binding site of BRD9 (PDB: 5F1H [15]). The co-crystallized ligand is in cyan, and the protein is represented by green ribbons with the molecular surface truncated at 7 Å from the ligand. (**C**) Key amino acids of BRD9 binding site.

Among the most important “readers” of the genetic code, bromodomains (BRDs) are conserved protein domains discovered for the first time in 1992 in the *Brahma* gene of *D. melanogaster* by Tamkun [5], which can interpret the PTMs on histone proteins. The intricate series of events that lead to gene expression or suppression is regulated by multimeric chromatin remodeling complexes and, among the known four (SWI/SNF, ISWI, CHD, and INO80) [6,7,8,9], the switch/sucrose nonfermentable complex (SWI/SNF) is the center of the attention for its particular involvement in cancer progression [10,11]. Mutations in the SWI/SNF subunits create a dysregulation in gene expression, and they were retrieved in 20% of human cancers [12], confirming the importance of this complex in antitumoral therapy. In particular, specific molecules able to interfere with the activity of BRD9, one of the “reading” subunits contained in human SWI/SNF, prevent gene transcription and expression, reducing the rate of cancer proliferation. BRD9, like the other bromodomains, contains four antiparallel left-handed α-helices (αA, αZ, αB, and αC) and two loops (ZA connecting αA and αZ helices and BC connecting αB and αC helices) [13,14] as shown in Figure 1A.

The peculiarity of this protein, which makes it challenging to target, is that the binding site undergoes a conformational rearrangement upon the interaction with a ligand, affecting particularly the side chains of Phe44, Phe47, and Tyr106 [16].

This binding-derived rearrangement may also explain the dramatic difference in the experimental binding affinity between molecules very similar to each other, as even small variations in scaffold substituent can make the molecule unsuitable for the interaction with the target [11,14,15,16,17,18]. Accordingly, we decided to investigate whether the molecular docking would not be completely reliable in discriminating between good/bad BRD9 ligands and which molecular dynamics parameters would be crucial in guiding the selection process. Moreover, being part of a multimeric complex, BRD9 is relatively small and suitable for extensive molecular dynamics studies that, considering the most recent developments in the in silico calculations, can be carried out in a reasonable time. For this reason, a retrospective analysis of a training set of known BRD9 inhibitors was performed and further assessed on a test set containing both active and inactive compounds.

## 2. Results

**Figure 2:**
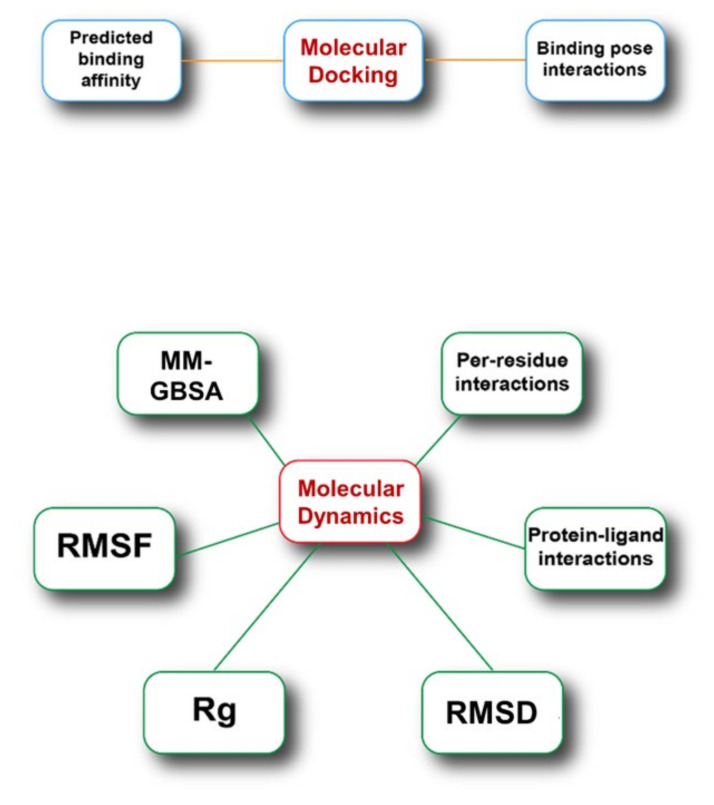
Schematic representation of the parameters analyzed in the process.

**Figure 3:**
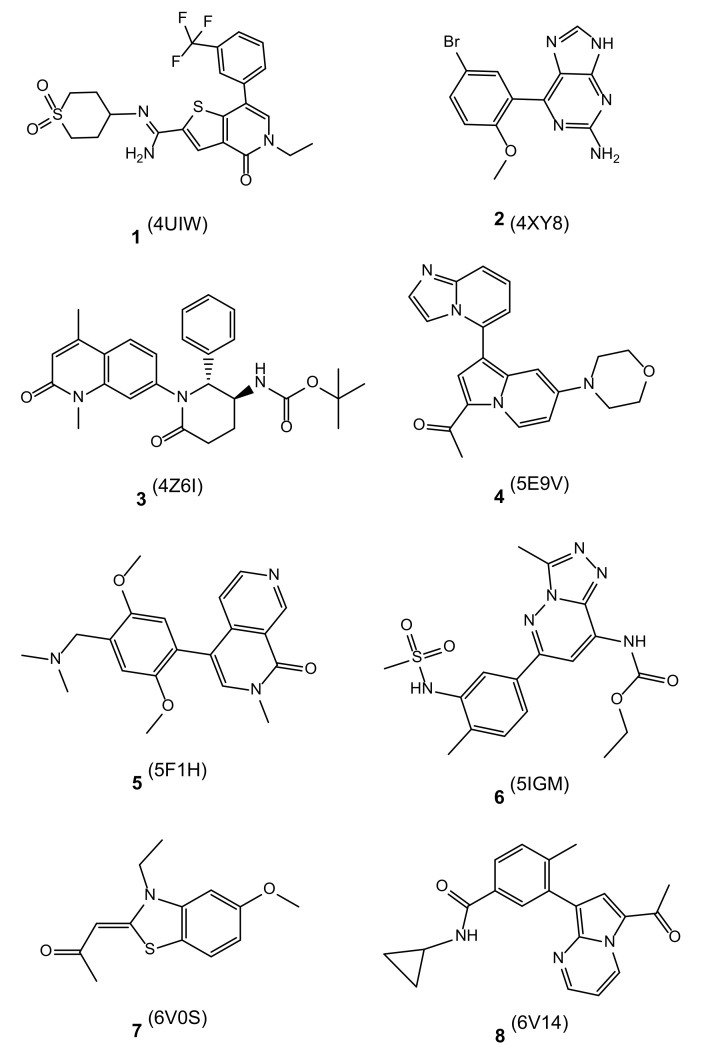
Molecular structure of the co-crystallized ligands **1**–**8** for each complex. The original PDB code related to the specific ligand is reported in parentheses.

BRD9 is considered a key factor in the progression of several types of cancer [12], but the development of new ligands for this protein is not straightforward due to their particular binding modes, which involve the rearrangement of some amino acid side chains. This results in a laborious drug discovery process with a high rate of failures. Computational methodologies can accelerate and guide the discovery and development of new BRD9 binders, helping in discarding compounds that will unlikely make significant contacts with our target. On these bases, to investigate the predictive capability of the sole molecular docking calculations, we performed both molecular docking and molecular dynamics studies on 8 different BRD9 ligand/protein complexes (Figure 2) and one *apo* form of the target and calculated some key structural or energetic parameters which are shown below. For simplicity, the related ligand/protein systems will be mentioned in the following paragraphs considering the original PDB code (**1**–**8**, Figure 3).

Molecular redocking experiments were initially performed to assess the reliability of this methodology in predicting the binding mode and, more importantly, the related binding affinity values. Afterward, we performed three independent molecular dynamics simulations (MD1-3) for each system and analyzed the corresponding structural and energetic parameters.

### 2.1. Molecular Redocking Experiments and Binding Free Energy (MM-GBSA)

The first assessment of the protein-ligand binding was represented by the redocking procedure. The molecule contained in each X-ray crystal structure was docked against the corresponding protein structure to retrieve the exact pose displayed in the 3D structure, and the associated energy was calculated (calculated binding affinities are given in Table 1). If only calculated binding energies were considered, the data would have misguided the potency estimation because the molecular docking energies were not always directly related to the K_D_ [11,16,17,18,19,20,21] (see Table 1). Interestingly, the interaction pattern made by each molecule with the key binding site amino acids (i.e., Phe44, Phe45, Ala46, Val49, Ile53, Tyr99, Asn100, Arg101, and Tyr106; see Figure 1B,C) does not show significant differences that could direct the selection process.

**Figure 4:**
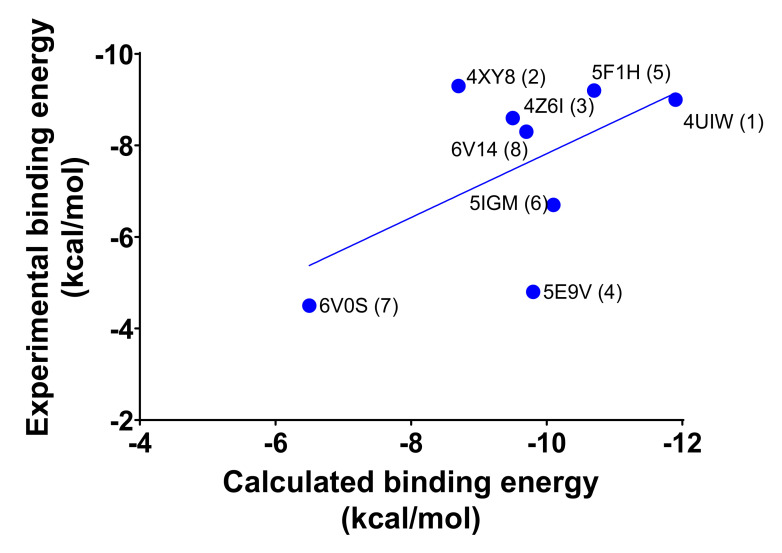
Correlation between the experimental and calculated binding affinities. The values of each complex are labeled accordingly.

Moreover, we derived the binding affinity from the experimental K_D_ using the equation shown below [22]:

(1) $$\Delta G_{\exp}\;=\;\mathrm{lnM}\cdot \frac{\mathrm{RT}}{1000}$$

where M is the K_D_ (M), R is the gas constant (1.987 cal·K^−1^·mol^−1^), and T is the temperature in Kelvin. The calculated linear regression shows an impaired correlation between calculated and experimental binding affinity (Figure 4), with some values very far from the linear function that describes the system. Overall, a minimal correlation between the experimental and calculated binding affinity (R^2^ = 0.31) was detected, but some values deviated greatly from this general trend. Specifically, the calculated binding affinity for **4** (5E9V), **6** (5IGM), and **7** (6V0S) differed by more than 2 kcal/mol from the experimental one [11,17,19,21], which is the first clue that molecular docking cannot be used alone to efficiently predict new BRD9 binders.

Moreover, an ensemble docking experiment was carried out using *apo*-BRD9 due to the benefit that this technique brings to the drug discovery process [23]. In detail, the *apo* form of the protein is simulated for a medium/long time to sample the variation in the three-dimensional structure, and the representative frames are extracted to be used as receptors for molecular docking experiments. In this way, it is possible to mimic a flexible receptor without resorting to an Induced-Fit docking, which is computationally more expensive when dealing with large binding sites. The eight compounds **1**–**8** were docked against 50 representative structures extracted from one *apo*-BRD9 MD, and the calculated docking scores were averaged (Table S1). The results obtained, though, did not match the experimental data, showing a poor docking score and slight differences between each other, making them inadequate for compound ranking.

The binding free energy variation (ΔG_bind_) represents one of the most used parameters to estimate a ligand-protein binding. In this work, the ΔG_bind_ was calculated with the MM-GBSA approach, which is an end-point method based on calculating the difference between the bound and unbound state of the complex [24]. Each trajectory snapshot was clustered into 10 groups based on its RMSD compared to frame 0 and a representative structure for each cluster was chosen (see Materials and Methods). The binding free energy of the 10 representative systems of each simulation was computed using Prime software [25,26,27], and the mean values were reported (Table 1).

Considering the data reported in Table 1, also the average MM-GBSA ΔG_bind_ calculated was not directly related to the K_D_. In particular, **4** (5E9V) showed a non-optimal ΔG_bind_, almost 30 kcal/mol higher than the structure immediately above, namely **6** (5IGM), but a K_D_ value that did not differ significantly from the latter. However, it remains clear that the ΔG_bind_ from MM-GBSA cannot be the only parameter to be considered to estimate the quality of protein-ligand binding. Therefore, we evaluated other structural and energetic parameters (namely RMSD, RMSF, protein-ligand interactions, and per-residue energetic contribution, *vide infra*) from the MDs, along with data regarding the BRD9-*apo* structure, to highlight which could represent key components in the binding evaluation.

### 2.2. RMSD

At first, the RMSD of each complex during the three replicas was evaluated considering the deviations in the backbone atoms. In this way, it was possible to have a preliminary idea about the stability of the system throughout time, taking the first frame as the reference structure.

**Figure 5:**
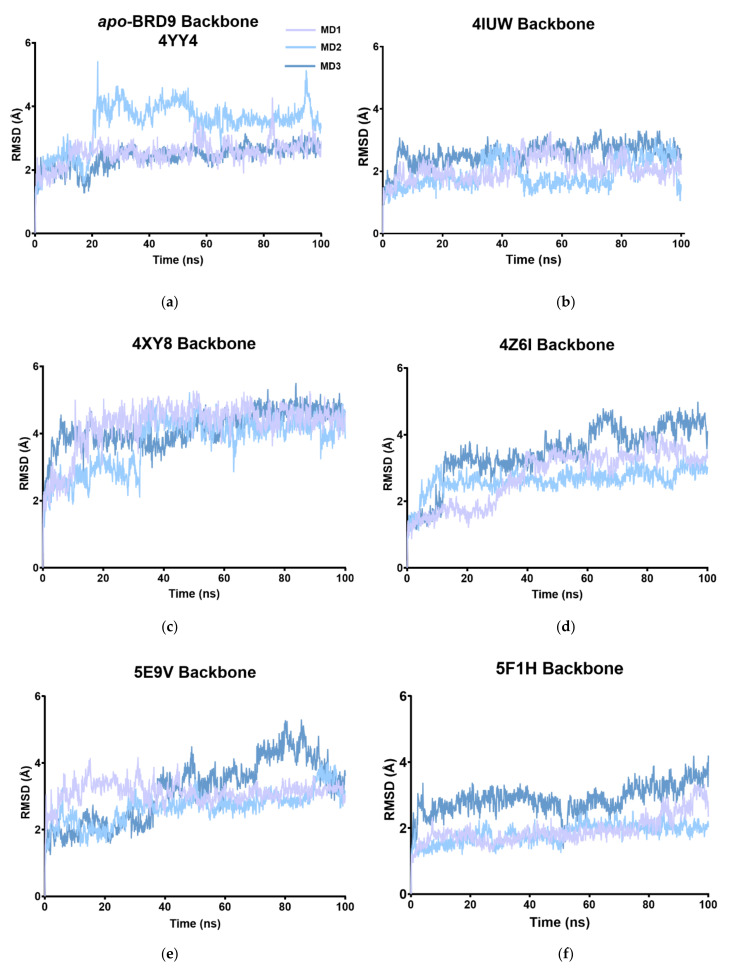

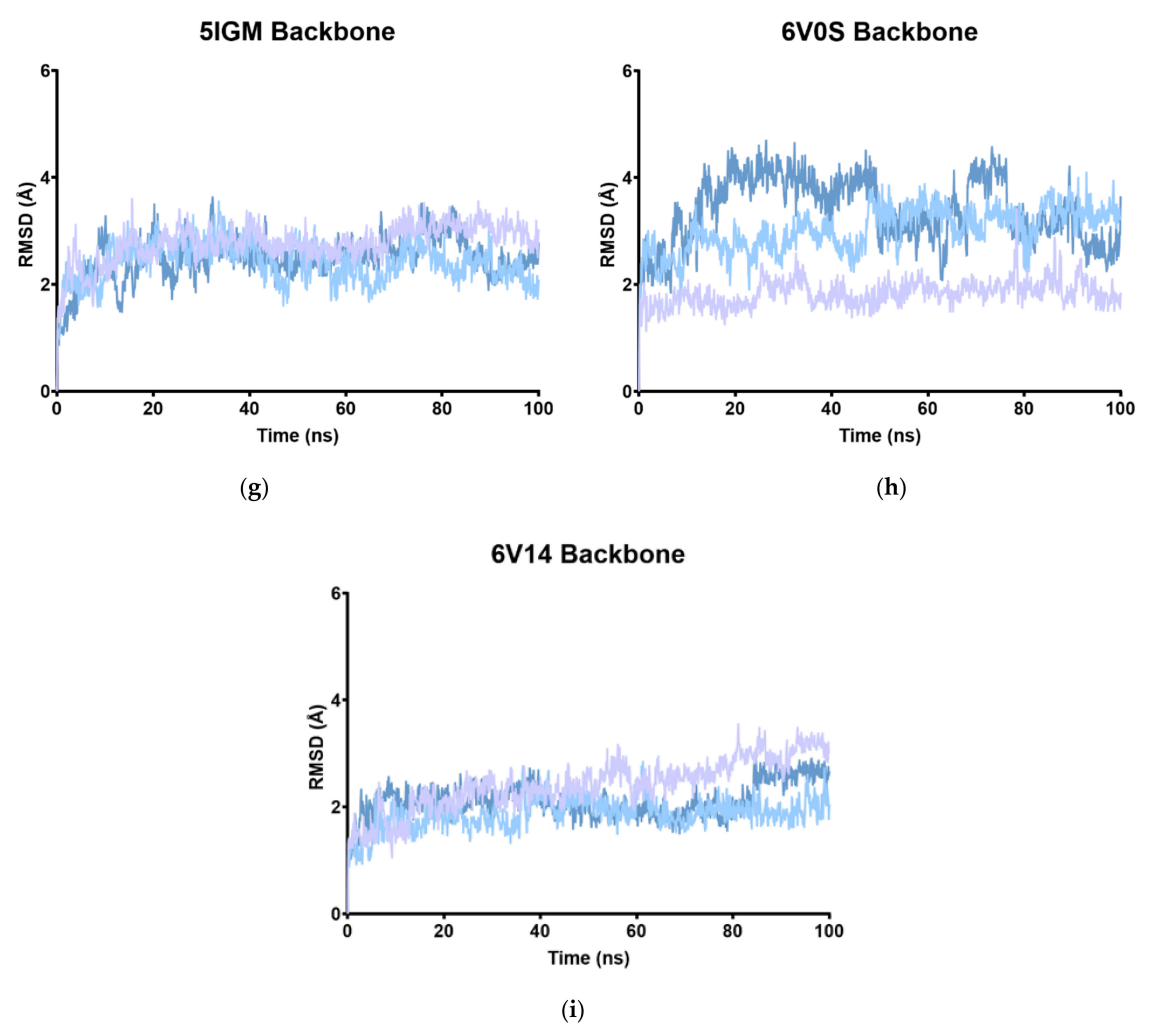
(**a**–**i**) RMSD of the backbone atoms of the nine systems referred to the original system. Each simulation was run independently three times, specifically for (**a**) *apo*-BRD9, (**b**) 4UIW, (**c**) 4XYZ, (**d**) 4Z6I, (**e**) 5E9V, (**f**) 5F1H, (**g**) 5IGM, (**h**) 6V0S, (**i**) 6V14.

Generally, all the systems considered tended to reach an equilibrium after 25 ns of simulation. The *apo* form of the protein showed an average RMSD value that remained almost constant throughout time (Figure 5 and Table 2). The global trend of the protein-ligand complexes was a narrow variation in the backbone RMSD and an average value sometimes lower than the *apo*-BRD9, indicating a discrete level of stability of the complexes. The 4XY8 system resulted largely unstable if compared to the unbound protein structure, with an average RMSD of 4.05 Å, and some instability signs, like small peaks above 4 Å, could be found also in 5E9V and 6V0S.

This preliminary data analysis showed that 4IUW, 5F1H, 5IGM, 6V0S, and 6V14 systems underwent fewer changes in the overall structure during the simulation time, and it may roughly indicate a more stabilized protein-ligand complex.

In addition, to understand whether a natural conformational variation of protein (thus, on the *apo* structure) may facilitate the interaction with a small molecule, we compared the binding site of each protein-ligand crystal with the snapshots taken from the three *apo*-BRD9 MDs. The residues considered were Gly43, Phe44, Phe45, Phe47, Pro48, Val49, Ile53, Ala54, Pro55, Tyr57, Met92, Asn95, Ala96, Met97, Tyr99, Asn100, Arg101, Thr104, and Tyr106. Among those, Phe44, Phe45, Ala46, Val49, Ile53, Tyr99, Asn100, Arg101, and Tyr106 (Figure 1C) were considered crucial for the binding. The RMSD of each crystal structure from all the 3000 snapshots of the *apo*-BRD9 was calculated, the results were sorted from the smallest to the greatest, and a threshold of 2.5 Å was set to filter the results. A total of 192 snapshots passed the filtering step, and the corresponding percentage (out of the 3000 frames) was reported (Table 3).

Interestingly, during the MD simulations, the binding site of *apo*-BRD9 frequently resembled 6V0S (Table 3), indicating that the unbound protein can naturally take a conformation that would facilitate the binding with the small molecule reported in this complex, suggesting a positive binding of its ligand to the target protein.

### 2.3. RMSF

We moved on with the evaluation of which residues or areas of the protein were involved in the different mobility highlighted by the RMSD, as there is an obvious connection between the high RMSD reported in some complexes and the fluctuation of its residues. The RMSF value is important to establish the fluctuations of residues side chains during the molecular dynamics simulations. A high RMSF value in the binding site amino acids indicates that such residues do not make strong interactions with the small molecule and, therefore, the binding is not optimal.

**Figure 6:**
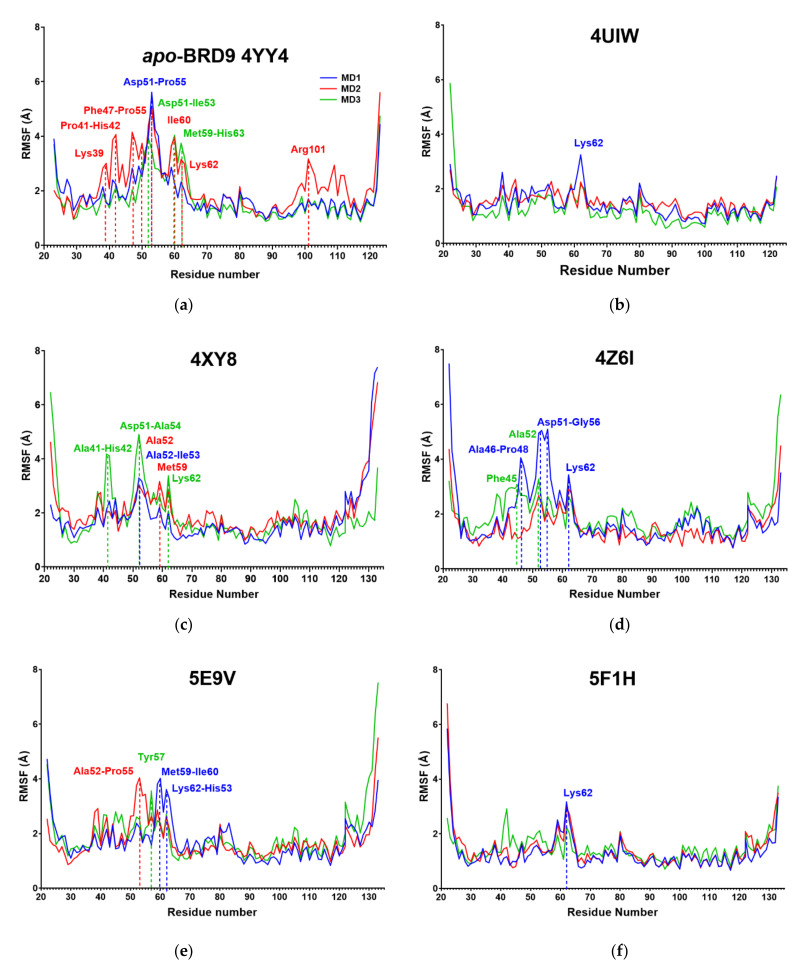

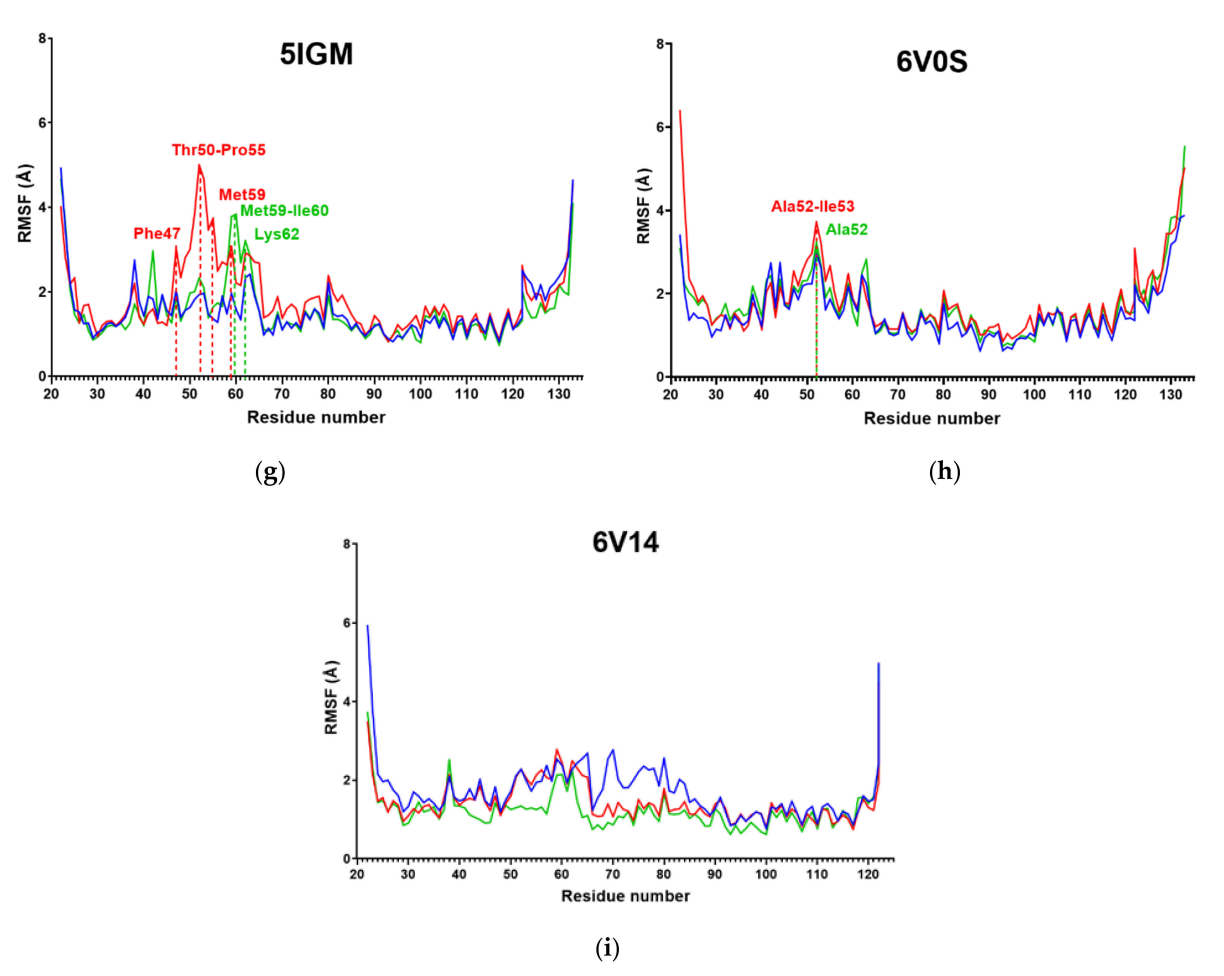
(**a**–**i**) RMSF of the 9 systems. Each simulation was run independently three times and the data reported are the mean of the backbone and side chains values. Residues with an RMSF above 3.0 Å are labeled, specifically for (**a**) *apo*-BRD9, (**b**) 4UIW, (**c**) 4XYZ, (**d**) 4Z6I, (**e**) 5E9V, (**f**) 5F1H, (**g**) 5IGM, (**h**) 6V0S, (**i**) 6V14.

The *apo*-BRD9 showed predictable mobility of the amino acids forming the binding site, particularly the segment Lys39-Lys62 [18] (Figure 6 and Table 4). This trend was reverted in 4UIW, 5F1H, 6V0S, and 6V14 systems, while some spikes remained in the other protein-ligand complexes. If the mean values of the three MDs were considered, only 4XY8 and 4Z6I showed binding site residues with a fluctuation above 4 Å (Table 4).

The analogy between the RMSD and the RMSF observed in 4UIW, 5F1H, 6V0S, and 6V14 corroborates the hypothesis that these complexes are strongly stabilized by the interaction with their ligand.

### 2.4. Radius of Gyration

**Figure 7:**
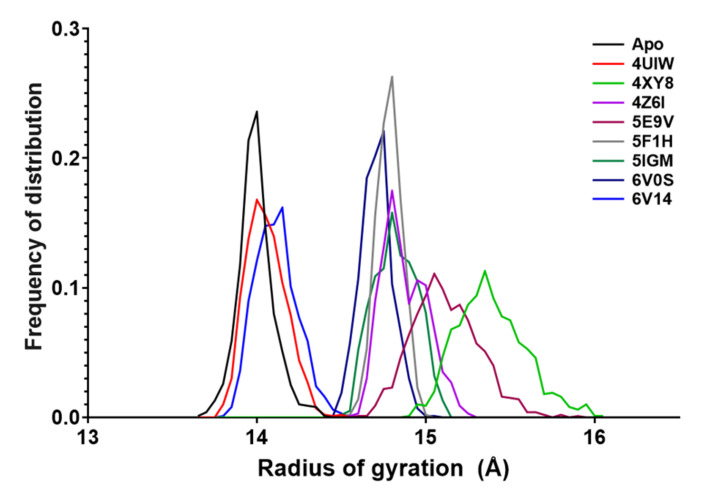
The radius of gyration of the 9 systems. The data were collected as the mean value at each timestep and plotted as the frequency of distribution.

The radius of gyration (Rg) is defined as the root mean square deviation of the atoms from the protein centroid and can provide further information about the stability and compactness of the protein during the MD experiments. For each simulation of the 9 complexes, we averaged the values computed in each timestep, and the corresponding frequency of distribution is reported in Figure 7.

Despite the different chain lengths of the considered structures, the data showed a range of variation in Rg values less than 1.5 Å for all the systems (Table 5, “range”), making them comparable to the *apo*-BRD9. It is interesting to observe how 5F1H (grey line, Figure 7) showed very little variations in the frequency distribution of the values, generating a single high peak. The general trend was a sharp curve with most of the values grouped around the mean value, and only a few exceptions, like 5E9V and 4XY8, had wider curve trends.

### 2.5. Protein-Ligand Interactions

**Figure 8:**
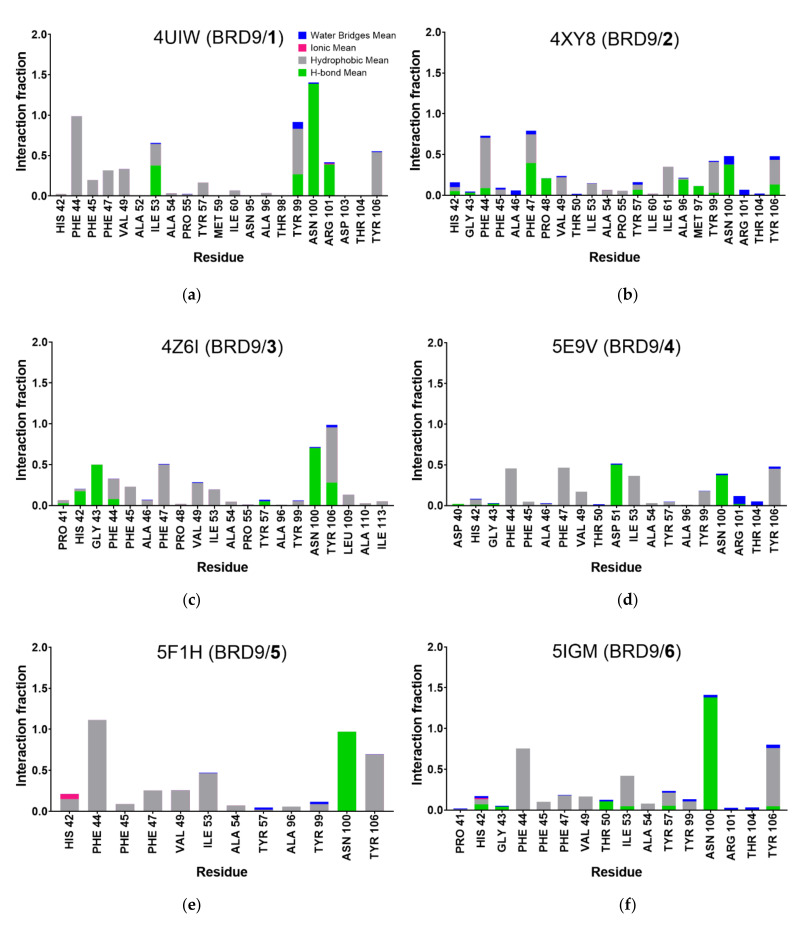

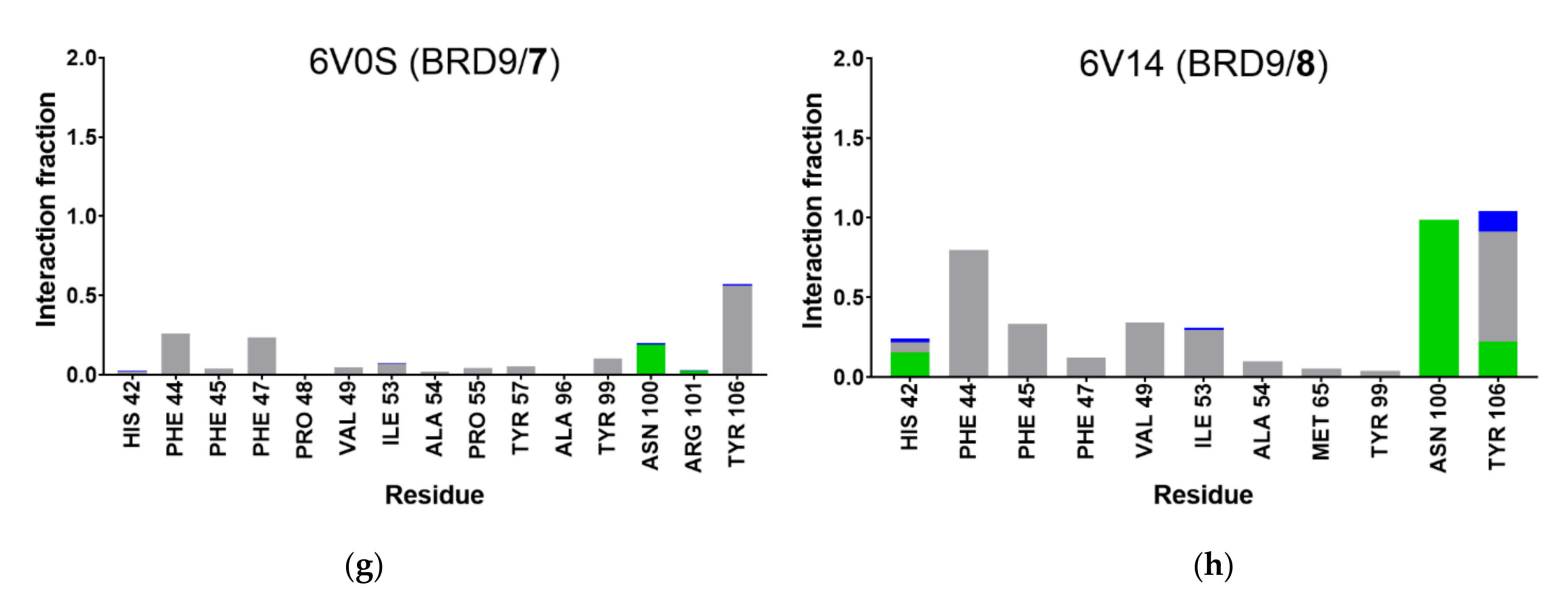
(**a**–**h**) Protein-ligand interactions of each ligand/protein complex. Values reported are the mean of the three replicated MDs and they are expressed as the fraction of total time. Values above 1.0 are due to the cumulative effect of the various type of interactions, specifically for (**a**) 4UIW, (**b**) 4XYZ, (**c**) 4Z6I, (**d**) 5E9V, (**e**) 5F1H, (**f**) 5IGM, (**g**) 6V0S, (**h**) 6V14.

The interactions made by the investigated ligand with the target were monitored during the three simulations, and the data were averaged to obtain the mean value for each residue. In Figure 8 we plotted such values, highlighting the contribution of each type, expressed as the fraction of the total simulation time (i.e., the ratio between the duration of the contact and the total length of the MD). If a single type of contact has an Interaction Fraction above 1.0, like in 4UIW, 4Z6I, 5F1H, and 5IGM systems, it means that the residue has multiple interaction points that are considered as separate entities and their contributions are cumulative.

As expected, compounds **1** and **5** showed a strong interaction pattern with the binding site amino acids while **7** made irrelevant contacts with the protein counterpart, in contrast with what could be hypothesized from the RMSD and RMSF analysis. The strong interaction made by **6** with Asn100, known to be a key residue in BRD9 [14,18], represented another peculiar aspect that can suggest an average protein-ligand binding. The main interaction types appeared to be hydrogen bond and hydrophobic contacts due to the chemical nature of the compounds (Figure 3), which contained extended aromatic moieties and several hydrogen bond donors/acceptors.

### 2.6. Per-Residue Interaction

**Figure 9:**
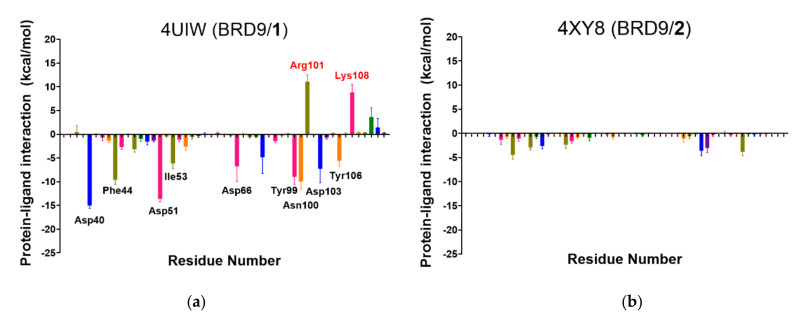

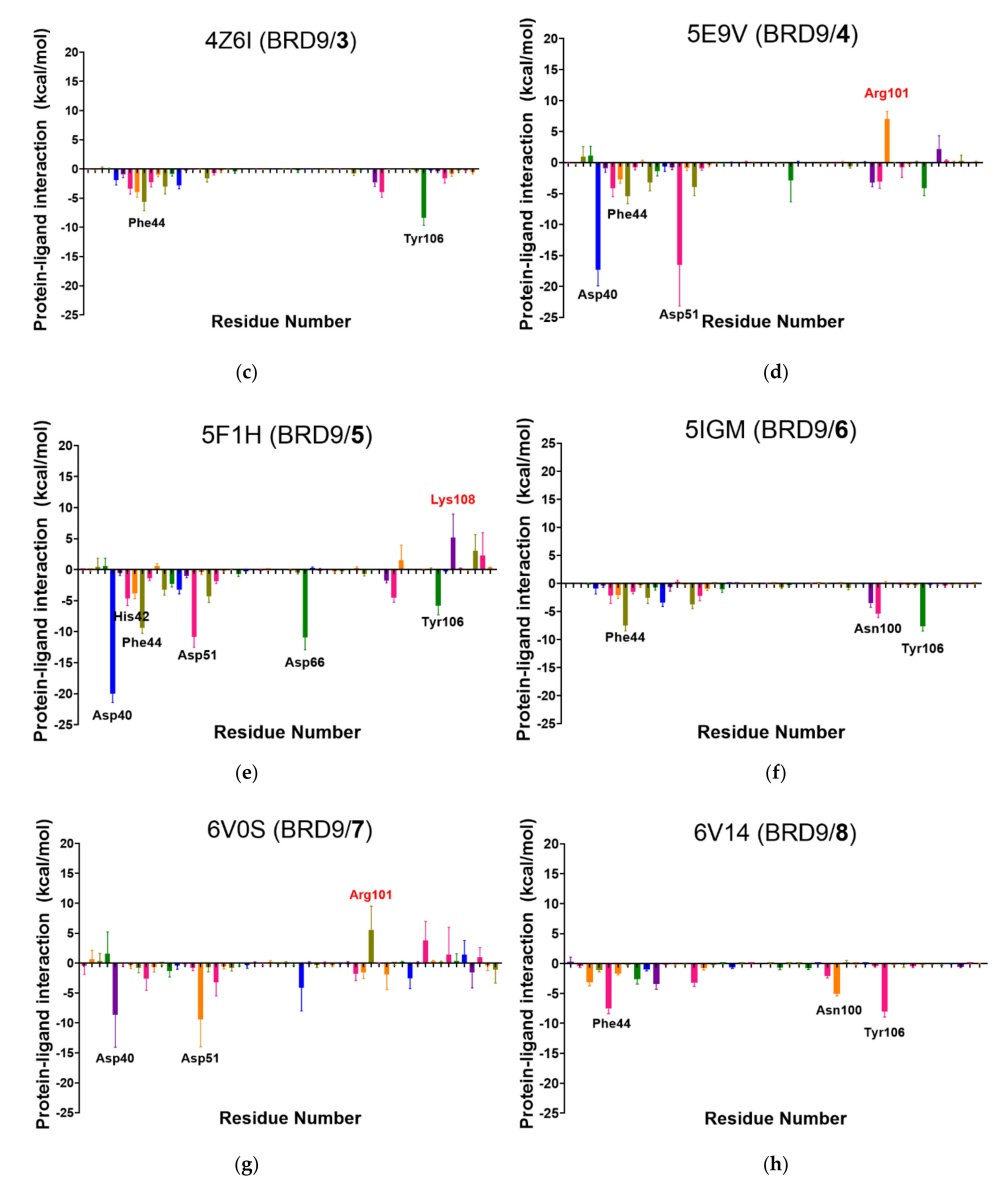
(**a**–**h**) Per-residue interactions of the 8 complexes reported as the mean value of the three replicated dynamics. Residues with energetic values below −5.0 kcal/mol are labeled in black and residues above +5.0 kcal/mol are labeled in red, specifically for (**a**) 4UIW, (**b**) 4XYZ, (**c**) 4Z6I, (**d**) 5E9V, (**e**) 5F1H, (**f**) 5IGM, (**g**) 6V0S, (**h**) 6V14.

It also appeared interesting to acknowledge the energetic contribution of each residue to the interaction with the small molecule; therefore, the binding energy was decomposed in its single components, and the mean value of each residue was reported (Figure 9).

This analysis gave us a deeper understanding of the role played by each residue in the binding with its small molecule. The results highlighted that **1** and **5** have a strong protein-ligand interaction, with several residues showing interaction energy values below −5.0 kcal/mol that compensated the disruptive contribution energies of some residues (Figure 9), in perfect accordance with the hypothesis formulated during the analysis of the previous parameters. Therefore, disruptive energetic contributions involving key binding site residues, like in the case of **1, 4**, and **7**, may indicate a destabilization of the BRD9/ligand binding and must be taken into account in the prediction.

### 2.7. Summarizing All the Data Analysis

Each parameter derived from the molecular docking and MD simulations was evaluated separately and classified as “good” (green), “intermediate” (yellow/orange), or “bad” (red); the threshold values reported below were then set to match the experimental affinity ranking scale; the results are reported in Table 6. It is worth noting that docking score-derived information reflects a static picture of the binding event. Specifically, the interaction patterns detected with this method (Table 6) are not sufficiently different from each other and, accordingly, they cannot represent discriminative factors for separating “good” binders from “bad” ones.

In detail:

- The docking score, the MM-GBSA energy, per-residue total energy, and per-residue binding site energy are colored according to their value, i.e., the best value in green (i.e., −9.3 kcal/mol), the worst (i.e., −4.4 kcal/mol) in red, and the others in green-to-red gradient.
- The number of interactions reported represents the amount of key binding site residues that interact with the ligand for at least 50% of the total time. Good values are above or equal to 3.
- The RMSD is classified according to the average value of the *apo* structure (2.79 Å). Values below 2.6 Å are considered good, values between 2.6 and 3.0 Å are considered intermediate because they resemble the unbound state of the protein, and values above 3.0 Å are classified as bad ones.
- The RMSF column reports the binding site residues with an average fluctuation value above 4.0 Å. If more than 2 residues show this behavior, the parameter is considered not favorable.
- The radius of gyration is discriminated according to the width of the *apo*-BRD9 curve (0.72 Å). Values below this threshold are considered positive.

From the analysis of the data reported in Table 6, it was clear that no parameter could be selected as a unique determiner of the binding between a small molecule and its target. The proposed analysis cannot determine the experimental binding affinity, but it is useful to give a general ranking when multiple ligands are available and to highlight the most promising compounds in a small set of molecules. In general:

- if the MM-GBSA, the per-residue contributions (both total and binding site), and the interactions are favorable, the compound considered is in the top part of the ranking (“Good” in Table 6).
- If the per-residue contributions are not satisfactory, but the overall interactions are encouraging, the compound is likely to belong to the middle part of the ranking (“Intermediate” in Table 6).
- Despite the MM-GBSA value, if both the per-residue contributions and the interactions made are not sufficient, the compound is placed among the worst in the final ranking (“Bad” in Table 6).

The per-residue energy contributions reported are useful to discriminate the role played by the binding site residues and the total variation of energy caused by the protein-ligand contacts. In some cases, like 5IGM (**6**), the binding site per-residue energy is even above zero, but this destabilizing energetic component is mitigated by the total interaction energy. If the total and the binding site per-residue energy coincide, like in 4Z6I (**3**), 5IGM (**6**), and 6V14 (**8**), it means that the whole binding energy involves the binding site key residues and, despite being of modest entity, this suggests that the molecule is a good binder. The binding prediction that can be made out of these data reflects the experimental binding values; the best compound is correctly identified, and the mediocre/bad binders are properly classified. One may find unexpected the “intermediate” classification given to 5E9V (**4**), especially considering the positive binding site per-residue energy and the lack of interactions with the key amino acids. This can be explained considering that the MM-GBSA ΔG_bind_, the absence of out-of-range fluctuations, and the total per-residue energy contribution (caused by the interactions made with non-fundamental amino acids [17]) mitigate the unfavorable outcomes of the per-residue energy and the modest protein/ligand interactions and result in a prediction in line with the experimental K_D_. From this analysis, 4Z6I (**3**) is the only misplaced protein-ligand complex, due to its per-residue interactions energy, that is entirely derived from binding site residues contributions, and a discrete MM-GBSA ΔG_bind_ was classified as “intermediate” as 6V14 (**8**), which shows a very similar analysis outcome. Moreover, to evaluate if 100 ns were enough in this type of study, we extended the 4UIW MD1 reaching a total simulation time of 1 µs. Interestingly, the analysis of the results obtained in this way (see Supplementary Materials Figures S1–S5) did not deviate significantly from the results obtained in 100 ns of simulation. These considerations suggest that a total simulation time of 100 ns is sufficient to study the protein-ligand complex behavior and this confirms that the proposed method is both quick and reliable.

### 2.8. Test Set

**Figure 10:**
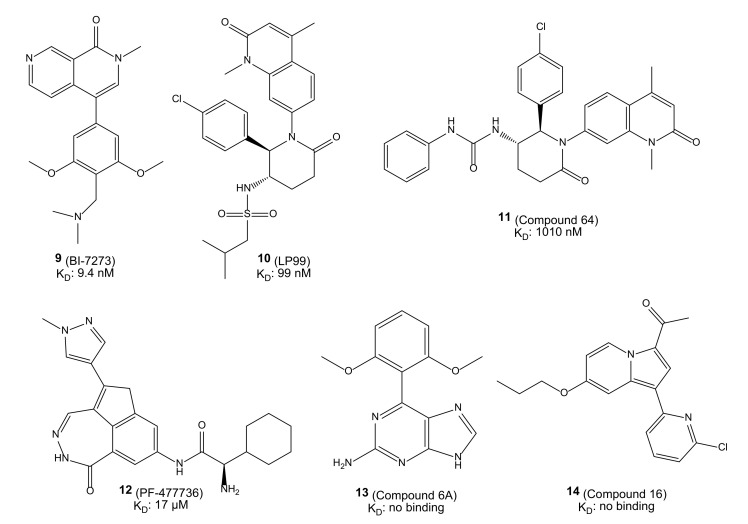
Chemical structures and K_D_ values of the six compounds **9**–**14** used in the test set.

To test the proposed methodology, six known BRD9 ligands [11,16,17,19] with different affinities for the target, ranging from low nM to high μM values, as well as no-binders, were selected (Figure 10).

**Figure 11:**
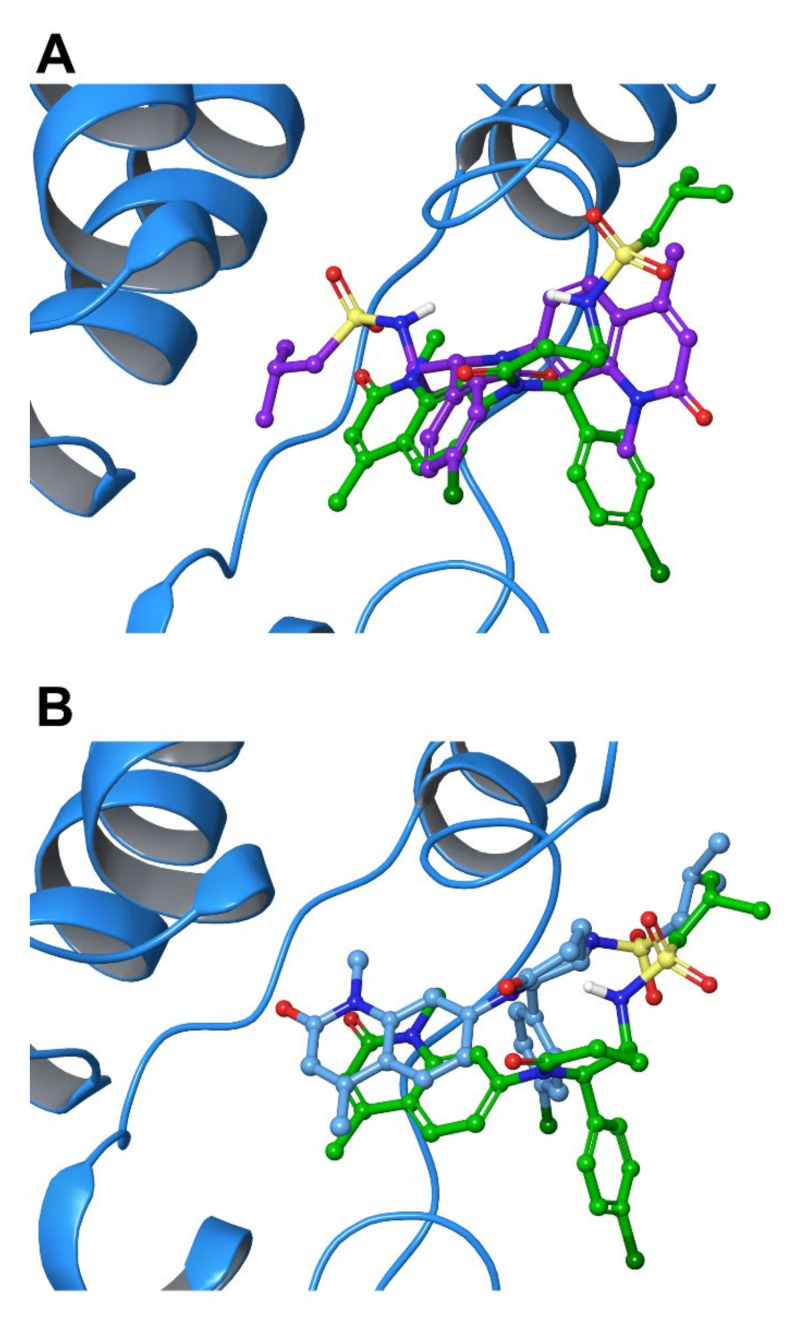
(**A**) Comparison between the two poses of **10** (**10a**, purple carbons, and **10b**, green carbons) in BRD9 binding site (cyan ribbons). (**B**) Superimposition between the good pose of **10** (green carbons) and its crystallized binding mode (cyan carbons).

To mimic a real case study, where no structural information is available, the ligands were first submitted to a molecular docking calculation to select the best binding pose. The six molecules were docked against BRD9 (PDB code: 5F1H) using Glide [28,29,30,31] in Extra Precision (XP) mode, saving 10 maximum poses for each compound. The binding poses were evaluated, separately considering both the docking score and the interactions made with the protein, and in five cases out of six the best ranked pose according to the docking score corresponded to that establishing the best interaction pattern. Interestingly, when the binding mode of **10** was analyzed, the pose that showed the best protein-residue interactions (i.e., Asn100 and Tyr106) was the second one if considering the docking score as the ranking parameter (Figure 11A). In this case, we decided to verify whether an incorrect evaluation in the pose choice could lead to wrong results and, for this reason, we analyzed both the poses returned by Glide (named from now on **10a** and **10b**).

Initial important considerations should be made on the docking score because, from the mere analysis of the calculated binding affinity, some inconsistencies with the experimental data are clear (Table 7). In particular, compound **10**, which has a good affinity for the target (K_D_ = 99 nM) poorly performed in molecular docking experiments. On the other hand, **14** featured a good predicted binding affinity, but it was reported to have low/no interaction with BRD9. This again confirmed, on BRD9, that an analysis based only on the molecular docking could lead to false positive and false negative results and a deeper testing phase was necessary to evaluate the binding more appropriately.

The complexes were then simulated in a single MD as before (see Section 4) and the analysis was carried out as outlined in the previous steps. The MM-GBSA was the starting point to discriminate good binders from poor ones. Like in the training set, the MDs were clustered into 10 groups according to their RMSD from frame 0 (see Materials and Methods), and the corresponding ΔG_bind_ for the representative structure of each cluster was calculated. The values are reported as the mean of the 10 values (Table 7). As in the training set, the correlation between experimental binding affinity and MM-GBSA was not perfect but, interestingly, **10a** showed the worst ΔG_bind_, following the docking score, despite being the second-best binder in the set. Conversely, **10b** showed a much better ΔG_bind_ maintaining an almost unchanged docking score, suggesting that the correct binding pose is fundamental in this type of experiment.

**Figure 12:**
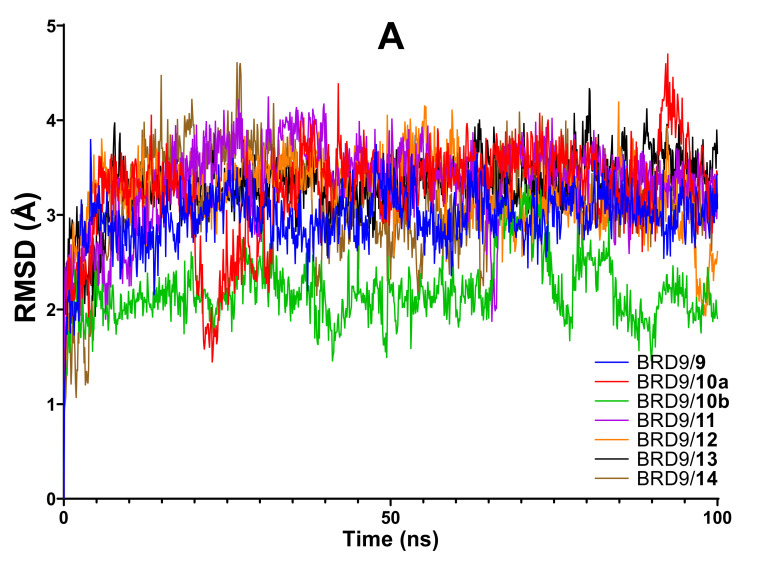

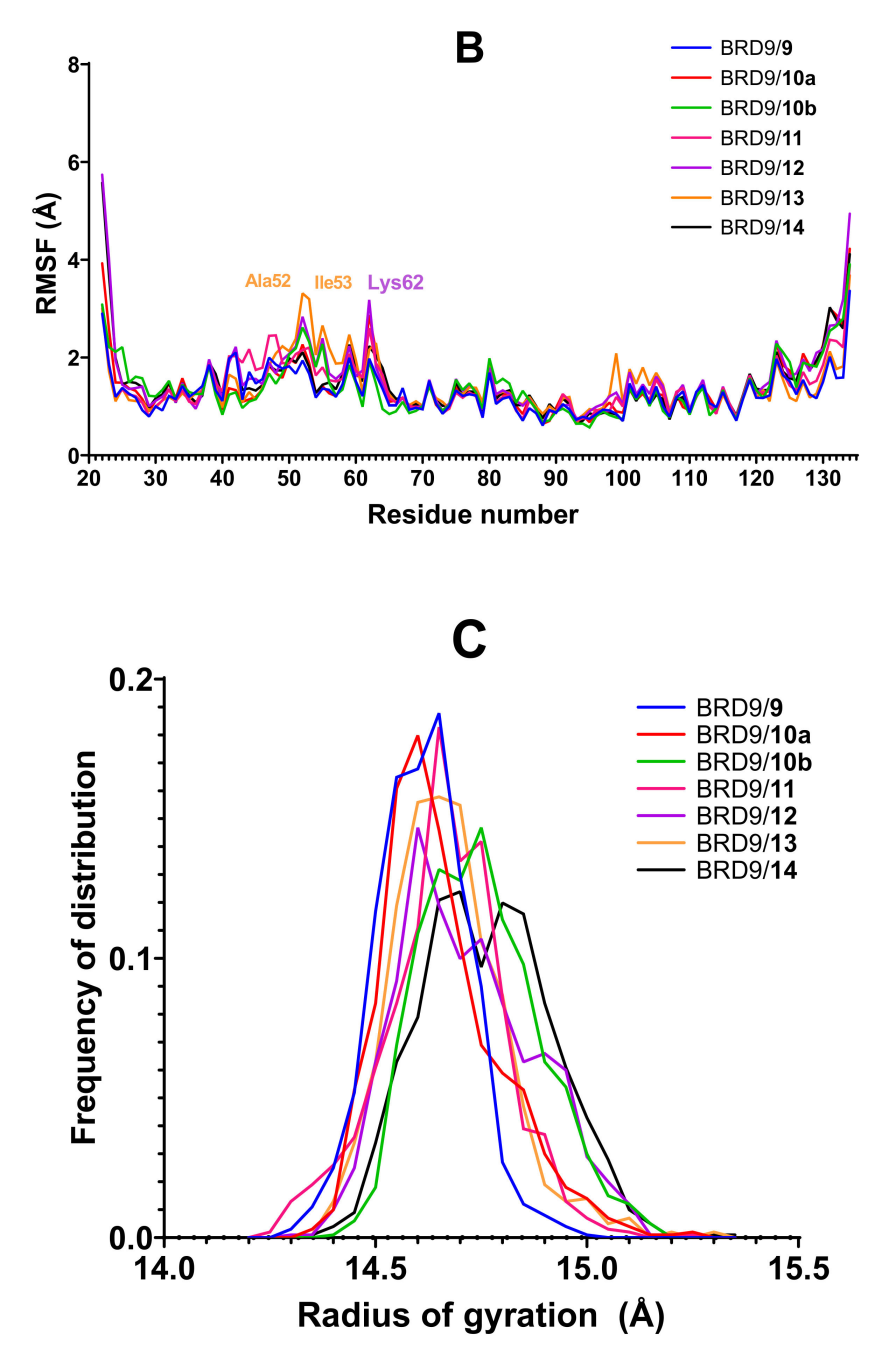
(**A**) RMSD, (**B**) RMSF, and (**C**) Rg analysis of the six protein-ligand complexes.

As predicted, the analysis of the most common and immediate MD parameters (RMSD, RMSF, and Rg) did not provide useful information to discriminate more and less active compounds (Figure 12A). In detail, no complexes showed RMSD values above 4 Å, except for a spike in the trend of **10a** and **14** which, however, could not indicate a significant instability in the complex.

In addition, the RMSF analysis showed no relevant fluctuation, excluding Ala52 and Ile53 (belonging to the binding site) in **13** and Lys62 in **12**, indicating instability in connection with these ligands (Figure 12B). The radius of gyration of the first five proteins followed a similar pattern, with the curves being narrow and all centered within 14.60 and 14.75 Å. The system related to compound **14**, instead, showed a double-peaked trend and the curve was shorter and wider than the others. The lack of compactness reported may suggest that BRD9/**14** is a less stable complex (Figure 12C). Nevertheless, based only on these data, one could not discriminate correctly the molecules tested.

**Figure 13:**
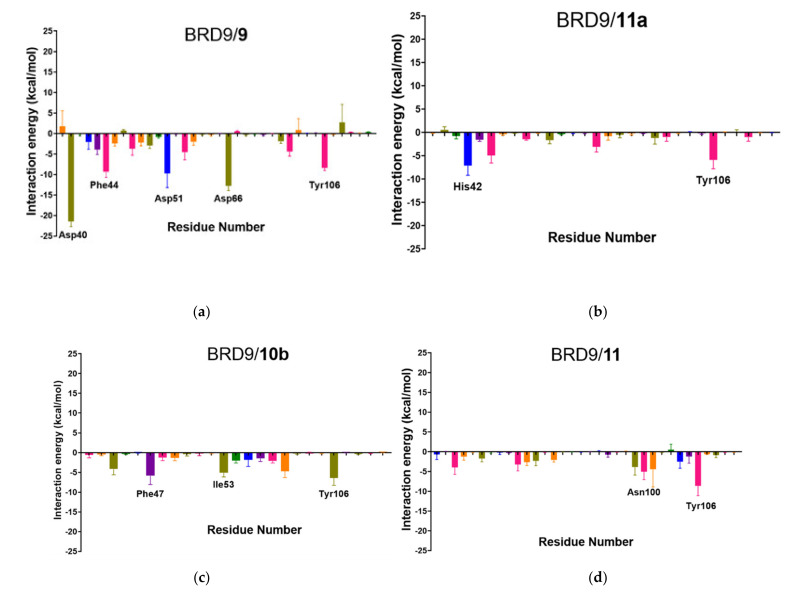

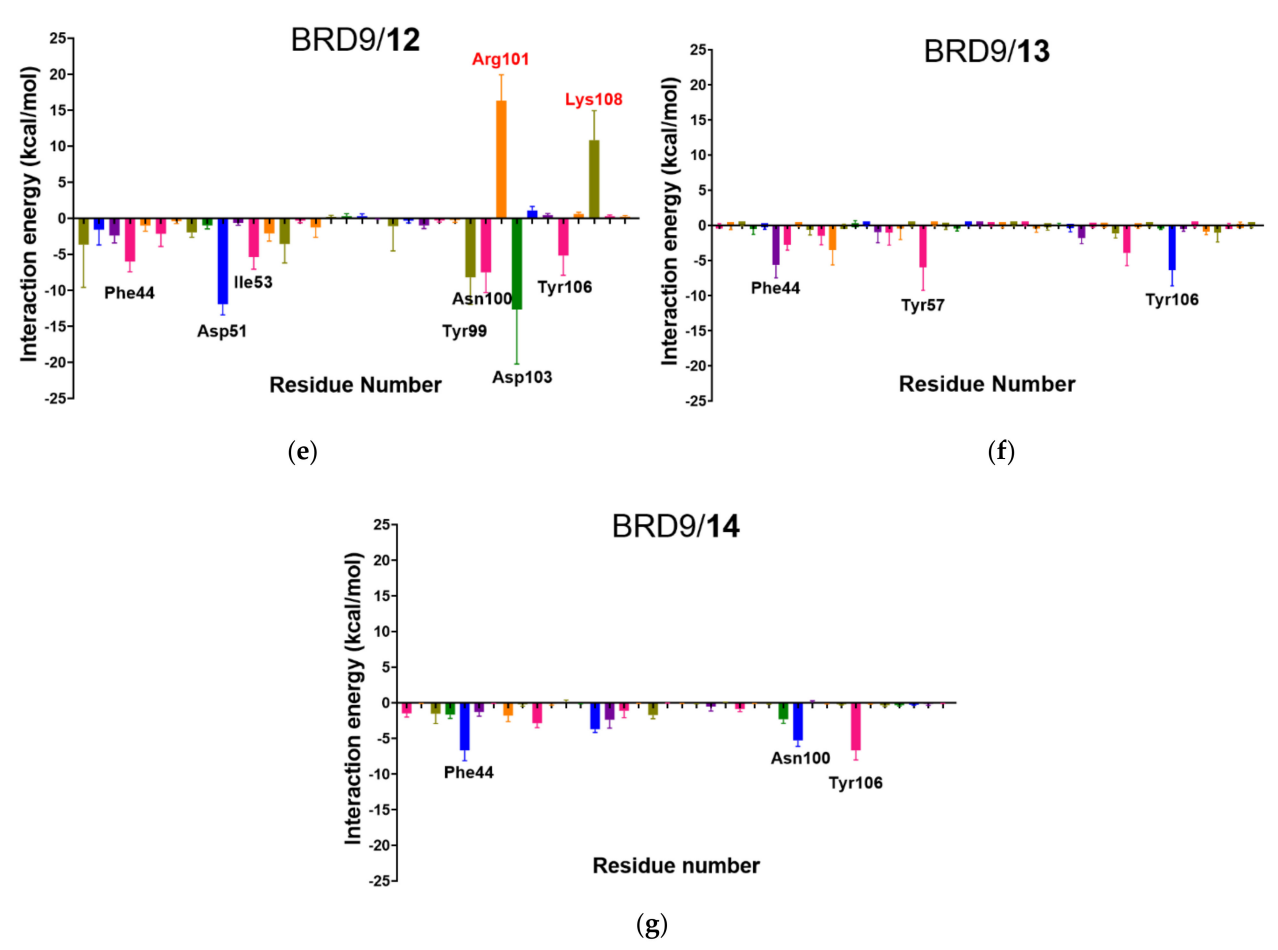
(**a**–**g**) Per-residue interaction of the six complexes. Residues with energetic values below −5.0 kcal/mol are labeled in black and residues with values above +5.0 kcal/mol are labeled in red, specifically for(*a*) BRD9/*9*, (*b*) BRD9/*10a*, (*c*) BRD9/*10b*, (*d*) BRD9/*11*, (*e*) BRD9/*12*, (*f*) BRD9/*13*, (*g*) BRD9/*14*.

**Figure 14:**
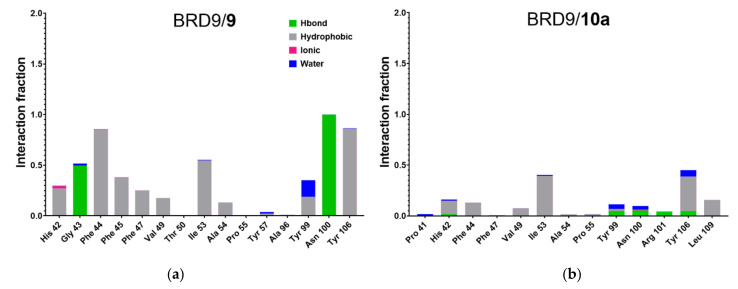

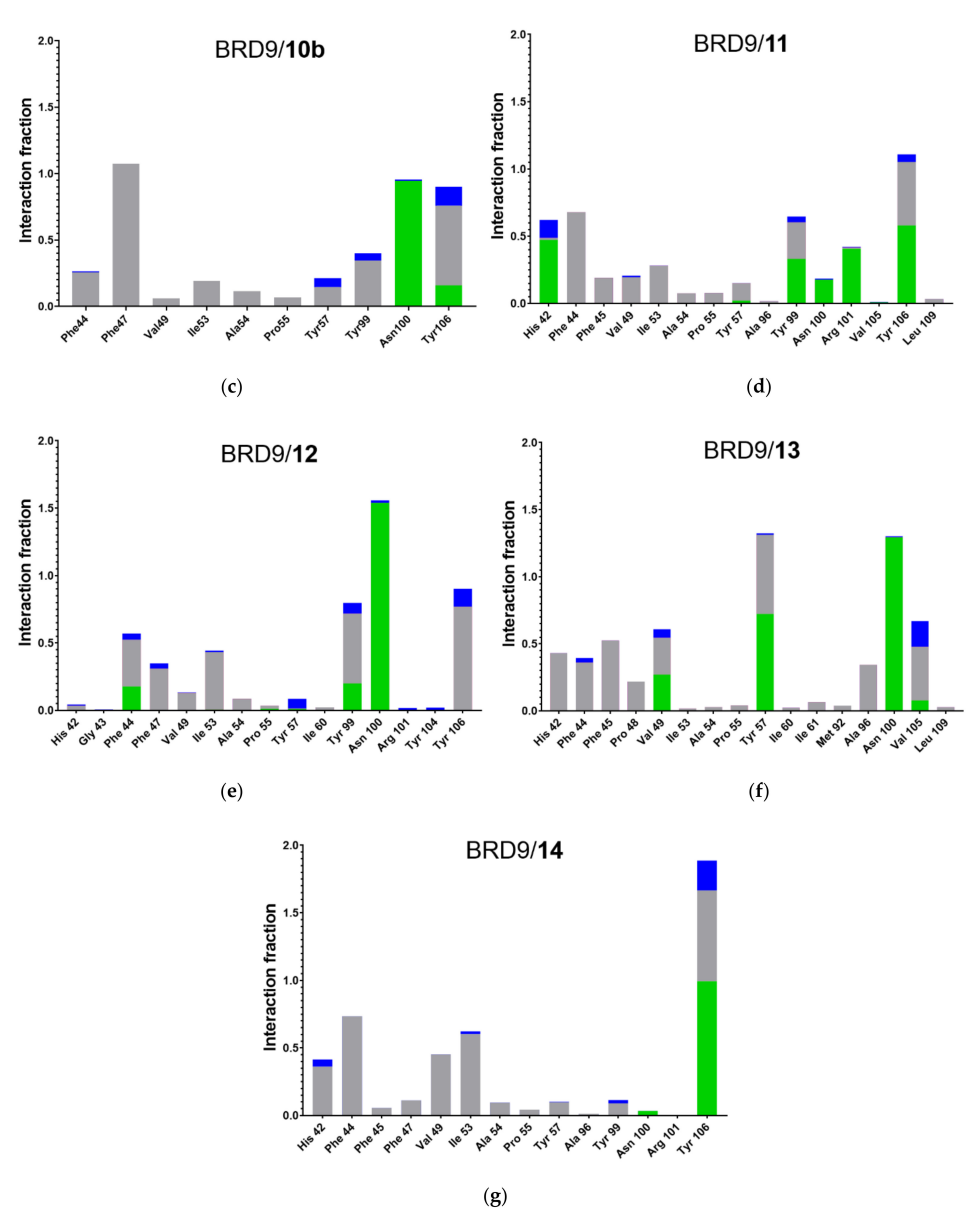
(**a**–**g**) Protein-ligand interaction histogram of the six complexes expressed as total time fraction. Values above 1.0 are the result of the cumulative effect of multiple interaction types or hydrogen bond donors/acceptors, specifically for (*a*) BRD9/*9*, (*b*) BRD9/*10a*, (*c*) BRD9/*10b*, (*d*) BRD9/*11*, (*e*) BRD9/*12*, (*f*) BRD9/*13*, (*g*) BRD9/*14*.

The analysis of the per-residue interactions (Figure 13) provided, as expected, a useful insight into the behavior of each molecule. Specifically, compound **9** received the strongest energetic contribution from Asp40, which, despite not being a key residue in the binding site of BRD9, had interaction energy around −20 kcal/mol; moreover, Phe44 and Tyr106, two crucial binding site amino acids, also showed considerable binding energy. The only relevant energies reported for **11** were computed for Asp100 and Tyr106, while the chaotic and unpaired situation of **12** indicated an unstable binding, particularly due to the high repulsive force generated by Arg101, one of the key residues of the binding site. The binding energies involving other key amino acids were modest and could not counterbalance the two massive disruptive contributions. As assessed before, **10a** performed poorly with irrelevant binding energies, following the trend highlighted in the previous steps. This confirmed that the starting binding pose is fundamental, and a wrong choice can greatly influence the predictions. Indeed, **10b** shows good binding energies with binding site amino acids, namely Phe47, Asn100, and Tyr106. The two non-bonding compounds (**13** and **14**) showed a mediocre binding with Phe44, Asn100, and Tyr106, making them comparable with poor binders like **11** or **12**, despite **14** having a docking score of −7.94 kcal/mol. Concerning the protein-ligand interactions, the trend highlighted was similar to per-residue interaction energies (Figure 14).

As expected, **9** showed a stable interaction pattern with key amino acids (Phe44, Ile53, Tyr99, Asn100, and Tyr106) throughout the whole dynamic (Figure 14), suggesting being the compound with the best activity. Compounds **11** and **12**, on the other hand, showed an opposite trend in the interactions with Asn100, which was involved in the binding for less than 0.5 and more than 1.5 fraction of time, respectively (due to the double hydrogen bond donor/acceptor on the side chain). The other amino acids were only marginally involved in the binding, with a slightly better performance of **12** compared to **13**. Interestingly, **10a** made almost no connection with BRD9, corroborating the hypothesis that the starting binding pose is fundamental to obtain reliable outcomes. A summary of the analysis performed is reported in Table 8.

The poor performance of **10a**, in stark contrast with **10b**, confirmed that the choice of the correct docking pose, from which the MD will be started, can influence the results up to provide false-positive or false-negative results. This often happens because a canonical MD simulation only explores the local energetic minimum and does not have either the time or the “strength” to explore the energetic surface, suggesting the use of alternative methods (e.g., metadynamics) in these uncertain cases. Concerning the other molecules, the trend is generally preserved, and **9** is by far the molecule with the best performance.

## 3. Discussion

In the case of BRD9 protein, semi-rigid molecular docking experiments and related data only may not be exhaustive for the selection of promising ligands. This finding is in agreement with previous reports, where the use of an Induced Fit docking was necessary to accommodate the small molecule properly in the binding site [16]. In detail, after a preliminary redocking step, we have here shown the correlation between the calculated and the experimental binding energy was not perfect, indicating that an in silico study should not be based only on this type of experiment. Therefore, an extensive retrospective MD study was carried out to assess which MD-derived parameters could be combined to formulate a reliable binding hypothesis. From the analysis of the data reported, it was possible to highlight the key MD parameters to be considered in the evaluation of a small set of possible BRD9 binders. In detail, common structural parameters like RMSD, RMSF, and Rg were proved to be not sufficient to point out the best binders correctly and are not directly related to the experimental K_D_. The energetic parameters, on the other hand, showed a deeper and more useful insight into the nature of the binding. The per-residue interactions and the MM-GBSA ΔG_bind_ were particularly crucial in evaluating the contacts between the target and the small molecule it contained. The molecular docking/molecular dynamics procedure was validated using a set of six molecules, four of them showing different binding affinities for BRD9, and two non-binders. Following the procedure tuned with the training set, the molecules were docked against a reference BRD9 crystal structure, and the binding poses were evaluated not only based on the docking score but also considering the interactions made by the compounds with the protein. Interestingly, compound **10** presented a particular outcome in which the top-ranked pose was not the one with the best protein/ligand interactions. To investigate the consequences of a wrong binding pose choice, both were simulated and analyzed to determine their differences. Not surprisingly, the complex containing the pose with the highest number of protein/ligand contacts showed better results if compared to the other one, confirming the initial hypothesis that the starting point is crucial for the correct outcomes of the MDs. Moreover, we performed a retrospective study on the crystal structure of BRD9/**10**, comparing the experimental binding mode with that was identified as the best one obtained by molecular docking experiments. Interestingly, the two molecules are oriented in a very similar way (Figure 11B), confirming that **10b** was indeed the best outcome of the calculation. In this respect, further consideration should be made on the binding pose selection process, even for relatively short MD simulations and small protein domains like BRD9: whether multiple poses are returned (i.e., a significant variation in the molecule arrangement inside the binding pocket), all those showing promising predicted binding with the protein counterpart should be taken into account in the following MD experiments.

## 4. Materials and Methods

### 4.1. Structure Selection and Preparation

At the time of the study, 46 crystal structures of BRD9 were available in the Protein Data Bank with 16 *apo* structures or BRD9/histone complexes. Out of the 30 BRD9-ligand structures, one representative crystal for each compound series was chosen. The eight chosen ligand-BRD9 co-crystal structures and the *apo* form of BRD9 (Table 9) were downloaded from the Protein Data Bank and processed with the Protein Preparation Wizard tool available in the Schrödinger Suite [32]. This tool resolves common mistakes in the imported structure, like the bond order and the protonation state, and optimizes the H-bond network at physiological pH. In this way, both protein and co-crystallized ligands are parametrized.

### 4.2. Redocking, Molecular Docking Experiments, and Ensemble Docking

For the redocking experiments, the required molecular docking grids were generated using the centroid of each ligand as the center of the box, and all the molecular docking experiments were carried out using the software Glide [28,29,30,31] in Extra Precision mode (XP). In the initial step, 10,000 poses were generated for each ligand, and the best 800 were kept for minimization. Eventually, only the 10 top-ranked poses were saved. For the molecular docking of the test set compounds, among the initial 8 structures of the training set, 5F1H [15] was chosen as the macromolecule counterpart because it featured an optimal resolution (1.82 Å), making this system suitable for molecular docking experiments. Also, the original ligand/protein complex [15] contained a well-characterized and potent inhibitor (BI-9564), featuring a molecular shape and volume intermediate between all the investigated compounds, thus making the related protein conformation suitable for binding the different investigated ligands. The tests were carried out with the same procedure illustrated above. In ensemble docking, the frames of one of the *apo*-BRD9 MD simulations were clustered into 50 groups according to their RMSD difference from frame 0 (see Section 4.4) and the representative structure of each cluster was used as the receptor for the molecular docking. The necessary grids were built using Glide [28,29,30,31] and the centroid of the volume between key residues in the binding site (i.e., Phe44, Tyr99, Asn100, Arg101, and Tyr106) was considered as the center of the box. The eight compounds were docked in XP mode following the parameters illustrated above.

### 4.3. Systems Setup

After the preparation phase, the resulting structures were prepared for the MD simulation. Using the System Setup tool of the software Desmond [33,34], they were inserted in a cubic box with a buffer distance of 10 Å on the three-axis and solvated with TIP3P water molecules. The systems were neutralized, adding an appropriate number of Na^+^ or Cl^−^ ions, and 0.15 M of NaCl was added to mimic the physiological conditions.

### 4.4. Molecular Dynamics

All the simulations of both *apo*-BRD9 and ligand-containing structures were carried out using the software Desmond [33,34] with the OPLS-2005 force field. Initially, each system was relaxed with an internal protocol: the first step is a Brownian Dynamics in NPT ensemble (i.e., keeping the number of molecules, the pressure, and the temperature of the system constant) at 10 K for 100 ns, followed by two 12 ns steps at 10 K, in NVT and NPT ensemble respectively, with restrains on heavy solute atoms. Then, two phases of 12 ns and 24 ns, respectively, were carried out in an NPT ensemble at 300 K with and without restrains. After the relaxation phase, the systems were simulated independently for 100 ns three times in an NPT ensemble at 310 K. For 4UIW, starting from the final frame of the MD1, an additional 900 ns of MD (total simulation time: 1µs) were carried out to study whether longer times could influence results. Eventually, to simplify some of the following analysis, each trajectory was clustered into 10 groups based on the RMSD from the initial frame. One representative structure was selected for each cluster. For the test set compounds, only one MD was carried out for each complex.

### 4.5. Per-Residue Interaction Analysis

To determine the energetic contribution of each residue in the ligand binding, a score-in-place molecular docking was performed with Glide [28,29,30,31] as before (see Section 4.2) on each representative structure taken from the MDs using the same parameters of the canonical docking score (see above for details). During molecular docking, the per-residue interaction energy was computed and, in the training set, the mean value of the three replicas was reported.

### 4.6. MM-GBSA

The free energy was calculated on the representative structure extracted from each MD cluster with the MM-GBSA method using the software Prime [25,26,27] in the VSGB solvent model. The residues within 6 Å from the ligand were left free to move to reduce steric clashes.

## 5. Conclusions

In this work, we reported an in silico molecular dynamics-driven exhaustive analysis method to guide the estimation of the binding affinity of a set of molecules against BRD9 protein. As highlighted from the training set, the docking score alone may misguide the selection of promising compounds and, therefore, has to be combined with other parameters. For this purpose, it is useful to perform molecular dynamics (MD) simulations and calculate the energetic and structural parameters of each complex. This is a fast way to roughly discriminate good binders from bad ones as they immediately stand out; the determination of the mediocre binders is instead challenging and not always univocally possible. The advantage of the proposed method is that it is relatively fast: generally, a single MD of a system containing 44,000 atoms on a machine equipped with six Gold Intel^®^ Xeon^®^ 6230 CPUs can take about 48 h, and the whole data extrapolation can last no more than few days, and it can help exclude compounds with poor performance for the selected target.

## Figures and Tables

**Table 1:** Experimental K_D_ [11,15,16,18,20,21,22] and binding free energies calculated with MM-GBSA method. The values reported for each MD are the mean of the 10 representative structures.

System (Ligand)	Experimental Data		Calculated Data				
	K_D_ (µM)	Experimental Binding Affinity (kcal/mol)	Calculated Binding Affinity (kcal/mol)	MM-GBSA (kcal/mol)			
				MD1	MD2	MD3	Av.
**4UIW** (**1**)	0.002	−11.9	−9.0	−81.7	−85.3	−96.4	−87.8
**5F1H** (**5**)	0.014	−10.7	−9.2	−75.7	−75.7	−72.4	−74.7
**5IGM** (**6**)	0.042	−10.1	−6.7	−71.1	−71.1	−71.1	−71.1
**5E9V** (**4**)	0.070	−9.8	−4.8	−51.5	−47.7	−52.2	−50.4
**6V14** (**8**)	0.082	−9.7	−8.3	−69.1	−64.4	−64.0	−65.9
**4XY8** (**2**)	0.397	−8.7	−9.3	−43.5	−51.3	−39.3	−44.7
**4Z6I** (**3**)	0.493	−8.6	−7.9	−54.2	−78.5	−76.9	−69.9
**6V0S** (**7**)	16	−6.5	−4.5	−25.6	−45.6	−43.4	−38.2

**Table 2:** Mean RMSD value of each system during the three simulations and the total average RMSD value. Average values lower than the *apo* form of the protein are highlighted in green.

RMSD									
	Apo	4UIW	4XY8	4Z6I	5E9V	5F1H	5IGM	6V0S	6V14
**MD1**	2.53	2.07	4.25	2.75	3.12	1.94	2.77	1.83	2.41
**MD2**	3.45	2.56	3.73	2.63	2.67	1.82	2.41	3.02	1.87
**MD3**	2.40	1.89	4.17	3.44	3.16	2.87	2.48	3.39	2.12
**Av.**	2.79	2.17	4.05	2.94	2.98	2.21	2.55	2.74	2.13

**Table 3:** The number of frames of the *apo* MDs that had an RMSD < 2.5 Å from the ligand/protein BRD9 structure was indicated with the related PDB code. The percentage is calculated considering both the 192 filtered frames (see text for details) and the total frames tested.

System	Frames of the *Apo* Form that Resemble the Crystal Structure		
	N° Apo Frames	Percentage on 192 Frames	Percentage on 3000 Frames
**4UIW**	3	2%	0.001%
**4XY8**	8	4%	0.003%
**4Z6I**	7	4%	0.002%
**5E9V**	26	14%	0.009%
**5F1H**	20	10%	0.007%
**5IGM**	15	8%	0.005%
**6V0S**	53	28%	0.018%
**6V14**	8	4%	0.003%

**Table 4:** Protein residues with a mean RMSF value above 4 Å. Key binding site residues are in bold.

System	Amino Acids with Mean RMSF above 4 Å
**Apo**	Phe47, Thr50, Asp51, Ala52, **Ile53**, Ala54, Pro55, Met59, Ile60, Lys62
**4UIW**	/
**4XY8**	Asp51, Ala52, **Ile53**
**4Z6I**	Ala52, **Ile53**, Pro55, Lys62
**5E9V**	Met59
**5F1H**	/
**5IGM**	Ala52
**6V0S**	Ala52
**6V14**	/

**Table 5:** Statistical analysis of the Rg values. The complex showing the lowest range and standard deviation value is highlighted.

Rg Statistics									
	Apo	4UIW	4XY8	4Z6I	5E9V	5F1H	5IGM	6V0S	6V14
Minimum	13.7	13.8	14.9	14.6	14.6	14.5	14.5	14.5	13.8
Maximum	14.4	14.5	16.0	15.2	15.9	15.0	15.1	15.1	14.5
Range	0.72	0.70	1.15	0.66	1.27	0.47	0.63	0.58	0.72
Mean	14.0	14.1	15.4	14.9	15.1	14.8	14.8	14.7	14.1
Std. Dev.	0.105	0.116	0.203	0.130	0.196	0.074	0.125	0.092	0.122

**Table 6:** Analysis of the seven parameters considered and the subsequent binding prediction. The eight ligand/protein complexes are ranked based on the experimental K_D_. Good values are in green, bad values are in red, and average values are in yellow/orange.

System (Ligand)	Molecular Docking Parameters		Molecular Dynamics Parameters							Prediction
	Docking Score	Interactions ^a^	MM-GBSA	Per-Residue Binding Site ^b^	Per-Residue Total^c^	Interactions ^d^	RMSD ^e^	RMSF ^f^	Rg ^g^	
**4UIW (1)**	−7.6	8	−87.80	−29.13	−62.96	5	2.17	0	0.7	Excellent
**5F1H (5)**	−9.2	7	−74.73	−15.20	−56.43	3	2.21	0	0.47	Good
**5IGM (6)**	−6.7	7	−71.09	−20.48	−20.48	3	2.55	0	0.63	Good
**5E9V (4)**	−4.8	7	−50.44	1.61	−32.19	0	2.98	0	1.27	Intermediate
**6V14 (9)**	−8.3	7	−65.86	−20.57	−20.57	2	2.13	0	0.72	Intermediate
**4XY8 (2)**	−9.3	7	−44.67	0.00	0.00	1	4.05	1	1.15	Bad
**4Z6I (3)**	−7.9	8	−69.85	−13.99	−13.99	2	2.94	2	0.66	Intermediate
**6V0S (7)**	−4.5	7	−38.17	5.55	−12.45	1	2.74	0	0.58	Bad

^a^ Number of interactions made with binding site residues. ^b^ Calculated considering only the energetic contribution of residues with values below −5.0 kcal/mol. ^c^ Calculated considering only the energetic contribution of binding site residues with values below −5.0 kcal/mol. ^d^ Number of binding site residues with an interaction time fraction above 0.5. ^e^ Calculated according to the average RMSD value of the *apo* form (2.79 Å). ^f^ Number of residues belonging to the binding site with a fluctuation above 4.0 Å. ^g^ Width of the distribution curve (*apo* form width: 0.72 Å).

**Table 7:** Calculated docking score and MM-GBSA ΔG_bind_ for the 6 tested compounds (**9**–**14**).

Complex	Experimental Data		Calculated Data	
	KD (µM)	Experimental Binding Affinity (kcal/mol)	Calculated Binding Affinity (kcal/mol)	MM-GBSA ΔG_bind_
**9**	0.0094	−10.9	−9.4	−78.88
**10a**	0.099	−9.6	−5.3	−52.01
**10b**			−5.2	−70.70
**11**	1.01	−8.2	−7.4	−70.41
**12**	17.0	−6.5	−5.9	−76.08
**13**	/	/	−5.7	−58.69
**14**	/	/	−7.9	−64.27

**Table 8:** Analysis of the seven parameters considered and the subsequent binding prediction. The six tested complexes related to the test set compounds **9**–**14** are ranked based on the experimental K_D_. Good values are in green, bad values are in red, and average values are in yellow/orange.

System (Ligand)	Molecular Docking Parameters		Molecular Dynamics Parameters							Prediction
	Docking Score	Interactions ^a^	MM-GBSA	Per-Residue Binding Site ^b^	Per-Residue Total ^c^	Interactions ^d^	RMSD ^e^	RMSF ^f^	Rg ^g^	
**9**	−9.4	8	−78.88	−17.67	−61.60	4	2.97	0	0.69	Excellent
**10a**	−5.3	7	−52.01	−5.88	−13.00	0	3.25	0	0.90	Bad
**10b**	−3.8	6	−70.70	−17.36	−17.36	3	2.19	0	0.75	Good
**11**	−7.4	7	−70.41	−13.66	−13.66	3	3.35	0	1.00	Intermediate
**12**	−5.9	9	−76.08	−15.37	−29.07	4	3.23	1	0.86	Good
**13**	−5.7	8	−58.69	−12.03	−17.96	2	3.32	0	0.94	Bad
**14**	−7.9	8	−64.27	−18.56	−18.56	3	3.15	2	1.01	Bad

^a^ Number of interactions made with binding site residues. ^b^ Calculated considering only the energetic contribution of residues with values below −5.0 kcal/mol. ^c^ Calculated considering only the energetic contribution of binding site residues with values below −5.0 kcal/mol. ^d^ Number of binding site residues with an interaction time fraction above 0.5. ^e^ Calculated according to the average RMSD value of the *apo* form (2.79 Å). ^f^ Number of residues belonging to the binding site with a fluctuation above 4.0 Å. ^g^ Width of the distribution curve (*apo* form width: 0.72 Å).

**Table 9:** Structural information of the ten crystal complexes used. References are reported in square brackets.

PDB	Ligand	Resolution (Å)
**4YY4** [20]	/	1.47
**4UIW** [18]	H1B	1.73
**4XY8** [16]	43U	1.70
**4Z6I** [19]	4L3	1.95
**5E9V** [17]	5LO	1.80
**5F1H** [15]	5U6	1.82
**5IGM** [21]	BMF	1.60
**6V0S** [11]	EAE	2.40
**6V14** [11]	QMG	1.70

## Data Availability

No new data were created or analyzed in this study. Data sharing is not applicable to this article.

## Supplementary Materials

The following are available online: Table S1: PDB coordinates of the 5F1H receptor used for the molecular docking and the selected poses for the test set compound; ensemble docking results, Figures S1–S5: Data related to 1 μs MD simulation of 4UIW system.

## Sample Availability

Samples of the compounds are commercially available.

## References

[1] Hui M., Jian Z., Peiyuan Z., Zhenwei W., Huibin Z. (2018). Research progress of selective small molecule bromodomain-containing protein 9 inhibitors. Future Med. Chem..

[2] Kouzarides T. (2000). Acetylation: A regulatory modification to rival phosphorylation?. EMBO J..

[3] Prabakaran S., Lippens G., Steen H., Gunawardena J. (2012). Post-translational modification: Nature’s escape from genetic imprisonment and the basis for dynamic information encoding. WIREs Sys. Biol. Med..

[4] Drazic A., Myklebust L.M., Ree R., Arnesen T. (2016). The world of protein acetylation. Biochim. Biophys. Acta.

[5] Tamkun J.W., Deuring R., Scott M.P., Kissinger M., Pattatucci A.M., Kaufman T.C., Kennison J.A. (1992). Brahma: A regulator of Drosophila homeotic genes structurally related to the yeast transcriptional activator SNF2SWI2. Cell.

[6] Mohrmann L., Verrijzer C.P. (2005). Composition and functional specificity of SWI2/SNF2 class chromatin remodeling complexes. Biochim. Biophys. Acta.

[7] Corona D.F.V., Tamkun J.W. (2004). Multiple roles for ISWI in transcription, chromosome organization and DNA replication. Biochim. Biophys. Acta.

[8] Marfella C.G.A., Imbalzano A.N. (2007). The Chd family of chromatin remodelers. Mutat. Res..

[9] Bao Y., Shen X. (2007). INO80 subfamily of chromatin remodeling complexes. Mutat. Res..

[10] Clapier C.R., Cairns B.R. (2009). The biology of chromatin remodeling complexes. Annu. Rev. Biochem..

[11] Karim R.M., Chan A., Zhu J.-Y., Schönbrunn E. (2020). Structural basis of inhibitor selectivity in the BRD7/9 subfamily of bromodomains. J. Med. Chem..

[12] Kadoch C., Hargreaves D.C., Hodges C., Elias L., Ho L., Ranish J., Crabtree G.R. (2013). Proteomic and bioinformatic analysis of mammalian SWI/SNF complexes identifies extensive roles in human malignancy. Nat. Genet..

[13] Wang Y., Wang L.F., Zhang L.L., Sun H.B., Zhao J. (2020). Molecular mechanism of inhibitor bindings to bromodomain-containing protein 9 explored based on molecular dynamics simulations and calculations of binding free energies. SAR QSAR Environ. Res..

[14] Su J., Liu X., Zhang S., Yan F., Zhang Q., Chen J. (2019). Insight into selective mechanism of class of I-BRD9 inhibitors toward BRD9 based on molecular dynamics simulations. Chem. Biol. Drug Des..

[15] Martin L.J., Koegl M., Bader G., Cockcroft X.-L., Fedorov O., Fiegen D., Gerstberger T., Hofmann M.H., Hohmann A.F., Kessler D. (2016). Structure-based design of an in vivo active selective BRD9 inhibitor. J. Med. Chem..

[16] Picaud S., Strocchia M., Terracciano S., Lauro G., Mendez J., Daniels D.L., Riccio R., Bifulco G., Bruno I., Filippakopoulos P. (2015). 9H-purine scaffold reveals induced-fit pocket plasticity of the BRD9 bromodomain. J. Med. Chem..

[17] Hay D.A., Rogers C.M., Fedorov O., Tallant C., Martin S., Monteiro O.P., Müller S., Knapp S., Schofield C.J., Brennan P.E. (2015). Design and synthesis of potent and selective inhibitors of BRD7 and BRD9 bromodomains. MedChemComm.

[18] Theodoulou N.H., Bamborough P., Bannister A.J., Becher I., Bit R.A., Che K.H., Chung C.-w., Dittmann A., Drewes G., Drewry D.H. (2016). Discovery of I-BRD9, a selective cell active chemical probe for bromodomain containing protein 9 inhibition. J. Med. Chem..

[19] Clark P.G.K., Vieira L.C.C., Tallant C., Fedorov O., Singleton D.C., Rogers C.M., Monteiro O.P., Bennett J.M., Baronio R., Müller S. (2015). LP99: Discovery and synthesis of the first selective BRD7/9 bromodomain inhibitor. Angew. Chem..

[20] Flynn E.M., Huang O.W., Poy F., Oppikofer M., Bellon S.F., Tang Y., Cochran A.G. (2015). A subset of human bromodomains recognizes butyryllysine and crotonyllysine histone peptide modifications. Structure.

[21] Picaud S., Leonards K., Lambert J.-P., Dovey O., Wells C., Fedorov O., Monteiro O., Fujisawa T., Wang C.-Y., Lingard H. (2016). Promiscuous targeting of bromodomains by bromosporine identifies BET proteins as master regulators of primary transcription response in leukemia. Sci. Adv..

[22] Borea P.A., Varani K., Gessi S., Gilli P., Dalpiaz A. (1998). Receptor binding thermodynamics as a tool for linking drug efficacy and affinity. Farmaco.

[23] Amaro R.E., Baudry J., Chodera J., Demir Ö., McCammon J.A., Miao Y., Smith J.C. (2018). Ensemble docking in drug discovery. Biophys. J..

[24] Wang E., Sun H., Wang J., Wang Z., Liu H., Zhang J.Z.H., Hou T. (2019). End-point binding free energy calculation with MM/PBSA and MM/GBSA: Strategies and applications in drug design. Chem. Rev..

[25] Jacobson M.P., Friesner R.A., Xiang Z., Honig B. (2002). On the role of the crystal environment in determining protein side-chain conformations. J. Mol. Biol..

[26] Jacobson M.P., Pincus D.L., Rapp C.S., Day T.J.F., Honig B., Shaw D.E., Friesner R.A. (2004). A hierarchical approach to all-atom protein loop prediction. Proteins Struct. Funct. Bioinform..

[27] (2020). Schrödinger Release 2020-1. Prime.

[28] (2020). Schrödinger Release 2020-1. Glide.

[29] Friesner R.A., Banks J.L., Murphy R.B., Halgren T.A., Klicic J.J., Mainz D.T., Repasky M.P., Knoll E.H., Shelley M., Perry J.K. (2004). Glide:  a new approach for rapid, accurate docking and scoring. 1. method and assessment of docking accuracy. J. Med. Chem..

[30] Friesner R.A., Murphy R.B., Repasky M.P., Frye L.L., Greenwood J.R., Halgren T.A., Sanschagrin P.C., Mainz D.T. (2006). Extra Precision Glide:  Docking and scoring incorporating a model of hydrophobic enclosure for protein—Ligand complexes. J. Med. Chem..

[31] Halgren T.A., Murphy R.B., Friesner R.A., Beard H.S., Frye L.L., Pollard W.T., Banks J.L. (2004). Glide:  A new approach for rapid, accurate docking and scoring. 2. enrichment factors in database screening. J. Med. Chem..

[32] (2020). Schrödinger Release 2020-1. Protein Preparation Wizard.

[33] (2020). Schrödinger Release 2020-1. Desmond Molecular Dynamics System, D.E. Shaw Research, New York, NY, 2020. Maestro-Desmond Interoperability Tools.

[34] Bowers K.J., Chow E., Xu H., Dror R.O., Eastwood M.P., Gregersen B.A., Klepeis J.L., Kolossvary I., Moraes M.A., Sacerdoti F.D. (2006). Scalable Algorithms for Molecular Dynamics Simulations on Commodity Clusters. SC′06: Proceedings of the 2006 ACM/IEEE conference on Supercomputing, Tampa, FL, USA, 11–17 November 2006.

