# Supplementary material for: Insights into the Ligand Binding to Bromodomain-Containing Protein 9 (BRD9): A Guide to the Selection of Potential Binders by Computational Methods
Source: Molecules. 2021 Nov 27;26(23):7192. doi: 10.3390/molecules26237192 (PMC8659208; doi:10.3390/molecules26237192)
Supplement: Supplementary file 1 [file molecules-26-07192-s001.zip › molecules-1459191-supplementary.pdf]

Insights into the ligand binding to bromodomain-containing protein 9 (BRD9): a guide to the selection of potential binders by computational methods

Simona De Vita [1], Maria Giovanna Chini [2], Giuseppe Bifulco [1] and Gianluigi Lauro [1,*]

[1] Department of Pharmacy, University of Salerno, Via Giovanni Paolo II 132, Fisciano 84084, Italy; sdevita@unisa.it (S.D.V), bifulco@unisa.it (G.B.), glauro@unisa.it (G.L.).
[2] Department of Biosciences and Territory, University of Molise, C.da Fonte Lappone, Pesche 86090, Italy; mariagiovanna.chini@unimol.it
* Correspondence: glauro@unisa.it; Telephone: +39 (0)89 969176, Fax: +39 (0)89 969602.

Test set coordinates

5F1H

```
REMARK   4      COMPLIES WITH FORMAT V. 3.0, 1-DEC-2006
REMARK 888
REMARK 888 WRITTEN BY MAESTRO (A PRODUCT OF SCHRODINGER, LLC)
TITLE     5F1H
MODEL        1
ATOM      1  CH3 ACE A  21       5.429  26.182  18.377  1.00  0.00
C
ATOM      2  C   ACE A  21       6.557  25.567  17.549  1.00  0.00
C
ATOM      3  O   ACE A  21       7.663  25.398  18.056  1.00  0.00
O
ATOM      4 1H   ACE A  21       5.801  26.406  19.402  1.00  0.00
H
ATOM      5 2H   ACE A  21       5.080  27.122  17.893  1.00  0.00
H
ATOM      6 3H   ACE A  21       4.578  25.468  18.437  1.00  0.00
H
ATOM      7  N   SER A  22       6.336  25.203  16.236  1.00 67.75
N
ANISOU    7  N   SER A  22     8468   7332   9943   2328   2914   -857
ATOM      8  CA  SER A  22       7.421  24.614  15.447  1.00 64.54
C
ANISOU    8  CA  SER A  22     8139   7062   9320   2252   2575   -728
ATOM      9  C   SER A  22       8.621  25.561  15.330  1.00 66.69
C
ANISOU    9  C   SER A  22     8679   7233   9428   2243   2471   -692
ATOM     10  O   SER A  22       8.440  26.771  15.199  1.00 68.48
O
ANISOU   10  O   SER A  22     8928   7310   9779   2381   2543   -704
ATOM     11  CB  SER A  22       6.921  24.231  14.057  1.00 67.99
C
ANISOU   11  CB  SER A  22     8277   7607   9947   2395   2301   -626
ATOM     12  OG  SER A  22       7.870  23.447  13.353  1.00 73.90
O
ANISOU   12  OG  SER A  22     9106   8485  10486   2300   2043   -515
ATOM     13  H1  SER A  22       5.427  25.342  15.819  1.00  0.00
H
ATOM     14  HA  SER A  22       7.753  23.706  15.951  1.00  0.00
H
ATOM     15  HB3 SER A  22       6.704  25.135  13.488  1.00  0.00
H
ATOM     16  HB2 SER A  22       5.988  23.676  14.148  1.00  0.00
H
ATOM     17  HG  SER A  22       8.685  23.944  13.253  1.00  0.00
H
ATOM     18  N   THR A  23       9.842  25.000  15.383  1.00 60.01
N
ANISOU   18  N   THR A  23     8010   6442   8349   2080   2308   -661
ATOM     19  CA  THR A  23      11.106  25.740  15.287  1.00 58.27
C
ANISOU   19  CA  THR A  23     7989   6101   8050   2034   2209   -659
```

```
ATOM     20  C   THR A  23      11.629  25.734  13.829  1.00 59.52
C
ANISOU   20  C   THR A  23     8058   6286   8271   2099   2018   -505
ATOM     21  O   THR A  23      11.195  24.881  13.053  1.00 57.25
O
ANISOU   21  O   THR A  23     7612   6148   7993   2138   1895   -412
ATOM     22  CB  THR A  23      12.157  25.128  16.242  1.00 64.59
C
ANISOU   22  CB  THR A  23     9020   6896   8626   1832   2127   -755
ATOM     23  OG1 THR A  23      12.431  23.780  15.856  1.00 62.51
O
ANISOU   23  OG1 THR A  23     8676   6805   8272   1736   1957   -681
ATOM     24  CG2 THR A  23      11.750  25.195  17.706  1.00 64.76
C
ANISOU   24  CG2 THR A  23     9273   6835   8499   1786   2326   -906
ATOM     25  H   THR A  23       9.934  24.001  15.496  1.00  0.00
H
ATOM     26  HA  THR A  23      10.926  26.773  15.586  1.00  0.00
H
ATOM     27  HB  THR A  23      13.079  25.698  16.129  1.00  0.00
H
ATOM     28  HG1 THR A  23      11.692  23.219  16.104  1.00  0.00
H
ATOM     29 HG21 THR A  23      12.531  24.749  18.322  1.00  0.00
H
ATOM     30 HG22 THR A  23      11.609  26.236  17.997  1.00  0.00
H
ATOM     31 HG23 THR A  23      10.818  24.648  17.850  1.00  0.00
H
ATOM     32  N   PRO A  24      12.582  26.627  13.438  1.00 56.50
N
ANISOU   32  N   PRO A  24     7802   5735   7932   2105   2012   -484
ATOM     33  CA  PRO A  24      13.104  26.590  12.059  1.00 55.25
C
ANISOU   33  CA  PRO A  24     7638   5553   7802   2164   1912   -330
ATOM     34  C   PRO A  24      13.771  25.267  11.674  1.00 56.23
C
ANISOU   34  C   PRO A  24     7716   5823   7825   2025   1752   -280
ATOM     35  O   PRO A  24      13.697  24.889  10.506  1.00 54.42
O
ANISOU   35  O   PRO A  24     7466   5639   7574   2107   1668   -146
ATOM     36  CB  PRO A  24      14.110  27.738  12.037  1.00 58.05
C
ANISOU   36  CB  PRO A  24     8151   5648   8257   2141   2028   -361
ATOM     37  CG  PRO A  24      13.674  28.653  13.118  1.00 64.71
C
ANISOU   37  CG  PRO A  24     9051   6388   9149   2162   2164   -504
ATOM     38  CD  PRO A  24      13.200  27.734  14.198  1.00 59.84
C
ANISOU   38  CD  PRO A  24     8396   5944   8397   2060   2126   -608
ATOM     39  HA  PRO A  24      12.295  26.804  11.360  1.00  0.00
H
ATOM     40  HB3 PRO A  24      14.048  28.255  11.079  1.00  0.00
H
ATOM     41  HB2 PRO A  24      15.103  27.353  12.268  1.00  0.00
H
```

```
ATOM     42  HG3 PRO A  24      12.837  29.256  12.765  1.00  0.00
H
ATOM     43  HG2 PRO A  24      14.532  29.220  13.478  1.00  0.00
H
ATOM     44  HD2 PRO A  24      14.060  27.355  14.750  1.00  0.00
H
ATOM     45  HD3 PRO A  24      12.433  28.237  14.787  1.00  0.00
H
ATOM     46  N   ILE A  25      14.413  24.564  12.644  1.00 52.50
N
ANISOU   46  N   ILE A  25     7265   5407   7275   1833   1691   -392
ATOM     47  CA  ILE A  25      15.048  23.262  12.398  1.00 50.77
C
ANISOU   47  CA  ILE A  25     6994   5317   6978   1701   1532   -360
ATOM     48  C   ILE A  25      13.969  22.204  12.149  1.00 52.68
C
ANISOU   48  C   ILE A  25     7099   5779   7136   1746   1472   -292
ATOM     49  O   ILE A  25      14.086  21.447  11.188  1.00 50.48
O
ANISOU   49  O   ILE A  25     6754   5591   6834   1753   1363   -191
ATOM     50  CB  ILE A  25      16.116  22.843  13.470  1.00 54.15
C
ANISOU   50  CB  ILE A  25     7508   5698   7367   1514   1424   -507
ATOM     51  CG1 ILE A  25      16.852  21.520  13.108  1.00 53.62
C
ANISOU   51  CG1 ILE A  25     7371   5738   7264   1396   1250   -469
ATOM     52  CG2 ILE A  25      15.546  22.775  14.888  1.00 55.98
C
ANISOU   52  CG2 ILE A  25     7871   5952   7448   1487   1455   -633
ATOM     53  CD1 ILE A  25      17.771  21.558  11.887  1.00 62.76
C
ANISOU   53  CD1 ILE A  25     8464   6801   8582   1378   1259   -390
ATOM     54  H   ILE A  25      14.469  24.933  13.582  1.00  0.00
H
ATOM     55  HA  ILE A  25      15.590  23.365  11.458  1.00  0.00
H
ATOM     56  HB  ILE A  25      16.873  23.627  13.477  1.00  0.00
H
ATOM     57 HG13 ILE A  25      16.118  20.724  12.980  1.00  0.00
H
ATOM     58 HG12 ILE A  25      17.419  21.176  13.973  1.00  0.00
H
ATOM     59 HG21 ILE A  25      16.333  22.480  15.583  1.00  0.00
H
ATOM     60 HG22 ILE A  25      15.159  23.754  15.172  1.00  0.00
H
ATOM     61 HG23 ILE A  25      14.740  22.042  14.921  1.00  0.00
H
ATOM     62 HD11 ILE A  25      18.223  20.577  11.741  1.00  0.00
H
ATOM     63 HD12 ILE A  25      17.191  21.825  11.003  1.00  0.00
H
ATOM     64 HD13 ILE A  25      18.555  22.299  12.044  1.00  0.00
H
ATOM     65  N   GLN A  26      12.892  22.201  12.971  1.00 49.94
N
ANISOU   65  N   GLN A  26     6711   5487   6779   1782   1573   -359
```

```
ATOM     66  CA  GLN A  26      11.767  21.273  12.831  1.00 49.50
C
ANISOU   66  CA  GLN A  26     6472   5594   6740   1815   1565   -336
ATOM     67  C   GLN A  26      11.095  21.426  11.465  1.00 53.70
C
ANISOU   67  C   GLN A  26     6848   6160   7397   2002   1456   -231
ATOM     68  O   GLN A  26      10.803  20.419  10.822  1.00 52.89
O
ANISOU   68  O   GLN A  26     6616   6190   7290   2001   1315   -185
ATOM     69  CB  GLN A  26      10.748  21.460  13.965  1.00 52.41
C
ANISOU   69  CB  GLN A  26     6828   5934   7150   1823   1792   -449
ATOM     70  CG  GLN A  26      11.167  20.782  15.267  1.00 59.23
C
ANISOU   70  CG  GLN A  26     7910   6790   7807   1647   1868   -540
ATOM     71  CD  GLN A  26      10.341  21.214  16.456  1.00 69.66
C
ANISOU   71  CD  GLN A  26     9336   8009   9123   1659   2171   -656
ATOM     72  OE1 GLN A  26      10.127  22.405  16.700  1.00 64.32
O
ANISOU   72  OE1 GLN A  26     8723   7197   8519   1747   2305   -716
ATOM     73  NE2 GLN A  26       9.904  20.252  17.257  1.00 58.86
N
ANISOU   73  NE2 GLN A  26     8032   6674   7659   1563   2325   -694
ATOM     74  H   GLN A  26      12.835  22.864  13.731  1.00  0.00
H
ATOM     75  HA  GLN A  26      12.160  20.259  12.899  1.00  0.00
H
ATOM     76  HB3 GLN A  26       9.780  21.071  13.650  1.00  0.00
H
ATOM     77  HB2 GLN A  26      10.597  22.525  14.144  1.00  0.00
H
ATOM     78  HG3 GLN A  26      12.219  20.991  15.462  1.00  0.00
H
ATOM     79  HG2 GLN A  26      11.096  19.701  15.151  1.00  0.00
H
ATOM     80 HE22 GLN A  26       9.347  20.484  18.067  1.00  0.00
H
ATOM     81 HE21 GLN A  26      10.128  19.288  17.057  1.00  0.00
H
ATOM     82  N   GLN A  27      10.899  22.683  11.009  1.00 51.06
N
ANISOU   82  N   GLN A  27     6563   5682   7155   2174   1496   -200
ATOM     83  CA  GLN A  27      10.296  23.000   9.711  1.00 51.99
C
ANISOU   83  CA  GLN A  27     6632   5774   7349   2403   1347   -100
ATOM     84  C   GLN A  27      11.158  22.464   8.571  1.00 54.81
C
ANISOU   84  C   GLN A  27     7128   6145   7553   2390   1194     26
ATOM     85  O   GLN A  27      10.638  21.804   7.664  1.00 54.97
O
ANISOU   85  O   GLN A  27     7086   6246   7555   2498    992     82
ATOM     86  CB  GLN A  27      10.069  24.517   9.561  1.00 55.34
C
ANISOU   86  CB  GLN A  27     7159   5996   7872   2590   1444    -87
ATOM     87  CG  GLN A  27       8.858  25.046  10.330  1.00 71.84
C
```

```
ANISOU   87  CG  GLN A  27     9062   8057  10176   2688   1569   -205
ATOM     88  CD  GLN A  27       7.559  24.389   9.923  1.00 91.48
C
ANISOU   88  CD  GLN A  27    11251  10641  12864   2831   1406   -236
ATOM     89  OE1 GLN A  27       7.113  24.638   8.700  1.00 87.92
O
ANISOU   89  OE1 GLN A  27    10578  10288  12538   2728   1507   -347
ATOM     90  NE2 GLN A  27       6.959  23.631  10.689  1.00 83.71
N
ANISOU   90  NE2 GLN A  27    10274   9604  11928   3077   1146   -151
ATOM     91  H   GLN A  27      11.174  23.474  11.574  1.00  0.00
H
ATOM     92  HA  GLN A  27       9.325  22.507   9.661  1.00  0.00
H
ATOM     93  HB3 GLN A  27       9.962  24.764   8.505  1.00  0.00
H
ATOM     94  HB2 GLN A  27      10.963  25.050   9.884  1.00  0.00
H
ATOM     95  HG3 GLN A  27       8.780  26.123  10.185  1.00  0.00
H
ATOM     96  HG2 GLN A  27       9.017  24.902  11.399  1.00  0.00
H
ATOM     97 HE22 GLN A  27       6.092  23.202  10.398  1.00  0.00
H
ATOM     98 HE21 GLN A  27       7.334  23.440  11.607  1.00  0.00
H
ATOM     99  N   LEU A  28      12.481  22.696   8.653  1.00 49.51
N
ANISOU   99  N   LEU A  28     6636   5374   6801   2250   1295     47
ATOM    100  CA  LEU A  28      13.431  22.218   7.658  1.00 48.53
C
ANISOU  100  CA  LEU A  28     6652   5215   6572   2209   1245    150
ATOM    101  C   LEU A  28      13.472  20.691   7.600  1.00 49.22
C
ANISOU  101  C   LEU A  28     6613   5507   6581   2082   1098    144
ATOM    102  O   LEU A  28      13.408  20.129   6.505  1.00 47.87
O
ANISOU  102  O   LEU A  28     6504   5366   6316   2156    975    234
ATOM    103  CB  LEU A  28      14.832  22.791   7.915  1.00 48.78
C
ANISOU  103  CB  LEU A  28     6821   5056   6656   2067   1425    127
ATOM    104  CG  LEU A  28      15.942  22.238   7.020  1.00 54.12
C
ANISOU  104  CG  LEU A  28     7611   5657   7297   1984   1460    203
ATOM    105  CD1 LEU A  28      15.791  22.702   5.582  1.00 56.23
C
ANISOU  105  CD1 LEU A  28     8140   5764   7462   2186   1520    360
ATOM    106  CD2 LEU A  28      17.279  22.583   7.553  1.00 57.38
C
ANISOU  106  CD2 LEU A  28     8019   5903   7880   1793   1604    106
ATOM    107  H   LEU A  28      12.854  23.222   9.430  1.00  0.00
H
ATOM    108  HA  LEU A  28      13.100  22.578   6.684  1.00  0.00
H
ATOM    109  HB3 LEU A  28      15.100  22.633   8.960  1.00  0.00
H
```

```
ATOM    110  HB2 LEU A  28      14.800  23.876   7.820  1.00  0.00
H
ATOM    111  HG  LEU A  28      15.857  21.151   7.025  1.00  0.00
H
ATOM    112 HD11 LEU A  28      16.599  22.287   4.979  1.00  0.00
H
ATOM    113 HD12 LEU A  28      14.833  22.362   5.189  1.00  0.00
H
ATOM    114 HD13 LEU A  28      15.832  23.791   5.545  1.00  0.00
H
ATOM    115 HD21 LEU A  28      18.048  22.177   6.896  1.00  0.00
H
ATOM    116 HD22 LEU A  28      17.381  23.667   7.605  1.00  0.00
H
ATOM    117 HD23 LEU A  28      17.393  22.160   8.551  1.00  0.00
H
ATOM    118  N   LEU A  29      13.560  20.024   8.764  1.00 45.01
N
ANISOU  118  N   LEU A  29     5952   5089   6062   1905   1112     39
ATOM    119  CA  LEU A  29      13.615  18.562   8.803  1.00 43.59
C
ANISOU  119  CA  LEU A  29     5669   5081   5814   1778    995     32
ATOM    120  C   LEU A  29      12.317  17.919   8.312  1.00 47.97
C
ANISOU  120  C   LEU A  29     6053   5775   6398   1887    864     42
ATOM    121  O   LEU A  29      12.383  16.919   7.601  1.00 46.46
O
ANISOU  121  O   LEU A  29     5831   5678   6145   1859    726     84
ATOM    122  CB  LEU A  29      14.052  18.026  10.175  1.00 42.84
C
ANISOU  122  CB  LEU A  29     5564   5026   5689   1586   1038    -75
ATOM    123  CG  LEU A  29      15.414  18.522  10.689  1.00 47.39
C
ANISOU  123  CG  LEU A  29     6270   5450   6285   1472   1068   -132
ATOM    124  CD1 LEU A  29      15.679  18.025  12.072  1.00 47.73
C
ANISOU  124  CD1 LEU A  29     6371   5510   6252   1339   1036   -249
ATOM    125  CD2 LEU A  29      16.558  18.135   9.766  1.00 48.32
C
ANISOU  125  CD2 LEU A  29     6405   5514   6441   1410   1017    -74
ATOM    126  H   LEU A  29      13.589  20.532   9.636  1.00  0.00
H
ATOM    127  HA  LEU A  29      14.390  18.265   8.097  1.00  0.00
H
ATOM    128  HB3 LEU A  29      14.051  16.936  10.152  1.00  0.00
H
ATOM    129  HB2 LEU A  29      13.283  18.257  10.913  1.00  0.00
H
ATOM    130  HG  LEU A  29      15.376  19.611  10.730  1.00  0.00
H
ATOM    131 HD11 LEU A  29      16.648  18.391  12.411  1.00  0.00
H
ATOM    132 HD12 LEU A  29      14.900  18.386  12.744  1.00  0.00
H
ATOM    133 HD13 LEU A  29      15.682  16.935  12.073  1.00  0.00
H
```

```
ATOM    134 HD21 LEU A  29      17.497  18.509  10.174  1.00  0.00
H
ATOM    135 HD22 LEU A  29      16.606  17.049   9.681  1.00  0.00
H
ATOM    136 HD23 LEU A  29      16.393  18.569   8.780  1.00  0.00
H
ATOM    137  N   GLU A  30      11.151  18.525   8.625  1.00 46.84
N
ANISOU  137  N   GLU A  30     5784   5620   6392   2019    900    -11
ATOM    138  CA  GLU A  30       9.846  18.051   8.145  1.00 48.14
C
ANISOU  138  CA  GLU A  30     5718   5870   6701   2146    752    -45
ATOM    139  C   GLU A  30       9.784  18.117   6.611  1.00 53.26
C
ANISOU  139  C   GLU A  30     6475   6484   7279   2341    497     52
ATOM    140  O   GLU A  30       9.268  17.193   5.981  1.00 53.19
O
ANISOU  140  O   GLU A  30     6345   6567   7295   2379    281     35
ATOM    141  CB  GLU A  30       8.696  18.854   8.773  1.00 51.38
C
ANISOU  141  CB  GLU A  30     5954   6218   7349   2264    869   -142
ATOM    142  CG  GLU A  30       8.148  18.208  10.033  0.50 59.86
C
ANISOU  142  CG  GLU A  30     6856   7350   8539   2103   1088   -264
ATOM    143  CD  GLU A  30       7.169  19.026  10.853  0.50 80.81
C
ANISOU  143  CD  GLU A  30     9378   9898  11426   2180   1322   -375
ATOM    144  OE1 GLU A  30       6.512  19.933  10.290  0.50 69.60
O
ANISOU  144  OE1 GLU A  30     7861   8391  10194   2402   1232   -382
ATOM    145  OE2 GLU A  30       7.039  18.736  12.064  0.50 76.49
O1-
ANISOU  145  OE2 GLU A  30     8855   9335  10872   2028   1604   -458
ATOM    146  H   GLU A  30      11.150  19.344   9.215  1.00  0.00
H
ATOM    147  HA  GLU A  30       9.734  17.009   8.444  1.00  0.00
H
ATOM    148  HB3 GLU A  30       7.893  18.966   8.045  1.00  0.00
H
ATOM    149  HB2 GLU A  30       9.042  19.862   9.004  1.00  0.00
H
ATOM    150  HG3 GLU A  30       8.978  17.900  10.668  1.00  0.00
H
ATOM    151  HG2 GLU A  30       7.690  17.253   9.776  1.00  0.00
H
ATOM    152  N   HIS A  31      10.346  19.195   6.023  1.00 50.55
N
ANISOU  152  N   HIS A  31     6401   5976   6829   2462    534    148
ATOM    153  CA  HIS A  31      10.438  19.413   4.579  1.00 51.58
C
ANISOU  153  CA  HIS A  31     6791   6003   6805   2664    353    264
ATOM    154  C   HIS A  31      11.301  18.308   3.945  1.00 52.39
C
ANISOU  154  C   HIS A  31     7025   6164   6715   2533    308    324
ATOM    155  O   HIS A  31      10.867  17.688   2.973  1.00 53.38
O
ANISOU  155  O   HIS A  31     7214   6321   6748   2656     57    347
```

```
ATOM    156  CB  HIS A  31      11.004  20.814   4.282  1.00 53.48
C
ANISOU  156  CB  HIS A  31     7335   6007   6976   2780    520    357
ATOM    157  CG  HIS A  31      11.571  20.966   2.907  1.00 58.07
C
ANISOU  157  CG  HIS A  31     8322   6425   7318   2924    469    502
ATOM    158  ND1 HIS A  31      10.758  21.211   1.817  1.00 62.72
N
ANISOU  158  ND1 HIS A  31     9094   6929   7806   3230    182    557
ATOM    159  CD2 HIS A  31      12.857  20.897   2.489  1.00 59.18
C
ANISOU  159  CD2 HIS A  31     8736   6438   7313   2806    687    590
ATOM    160  CE1 HIS A  31      11.569  21.276   0.774  1.00 62.93
C
ANISOU  160  CE1 HIS A  31     9571   6779   7560   3292    255    694
ATOM    161  NE2 HIS A  31      12.842  21.096   1.130  1.00 61.18
N
ANISOU  161  NE2 HIS A  31     9397   6520   7330   3032    595    717
ATOM    162  H   HIS A  31      10.745  19.929   6.590  1.00  0.00
H
ATOM    163  HA  HIS A  31       9.435  19.351   4.157  1.00  0.00
H
ATOM    164  HB3 HIS A  31      11.770  21.057   5.018  1.00  0.00
H
ATOM    165  HB2 HIS A  31      10.223  21.559   4.435  1.00  0.00
H
ATOM    166  HD1 HIS A  31       9.760  21.299   1.941  1.00  0.00
H
ATOM    167  HD2 HIS A  31      13.647  20.714   3.202  1.00  0.00
H
ATOM    168  HE1 HIS A  31      11.167  21.456  -0.212  1.00  0.00
H
ATOM    169  N   PHE A  32      12.509  18.055   4.502  1.00 45.81
N
ANISOU  169  N   PHE A  32     6228   5329   5848   2295    527    328
ATOM    170  CA  PHE A  32      13.409  17.000   4.016  1.00 43.86
C
ANISOU  170  CA  PHE A  32     6067   5119   5479   2154    525    367
ATOM    171  C   PHE A  32      12.742  15.626   4.089  1.00 46.11
C
ANISOU  171  C   PHE A  32     6127   5613   5780   2090    321    304
ATOM    172  O   PHE A  32      12.827  14.861   3.133  1.00 44.92
O
ANISOU  172  O   PHE A  32     6086   5482   5501   2124    184    345
ATOM    173  CB  PHE A  32      14.739  16.974   4.803  1.00 43.66
C
ANISOU  173  CB  PHE A  32     6035   5037   5515   1920    749    338
ATOM    174  CG  PHE A  32      15.744  18.053   4.467  1.00 45.91
C
ANISOU  174  CG  PHE A  32     6547   5069   5828   1933    983    390
ATOM    175  CD1 PHE A  32      15.843  18.561   3.173  1.00 50.09
C
ANISOU  175  CD1 PHE A  32     7394   5416   6223   2101   1049    511
ATOM    176  CD2 PHE A  32      16.633  18.520   5.429  1.00 47.57
C
ANISOU  176  CD2 PHE A  32     6684   5188   6203   1775   1142    307
```

```
ATOM    177  CE1 PHE A  32      16.796  19.531   2.855  1.00 52.42
C
ANISOU  177  CE1 PHE A  32     7911   5430   6574   2094   1344    559
ATOM    178  CE2 PHE A  32      17.585  19.492   5.111  1.00 52.02
C
ANISOU  178  CE2 PHE A  32     7410   5483   6871   1765   1383    328
ATOM    179  CZ  PHE A  32      17.658  19.993   3.827  1.00 51.80
C
ANISOU  179  CZ  PHE A  32     7680   5266   6735   1915   1519    459
ATOM    180  H   PHE A  32      12.827  18.603   5.288  1.00  0.00
H
ATOM    181  HA  PHE A  32      13.639  17.208   2.971  1.00  0.00
H
ATOM    182  HB3 PHE A  32      15.208  15.997   4.684  1.00  0.00
H
ATOM    183  HB2 PHE A  32      14.525  17.003   5.871  1.00  0.00
H
ATOM    184  HD1 PHE A  32      15.177  18.205   2.401  1.00  0.00
H
ATOM    185  HD2 PHE A  32      16.593  18.131   6.436  1.00  0.00
H
ATOM    186  HE1 PHE A  32      16.857  19.918   1.849  1.00  0.00
H
ATOM    187  HE2 PHE A  32      18.264  19.852   5.870  1.00  0.00
H
ATOM    188  HZ  PHE A  32      18.391  20.748   3.584  1.00  0.00
H
ATOM    189  N   LEU A  33      12.031  15.349   5.195  1.00 43.50
N
ANISOU  189  N   LEU A  33     5510   5408   5608   2005    330    199
ATOM    190  CA  LEU A  33      11.349  14.076   5.434  1.00 43.41
C
ANISOU  190  CA  LEU A  33     5258   5562   5673   1919    209    122
ATOM    191  C   LEU A  33      10.193  13.820   4.482  1.00 49.53
C
ANISOU  191  C   LEU A  33     5932   6366   6521   2114    -75     88
ATOM    192  O   LEU A  33      10.073  12.705   3.983  1.00 49.13
O
ANISOU  192  O   LEU A  33     5814   6402   6451   2067   -234     61
ATOM    193  CB  LEU A  33      10.896  13.954   6.900  1.00 43.57
C
ANISOU  193  CB  LEU A  33     5070   5641   5845   1788    380     21
ATOM    194  CG  LEU A  33      10.352  12.588   7.330  1.00 48.06
C
ANISOU  194  CG  LEU A  33     5438   6333   6488   1639    372    -50
ATOM    195  CD1 LEU A  33      11.466  11.577   7.502  1.00 45.84
C
ANISOU  195  CD1 LEU A  33     5287   6089   6041   1444    409     -7
ATOM    196  CD2 LEU A  33       9.564  12.705   8.591  1.00 51.67
C
ANISOU  196  CD2 LEU A  33     5725   6785   7122   1586    579   -153
ATOM    197  H   LEU A  33      11.943  16.041   5.925  1.00  0.00
H
ATOM    198  HA  LEU A  33      12.081  13.287   5.262  1.00  0.00
H
ATOM    199  HB3 LEU A  33      10.150  14.720   7.110  1.00  0.00
H
```

```
ATOM    200  HB2 LEU A  33      11.720  14.235   7.555  1.00  0.00
H
ATOM    201  HG  LEU A  33       9.685  12.227   6.547  1.00  0.00
H
ATOM    202 HD11 LEU A  33      11.044  10.619   7.807  1.00  0.00
H
ATOM    203 HD12 LEU A  33      11.996  11.455   6.557  1.00  0.00
H
ATOM    204 HD13 LEU A  33      12.161  11.927   8.265  1.00  0.00
H
ATOM    205 HD21 LEU A  33       9.188  11.723   8.877  1.00  0.00
H
ATOM    206 HD22 LEU A  33      10.202  13.094   9.384  1.00  0.00
H
ATOM    207 HD23 LEU A  33       8.725  13.383   8.434  1.00  0.00
H
ATOM    208  N   ARG A  34       9.350  14.847   4.222  1.00 48.64
N
ANISOU  208  N   ARG A  34     5807   6163   6511   2344   -171      74
ATOM    209  CA  ARG A  34       8.214  14.744   3.302  1.00 51.09
C
ANISOU  209  CA  ARG A  34     6022   6457   6932   2582   -528      13
ATOM    210  C   ARG A  34       8.677  14.379   1.898  1.00 56.22
C
ANISOU  210  C   ARG A  34     7015   7056   7289   2698   -768     102
ATOM    211  O   ARG A  34       8.040  13.548   1.247  1.00 57.72
O
ANISOU  211  O   ARG A  34     7108   7299   7526   2766  -1080      22
ATOM    212  CB  ARG A  34       7.425  16.062   3.252  1.00 52.66
C
ANISOU  212  CB  ARG A  34     6208   6524   7277   2834   -593      -3
ATOM    213  CG  ARG A  34       6.287  16.144   4.253  1.00 60.62
C
ANISOU  213  CG  ARG A  34     6758   7567   8709   2836   -553    -173
ATOM    214  CD  ARG A  34       5.335  17.301   3.944  1.00 69.11
C
ANISOU  214  CD  ARG A  34     7782   8493   9983   3150   -748    -214
ATOM    215  NE  ARG A  34       6.045  18.530   3.566  1.00 72.91
N
ANISOU  215  NE  ARG A  34     8672   8828  10204   3274   -645     -58
ATOM    216  CZ  ARG A  34       6.510  19.428   4.429  1.00 85.28
C
ANISOU  216  CZ  ARG A  34    10275  10338  11789   3189   -292     -32
ATOM    217  NH1 ARG A  34       6.330  19.262   5.734  1.00 74.70
N
ANISOU  217  NH1 ARG A  34     8641   9073  10668   2995    -12    -144
ATOM    218  NH2 ARG A  34       7.153  20.503   3.993  1.00 69.44
N1+
ANISOU  218  NH2 ARG A  34     8636   8171   9576   3298   -202      99
ATOM    219  H   ARG A  34       9.489  15.741   4.672  1.00  0.00
H
ATOM    220  HA  ARG A  34       7.550  13.958   3.661  1.00  0.00
H
ATOM    221  HB3 ARG A  34       7.033  16.210   2.246  1.00  0.00
H
ATOM    222  HB2 ARG A  34       8.107  16.897   3.411  1.00  0.00
H
```

```
ATOM    223  HG3 ARG A  34       6.695  16.268   5.256  1.00  0.00
H
ATOM    224  HG2 ARG A  34       5.731  15.206   4.248  1.00  0.00
H
ATOM    225  HD3 ARG A  34       4.709  17.497   4.814  1.00  0.00
H
ATOM    226  HD2 ARG A  34       4.661  17.010   3.139  1.00  0.00
H
ATOM    227  HE  ARG A  34       6.171  18.671   2.574  1.00  0.00
H
ATOM    228 HH12 ARG A  34       6.686  19.948   6.384  1.00  0.00
H
ATOM    229 HH11 ARG A  34       5.837  18.450   6.077  1.00  0.00
H
ATOM    230 HH22 ARG A  34       7.505  21.183   4.652  1.00  0.00
H
ATOM    231 HH21 ARG A  34       7.291  20.642   3.002  1.00  0.00
H
ATOM    232  N   GLN A  35       9.780  15.008   1.437  1.00 52.13
N
ANISOU  232  N   GLN A  35     6912   6407   6489   2714   -598    252
ATOM    233  CA  GLN A  35      10.376  14.778   0.119  1.00 53.40
C
ANISOU  233  CA  GLN A  35     7505   6459   6324   2817   -705    356
ATOM    234  C   GLN A  35      10.900  13.343   0.005  1.00 55.07
C
ANISOU  234  C   GLN A  35     7653   6799   6474   2607   -705    328
ATOM    235  O   GLN A  35      10.761  12.724  -1.052  1.00 56.19
O
ANISOU  235  O   GLN A  35     8000   6915   6435   2713   -948    328
ATOM    236  CB  GLN A  35      11.516  15.774  -0.139  1.00 54.81
C
ANISOU  236  CB  GLN A  35     8090   6431   6306   2822   -379    506
ATOM    237  CG  GLN A  35      11.065  17.156  -0.576  1.00 80.01
C
ANISOU  237  CG  GLN A  35    11530   9424   9446   3100   -423    572
ATOM    238  CD  GLN A  35      12.255  18.023  -0.897  1.00108.67
C
ANISOU  238  CD  GLN A  35    15552  12819  12921   3065    -31    711
ATOM    239  OE1 GLN A  35      12.537  18.198  -2.181  1.00103.96
O
ANISOU  239  OE1 GLN A  35    14802  12201  12496   2898    273    698
ATOM    240  NE2 GLN A  35      12.944  18.532  -0.006  1.00106.66
N
ANISOU  240  NE2 GLN A  35    15823  12357  12347   3205     -5    830
ATOM    241  H   GLN A  35      10.251  15.690   2.015  1.00  0.00
H
ATOM    242  HA  GLN A  35       9.607  14.927  -0.639  1.00  0.00
H
ATOM    243  HB3 GLN A  35      12.193  15.362  -0.888  1.00  0.00
H
ATOM    244  HB2 GLN A  35      12.129  15.861   0.758  1.00  0.00
H
ATOM    245  HG3 GLN A  35      10.484  17.618   0.223  1.00  0.00
H
ATOM    246  HG2 GLN A  35      10.431  17.069  -1.458  1.00  0.00
H
```

```
ATOM    247 HE22 GLN A  35      13.737  19.109  -0.248  1.00  0.00
H
ATOM    248 HE21 GLN A  35      12.714  18.371   0.964  1.00  0.00
H
ATOM    249  N   LEU A  36      11.483  12.814   1.099  1.00 48.17
N
ANISOU  249  N   LEU A  36     6521   6044   5736   2327   -456    295
ATOM    250  CA  LEU A  36      12.005  11.444   1.148  1.00 45.82
C
ANISOU  250  CA  LEU A  36     6135   5859   5414   2116   -434    268
ATOM    251  C   LEU A  36      10.881  10.421   1.163  1.00 50.74
C
ANISOU  251  C   LEU A  36     6454   6627   6198   2114   -706    137
ATOM    252  O   LEU A  36      10.969   9.417   0.452  1.00 50.62
O
ANISOU  252  O   LEU A  36     6509   6643   6080   2090   -857    119
ATOM    253  CB  LEU A  36      12.918  11.249   2.370  1.00 43.28
C
ANISOU  253  CB  LEU A  36     5671   5584   5188   1857   -139    267
ATOM    254  CG  LEU A  36      14.227  12.025   2.342  1.00 47.53
C
ANISOU  254  CG  LEU A  36     6450   5958   5650   1807    126    355
ATOM    255  CD1 LEU A  36      14.901  12.016   3.697  1.00 45.91
C
ANISOU  255  CD1 LEU A  36     6073   5783   5590   1601    305    307
ATOM    256  CD2 LEU A  36      15.136  11.531   1.253  1.00 51.12
C
ANISOU  256  CD2 LEU A  36     7172   6312   5939   1792    182    422
ATOM    257  H   LEU A  36      11.577  13.367   1.939  1.00  0.00
H
ATOM    258  HA  LEU A  36      12.601  11.277   0.251  1.00  0.00
H
ATOM    259  HB3 LEU A  36      13.131  10.187   2.494  1.00  0.00
H
ATOM    260  HB2 LEU A  36      12.367  11.504   3.275  1.00  0.00
H
ATOM    261  HG  LEU A  36      13.982  13.062   2.111  1.00  0.00
H
ATOM    262 HD11 LEU A  36      15.833  12.579   3.643  1.00  0.00
H
ATOM    263 HD12 LEU A  36      14.242  12.475   4.434  1.00  0.00
H
ATOM    264 HD13 LEU A  36      15.114  10.988   3.991  1.00  0.00
H
ATOM    265 HD21 LEU A  36      16.061  12.107   1.262  1.00  0.00
H
ATOM    266 HD22 LEU A  36      15.363  10.478   1.418  1.00  0.00
H
ATOM    267 HD23 LEU A  36      14.644  11.649   0.287  1.00  0.00
H
ATOM    268  N   GLN A  37       9.813  10.689   1.956  1.00 48.25
N
ANISOU  268  N   GLN A  37     5795   6371   6167   2135   -743     31
ATOM    269  CA  GLN A  37       8.638   9.820   2.091  1.00 49.14
C
ANISOU  269  CA  GLN A  37     5539   6576   6554   2120   -944   -128
```

```
ATOM    270  C   GLN A  37       7.863   9.657   0.785  1.00 56.19
C
ANISOU  270  C   GLN A  37      6496   7418   7435   2364  -1398   -198
ATOM    271  O   GLN A  37       7.202   8.631   0.608  1.00 57.07
O
ANISOU  271  O   GLN A  37      6362   7591   7732   2322  -1604   -335
ATOM    272  CB  GLN A  37       7.722  10.272   3.242  1.00 51.04
C
ANISOU  272  CB  GLN A  37      5412   6833   7146   2088   -790   -234
ATOM    273  CG  GLN A  37       8.314   9.975   4.622  1.00 56.82
C
ANISOU  273  CG  GLN A  37      6094   7618   7876   1824   -397   -208
ATOM    274  CD  GLN A  37       7.285   9.567   5.650  1.00 70.86
C
ANISOU  274  CD  GLN A  37      7507   9417   9998   1718   -225   -349
ATOM    275  OE1 GLN A  37       7.003  10.302   6.601  1.00 64.03
O
ANISOU  275  OE1 GLN A  37      6578   8502   9250   1713     17   -375
ATOM    276  NE2 GLN A  37       6.754   8.355   5.528  1.00 58.58
N
ANISOU  276  NE2 GLN A  37      5732   7912   8611   1612   -289   -445
ATOM    277  H   GLN A  37       9.792  11.536   2.505  1.00  0.00
H
ATOM    278  HA  GLN A  37       9.010   8.831   2.358  1.00  0.00
H
ATOM    279  HB3 GLN A  37       6.754   9.779   3.149  1.00  0.00
H
ATOM    280  HB2 GLN A  37       7.529  11.341   3.153  1.00  0.00
H
ATOM    281  HG3 GLN A  37       8.855  10.851   4.980  1.00  0.00
H
ATOM    282  HG2 GLN A  37       9.066   9.191   4.532  1.00  0.00
H
ATOM    283 HE22 GLN A  37       6.062   8.038   6.192  1.00  0.00
H
ATOM    284 HE21 GLN A  37       7.043   7.751   4.772  1.00  0.00
H
ATOM    285  N   ARG A  38       7.979  10.643  -0.144  1.00 54.52
N
ANISOU  285  N   ARG A  38      6655   7067   6993   2624  -1562   -110
ATOM    286  CA  ARG A  38       7.349  10.610  -1.472  1.00 57.74
C
ANISOU  286  CA  ARG A  38      7280   7377   7284   2911  -2047   -160
ATOM    287  C   ARG A  38       7.917   9.455  -2.301  1.00 60.41
C
ANISOU  287  C   ARG A  38      7876   7732   7346   2838  -2151   -145
ATOM    288  O   ARG A  38       7.200   8.881  -3.122  1.00 62.50
O
ANISOU  288  O   ARG A  38      8164   7968   7614   2991  -2592   -267
ATOM    289  CB  ARG A  38       7.566  11.931  -2.229  1.00 60.38
C
ANISOU  289  CB  ARG A  38      8090   7514   7336   3197  -2113    -26
ATOM    290  CG  ARG A  38       6.650  13.066  -1.798  1.00 76.59
C
ANISOU  290  CG  ARG A  38      9916   9505   9678   3386  -2209    -83
ATOM    291  CD  ARG A  38       6.556  14.121  -2.883  1.00 93.62
C
```

```
ANISOU  291  CD  ARG A  38    12593  11434  11546   3751  -2459     18
ATOM    292  NE  ARG A  38       5.979  15.373  -2.390  1.00107.71
N
ANISOU  292  NE  ARG A  38    14231  13135  13560   3902  -2415     14
ATOM    293  CZ  ARG A  38       6.692  16.431  -2.015  1.00124.93
C
ANISOU  293  CZ  ARG A  38    16670  15212  15588   3889  -2039    174
ATOM    294  NH1 ARG A  38       6.084  17.528  -1.584  1.00115.87
N
ANISOU  294  NH1 ARG A  38    15373  13981  14670   4035  -2021    153
ATOM    295  NH2 ARG A  38       8.018  16.402  -2.070  1.00110.79
N1+
ANISOU  295  NH2 ARG A  38    15265  13373  13456   3730  -1671    339
ATOM    296  H   ARG A  38       8.527  11.467   0.059  1.00  0.00
H
ATOM    297  HA  ARG A  38       6.278  10.453  -1.345  1.00  0.00
H
ATOM    298  HB3 ARG A  38       7.447  11.758  -3.299  1.00  0.00
H
ATOM    299  HB2 ARG A  38       8.604  12.244  -2.119  1.00  0.00
H
ATOM    300  HG3 ARG A  38       7.037  13.518  -0.885  1.00  0.00
H
ATOM    301  HG2 ARG A  38       5.656  12.671  -1.589  1.00  0.00
H
ATOM    302  HD3 ARG A  38       5.950  13.742  -3.706  1.00  0.00
H
ATOM    303  HD2 ARG A  38       7.549  14.314  -3.288  1.00  0.00
H
ATOM    304  HE  ARG A  38       4.970  15.402  -2.344  1.00  0.00
H
ATOM    305 HH12 ARG A  38       6.629  18.329  -1.300  1.00  0.00
H
ATOM    306 HH11 ARG A  38       5.076  17.562  -1.540  1.00  0.00
H
ATOM    307 HH22 ARG A  38       8.553  17.209  -1.784  1.00  0.00
H
ATOM    308 HH21 ARG A  38       8.492  15.573  -2.398  1.00  0.00
H
ATOM    309  N   LYS A  39       9.198   9.103  -2.062  1.00 53.76
N
ANISOU  309  N   LYS A  39     7211   6918   6299   2610  -1761    -16
ATOM    310  CA  LYS A  39       9.890   8.009  -2.749  1.00 52.75
C
ANISOU  310  CA  LYS A  39     7320   6791   5930   2512  -1763      6
ATOM    311  C   LYS A  39       9.471   6.612  -2.249  1.00 54.70
C
ANISOU  311  C   LYS A  39     7146   7201   6435   2299  -1833   -138
ATOM    312  O   LYS A  39       9.830   5.613  -2.870  1.00 54.60
O
ANISOU  312  O   LYS A  39     7287   7191   6267   2236  -1904   -155
ATOM    313  CB  LYS A  39      11.422   8.194  -2.695  1.00 53.17
C
ANISOU  313  CB  LYS A  39     7686   6766   5749   2367  -1314    176
ATOM    314  CG  LYS A  39      11.945   9.521  -3.282  1.00 67.82
C
ANISOU  314  CG  LYS A  39    10016   8403   7349   2559  -1169    323
```

```
ATOM    315  CD  LYS A  39      11.622   9.745  -4.774  1.00 84.12
C
ANISOU  315  CD  LYS A  39    12628  10280   9053   2868  -1476    351
ATOM    316  CE  LYS A  39      12.506   8.979  -5.733  1.00 95.87
C
ANISOU  316  CE  LYS A  39    14555  11656  10214   2826  -1355    404
ATOM    317  NZ  LYS A  39      12.087   9.193  -7.144  1.00108.27
N1+
ANISOU  317  NZ  LYS A  39    16741  13021  11378   3154  -1687    421
ATOM    318  H   LYS A  39       9.740   9.605  -1.373  1.00  0.00
H
ATOM    319  HA  LYS A  39       9.603   8.066  -3.799  1.00  0.00
H
ATOM    320  HB3 LYS A  39      11.904   7.360  -3.205  1.00  0.00
H
ATOM    321  HB2 LYS A  39      11.760   8.098  -1.663  1.00  0.00
H
ATOM    322  HG3 LYS A  39      13.023   9.581  -3.134  1.00  0.00
H
ATOM    323  HG2 LYS A  39      11.551  10.353  -2.699  1.00  0.00
H
ATOM    324  HD3 LYS A  39      11.682  10.810  -5.000  1.00  0.00
H
ATOM    325  HD2 LYS A  39      10.579   9.487  -4.960  1.00  0.00
H
ATOM    326  HE3 LYS A  39      12.460   7.916  -5.498  1.00  0.00
H
ATOM    327  HE2 LYS A  39      13.540   9.301  -5.609  1.00  0.00
H
ATOM    328  HZ1 LYS A  39      12.147  10.176  -7.368  1.00  0.00
H
ATOM    329  HZ2 LYS A  39      12.694   8.670  -7.759  1.00  0.00
H
ATOM    330  HZ3 LYS A  39      11.135   8.878  -7.264  1.00  0.00
H
ATOM    331  N   ASP A  40       8.687   6.539  -1.159  1.00 49.96
N
ANISOU  331  N   ASP A  40     6045   6708   6229   2191  -1783   -246
ATOM    332  CA  ASP A  40       8.200   5.272  -0.607  1.00 48.81
C
ANISOU  332  CA  ASP A  40     5495   6675   6375   1986  -1786   -386
ATOM    333  C   ASP A  40       6.648   5.280  -0.588  1.00 56.15
C
ANISOU  333  C   ASP A  40     6002   7596   7738   2109  -2097   -600
ATOM    334  O   ASP A  40       6.059   5.430   0.485  1.00 55.65
O
ANISOU  334  O   ASP A  40     5558   7563   8023   2010  -1883   -667
ATOM    335  CB  ASP A  40       8.831   5.022   0.787  1.00 46.50
C
ANISOU  335  CB  ASP A  40     5038   6463   6166   1706  -1333   -318
ATOM    336  CG  ASP A  40       8.299   3.827   1.565  1.00 49.46
C
ANISOU  336  CG  ASP A  40     5040   6914   6837   1490  -1239   -440
ATOM    337  OD1 ASP A  40       8.035   2.770   0.939  1.00 48.33
O
ANISOU  337  OD1 ASP A  40     4839   6784   6739   1457  -1443   -534
```

```
ATOM    338  OD2 ASP A  40       8.125   3.956   2.793  1.00 53.51
O1-
ANISOU  338  OD2 ASP A  40      5355   7447   7528   1357   -946   -445
ATOM    339  H   ASP A  40       8.407   7.383  -0.681  1.00  0.00
H
ATOM    340  HA  ASP A  40       8.524   4.470  -1.271  1.00  0.00
H
ATOM    341  HB3 ASP A  40       8.728   5.921   1.395  1.00  0.00
H
ATOM    342  HB2 ASP A  40       9.912   4.929   0.681  1.00  0.00
H
ATOM    343  N   PRO A  41       5.963   5.158  -1.764  1.00 57.13
N
ANISOU  343  N   PRO A  41      6197   7646   7865   2339  -2606   -727
ATOM    344  CA  PRO A  41       4.487   5.231  -1.763  1.00 60.86
C
ANISOU  344  CA  PRO A  41      6205   8075   8846   2475  -2949   -969
ATOM    345  C   PRO A  41       3.761   4.065  -1.096  1.00 65.32
C
ANISOU  345  C   PRO A  41      6214   8693   9911   2238  -2865  -1172
ATOM    346  O   PRO A  41       2.635   4.249  -0.630  1.00 67.61
O
ANISOU  346  O   PRO A  41      6009   8935  10743   2261  -2902  -1359
ATOM    347  CB  PRO A  41       4.135   5.342  -3.248  1.00 66.31
C
ANISOU  347  CB  PRO A  41      7219   8643   9332   2801  -3571  -1046
ATOM    348  CG  PRO A  41       5.258   4.682  -3.946  1.00 68.71
C
ANISOU  348  CG  PRO A  41      8033   8957   9117   2729  -3511   -912
ATOM    349  CD  PRO A  41       6.483   4.998  -3.139  1.00 59.76
C
ANISOU  349  CD  PRO A  41      7056   7896   7753   2504  -2901   -674
ATOM    350  HA  PRO A  41       4.187   6.154  -1.267  1.00  0.00
H
ATOM    351  HB3 PRO A  41       4.101   6.393  -3.533  1.00  0.00
H
ATOM    352  HB2 PRO A  41       3.215   4.791  -3.443  1.00  0.00
H
ATOM    353  HG3 PRO A  41       5.363   5.106  -4.945  1.00  0.00
H
ATOM    354  HG2 PRO A  41       5.097   3.604  -3.952  1.00  0.00
H
ATOM    355  HD2 PRO A  41       7.164   4.147  -3.170  1.00  0.00
H
ATOM    356  HD3 PRO A  41       6.903   5.945  -3.478  1.00  0.00
H
ATOM    357  N   HIS A  42       4.392   2.878  -1.048  1.00 59.36
N
ANISOU  357  N   HIS A  42      5538   8007   9010   2012  -2720  -1142
ATOM    358  CA  HIS A  42       3.791   1.690  -0.436  1.00 60.08
C
ANISOU  358  CA  HIS A  42      5169   8119   9539   1769  -2589  -1315
ATOM    359  C   HIS A  42       3.967   1.636   1.087  1.00 60.45
C
ANISOU  359  C   HIS A  42      5008   8209   9751   1505  -1980  -1237
ATOM    360  O   HIS A  42       3.400   0.755   1.741  1.00 61.73
O
```

```
ANISOU  360  O   HIS A  42      4806   8346  10303   1302  -1774  -1370
ATOM    361  CB  HIS A  42        4.289   0.399  -1.104  1.00 60.69
C
ANISOU  361  CB  HIS A  42      5437   8222   9402   1660  -2719  -1331
ATOM    362  CG  HIS A  42        4.109   0.373  -2.590  1.00 67.29
C
ANISOU  362  CG  HIS A  42      6552   8985  10030   1922  -3317  -1425
ATOM    363  ND1 HIS A  42        5.037   0.431  -3.573  1.00 73.92
N
ANISOU  363  ND1 HIS A  42      7063   9729  11294   2086  -3840  -1716
ATOM    364  CD2 HIS A  42        2.852   0.272  -3.165  1.00 68.58
C
ANISOU  364  CD2 HIS A  42      7318   9126   9614   2049  -3453  -1278
ATOM    365  CE1 HIS A  42        4.353   0.370  -4.767  1.00 75.37
C
ANISOU  365  CE1 HIS A  42      7712   9844  11083   2326  -4325  -1729
ATOM    366  NE2 HIS A  42        3.054   0.281  -4.473  1.00 72.62
N
ANISOU  366  NE2 HIS A  42      7954   9530  10110   2307  -4074  -1462
ATOM    367  H   HIS A  42        5.316   2.781  -1.443  1.00  0.00
H
ATOM    368  HA  HIS A  42        2.719   1.748  -0.626  1.00  0.00
H
ATOM    369  HB3 HIS A  42        3.778  -0.457  -0.663  1.00  0.00
H
ATOM    370  HB2 HIS A  42        5.342   0.251  -0.865  1.00  0.00
H
ATOM    371  HD1 HIS A  42        6.085   0.509  -3.323  1.00  0.00
H
ATOM    372  HD2 HIS A  42        2.032   0.211  -2.579  1.00  0.00
H
ATOM    373  HE1 HIS A  42        4.859   0.397  -5.641  1.00  0.00
H
ATOM    374  N   GLY A  43        4.731   2.583   1.629  1.00 52.69
N
ANISOU  374  N   GLY A  43      4291   7260   8470   1517  -1697  -1032
ATOM    375  CA  GLY A  43        4.972   2.705   3.060  1.00 50.13
C
ANISOU  375  CA  GLY A  43      3892   6952   8204   1314  -1169   -948
ATOM    376  C   GLY A  43        5.824   1.608   3.655  1.00 50.86
C
ANISOU  376  C   GLY A  43      4129   7093   8105   1054   -874   -850
ATOM    377  O   GLY A  43        5.638   1.251   4.823  1.00 49.84
O
ANISOU  377  O   GLY A  43      3866   6932   8138    868   -488   -860
ATOM    378  H   GLY A  43        5.181   3.268   1.039  1.00  0.00
H
ATOM    379  HA3 GLY A  43        4.017   2.745   3.584  1.00  0.00
H
ATOM    380  HA2 GLY A  43        5.432   3.671   3.267  1.00  0.00
H
ATOM    381  N   PHE A  44        6.785   1.079   2.867  1.00 46.08
N
ANISOU  381  N   PHE A  44      3835   6534   7141   1051  -1034   -751
ATOM    382  CA  PHE A  44        7.705   0.038   3.326  1.00 43.36
C
ANISOU  382  CA  PHE A  44      3644   6218   6611    834   -807   -654
```

```
ATOM    383  C   PHE A  44       8.621   0.551   4.433  1.00 44.33
C
ANISOU  383  C   PHE A  44      3975   6348   6522    745   -464   -490
ATOM    384  O   PHE A  44       9.004  -0.219   5.301  1.00 43.37
O
ANISOU  384  O   PHE A  44      3892   6213   6375    562   -219   -449
ATOM    385  CB  PHE A  44       8.546  -0.514   2.166  1.00 44.11
C
ANISOU  385  CB  PHE A  44      4026   6334   6402    876  -1043   -599
ATOM    386  CG  PHE A  44       7.776  -1.181   1.047  1.00 47.73
C
ANISOU  386  CG  PHE A  44      4364   6770   7001    956  -1422   -773
ATOM    387  CD1 PHE A  44       6.778  -2.112   1.323  1.00 52.25
C
ANISOU  387  CD1 PHE A  44      4552   7314   7985    833  -1434   -960
ATOM    388  CD2 PHE A  44       8.095  -0.931  -0.282  1.00 50.18
C
ANISOU  388  CD2 PHE A  44      4985   7053   7028   1151  -1754   -759
ATOM    389  CE1 PHE A  44       6.086  -2.747   0.288  1.00 55.72
C
ANISOU  389  CE1 PHE A  44      4865   7715   8592    908  -1835  -1156
ATOM    390  CE2 PHE A  44       7.404  -1.573  -1.318  1.00 55.64
C
ANISOU  390  CE2 PHE A  44      5627   7703   7811   1243  -2161   -939
ATOM    391  CZ  PHE A  44       6.407  -2.475  -1.024  1.00 55.56
C
ANISOU  391  CZ  PHE A  44      5181   7681   8248   1120  -2229  -1147
ATOM    392  H   PHE A  44       6.892   1.401   1.916  1.00  0.00
H
ATOM    393  HA  PHE A  44       7.112  -0.781   3.732  1.00  0.00
H
ATOM    394  HB3 PHE A  44       9.286  -1.211   2.558  1.00  0.00
H
ATOM    395  HB2 PHE A  44       9.159   0.287   1.753  1.00  0.00
H
ATOM    396  HD1 PHE A  44       6.531  -2.351   2.347  1.00  0.00
H
ATOM    397  HD2 PHE A  44       8.884  -0.235  -0.526  1.00  0.00
H
ATOM    398  HE1 PHE A  44       5.300  -3.451   0.517  1.00  0.00
H
ATOM    399  HE2 PHE A  44       7.654  -1.360  -2.347  1.00  0.00
H
ATOM    400  HZ  PHE A  44       5.876  -2.970  -1.824  1.00  0.00
H
ATOM    401  N   PHE A  45       8.944   1.854   4.415  1.00 40.47
N
ANISOU  401  N   PHE A  45      3636   5853   5885    885   -469   -407
ATOM    402  CA  PHE A  45       9.822   2.497   5.397  1.00 37.50
C
ANISOU  402  CA  PHE A  45      3459   5462   5328    829   -211   -283
ATOM    403  C   PHE A  45       9.043   3.405   6.362  1.00 43.38
C
ANISOU  403  C   PHE A  45      4065   6166   6250    858    -11   -329
ATOM    404  O   PHE A  45       9.657   4.099   7.169  1.00 41.29
O
ANISOU  404  O   PHE A  45      3979   5873   5836    837    169   -251
```

```
ATOM    405  CB  PHE A  45      10.939   3.274   4.672  1.00 37.42
C
ANISOU  405  CB  PHE A  45     3742   5436   5037    938   -306   -162
ATOM    406  CG  PHE A  45      11.867   2.389   3.880  1.00 37.28
C
ANISOU  406  CG  PHE A  45     3897   5420   4847    888   -397   -111
ATOM    407  CD1 PHE A  45      12.944   1.760   4.494  1.00 37.78
C
ANISOU  407  CD1 PHE A  45     4071   5468   4818    736   -250    -45
ATOM    408  CD2 PHE A  45      11.666   2.180   2.520  1.00 40.03
C
ANISOU  408  CD2 PHE A  45     4323   5760   5126   1009   -642   -142
ATOM    409  CE1 PHE A  45      13.805   0.939   3.761  1.00 38.02
C
ANISOU  409  CE1 PHE A  45     4231   5476   4739    693   -305    -11
ATOM    410  CE2 PHE A  45      12.519   1.344   1.791  1.00 41.72
C
ANISOU  410  CE2 PHE A  45     4724   5951   5177    960   -675   -104
ATOM    411  CZ  PHE A  45      13.580   0.727   2.419  1.00 37.81
C
ANISOU  411  CZ  PHE A  45     4281   5442   4641    796   -485    -39
ATOM    412  H   PHE A  45       8.575   2.456   3.693  1.00  0.00
H
ATOM    413  HA  PHE A  45      10.293   1.711   5.987  1.00  0.00
H
ATOM    414  HB3 PHE A  45      11.516   3.843   5.401  1.00  0.00
H
ATOM    415  HB2 PHE A  45      10.493   4.015   4.009  1.00  0.00
H
ATOM    416  HD1 PHE A  45      13.123   1.903   5.549  1.00  0.00
H
ATOM    417  HD2 PHE A  45      10.844   2.665   2.015  1.00  0.00
H
ATOM    418  HE1 PHE A  45      14.648   0.470   4.247  1.00  0.00
H
ATOM    419  HE2 PHE A  45      12.346   1.183   0.737  1.00  0.00
H
ATOM    420  HZ  PHE A  45      14.235   0.077   1.858  1.00  0.00
H
ATOM    421  N   ALA A  46       7.691   3.378   6.292  1.00 44.10
N
ANISOU  421  N   ALA A  46     3827   6232   6695    904    -41   -479
ATOM    422  CA  ALA A  46       6.808   4.213   7.120  1.00 46.25
C
ANISOU  422  CA  ALA A  46     3918   6437   7217    941    178   -554
ATOM    423  C   ALA A  46       6.800   3.877   8.620  1.00 52.04
C
ANISOU  423  C   ALA A  46     4714   7101   7958    752    625   -544
ATOM    424  O   ALA A  46       6.607   4.781   9.435  1.00 52.41
O
ANISOU  424  O   ALA A  46     4815   7084   8015    782    855   -541
ATOM    425  CB  ALA A  46       5.389   4.180   6.572  1.00 50.10
C
ANISOU  425  CB  ALA A  46     3984   6882   8171   1043      7   -750
ATOM    426  H   ALA A  46       7.231   2.757   5.641  1.00  0.00
H
```

```
ATOM    427  HA  ALA A  46       7.161   5.240   7.026  1.00  0.00
H
ATOM    428  HB1 ALA A  46       4.744   4.802   7.193  1.00  0.00
H
ATOM    429  HB2 ALA A  46       5.385   4.560   5.550  1.00  0.00
H
ATOM    430  HB3 ALA A  46       5.020   3.154   6.579  1.00  0.00
H
ATOM    431  N   PHE A  47       6.978   2.593   8.982  1.00 49.05
N
ANISOU  431  N   PHE A  47      4372   6708   7557    571    756   -540
ATOM    432  CA  PHE A  47       6.946   2.135  10.376  1.00 50.22
C
ANISOU  432  CA  PHE A  47      4678   6746   7659    404   1186   -522
ATOM    433  C   PHE A  47       8.011   1.039  10.667  1.00 51.02
C
ANISOU  433  C   PHE A  47      5087   6853   7446    267   1190   -407
ATOM    434  O   PHE A  47       8.603   0.535   9.707  1.00 49.26
O
ANISOU  434  O   PHE A  47      4857   6723   7136    283    890   -371
ATOM    435  CB  PHE A  47       5.513   1.704  10.776  1.00 56.55
C
ANISOU  435  CB  PHE A  47      5129   7418   8942    323   1496   -693
ATOM    436  CG  PHE A  47       4.903   0.567   9.987  1.00 60.84
C
ANISOU  436  CG  PHE A  47      5336   7962   9817    255   1352   -818
ATOM    437  CD1 PHE A  47       4.206   0.812   8.806  1.00 65.07
C
ANISOU  437  CD1 PHE A  47      5490   8547  10685    398    975   -961
ATOM    438  CD2 PHE A  47       4.964  -0.742  10.459  1.00 64.70
C
ANISOU  438  CD2 PHE A  47      5907   8372  10303     60   1582   -808
ATOM    439  CE1 PHE A  47       3.629  -0.241   8.083  1.00 69.37
C
ANISOU  439  CE1 PHE A  47      5728   9074  11556    339    796  -1110
ATOM    440  CE2 PHE A  47       4.383  -1.792   9.737  1.00 66.86
C
ANISOU  440  CE2 PHE A  47      5857   8629  10918    -14   1452   -944
ATOM    441  CZ  PHE A  47       3.723  -1.534   8.554  1.00 67.19
C
ANISOU  441  CZ  PHE A  47      5504   8729  11295    123   1049  -1105
ATOM    442  H   PHE A  47       7.144   1.888   8.278  1.00  0.00
H
ATOM    443  HA  PHE A  47       7.201   2.995  10.996  1.00  0.00
H
ATOM    444  HB3 PHE A  47       4.852   2.569  10.729  1.00  0.00
H
ATOM    445  HB2 PHE A  47       5.496   1.454  11.837  1.00  0.00
H
ATOM    446  HD1 PHE A  47       4.105   1.822   8.438  1.00  0.00
H
ATOM    447  HD2 PHE A  47       5.464  -0.957  11.392  1.00  0.00
H
ATOM    448  HE1 PHE A  47       3.111  -0.040   7.157  1.00  0.00
H
ATOM    449  HE2 PHE A  47       4.452  -2.804  10.107  1.00  0.00
H
```

```
ATOM    450  HZ  PHE A  47       3.279  -2.345   7.996  1.00  0.00
H
ATOM    451  N   PRO A  48       8.320   0.693  11.955  1.00 46.72
N
ANISOU  451  N   PRO A  48     4860   6190   6701    154   1505   -346
ATOM    452  CA  PRO A  48       9.362  -0.323  12.217  1.00 44.87
C
ANISOU  452  CA  PRO A  48     4931   5940   6176     61   1441   -240
ATOM    453  C   PRO A  48       9.086  -1.700  11.618  1.00 48.00
C
ANISOU  453  C   PRO A  48     5151   6343   6743    -47   1402   -278
ATOM    454  O   PRO A  48       7.941  -2.159  11.627  1.00 48.16
O
ANISOU  454  O   PRO A  48     4901   6290   7108   -123   1617   -395
ATOM    455  CB  PRO A  48       9.405  -0.403  13.752  1.00 48.14
C
ANISOU  455  CB  PRO A  48     5743   6176   6372     -8   1799   -196
ATOM    456  CG  PRO A  48       8.817   0.872  14.226  1.00 53.67
C
ANISOU  456  CG  PRO A  48     6415   6836   7141     72   1982   -251
ATOM    457  CD  PRO A  48       7.763   1.212  13.225  1.00 50.13
C
ANISOU  457  CD  PRO A  48     5445   6469   7133    128   1920   -373
ATOM    458  HA  PRO A  48      10.321   0.043  11.849  1.00  0.00
H
ATOM    459  HB3 PRO A  48      10.442  -0.468  14.082  1.00  0.00
H
ATOM    460  HB2 PRO A  48       8.785  -1.234  14.088  1.00  0.00
H
ATOM    461  HG3 PRO A  48       9.583   1.647  14.222  1.00  0.00
H
ATOM    462  HG2 PRO A  48       8.354   0.716  15.200  1.00  0.00
H
ATOM    463  HD2 PRO A  48       6.849   0.669  13.466  1.00  0.00
H
ATOM    464  HD3 PRO A  48       7.666   2.296  13.159  1.00  0.00
H
ATOM    465  N   VAL A  49      10.146  -2.363  11.104  1.00 42.81
N
ANISOU  465  N   VAL A  49     4627   5749   5888    -58   1141   -196
ATOM    466  CA  VAL A  49      10.033  -3.709  10.532  1.00 42.55
C
ANISOU  466  CA  VAL A  49     4476   5715   5977   -159   1086   -226
ATOM    467  C   VAL A  49       9.985  -4.712  11.695  1.00 47.73
C
ANISOU  467  C   VAL A  49     5389   6194   6552   -303   1419   -182
ATOM    468  O   VAL A  49      10.879  -4.716  12.545  1.00 47.81
O
ANISOU  468  O   VAL A  49     5802   6128   6235   -292   1445    -70
ATOM    469  CB  VAL A  49      11.168  -4.056   9.514  1.00 43.60
C
ANISOU  469  CB  VAL A  49     4669   5951   5947   -114    731   -163
ATOM    470  CG1 VAL A  49      10.922  -5.414   8.863  1.00 43.75
C
ANISOU  470  CG1 VAL A  49     4548   5960   6114   -214    679   -218
ATOM    471  CG2 VAL A  49      11.319  -2.982   8.442  1.00 41.92
C
```

```
ANISOU  471  CG2 VAL A  49      4334   5859   5735     41    467   -179
ATOM    472  H   VAL A  49       11.060  -1.932  11.104  1.00  0.00
H
ATOM    473  HA  VAL A  49        9.082  -3.767  10.003  1.00  0.00
H
ATOM    474  HB  VAL A  49       12.107  -4.114  10.065  1.00  0.00
H
ATOM    475 HG11 VAL A  49       11.726  -5.631   8.159  1.00  0.00
H
ATOM    476 HG12 VAL A  49       10.894  -6.186   9.632  1.00  0.00
H
ATOM    477 HG13 VAL A  49        9.970  -5.396   8.332  1.00  0.00
H
ATOM    478 HG21 VAL A  49       12.118  -3.263   7.756  1.00  0.00
H
ATOM    479 HG22 VAL A  49       10.384  -2.885   7.890  1.00  0.00
H
ATOM    480 HG23 VAL A  49       11.563  -2.030   8.913  1.00  0.00
H
ATOM    481  N   THR A  50        8.942  -5.547  11.733  1.00 45.33
N
ANISOU  481  N   THR A  50      4872   5792   6558   -427   1664   -282
ATOM    482  CA  THR A  50        8.767  -6.562  12.779  1.00 46.60
C
ANISOU  482  CA  THR A  50      5300   5737   6670   -572   2056   -241
ATOM    483  C   THR A  50        9.238  -7.917  12.255  1.00 49.57
C
ANISOU  483  C   THR A  50      5694   6103   7037   -658   1919   -214
ATOM    484  O   THR A  50        9.315  -8.113  11.037  1.00 47.22
O
ANISOU  484  O   THR A  50      5103   5952   6886   -631   1593   -280
ATOM    485  CB  THR A  50        7.291  -6.658  13.205  1.00 53.38
C
ANISOU  485  CB  THR A  50      5901   6429   7950   -679   2525   -385
ATOM    486  OG1 THR A  50        6.545  -7.226  12.129  1.00 51.92
O
ANISOU  486  OG1 THR A  50      5190   6303   8233   -726   2377   -555
ATOM    487  CG2 THR A  50        6.697  -5.315  13.627  1.00 54.11
C
ANISOU  487  CG2 THR A  50      5944   6508   8106   -593   2702   -431
ATOM    488  H   THR A  50        8.228  -5.496  11.020  1.00  0.00
H
ATOM    489  HA  THR A  50        9.369  -6.287  13.645  1.00  0.00
H
ATOM    490  HB  THR A  50        7.228  -7.337  14.055  1.00  0.00
H
ATOM    491  HG1 THR A  50        5.699  -7.540  12.456  1.00  0.00
H
ATOM    492 HG21 THR A  50        5.655  -5.453  13.915  1.00  0.00
H
ATOM    493 HG22 THR A  50        7.258  -4.919  14.473  1.00  0.00
H
ATOM    494 HG23 THR A  50        6.754  -4.614  12.794  1.00  0.00
H
ATOM    495  N   ASP A  51        9.501  -8.865  13.169  1.00 48.82
N
ANISOU  495  N   ASP A  51      5982   5809   6760   -752   2180   -121
```

```
ATOM    496  CA  ASP A  51       9.907 -10.228  12.822  1.00 49.36
C
ANISOU  496  CA  ASP A  51      6110   5820   6825   -840   2111    -89
ATOM    497  C   ASP A  51       8.772 -10.998  12.125  1.00 56.31
C
ANISOU  497  C   ASP A  51      6531   6664   8198   -979   2249   -261
ATOM    498  O   ASP A  51       9.048 -11.918  11.354  1.00 55.14
O
ANISOU  498  O   ASP A  51      6272   6554   8125  -1024   2047   -287
ATOM    499  CB  ASP A  51      10.416 -10.979  14.062  1.00 53.20
C
ANISOU  499  CB  ASP A  51      7179   6057   6978   -880   2366     58
ATOM    500  CG  ASP A  51      11.781 -10.547  14.575  1.00 60.32
C
ANISOU  500  CG  ASP A  51      8524   6978   7418   -730   2049    203
ATOM    501  OD1 ASP A  51      12.315  -9.527  14.076  1.00 58.48
O
ANISOU  501  OD1 ASP A  51      8139   6942   7139   -612   1711    190
ATOM    502  OD2 ASP A  51      12.311 -11.222  15.482  1.00 66.26
O1-
ANISOU  502  OD2 ASP A  51      9781   7520   7874   -724   2130    318
ATOM    503  H   ASP A  51       9.422  -8.653  14.153  1.00  0.00
H
ATOM    504  HA  ASP A  51      10.736 -10.155  12.118  1.00  0.00
H
ATOM    505  HB3 ASP A  51      10.432 -12.049  13.854  1.00  0.00
H
ATOM    506  HB2 ASP A  51       9.684 -10.887  14.864  1.00  0.00
H
ATOM    507  N   ALA A  52       7.504 -10.598  12.372  1.00 56.69
N
ANISOU  507  N   ALA A  52      6290   6626   8624  -1041   2579   -403
ATOM    508  CA  ALA A  52       6.319 -11.184  11.733  1.00 59.23
C
ANISOU  508  CA  ALA A  52      6089   6885   9530  -1164   2684   -624
ATOM    509  C   ALA A  52       6.355 -10.843  10.240  1.00 61.87
C
ANISOU  509  C   ALA A  52      6018   7471  10017  -1050   2111   -745
ATOM    510  O   ALA A  52       6.186 -11.732   9.407  1.00 62.31
O
ANISOU  510  O   ALA A  52      5838   7531  10305  -1116   1931   -861
ATOM    511  CB  ALA A  52       5.050 -10.630  12.370  1.00 63.46
C
ANISOU  511  CB  ALA A  52      6389   7250  10473  -1227   3155   -761
ATOM    512  H   ALA A  52       7.329  -9.853  13.031  1.00  0.00
H
ATOM    513  HA  ALA A  52       6.341 -12.267  11.856  1.00  0.00
H
ATOM    514  HB1 ALA A  52       4.178 -11.072  11.888  1.00  0.00
H
ATOM    515  HB2 ALA A  52       5.040 -10.874  13.432  1.00  0.00
H
ATOM    516  HB3 ALA A  52       5.023  -9.547  12.246  1.00  0.00
H
ATOM    517  N   ILE A  53       6.645  -9.565   9.914  1.00 56.38
N
ANISOU  517  N   ILE A  53      5312   6962   9149   -872   1827   -711
```

```
ATOM    518  CA  ILE A  53       6.767  -9.045   8.550  1.00 54.55
C
ANISOU  518  CA  ILE A  53      4834   6938   8953   -723   1302   -791
ATOM    519  C   ILE A  53       8.057  -9.561   7.882  1.00 55.37
C
ANISOU  519  C   ILE A  53      5201   7154   8684   -682    978   -667
ATOM    520  O   ILE A  53       8.010  -9.993   6.727  1.00 55.46
O
ANISOU  520  O   ILE A  53      5040   7233   8798   -655    661   -771
ATOM    521  CB  ILE A  53       6.614  -7.493   8.549  1.00 56.94
C
ANISOU  521  CB  ILE A  53      5084   7347   9204   -550   1194   -784
ATOM    522  CG1 ILE A  53       5.129  -7.103   8.818  1.00 61.31
C
ANISOU  522  CG1 ILE A  53      5229   7778  10287   -579   1450   -979
ATOM    523  CG2 ILE A  53       7.119  -6.847   7.252  1.00 54.89
C
ANISOU  523  CG2 ILE A  53      4776   7277   8801   -364    670   -792
ATOM    524  CD1 ILE A  53       4.917  -5.734   9.535  1.00 70.22
C
ANISOU  524  CD1 ILE A  53      6395   8927  11358   -455   1568   -943
ATOM    525  H   ILE A  53       6.797  -8.881  10.641  1.00  0.00
H
ATOM    526  HA  ILE A  53       5.930  -9.448   7.980  1.00  0.00
H
ATOM    527  HB  ILE A  53       7.214  -7.100   9.370  1.00  0.00
H
ATOM    528 HG13 ILE A  53       4.649  -7.891   9.398  1.00  0.00
H
ATOM    529 HG12 ILE A  53       4.580  -7.105   7.877  1.00  0.00
H
ATOM    530 HG21 ILE A  53       6.987  -5.766   7.308  1.00  0.00
H
ATOM    531 HG22 ILE A  53       8.176  -7.077   7.118  1.00  0.00
H
ATOM    532 HG23 ILE A  53       6.553  -7.238   6.406  1.00  0.00
H
ATOM    533 HD11 ILE A  53       3.850  -5.558   9.675  1.00  0.00
H
ATOM    534 HD12 ILE A  53       5.412  -5.751  10.506  1.00  0.00
H
ATOM    535 HD13 ILE A  53       5.340  -4.935   8.926  1.00  0.00
H
ATOM    536  N   ALA A  54       9.191  -9.549   8.611  1.00 48.90
N
ANISOU  536  N   ALA A  54      4800   6322   7457   -672   1055   -467
ATOM    537  CA  ALA A  54      10.464 -10.031   8.075  1.00 45.84
C
ANISOU  537  CA  ALA A  54      4634   5998   6784   -635    798   -361
ATOM    538  C   ALA A  54      11.158 -11.024   9.023  1.00 49.20
C
ANISOU  538  C   ALA A  54      5404   6270   7021   -732   1000   -231
ATOM    539  O   ALA A  54      11.875 -10.604   9.935  1.00 46.61
O
ANISOU  539  O   ALA A  54      5397   5892   6419   -682   1068   -100
ATOM    540  CB  ALA A  54      11.378  -8.862   7.736  1.00 44.09
C
```

```
ANISOU  540  CB  ALA A  54     4544   5910   6298   -470    556   -271
ATOM    541  H   ALA A  54       9.179  -9.200   9.559  1.00  0.00
H
ATOM    542  HA  ALA A  54      10.250 -10.560   7.146  1.00  0.00
H
ATOM    543  HB1 ALA A  54      12.320  -9.240   7.339  1.00  0.00
H
ATOM    544  HB2 ALA A  54      10.898  -8.229   6.990  1.00  0.00
H
ATOM    545  HB3 ALA A  54      11.572  -8.279   8.636  1.00  0.00
H
ATOM    546  N   PRO A  55      10.950 -12.353   8.816  1.00 48.12
N
ANISOU  546  N   PRO A  55     5225   6032   7026   -859   1073   -275
ATOM    547  CA  PRO A  55      11.572 -13.358   9.702  1.00 48.32
C
ANISOU  547  CA  PRO A  55     5616   5876   6867   -933   1257   -144
ATOM    548  C   PRO A  55      13.056 -13.156  10.004  1.00 48.62
C
ANISOU  548  C   PRO A  55     5999   5931   6541   -814   1035     15
ATOM    549  O   PRO A  55      13.858 -12.932   9.099  1.00 45.54
O
ANISOU  549  O   PRO A  55     5526   5671   6107   -730    728     12
ATOM    550  CB  PRO A  55      11.315 -14.672   8.965  1.00 51.23
C
ANISOU  550  CB  PRO A  55     5820   6182   7464  -1055   1251   -237
ATOM    551  CG  PRO A  55      10.034 -14.435   8.247  1.00 57.25
C
ANISOU  551  CG  PRO A  55     6124   7007   8622  -1098   1234   -450
ATOM    552  CD  PRO A  55      10.106 -13.004   7.789  1.00 51.27
C
ANISOU  552  CD  PRO A  55     5263   6450   7768   -931    972   -459
ATOM    553  HA  PRO A  55      11.025 -13.376  10.645  1.00  0.00
H
ATOM    554  HB3 PRO A  55      11.178 -15.472   9.692  1.00  0.00
H
ATOM    555  HB2 PRO A  55      12.110 -14.839   8.238  1.00  0.00
H
ATOM    556  HG3 PRO A  55       9.203 -14.550   8.943  1.00  0.00
H
ATOM    557  HG2 PRO A  55       9.980 -15.090   7.378  1.00  0.00
H
ATOM    558  HD2 PRO A  55      10.613 -12.959   6.825  1.00  0.00
H
ATOM    559  HD3 PRO A  55       9.108 -12.567   7.814  1.00  0.00
H
ATOM    560  N   GLY A  56      13.381 -13.195  11.293  1.00 46.01
N
ANISOU  560  N   GLY A  56     6068   5440   5973   -799   1198    136
ATOM    561  CA  GLY A  56      14.737 -13.042  11.803  1.00 44.54
C
ANISOU  561  CA  GLY A  56     6224   5212   5486   -676    959    259
ATOM    562  C   GLY A  56      15.345 -11.665  11.631  1.00 45.04
C
ANISOU  562  C   GLY A  56     6226   5428   5461   -539    715    258
ATOM    563  O   GLY A  56      16.573 -11.546  11.630  1.00 44.49
O
```

```
ANISOU  563  O   GLY A  56     6267  5353  5285  -442   437   304
ATOM    564  H   GLY A  56      12.665 -13.339  11.990  1.00  0.00
H
ATOM    565  HA3 GLY A  56      15.384 -13.783  11.333  1.00  0.00
H
ATOM    566  HA2 GLY A  56      14.758 -13.316  12.858  1.00  0.00
H
ATOM    567  N   TYR A  57      14.498 -10.615  11.483  1.00 39.18
N
ANISOU  567  N   TYR A  57     5284  4796  4806  -529   824   193
ATOM    568  CA  TYR A  57      14.944  -9.229  11.294  1.00 35.84
C
ANISOU  568  CA  TYR A  57     4799  4501  4317  -406   638   187
ATOM    569  C   TYR A  57      15.748  -8.734  12.497  1.00 38.25
C
ANISOU  569  C   TYR A  57     5495  4688  4350  -318   573   268
ATOM    570  O   TYR A  57      16.838  -8.194  12.309  1.00 36.00
O
ANISOU  570  O   TYR A  57     5225  4435  4017  -220   295   278
ATOM    571  CB  TYR A  57      13.758  -8.278  11.011  1.00 36.40
C
ANISOU  571  CB  TYR A  57     4595  4681  4556  -404   777    98
ATOM    572  CG  TYR A  57      14.189  -6.918  10.504  1.00 35.72
C
ANISOU  572  CG  TYR A  57     4407  4729  4436  -276   575    89
ATOM    573  CD1 TYR A  57      14.499  -6.718   9.161  1.00 35.95
C
ANISOU  573  CD1 TYR A  57     4220  4885  4556  -222   355    52
ATOM    574  CD2 TYR A  57      14.296  -5.831  11.367  1.00 36.40
C
ANISOU  574  CD2 TYR A  57     4660  4787  4384  -206   629   116
ATOM    575  CE1 TYR A  57      14.908  -5.474   8.690  1.00 34.17
C
ANISOU  575  CE1 TYR A  57     3950  4739  4293  -104   224    55
ATOM    576  CE2 TYR A  57      14.701  -4.578  10.907  1.00 35.92
C
ANISOU  576  CE2 TYR A  57     4509  4824  4315   -95   470   105
ATOM    577  CZ  TYR A  57      15.010  -4.405   9.566  1.00 40.56
C
ANISOU  577  CZ  TYR A  57     4884  5523  5005   -47   282    81
ATOM    578  OH  TYR A  57      15.432  -3.181   9.103  1.00 38.65
O
ANISOU  578  OH  TYR A  57     4601  5335  4749    60   177    82
ATOM    579  H   TYR A  57      13.499 -10.765  11.500  1.00  0.00
H
ATOM    580  HA  TYR A  57      15.601  -9.208  10.425  1.00  0.00
H
ATOM    581  HB3 TYR A  57      13.168  -8.155  11.919  1.00  0.00
H
ATOM    582  HB2 TYR A  57      13.089  -8.740  10.285  1.00  0.00
H
ATOM    583  HD1 TYR A  57      14.424  -7.538   8.462  1.00  0.00
H
ATOM    584  HD2 TYR A  57      14.063  -5.949  12.415  1.00  0.00
H
ATOM    585  HE1 TYR A  57      15.145  -5.344   7.644  1.00  0.00
H
```

```
ATOM    586  HE2 TYR A  57      14.773  -3.748  11.594  1.00  0.00
H
ATOM    587  HH  TYR A  57      15.288  -3.130   8.155  1.00  0.00
H
ATOM    588  N   SER A  58      15.216  -8.931  13.720  1.00 37.18
N
ANISOU  588  N   SER A  58     5694   4383   4051   -352    836    309
ATOM    589  CA  SER A  58      15.842  -8.487  14.976  1.00 38.29
C
ANISOU  589  CA  SER A  58     6310   4367   3870   -254    767    372
ATOM    590  C   SER A  58      17.181  -9.195  15.273  1.00 43.09
C
ANISOU  590  C   SER A  58     7189   4850   4332   -173    421    431
ATOM    591  O   SER A  58      18.028  -8.634  15.969  1.00 42.89
O
ANISOU  591  O   SER A  58     7437   4737   4121    -49    162    438
ATOM    592  CB  SER A  58      14.874  -8.636  16.145  1.00 42.68
C
ANISOU  592  CB  SER A  58     7233   4725   4257   -310   1195    404
ATOM    593  OG  SER A  58      14.569 -10.002  16.357  1.00 47.37
O
ANISOU  593  OG  SER A  58     7978   5161   4861   -412   1414    451
ATOM    594  H   SER A  58      14.332  -9.411  13.815  1.00  0.00
H
ATOM    595  HA  SER A  58      16.057  -7.424  14.870  1.00  0.00
H
ATOM    596  HB3 SER A  58      13.957  -8.089  15.929  1.00  0.00
H
ATOM    597  HB2 SER A  58      15.326  -8.221  17.046  1.00  0.00
H
ATOM    598  HG  SER A  58      13.720 -10.206  15.958  1.00  0.00
H
ATOM    599  N   MET A  59      17.364 -10.409  14.726  1.00 40.95
N
ANISOU  599  N   MET A  59     6820   4556   4182   -237    394    452
ATOM    600  CA  MET A  59      18.570 -11.232  14.835  1.00 42.50
C
ANISOU  600  CA  MET A  59     7191   4625   4332   -164     77    494
ATOM    601  C   MET A  59      19.733 -10.576  14.062  1.00 41.70
C
ANISOU  601  C   MET A  59     6790   4638   4415    -74   -295    428
ATOM    602  O   MET A  59      20.884 -10.714  14.471  1.00 40.70
O
ANISOU  602  O   MET A  59     6829   4375   4262     39   -627    427
ATOM    603  CB  MET A  59      18.259 -12.631  14.260  1.00 46.54
C
ANISOU  603  CB  MET A  59     7593   5102   4986   -279    221    514
ATOM    604  CG  MET A  59      19.442 -13.600  14.188  1.00 52.94
C
ANISOU  604  CG  MET A  59     8540   5770   5803   -209    -76    552
ATOM    605  SD  MET A  59      19.685 -14.676  15.626  1.00 62.96
S
ANISOU  605  SD  MET A  59    10517   6681   6722   -138    -65    679
ATOM    606  CE  MET A  59      18.040 -15.418  15.797  1.00 61.36
C
ANISOU  606  CE  MET A  59    10353   6418   6542   -350    550    713
```

```
ATOM    607  H   MET A  59      16.628 -10.833  14.180  1.00  0.00
H
ATOM    608  HA  MET A  59      18.846 -11.328  15.885  1.00  0.00
H
ATOM    609  HB3 MET A  59      17.822 -12.523  13.268  1.00  0.00
H
ATOM    610  HB2 MET A  59      17.455 -13.087  14.838  1.00  0.00
H
ATOM    611  HG3 MET A  59      20.357 -13.037  14.002  1.00  0.00
H
ATOM    612  HG2 MET A  59      19.350 -14.213  13.292  1.00  0.00
H
ATOM    613  HE1 MET A  59      18.037 -16.105  16.643  1.00  0.00
H
ATOM    614  HE2 MET A  59      17.302 -14.633  15.964  1.00  0.00
H
ATOM    615  HE3 MET A  59      17.790 -15.963  14.887  1.00  0.00
H
ATOM    616  N   ILE A  60      19.416  -9.864  12.961  1.00 35.89
N
ANISOU  616  N   ILE A  60     5632   4117   3887   -116   -229    364
ATOM    617  CA  ILE A  60      20.368  -9.207  12.045  1.00 33.88
C
ANISOU  617  CA  ILE A  60     5088   3951   3835    -56   -451    302
ATOM    618  C   ILE A  60      20.520  -7.712  12.317  1.00 35.53
C
ANISOU  618  C   ILE A  60     5277   4207   4015     20   -515    263
ATOM    619  O   ILE A  60      21.644  -7.210  12.387  1.00 35.49
O
ANISOU  619  O   ILE A  60     5246   4129   4110    105   -764    216
ATOM    620  CB  ILE A  60      19.943  -9.455  10.555  1.00 36.22
C
ANISOU  620  CB  ILE A  60     5025   4408   4328   -133   -331    264
ATOM    621  CG1 ILE A  60      19.653 -10.968  10.254  1.00 38.72
C
ANISOU  621  CG1 ILE A  60     5347   4678   4688   -227   -241    282
ATOM    622  CG2 ILE A  60      20.944  -8.844   9.544  1.00 36.12
C
ANISOU  622  CG2 ILE A  60     4790   4430   4507    -74   -471    211
ATOM    623  CD1 ILE A  60      20.797 -11.982  10.493  1.00 45.36
C
ANISOU  623  CD1 ILE A  60     6317   5345   5574   -193   -426    309
ATOM    624  H   ILE A  60      18.448  -9.742  12.700  1.00  0.00
H
ATOM    625  HA  ILE A  60      21.343  -9.671  12.193  1.00  0.00
H
ATOM    626  HB  ILE A  60      19.000  -8.927  10.412  1.00  0.00
H
ATOM    627 HG13 ILE A  60      19.296 -11.067   9.229  1.00  0.00
H
ATOM    628 HG12 ILE A  60      18.774 -11.282  10.816  1.00  0.00
H
ATOM    629 HG21 ILE A  60      20.603  -9.044   8.528  1.00  0.00
H
ATOM    630 HG22 ILE A  60      21.007  -7.767   9.700  1.00  0.00
H
```

```
ATOM    631 HG23 ILE A  60      21.928  -9.290   9.691  1.00  0.00
H
ATOM    632 HD11 ILE A  60      20.452 -12.985  10.243  1.00  0.00
H
ATOM    633 HD12 ILE A  60      21.649 -11.724   9.864  1.00  0.00
H
ATOM    634 HD13 ILE A  60      21.097 -11.952  11.540  1.00  0.00
H
ATOM    635  N   ILE A  61      19.393  -6.999  12.406  1.00 30.64
N
ANISOU  635  N   ILE A  61     4626   3695   3318    -13   -285    262
ATOM    636  CA  ILE A  61      19.360  -5.544  12.580  1.00 29.94
C
ANISOU  636  CA  ILE A  61     4505   3661   3211     52   -297    225
ATOM    637  C   ILE A  61      19.233  -5.164  14.057  1.00 36.53
C
ANISOU  637  C   ILE A  61     5735   4359   3787    102   -291    239
ATOM    638  O   ILE A  61      18.177  -5.359  14.663  1.00 36.57
O
ANISOU  638  O   ILE A  61     5915   4336   3643     52    -15    271
ATOM    639  CB  ILE A  61      18.254  -4.931  11.673  1.00 31.28
C
ANISOU  639  CB  ILE A  61     4389   4012   3484     16    -85    201
ATOM    640  CG1 ILE A  61      18.462  -5.314  10.174  1.00 29.98
C
ANISOU  640  CG1 ILE A  61     3940   3947   3506     -5   -133    181
ATOM    641  CG2 ILE A  61      18.096  -3.413  11.877  1.00 31.01
C
ANISOU  641  CG2 ILE A  61     4334   4021   3429     89    -65    170
ATOM    642  CD1 ILE A  61      19.894  -5.013   9.548  1.00 34.55
C
ANISOU  642  CD1 ILE A  61     4435   4477   4214     54   -316    160
ATOM    643  H   ILE A  61      18.497  -7.462  12.354  1.00  0.00
H
ATOM    644  HA  ILE A  61      20.316  -5.156  12.229  1.00  0.00
H
ATOM    645  HB  ILE A  61      17.312  -5.385  11.982  1.00  0.00
H
ATOM    646 HG13 ILE A  61      17.697  -4.827   9.570  1.00  0.00
H
ATOM    647 HG12 ILE A  61      18.225  -6.369  10.037  1.00  0.00
H
ATOM    648 HG21 ILE A  61      17.312  -3.037  11.220  1.00  0.00
H
ATOM    649 HG22 ILE A  61      17.828  -3.211  12.914  1.00  0.00
H
ATOM    650 HG23 ILE A  61      19.036  -2.914  11.642  1.00  0.00
H
ATOM    651 HD11 ILE A  61      19.907  -5.325   8.504  1.00  0.00
H
ATOM    652 HD12 ILE A  61      20.101  -3.945   9.610  1.00  0.00
H
ATOM    653 HD13 ILE A  61      20.656  -5.563  10.101  1.00  0.00
H
ATOM    654  N   LYS A  62      20.329  -4.619  14.622  1.00 34.55
N
ANISOU  654  N   LYS A  62     5631   3992   3503    203   -591    196
```

```
ATOM    655  CA  LYS A  62      20.418  -4.240  16.033  1.00 37.02
C
ANISOU  655  CA  LYS A  62     6396   4139   3533    285   -686    186
ATOM    656  C   LYS A  62      20.003  -2.797  16.286  1.00 43.02
C
ANISOU  656  C   LYS A  62     7143   4954   4247    315   -581    135
ATOM    657  O   LYS A  62      19.643  -2.452  17.415  1.00 45.37
O
ANISOU  657  O   LYS A  62     7842   5134   4261    356   -504    136
ATOM    658  CB  LYS A  62      21.842  -4.497  16.579  1.00 40.44
C
ANISOU  658  CB  LYS A  62     7011   4376   3978    400  -1154    131
ATOM    659  CG  LYS A  62      22.311  -5.952  16.478  1.00 45.65
C
ANISOU  659  CG  LYS A  62     7750   4932   4664    399  -1289    182
ATOM    660  CD  LYS A  62      21.558  -6.871  17.435  1.00 55.19
C
ANISOU  660  CD  LYS A  62     9477   5998   5493    392  -1111    287
ATOM    661  CE  LYS A  62      21.293  -8.221  16.830  1.00 57.40
C
ANISOU  661  CE  LYS A  62     9654   6295   5861    298   -949    365
ATOM    662  NZ  LYS A  62      20.353  -8.998  17.673  1.00 62.45
N1+
ANISOU  662  NZ  LYS A  62    10758   6793   6177    253   -629    468
ATOM    663  H   LYS A  62      21.154  -4.450  14.064  1.00  0.00
H
ATOM    664  HA  LYS A  62      19.733  -4.881  16.589  1.00  0.00
H
ATOM    665  HB3 LYS A  62      21.894  -4.172  17.618  1.00  0.00
H
ATOM    666  HB2 LYS A  62      22.550  -3.853  16.058  1.00  0.00
H
ATOM    667  HG3 LYS A  62      23.379  -6.004  16.690  1.00  0.00
H
ATOM    668  HG2 LYS A  62      22.178  -6.306  15.456  1.00  0.00
H
ATOM    669  HD3 LYS A  62      20.612  -6.407  17.714  1.00  0.00
H
ATOM    670  HD2 LYS A  62      22.135  -6.992  18.352  1.00  0.00
H
ATOM    671  HE3 LYS A  62      22.232  -8.767  16.735  1.00  0.00
H
ATOM    672  HE2 LYS A  62      20.868  -8.095  15.834  1.00  0.00
H
ATOM    673  HZ1 LYS A  62      19.514  -8.458  17.828  1.00  0.00
H
ATOM    674  HZ2 LYS A  62      20.116  -9.861  17.206  1.00  0.00
H
ATOM    675  HZ3 LYS A  62      20.788  -9.205  18.561  1.00  0.00
H
ATOM    676  N   HIS A  63      20.076  -1.944  15.254  1.00 38.05
N
ANISOU  676  N   HIS A  63     6105   4476   3877    305   -562     92
ATOM    677  CA  HIS A  63      19.720  -0.535  15.387  1.00 38.38
C
ANISOU  677  CA  HIS A  63     6103   4565   3915    341   -465     44
```

```
ATOM    678  C   HIS A  63      18.645  -0.156  14.352  1.00 38.60
C
ANISOU  678  C   HIS A  63     5794   4787   4086    290   -173     72
ATOM    679  O   HIS A  63      18.957   0.466  13.326  1.00 36.29
O
ANISOU  679  O   HIS A  63     5213   4579   3995    309   -215     51
ATOM    680  CB  HIS A  63      20.987   0.341  15.325  1.00 39.98
C
ANISOU  680  CB  HIS A  63     6208   4688   4293    414   -771    -54
ATOM    681  CG  HIS A  63      22.049  -0.091  16.297  1.00 46.19
C
ANISOU  681  CG  HIS A  63     7286   5261   5001    488  -1150   -117
ATOM    682  ND1 HIS A  63      23.159  -0.839  16.092  1.00 50.96
N
ANISOU  682  ND1 HIS A  63     8350   5714   5297    565  -1261   -156
ATOM    683  CD2 HIS A  63      21.966   0.221  17.645  1.00 48.91
C
ANISOU  683  CD2 HIS A  63     7545   5501   5538    513  -1451   -156
ATOM    684  CE1 HIS A  63      23.784  -0.969  17.317  1.00 52.25
C
ANISOU  684  CE1 HIS A  63     8706   5686   5461    648  -1682   -221
ATOM    685  NE2 HIS A  63      23.036  -0.321  18.209  1.00 51.48
N
ANISOU  685  NE2 HIS A  63     8260   5609   5692    620  -1811   -227
ATOM    686  H   HIS A  63      20.385  -2.271  14.350  1.00  0.00
H
ATOM    687  HA  HIS A  63      19.281  -0.402  16.376  1.00  0.00
H
ATOM    688  HB3 HIS A  63      20.719   1.379  15.520  1.00  0.00
H
ATOM    689  HB2 HIS A  63      21.392   0.320  14.313  1.00  0.00
H
ATOM    690  HD2 HIS A  63      21.187   0.762  17.991  1.00  0.00
H
ATOM    691  HE1 HIS A  63      24.648  -1.487  17.391  1.00  0.00
H
ATOM    692  HE2 HIS A  63      23.205  -0.204  19.269  1.00  0.00
H
ATOM    693  N   PRO A  64      17.371  -0.573  14.578  1.00 35.79
N
ANISOU  693  N   PRO A  64     5479   4471   3649    231    124    107
ATOM    694  CA  PRO A  64      16.321  -0.278  13.584  1.00 34.97
C
ANISOU  694  CA  PRO A  64     5027   4526   3734    205    318    100
ATOM    695  C   PRO A  64      15.984   1.207  13.459  1.00 37.51
C
ANISOU  695  C   PRO A  64     5244   4896   4112    281    378     60
ATOM    696  O   PRO A  64      16.183   1.978  14.398  1.00 37.40
O
ANISOU  696  O   PRO A  64     5462   4788   3962    325    388     32
ATOM    697  CB  PRO A  64      15.119  -1.099  14.070  1.00 38.37
C
ANISOU  697  CB  PRO A  64     5519   4922   4138    119    620    112
ATOM    698  CG  PRO A  64      15.340  -1.273  15.528  1.00 45.18
C
ANISOU  698  CG  PRO A  64     6857   5594   4714    125    692    134
```

```
ATOM    699  CD  PRO A  64      16.830  -1.341  15.723  1.00 40.06
C
ANISOU  699  CD  PRO A  64     6391   4875   3956    192    306    142
ATOM    700  HA  PRO A  64      16.638  -0.654  12.611  1.00  0.00
H
ATOM    701  HB3 PRO A  64      15.128  -2.075  13.584  1.00  0.00
H
ATOM    702  HB2 PRO A  64      14.204  -0.530  13.909  1.00  0.00
H
ATOM    703  HG3 PRO A  64      14.889  -2.210  15.853  1.00  0.00
H
ATOM    704  HG2 PRO A  64      14.944  -0.408  16.060  1.00  0.00
H
ATOM    705  HD2 PRO A  64      17.095  -0.837  16.652  1.00  0.00
H
ATOM    706  HD3 PRO A  64      17.156  -2.378  15.648  1.00  0.00
H
ATOM    707  N   MET A  65      15.498   1.604  12.282  1.00 32.40
N
ANISOU  707  N   MET A  65     4284   4376   3650    310    391     51
ATOM    708  CA  MET A  65      15.102   2.986  11.984  1.00 31.40
C
ANISOU  708  CA  MET A  65     4043   4290   3598    401    438     23
ATOM    709  C   MET A  65      14.086   2.960  10.854  1.00 35.77
C
ANISOU  709  C   MET A  65     4303   4957   4331    434    472      8
ATOM    710  O   MET A  65      14.108   2.051  10.025  1.00 34.52
O
ANISOU  710  O   MET A  65     4031   4855   4230    400    379     19
ATOM    711  CB  MET A  65      16.329   3.857  11.625  1.00 31.97
C
ANISOU  711  CB  MET A  65     4142   4321   3686    463    260     24
ATOM    712  CG  MET A  65      16.051   5.377  11.596  1.00 35.05
C
ANISOU  712  CG  MET A  65     4504   4698   4116    558    328     -3
ATOM    713  SD  MET A  65      15.170   6.058  13.032  1.00 40.48
S
ANISOU  713  SD  MET A  65     5368   5320   4694    571    539    -54
ATOM    714  CE  MET A  65      16.368   5.758  14.355  1.00 38.38
C
ANISOU  714  CE  MET A  65     5475   4894   4213    532    385    -85
ATOM    715  H   MET A  65      15.384   0.937  11.532  1.00  0.00
H
ATOM    716  HA  MET A  65      14.625   3.408  12.869  1.00  0.00
H
ATOM    717  HB3 MET A  65      16.724   3.544  10.658  1.00  0.00
H
ATOM    718  HB2 MET A  65      17.136   3.651  12.328  1.00  0.00
H
ATOM    719  HG3 MET A  65      15.503   5.625  10.687  1.00  0.00
H
ATOM    720  HG2 MET A  65      16.992   5.912  11.465  1.00  0.00
H
ATOM    721  HE1 MET A  65      15.966   6.124  15.300  1.00  0.00
H
ATOM    722  HE2 MET A  65      16.563   4.688  14.434  1.00  0.00
H
```

```
ATOM    723  HE3 MET A  65      17.298   6.281  14.129  1.00  0.00
H
ATOM    724  N   ASP A  66      13.187   3.942  10.842  1.00 34.03
N
ANISOU  724  N   ASP A  66     3972   4753   4206    512    580    -32
ATOM    725  CA  ASP A  66      12.115   4.084   9.855  1.00 34.64
C
ANISOU  725  CA  ASP A  66     3771   4906   4484    586    549    -76
ATOM    726  C   ASP A  66      11.673   5.541   9.850  1.00 38.77
C
ANISOU  726  C   ASP A  66     4250   5409   5071    720    589    -99
ATOM    727  O   ASP A  66      11.977   6.266  10.801  1.00 37.74
O
ANISOU  727  O   ASP A  66     4288   5207   4843    719    711    -96
ATOM    728  CB  ASP A  66      10.928   3.161  10.201  1.00 37.79
C
ANISOU  728  CB  ASP A  66     3996   5297   5065    499    715   -151
ATOM    729  CG  ASP A  66      10.286   3.474  11.538  1.00 48.72
C
ANISOU  729  CG  ASP A  66     5466   6577   6470    452   1043   -191
ATOM    730  OD1 ASP A  66      10.803   2.999  12.566  1.00 50.20
O
ANISOU  730  OD1 ASP A  66     5948   6678   6447    361   1173   -148
ATOM    731  OD2 ASP A  66       9.292   4.235  11.557  1.00 49.72
O1-
ANISOU  731  OD2 ASP A  66     5398   6682   6812    524   1165   -269
ATOM    732  H   ASP A  66      13.215   4.661  11.551  1.00  0.00
H
ATOM    733  HA  ASP A  66      12.498   3.822   8.869  1.00  0.00
H
ATOM    734  HB3 ASP A  66      11.263   2.124  10.194  1.00  0.00
H
ATOM    735  HB2 ASP A  66      10.176   3.228   9.414  1.00  0.00
H
ATOM    736  N   PHE A  67      10.942   5.958   8.801  1.00 36.83
N
ANISOU  736  N   PHE A  67     3805   5207   4980    849    460   -131
ATOM    737  CA  PHE A  67      10.434   7.329   8.637  1.00 37.69
C
ANISOU  737  CA  PHE A  67     3862   5283   5175   1009    464   -151
ATOM    738  C   PHE A  67       9.559   7.819   9.791  1.00 44.03
C
ANISOU  738  C   PHE A  67     4589   6022   6119    992    734   -229
ATOM    739  O   PHE A  67       9.658   8.989  10.163  1.00 44.48
O
ANISOU  739  O   PHE A  67     4740   6020   6140   1070    815   -222
ATOM    740  CB  PHE A  67       9.687   7.485   7.303  1.00 40.79
C
ANISOU  740  CB  PHE A  67     4079   5708   5711   1174    216   -188
ATOM    741  CG  PHE A  67      10.541   7.528   6.054  1.00 41.29
C
ANISOU  741  CG  PHE A  67     4331   5778   5578   1258     -6   -100
ATOM    742  CD1 PHE A  67      11.688   8.315   6.001  1.00 42.59
C
ANISOU  742  CD1 PHE A  67     4747   5878   5557   1283     51     -3
ATOM    743  CD2 PHE A  67      10.152   6.854   4.904  1.00 44.08
C
```

```
ANISOU  743  CD2 PHE A  67      4627   6167   5954   1323   -255   -134
ATOM    744  CE1 PHE A  67       12.449   8.392   4.833  1.00 43.44
C
ANISOU  744  CE1 PHE A  67      5058   5941   5508   1356    -62     74
ATOM    745  CE2 PHE A  67       10.915   6.929   3.736  1.00 46.34
C
ANISOU  745  CE2 PHE A  67      5169   6420   6019   1413   -407    -52
ATOM    746  CZ  PHE A  67       12.051   7.706   3.704  1.00 43.23
C
ANISOU  746  CZ  PHE A  67      5035   5946   5447   1427   -275     58
ATOM    747  H   PHE A  67       10.709   5.313   8.060  1.00  0.00
H
ATOM    748  HA  PHE A  67       11.302   7.987   8.594  1.00  0.00
H
ATOM    749  HB3 PHE A  67        9.064   8.378   7.343  1.00  0.00
H
ATOM    750  HB2 PHE A  67        8.951   6.686   7.208  1.00  0.00
H
ATOM    751  HD1 PHE A  67       11.999   8.876   6.870  1.00  0.00
H
ATOM    752  HD2 PHE A  67        9.249   6.262   4.905  1.00  0.00
H
ATOM    753  HE1 PHE A  67       13.349   8.988   4.813  1.00  0.00
H
ATOM    754  HE2 PHE A  67       10.614   6.377   2.858  1.00  0.00
H
ATOM    755  HZ  PHE A  67       12.631   7.779   2.796  1.00  0.00
H
ATOM    756  N   GLY A  68        8.712   6.932  10.322  1.00 41.71
N
ANISOU  756  N   GLY A  68      4138   5712   5999    886    908   -308
ATOM    757  CA  GLY A  68        7.801   7.220  11.429  1.00 43.61
C
ANISOU  757  CA  GLY A  68      4318   5847   6404    847   1262   -394
ATOM    758  C   GLY A  68        8.535   7.562  12.708  1.00 46.39
C
ANISOU  758  C   GLY A  68      5055   6113   6459    777   1481   -343
ATOM    759  O   GLY A  68        8.168   8.524  13.389  1.00 47.19
O
ANISOU  759  O   GLY A  68      5213   6124   6592    830   1688   -387
ATOM    760  H   GLY A  68        8.673   5.991   9.958  1.00  0.00
H
ATOM    761  HA3 GLY A  68        7.156   6.358  11.601  1.00  0.00
H
ATOM    762  HA2 GLY A  68        7.147   8.048  11.154  1.00  0.00
H
ATOM    763  N   THR A  69        9.605   6.793  13.019  1.00 41.36
N
ANISOU  763  N   THR A  69      4696   5485   5534    674   1400   -263
ATOM    764  CA  THR A  69       10.460   7.026  14.187  1.00 41.20
C
ANISOU  764  CA  THR A  69      5088   5365   5201    629   1484   -230
ATOM    765  C   THR A  69       11.153   8.382  14.029  1.00 43.40
C
ANISOU  765  C   THR A  69      5447   5638   5404    741   1332   -218
ATOM    766  O   THR A  69       11.200   9.153  14.984  1.00 44.36
O
```

```
ANISOU  766  O   THR A  69     5797   5651   5408    760   1477   -255
ATOM    767  CB  THR A  69      11.435   5.852  14.388  1.00 46.80
C
ANISOU  767  CB  THR A  69     6020   6072   5690    525   1345   -165
ATOM    768  OG1 THR A  69      10.669   4.682  14.652  1.00 47.40
O
ANISOU  768  OG1 THR A  69     6046   6114   5850    418   1560   -181
ATOM    769  CG2 THR A  69      12.401   6.073  15.551  1.00 44.89
C
ANISOU  769  CG2 THR A  69     6226   5702   5126    512   1317   -152
ATOM    770  H   THR A  69       9.851   6.009  12.432  1.00  0.00
H
ATOM    771  HA  THR A  69       9.817   7.080  15.066  1.00  0.00
H
ATOM    772  HB  THR A  69      12.008   5.704  13.472  1.00  0.00
H
ATOM    773  HG1 THR A  69      10.204   4.417  13.855  1.00  0.00
H
ATOM    774 HG21 THR A  69      13.063   5.212  15.643  1.00  0.00
H
ATOM    775 HG22 THR A  69      12.994   6.969  15.366  1.00  0.00
H
ATOM    776 HG23 THR A  69      11.836   6.196  16.475  1.00  0.00
H
ATOM    777  N   MET A  70      11.651   8.680  12.810  1.00 37.12
N
ANISOU  777  N   MET A  70     4493   4933   4679    816   1071   -173
ATOM    778  CA  MET A  70      12.290   9.956  12.493  1.00 35.59
C
ANISOU  778  CA  MET A  70     4355   4706   4463    918    965   -157
ATOM    779  C   MET A  70      11.280  11.109  12.635  1.00 41.05
C
ANISOU  779  C   MET A  70     4942   5353   5303   1034   1129   -211
ATOM    780  O   MET A  70      11.620  12.151  13.197  1.00 40.21
O
ANISOU  780  O   MET A  70     4991   5156   5132   1076   1187   -233
ATOM    781  CB  MET A  70      12.861   9.923  11.081  1.00 36.13
C
ANISOU  781  CB  MET A  70     4315   4837   4575    975    740    -90
ATOM    782  CG  MET A  70      14.113   9.064  10.963  1.00 37.94
C
ANISOU  782  CG  MET A  70     4654   5066   4696    873    598    -50
ATOM    783  SD  MET A  70      14.535   8.741   9.245  1.00 40.54
S
ANISOU  783  SD  MET A  70     4880   5449   5073    925    432     21
ATOM    784  CE  MET A  70      15.138  10.287   8.746  1.00 38.02
C
ANISOU  784  CE  MET A  70     4651   5012   4781   1041    454     48
ATOM    785  H   MET A  70      11.592   8.005  12.061  1.00  0.00
H
ATOM    786  HA  MET A  70      13.107  10.120  13.195  1.00  0.00
H
ATOM    787  HB3 MET A  70      13.087  10.939  10.758  1.00  0.00
H
ATOM    788  HB2 MET A  70      12.102   9.553  10.392  1.00  0.00
H
```

```
ATOM    789  HG3 MET A  70      13.954   8.118  11.481  1.00  0.00
H
ATOM    790  HG2 MET A  70      14.946   9.568  11.453  1.00  0.00
H
ATOM    791  HE1 MET A  70      15.439  10.240   7.699  1.00  0.00
H
ATOM    792  HE2 MET A  70      14.357  11.038   8.865  1.00  0.00
H
ATOM    793  HE3 MET A  70      15.998  10.557   9.359  1.00  0.00
H
ATOM    794  N   LYS A  71      10.032  10.904  12.156  1.00 39.90
N
ANISOU  794  N   LYS A  71     4514   5251   5395   1088   1189   -252
ATOM    795  CA  LYS A  71       8.954  11.896  12.261  1.00 41.54
C
ANISOU  795  CA  LYS A  71     4553   5399   5830   1212   1335   -325
ATOM    796  C   LYS A  71       8.704  12.259  13.732  1.00 47.90
C
ANISOU  796  C   LYS A  71     5547   6078   6573   1148   1682   -392
ATOM    797  O   LYS A  71       8.666  13.447  14.055  1.00 47.43
O
ANISOU  797  O   LYS A  71     5564   5936   6521   1238   1770   -421
ATOM    798  CB  LYS A  71       7.663  11.395  11.570  1.00 45.53
C
ANISOU  798  CB  LYS A  71     4673   5945   6680   1270   1296   -399
ATOM    799  CG  LYS A  71       6.437  12.314  11.718  1.00 59.48
C
ANISOU  799  CG  LYS A  71     6191   7623   8788   1407   1441   -509
ATOM    800  CD  LYS A  71       6.551  13.628  10.941  1.00 66.66
C
ANISOU  800  CD  LYS A  71     7102   8512   9713   1626   1218   -469
ATOM    801  CE  LYS A  71       5.363  14.525  11.198  1.00 78.71
C
ANISOU  801  CE  LYS A  71     8384   9926  11596   1769   1365   -585
ATOM    802  NZ  LYS A  71       5.461  15.802  10.445  1.00 85.73
N1+
ANISOU  802  NZ  LYS A  71     9326  10769  12477   1999   1148   -533
ATOM    803  H   LYS A  71       9.803  10.033  11.698  1.00  0.00
H
ATOM    804  HA  LYS A  71       9.280  12.799  11.745  1.00  0.00
H
ATOM    805  HB3 LYS A  71       7.416  10.402  11.945  1.00  0.00
H
ATOM    806  HB2 LYS A  71       7.862  11.228  10.511  1.00  0.00
H
ATOM    807  HG3 LYS A  71       6.273  12.531  12.774  1.00  0.00
H
ATOM    808  HG2 LYS A  71       5.544  11.780  11.395  1.00  0.00
H
ATOM    809  HD3 LYS A  71       6.620  13.414   9.874  1.00  0.00
H
ATOM    810  HD2 LYS A  71       7.465  14.144  11.233  1.00  0.00
H
ATOM    811  HE3 LYS A  71       5.293  14.738  12.265  1.00  0.00
H
ATOM    812  HE2 LYS A  71       4.448  14.005  10.913  1.00  0.00
H
```

```
ATOM    813  HZ1 LYS A  71       5.650  15.606   9.472  1.00  0.00
H
ATOM    814  HZ2 LYS A  71       4.590  16.307  10.523  1.00  0.00
H
ATOM    815  HZ3 LYS A  71       6.210  16.362  10.827  1.00  0.00
H
ATOM    816  N   ASP A  72       8.593  11.238  14.618  1.00 46.37
N
ANISOU  816  N   ASP A  72     5490   5846   6284    999   1889   -413
ATOM    817  CA  ASP A  72       8.371  11.420  16.063  1.00 48.35
C
ANISOU  817  CA  ASP A  72     6042   5936   6393    934   2255   -471
ATOM    818  C   ASP A  72       9.537  12.156  16.711  1.00 51.29
C
ANISOU  818  C   ASP A  72     6822   6242   6424    947   2141   -449
ATOM    819  O   ASP A  72       9.326  12.913  17.651  1.00 52.79
O
ANISOU  819  O   ASP A  72     7245   6291   6522    970   2379   -515
ATOM    820  CB  ASP A  72       8.119  10.076  16.774  1.00 51.18
C
ANISOU  820  CB  ASP A  72     6547   6235   6665    780   2486   -473
ATOM    821  CG  ASP A  72       6.925   9.284  16.264  1.00 62.27
C
ANISOU  821  CG  ASP A  72     7529   7659   8473    739   2646   -533
ATOM    822  OD1 ASP A  72       5.946   9.911  15.795  1.00 63.94
O
ANISOU  822  OD1 ASP A  72     7364   7858   9073    835   2723   -624
ATOM    823  OD2 ASP A  72       6.957   8.040  16.359  1.00 69.07
O1-
ANISOU  823  OD2 ASP A  72     8428   8524   9294    616   2687   -507
ATOM    824  H   ASP A  72       8.662  10.282  14.299  1.00  0.00
H
ATOM    825  HA  ASP A  72       7.479  12.034  16.188  1.00  0.00
H
ATOM    826  HB3 ASP A  72       8.005  10.250  17.844  1.00  0.00
H
ATOM    827  HB2 ASP A  72       9.015   9.459  16.707  1.00  0.00
H
ATOM    828  N   LYS A  73      10.765  11.941  16.205  1.00 45.04
N
ANISOU  828  N   LYS A  73     6103   5527   5482    933   1783   -379
ATOM    829  CA  LYS A  73      11.952  12.627  16.705  1.00 44.06
C
ANISOU  829  CA  LYS A  73     6282   5321   5138    944   1610   -393
ATOM    830  C   LYS A  73      11.918  14.114  16.318  1.00 48.15
C
ANISOU  830  C   LYS A  73     6695   5805   5796   1064   1602   -422
ATOM    831  O   LYS A  73      12.282  14.947  17.146  1.00 49.44
O
ANISOU  831  O   LYS A  73     7117   5837   5829   1081   1641   -492
ATOM    832  CB  LYS A  73      13.240  11.927  16.241  1.00 44.35
C
ANISOU  832  CB  LYS A  73     6354   5412   5084    888   1267   -338
ATOM    833  CG  LYS A  73      13.508  10.597  16.951  1.00 46.53
C
ANISOU  833  CG  LYS A  73     6882   5655   5144    786   1243   -324
```

```
ATOM    834  CD  LYS A  73      14.808   9.924  16.498  1.00 49.29
C
ANISOU  834  CD  LYS A  73     7224   6038   5467    745    894   -286
ATOM    835  CE  LYS A  73      16.056  10.559  17.080  1.00 58.12
C
ANISOU  835  CE  LYS A  73     8590   7016   6476    764    640   -364
ATOM    836  NZ  LYS A  73      17.275   9.752  16.804  1.00 59.05
N1+
ANISOU  836  NZ  LYS A  73     8679   7127   6631    721    320   -353
ATOM    837  H   LYS A  73      10.892  11.283  15.449  1.00  0.00
H
ATOM    838  HA  LYS A  73      11.926  12.573  17.793  1.00  0.00
H
ATOM    839  HB3 LYS A  73      14.089  12.594  16.391  1.00  0.00
H
ATOM    840  HB2 LYS A  73      13.193  11.759  15.165  1.00  0.00
H
ATOM    841  HG3 LYS A  73      12.671   9.920  16.779  1.00  0.00
H
ATOM    842  HG2 LYS A  73      13.542  10.762  18.028  1.00  0.00
H
ATOM    843  HD3 LYS A  73      14.866   9.944  15.410  1.00  0.00
H
ATOM    844  HD2 LYS A  73      14.781   8.868  16.766  1.00  0.00
H
ATOM    845  HE3 LYS A  73      15.935  10.676  18.157  1.00  0.00
H
ATOM    846  HE2 LYS A  73      16.181  11.559  16.665  1.00  0.00
H
ATOM    847  HZ1 LYS A  73      17.324   9.542  15.817  1.00  0.00
H
ATOM    848  HZ2 LYS A  73      18.095  10.275  17.077  1.00  0.00
H
ATOM    849  HZ3 LYS A  73      17.235   8.891  17.330  1.00  0.00
H
ATOM    850  N   ILE A  74      11.421  14.455  15.101  1.00 44.21
N
ANISOU  850  N   ILE A  74     5856   5398   5545   1159   1546   -375
ATOM    851  CA  ILE A  74      11.280  15.858  14.661  1.00 44.81
C
ANISOU  851  CA  ILE A  74     5852   5418   5758   1297   1554   -385
ATOM    852  C   ILE A  74      10.250  16.561  15.552  1.00 51.69
C
ANISOU  852  C   ILE A  74     6753   6183   6705   1348   1870   -479
ATOM    853  O   ILE A  74      10.552  17.621  16.103  1.00 52.12
O
ANISOU  853  O   ILE A  74     6986   6115   6700   1389   1938   -531
ATOM    854  CB  ILE A  74      10.924  16.030  13.150  1.00 47.26
C
ANISOU  854  CB  ILE A  74     5879   5814   6265   1423   1394   -308
ATOM    855  CG1 ILE A  74      11.990  15.407  12.218  1.00 44.99
C
ANISOU  855  CG1 ILE A  74     5614   5592   5888   1376   1148   -218
ATOM    856  CG2 ILE A  74      10.690  17.524  12.805  1.00 48.90
C
ANISOU  856  CG2 ILE A  74     6062   5923   6596   1590   1429   -310
```

```
ATOM    857  CD1 ILE A  74      11.515  15.229  10.734  1.00 50.05
C
ANISOU  857  CD1 ILE A  74     6061   6314   6640   1492    987   -144
ATOM    858  H   ILE A  74      11.130  13.735  14.455  1.00  0.00
H
ATOM    859  HA  ILE A  74      12.240  16.348  14.826  1.00  0.00
H
ATOM    860  HB  ILE A  74       9.987  15.503  12.973  1.00  0.00
H
ATOM    861 HG13 ILE A  74      12.299  14.441  12.617  1.00  0.00
H
ATOM    862 HG12 ILE A  74      12.891  16.021  12.238  1.00  0.00
H
ATOM    863 HG21 ILE A  74      10.444  17.619  11.747  1.00  0.00
H
ATOM    864 HG22 ILE A  74       9.867  17.912  13.406  1.00  0.00
H
ATOM    865 HG23 ILE A  74      11.595  18.093  13.019  1.00  0.00
H
ATOM    866 HD11 ILE A  74      12.319  14.787  10.145  1.00  0.00
H
ATOM    867 HD12 ILE A  74      10.643  14.575  10.707  1.00  0.00
H
ATOM    868 HD13 ILE A  74      11.252  16.201  10.317  1.00  0.00
H
ATOM    869  N   VAL A  75       9.053  15.949  15.711  1.00 50.44
N
ANISOU  869  N   VAL A  75     6411   6043   6709   1335   2087   -518
ATOM    870  CA  VAL A  75       7.938  16.453  16.535  1.00 53.65
C
ANISOU  870  CA  VAL A  75     6796   6319   7267   1370   2472   -624
ATOM    871  C   VAL A  75       8.381  16.671  18.000  1.00 60.39
C
ANISOU  871  C   VAL A  75     8132   7016   7796   1288   2697   -685
ATOM    872  O   VAL A  75       8.064  17.711  18.585  1.00 61.92
O
ANISOU  872  O   VAL A  75     8440   7074   8014   1355   2908   -763
ATOM    873  CB  VAL A  75       6.677  15.538  16.414  1.00 59.43
C
ANISOU  873  CB  VAL A  75     7202   7069   8309   1338   2682   -676
ATOM    874  CG1 VAL A  75       5.563  15.962  17.377  1.00 62.79
C
ANISOU  874  CG1 VAL A  75     7610   7310   8937   1349   3175   -805
ATOM    875  CG2 VAL A  75       6.153  15.505  14.979  1.00 58.89
C
ANISOU  875  CG2 VAL A  75     6683   7120   8574   1465   2391   -654
ATOM    876  H   VAL A  75       8.868  15.073  15.244  1.00  0.00
H
ATOM    877  HA  VAL A  75       7.663  17.429  16.136  1.00  0.00
H
ATOM    878  HB  VAL A  75       6.977  14.524  16.680  1.00  0.00
H
ATOM    879 HG11 VAL A  75       4.707  15.298  17.258  1.00  0.00
H
ATOM    880 HG12 VAL A  75       5.927  15.904  18.403  1.00  0.00
H
```

```
ATOM    881 HG13 VAL A  75       5.262  16.986  17.156  1.00  0.00
H
ATOM    882 HG21 VAL A  75       5.276  14.860  14.927  1.00  0.00
H
ATOM    883 HG22 VAL A  75       5.881  16.514  14.668  1.00  0.00
H
ATOM    884 HG23 VAL A  75       6.928  15.117  14.318  1.00  0.00
H
ATOM    885  N   ALA A  76       9.141  15.713  18.570  1.00 56.83
N
ANISOU  885  N   ALA A  76     7997   6568   7029   1161   2615   -655
ATOM    886  CA  ALA A  76       9.634  15.797  19.952  1.00 58.65
C
ANISOU  886  CA  ALA A  76     8776   6626   6882   1107   2736   -717
ATOM    887  C   ALA A  76      10.883  16.688  20.110  1.00 62.45
C
ANISOU  887  C   ALA A  76     9491   7060   7177   1143   2406   -746
ATOM    888  O   ALA A  76      11.366  16.846  21.233  1.00 64.61
O
ANISOU  888  O   ALA A  76    10244   7177   7130   1121   2403   -822
ATOM    889  CB  ALA A  76       9.903  14.402  20.505  1.00 59.55
C
ANISOU  889  CB  ALA A  76     9167   6723   6738    988   2755   -678
ATOM    890  H   ALA A  76       9.396  14.890  18.042  1.00  0.00
H
ATOM    891  HA  ALA A  76       8.840  16.239  20.555  1.00  0.00
H
ATOM    892  HB1 ALA A  76      10.268  14.481  21.529  1.00  0.00
H
ATOM    893  HB2 ALA A  76       8.981  13.821  20.492  1.00  0.00
H
ATOM    894  HB3 ALA A  76      10.653  13.905  19.890  1.00  0.00
H
ATOM    895  N   ASN A  77      11.381  17.290  18.996  1.00 56.44
N
ANISOU  895  N   ASN A  77     8417   6398   6630   1205   2141   -700
ATOM    896  CA  ASN A  77      12.559  18.172  18.942  1.00 55.32
C
ANISOU  896  CA  ASN A  77     8387   6189   6444   1227   1864   -738
ATOM    897  C   ASN A  77      13.839  17.444  19.419  1.00 58.09
C
ANISOU  897  C   ASN A  77     9010   6502   6560   1137   1544   -766
ATOM    898  O   ASN A  77      14.638  17.992  20.182  1.00 58.08
O
ANISOU  898  O   ASN A  77     9306   6346   6415   1137   1387   -880
ATOM    899  CB  ASN A  77      12.304  19.509  19.689  1.00 59.18
C
ANISOU  899  CB  ASN A  77     9067   6503   6915   1300   2049   -853
ATOM    900  CG  ASN A  77      13.159  20.665  19.221  1.00 82.10
C
ANISOU  900  CG  ASN A  77    11909   9337   9949   1345   1846   -886
ATOM    901  OD1 ASN A  77      13.138  21.063  18.050  1.00 73.23
O
ANISOU  901  OD1 ASN A  77    10471   8282   9072   1409   1801   -800
ATOM    902  ND2 ASN A  77      13.897  21.264  20.144  1.00 77.97
N
ANISOU  902  ND2 ASN A  77    11719   8643   9264   1322   1731  -1023
```

```
ATOM    903  H   ASN A  77      10.934  17.147  18.102  1.00  0.00
H
ATOM    904  HA  ASN A  77      12.714  18.424  17.893  1.00  0.00
H
ATOM    905  HB3 ASN A  77      12.454  19.359  20.758  1.00  0.00
H
ATOM    906  HB2 ASN A  77      11.252  19.778  19.598  1.00  0.00
H
ATOM    907 HD22 ASN A  77      14.488  22.043  19.891  1.00  0.00
H
ATOM    908 HD21 ASN A  77      13.868  20.942  21.101  1.00  0.00
H
ATOM    909  N   GLU A  78      14.018  16.197  18.936  1.00 53.42
N
ANISOU  909  N   GLU A  78     8302   6038   5959   1071   1419   -675
ATOM    910  CA  GLU A  78      15.136  15.311  19.259  1.00 52.91
C
ANISOU  910  CA  GLU A  78     8434   5945   5725   1000   1102   -688
ATOM    911  C   GLU A  78      16.341  15.458  18.332  1.00 55.14
C
ANISOU  911  C   GLU A  78     8479   6250   6223    986    777   -683
ATOM    912  O   GLU A  78      17.385  14.851  18.590  1.00 55.35
O
ANISOU  912  O   GLU A  78     8628   6212   6190    942    475   -732
ATOM    913  CB  GLU A  78      14.660  13.850  19.310  1.00 54.02
C
ANISOU  913  CB  GLU A  78     8599   6175   5752    934   1183   -601
ATOM    914  CG  GLU A  78      14.551  13.311  20.725  1.00 67.27
C
ANISOU  914  CG  GLU A  78    10807   7696   7057    911   1284   -651
ATOM    915  CD  GLU A  78      13.349  12.428  20.990  1.00 88.38
C
ANISOU  915  CD  GLU A  78    13519  10382   9679    865   1702   -591
ATOM    916  OE1 GLU A  78      13.186  11.406  20.284  1.00 82.00
O
ANISOU  916  OE1 GLU A  78    12436   9710   9011    807   1701   -501
ATOM    917  OE2 GLU A  78      12.584  12.743  21.929  1.00 85.61
O1-
ANISOU  917  OE2 GLU A  78    13488   9876   9162    881   2054   -649
ATOM    918  H   GLU A  78      13.346  15.803  18.293  1.00  0.00
H
ATOM    919  HA  GLU A  78      15.471  15.573  20.263  1.00  0.00
H
ATOM    920  HB3 GLU A  78      15.345  13.226  18.737  1.00  0.00
H
ATOM    921  HB2 GLU A  78      13.692  13.767  18.815  1.00  0.00
H
ATOM    922  HG3 GLU A  78      14.549  14.144  21.429  1.00  0.00
H
ATOM    923  HG2 GLU A  78      15.461  12.765  20.974  1.00  0.00
H
ATOM    924  N   TYR A  79      16.210  16.237  17.247  1.00 49.49
N
ANISOU  924  N   TYR A  79     7443   5592   5770   1033    848   -629
ATOM    925  CA  TYR A  79      17.344  16.425  16.349  1.00 47.39
C
ANISOU  925  CA  TYR A  79     6984   5295   5727   1013    647   -624
```

```
ATOM    926  C   TYR A  79      17.997  17.768  16.618  1.00 52.11
C
ANISOU  926  C   TYR A  79     7640   5704   6454   1040    610   -746
ATOM    927  O   TYR A  79      17.329  18.803  16.532  1.00 52.34
O
ANISOU  927  O   TYR A  79     7659   5702   6524   1116    818   -744
ATOM    928  CB  TYR A  79      16.942  16.284  14.873  1.00 46.17
C
ANISOU  928  CB  TYR A  79     6528   5272   5741   1051    745   -480
ATOM    929  CG  TYR A  79      16.568  14.883  14.446  1.00 45.49
C
ANISOU  929  CG  TYR A  79     6345   5352   5589   1008    717   -384
ATOM    930  CD1 TYR A  79      17.522  13.867  14.399  1.00 46.11
C
ANISOU  930  CD1 TYR A  79     6435   5434   5651    920    514   -386
ATOM    931  CD2 TYR A  79      15.273  14.581  14.036  1.00 45.65
C
ANISOU  931  CD2 TYR A  79     6227   5503   5613   1061    876   -309
ATOM    932  CE1 TYR A  79      17.186  12.578  13.985  1.00 44.42
C
ANISOU  932  CE1 TYR A  79     6137   5357   5383    878    498   -302
ATOM    933  CE2 TYR A  79      14.933  13.304  13.595  1.00 45.20
C
ANISOU  933  CE2 TYR A  79     6060   5579   5535   1014    841   -243
ATOM    934  CZ  TYR A  79      15.888  12.300  13.590  1.00 49.12
C
ANISOU  934  CZ  TYR A  79     6608   6083   5972    918    667   -233
ATOM    935  OH  TYR A  79      15.546  11.040  13.167  1.00 45.61
O
ANISOU  935  OH  TYR A  79     6066   5756   5509    868    644   -174
ATOM    936  H   TYR A  79      15.328  16.691  17.055  1.00  0.00
H
ATOM    937  HA  TYR A  79      18.076  15.648  16.570  1.00  0.00
H
ATOM    938  HB3 TYR A  79      17.752  16.649  14.242  1.00  0.00
H
ATOM    939  HB2 TYR A  79      16.114  16.960  14.659  1.00  0.00
H
ATOM    940  HD1 TYR A  79      18.543  14.071  14.686  1.00  0.00
H
ATOM    941  HD2 TYR A  79      14.509  15.344  14.056  1.00  0.00
H
ATOM    942  HE1 TYR A  79      17.937  11.802  13.973  1.00  0.00
H
ATOM    943  HE2 TYR A  79      13.928  13.099  13.258  1.00  0.00
H
ATOM    944  HH  TYR A  79      16.185  10.406  13.500  1.00  0.00
H
ATOM    945  N   LYS A  80      19.283  17.752  16.997  1.00 49.03
N
ANISOU  945  N   LYS A  80     7296   5167   6166    983    334   -873
ATOM    946  CA  LYS A  80      20.005  18.995  17.272  1.00 50.67
C
ANISOU  946  CA  LYS A  80     7524   5162   6568    989    271  -1027
ATOM    947  C   LYS A  80      20.674  19.534  16.006  1.00 52.13
C
ANISOU  947  C   LYS A  80     7404   5276   7128    971    359   -985
```

```
ATOM    948  O   LYS A  80      21.014  20.716  15.949  1.00 53.19
O
ANISOU  948  O   LYS A  80     7508   5229   7472    981    430  -1075
ATOM    949  CB  LYS A  80      20.978  18.853  18.468  1.00 56.49
C
ANISOU  949  CB  LYS A  80     8492   5717   7253    955   -101  -1242
ATOM    950  CG  LYS A  80      20.339  18.639  19.835  0.00 76.56
C
ANISOU  950  CG  LYS A  80    10845   8176  10068    886   -423  -1320
ATOM    951  CD  LYS A  80      21.401  18.490  20.924  0.00 91.68
C
ANISOU  951  CD  LYS A  80    13015   9889  11930    893   -875  -1558
ATOM    952  CE  LYS A  80      20.803  18.299  22.299  0.00101.94
C
ANISOU  952  CE  LYS A  80    14057  11061  13616    839  -1223  -1677
ATOM    953  NZ  LYS A  80      21.851  18.132  23.339  0.00111.96
N1+
ANISOU  953  NZ  LYS A  80    15601  12128  14810    883  -1760  -1917
ATOM    954  H   LYS A  80      19.769  16.872  17.097  1.00  0.00
H
ATOM    955  HA  LYS A  80      19.257  19.731  17.566  1.00  0.00
H
ATOM    956  HB3 LYS A  80      21.628  19.727  18.508  1.00  0.00
H
ATOM    957  HB2 LYS A  80      21.678  18.042  18.268  1.00  0.00
H
ATOM    958  HG3 LYS A  80      19.718  17.744  19.809  1.00  0.00
H
ATOM    959  HG2 LYS A  80      19.694  19.486  20.071  1.00  0.00
H
ATOM    960  HD3 LYS A  80      22.043  19.371  20.927  1.00  0.00
H
ATOM    961  HD2 LYS A  80      22.045  17.643  20.688  1.00  0.00
H
ATOM    962  HE3 LYS A  80      20.154  17.423  22.294  1.00  0.00
H
ATOM    963  HE2 LYS A  80      20.180  19.159  22.546  1.00  0.00
H
ATOM    964  HZ1 LYS A  80      22.163  17.171  23.352  1.00  0.00
H
ATOM    965  HZ2 LYS A  80      22.633  18.736  23.130  1.00  0.00
H
ATOM    966  HZ3 LYS A  80      21.471  18.375  24.243  1.00  0.00
H
ATOM    967  N   SER A  81      20.835  18.669  14.984  1.00 45.38
N
ANISOU  967  N   SER A  81     6360   4536   6345    944    391   -848
ATOM    968  CA  SER A  81      21.450  19.037  13.714  1.00 44.00
C
ANISOU  968  CA  SER A  81     5977   4270   6472    930    538   -787
ATOM    969  C   SER A  81      20.892  18.233  12.540  1.00 46.05
C
ANISOU  969  C   SER A  81     6150   4712   6633    962    664   -583
ATOM    970  O   SER A  81      20.292  17.170  12.745  1.00 43.12
O
ANISOU  970  O   SER A  81     5810   4535   6041    959    578   -521
```

```
ATOM    971  CB  SER A  81      22.963  18.844  13.789  1.00 47.39
C
ANISOU  971  CB  SER A  81     6264   4494   7248    824    348   -948
ATOM    972  OG  SER A  81      23.314  17.471  13.814  1.00 49.74
O
ANISOU  972  OG  SER A  81     6515   4890   7493    772    136   -937
ATOM    973  H   SER A  81      20.523  17.713  15.077  1.00  0.00
H
ATOM    974  HA  SER A  81      21.249  20.093  13.531  1.00  0.00
H
ATOM    975  HB3 SER A  81      23.347  19.333  14.684  1.00  0.00
H
ATOM    976  HB2 SER A  81      23.433  19.323  12.930  1.00  0.00
H
ATOM    977  HG  SER A  81      24.056  17.341  14.409  1.00  0.00
H
ATOM    978  N   VAL A  82      21.138  18.727  11.304  1.00 43.68
N
ANISOU  978  N   VAL A  82     5780   4318   6499    993    875   -491
ATOM    979  CA  VAL A  82      20.766  18.047  10.056  1.00 42.82
C
ANISOU  979  CA  VAL A  82     5649   4327   6293   1040    973   -316
ATOM    980  C   VAL A  82      21.622  16.771   9.928  1.00 45.70
C
ANISOU  980  C   VAL A  82     5906   4716   6743    925    835   -345
ATOM    981  O   VAL A  82      21.112  15.752   9.464  1.00 44.52
O
ANISOU  981  O   VAL A  82     5751   4746   6417    938    791   -242
ATOM    982  CB  VAL A  82      20.872  18.978   8.816  1.00 48.13
C
ANISOU  982  CB  VAL A  82     6393   4828   7066   1124   1249   -212
ATOM    983  CG1 VAL A  82      20.615  18.219   7.513  1.00 47.46
C
ANISOU  983  CG1 VAL A  82     6369   4829   6836   1186   1311    -49
ATOM    984  CG2 VAL A  82      19.914  20.158   8.937  1.00 48.98
C
ANISOU  984  CG2 VAL A  82     6613   4911   7084   1263   1351   -176
ATOM    985  H   VAL A  82      21.605  19.616  11.199  1.00  0.00
H
ATOM    986  HA  VAL A  82      19.725  17.737  10.147  1.00  0.00
H
ATOM    987  HB  VAL A  82      21.887  19.373   8.779  1.00  0.00
H
ATOM    988 HG11 VAL A  82      20.698  18.905   6.670  1.00  0.00
H
ATOM    989 HG12 VAL A  82      21.350  17.421   7.406  1.00  0.00
H
ATOM    990 HG13 VAL A  82      19.613  17.789   7.534  1.00  0.00
H
ATOM    991 HG21 VAL A  82      20.007  20.794   8.057  1.00  0.00
H
ATOM    992 HG22 VAL A  82      18.891  19.790   9.011  1.00  0.00
H
ATOM    993 HG23 VAL A  82      20.158  20.735   9.829  1.00  0.00
H
ATOM    994  N   THR A  83      22.901  16.822  10.389  1.00 43.04
N
```

```
ANISOU  994  N   THR A  83     5463   4184   6708    817    741   -508
ATOM    995  CA  THR A  83      23.851  15.703  10.412  1.00 43.08
C
ANISOU  995  CA  THR A  83     5330   4159   6879    718    575   -577
ATOM    996  C   THR A  83      23.245  14.493  11.145  1.00 45.35
C
ANISOU  996  C   THR A  83     5690   4681   6859    717    320   -549
ATOM    997  O   THR A  83      23.273  13.389  10.598  1.00 43.14
O
ANISOU  997  O   THR A  83     5358   4501   6534    689    296   -474
ATOM    998  CB  THR A  83      25.210  16.165  10.978  1.00 52.24
C
ANISOU  998  CB  THR A  83     6333   5034   8481    630    453   -808
ATOM    999  OG1 THR A  83      25.764  17.114  10.070  1.00 55.51
O
ANISOU  999  OG1 THR A  83     6676   5205   9212    614    789   -814
ATOM   1000  CG2 THR A  83      26.205  15.014  11.179  1.00 51.13
C
ANISOU 1000  CG2 THR A  83     6014   4829   8583    547    230   -911
ATOM   1001  H   THR A  83      23.271  17.687  10.757  1.00  0.00
H
ATOM   1002  HA  THR A  83      24.019  15.402   9.378  1.00  0.00
H
ATOM   1003  HB  THR A  83      25.042  16.655  11.937  1.00  0.00
H
ATOM   1004  HG1 THR A  83      25.337  17.964  10.195  1.00  0.00
H
ATOM   1005 HG21 THR A  83      27.140  15.407  11.579  1.00  0.00
H
ATOM   1006 HG22 THR A  83      25.787  14.290  11.878  1.00  0.00
H
ATOM   1007 HG23 THR A  83      26.396  14.527  10.223  1.00  0.00
H
ATOM   1008  N   GLU A  84      22.663  14.719  12.352  1.00 41.69
N
ANISOU 1008  N   GLU A  84     5382   4283   6176    748    179   -606
ATOM   1009  CA  GLU A  84      21.987  13.692  13.161  1.00 40.47
C
ANISOU 1009  CA  GLU A  84     5372   4301   5705    749     20   -577
ATOM   1010  C   GLU A  84      20.813  13.075  12.393  1.00 42.17
C
ANISOU 1010  C   GLU A  84     5567   4740   5716    785    192   -399
ATOM   1011  O   GLU A  84      20.641  11.854  12.415  1.00 40.94
O
ANISOU 1011  O   GLU A  84     5414   4696   5445    747    107   -351
ATOM   1012  CB  GLU A  84      21.484  14.277  14.488  1.00 42.95
C
ANISOU 1012  CB  GLU A  84     5923   4587   5809    788    -47   -667
ATOM   1013  CG  GLU A  84      22.562  14.382  15.553  1.00 54.05
C
ANISOU 1013  CG  GLU A  84     7434   5788   7315    762   -368   -872
ATOM   1014  CD  GLU A  84      22.136  14.879  16.924  1.00 68.11
C
ANISOU 1014  CD  GLU A  84     9562   7516   8802    810   -465   -969
ATOM   1015  OE1 GLU A  84      20.927  15.121  17.142  1.00 51.20
O
ANISOU 1015  OE1 GLU A  84     7555   5488   6410    853   -210   -880
```

```
ATOM   1016  OE2 GLU A  84       23.027  15.014  17.791  1.00 69.55
O1-
ANISOU 1016  OE2 GLU A  84      9892   7516   9019    813   -803  -1152
ATOM   1017  H   GLU A  84       22.676  15.646  12.753  1.00  0.00
H
ATOM   1018  HA  GLU A  84       22.705  12.903  13.384  1.00  0.00
H
ATOM   1019  HB3 GLU A  84       20.662  13.667  14.864  1.00  0.00
H
ATOM   1020  HB2 GLU A  84       21.055  15.263  14.309  1.00  0.00
H
ATOM   1021  HG3 GLU A  84       23.373  15.008  15.181  1.00  0.00
H
ATOM   1022  HG2 GLU A  84       23.056  13.416  15.660  1.00  0.00
H
ATOM   1023  N   PHE A  85       20.014  13.926  11.715  1.00 37.68
N
ANISOU 1023  N   PHE A  85      4977   4212   5127    869    406   -316
ATOM   1024  CA  PHE A  85       18.885  13.516  10.881  1.00 35.72
C
ANISOU 1024  CA  PHE A  85      4684   4139   4748    934    513   -181
ATOM   1025  C   PHE A  85       19.388  12.644   9.716  1.00 37.10
C
ANISOU 1025  C   PHE A  85      4775   4339   4983    904    500   -106
ATOM   1026  O   PHE A  85       18.851  11.565   9.496  1.00 35.60
O
ANISOU 1026  O   PHE A  85      4551   4297   4678    888    445    -53
ATOM   1027  CB  PHE A  85       18.116  14.750  10.381  1.00 37.50
C
ANISOU 1027  CB  PHE A  85      4924   4341   4985   1064    677   -133
ATOM   1028  CG  PHE A  85       16.948  14.449   9.473  1.00 38.29
C
ANISOU 1028  CG  PHE A  85      4967   4582   4999   1170    709    -27
ATOM   1029  CD1 PHE A  85       15.721  14.062   9.998  1.00 40.93
C
ANISOU 1029  CD1 PHE A  85      5242   5050   5259   1196    711    -41
ATOM   1030  CD2 PHE A  85       17.078  14.547   8.091  1.00 40.25
C
ANISOU 1030  CD2 PHE A  85      5239   4794   5260   1250    739     68
ATOM   1031  CE1 PHE A  85       14.642  13.782   9.158  1.00 42.92
C
ANISOU 1031  CE1 PHE A  85      5387   5407   5516   1301    684     14
ATOM   1032  CE2 PHE A  85       16.000  14.255   7.248  1.00 43.62
C
ANISOU 1032  CE2 PHE A  85      5641   5329   5603   1374    680    138
ATOM   1033  CZ  PHE A  85       14.791  13.867   7.787  1.00 42.34
C
ANISOU 1033  CZ  PHE A  85      5347   5308   5432   1399    624     98
ATOM   1034  H   PHE A  85       20.179  14.921  11.765  1.00  0.00
H
ATOM   1035  HA  PHE A  85       18.210  12.916  11.491  1.00  0.00
H
ATOM   1036  HB3 PHE A  85       18.806  15.422   9.871  1.00  0.00
H
ATOM   1037  HB2 PHE A  85       17.768  15.329  11.236  1.00  0.00
H
```

```
ATOM   1038  HD1 PHE A  85      15.594  13.975  11.067  1.00  0.00
H
ATOM   1039  HD2 PHE A  85      18.019  14.851   7.657  1.00  0.00
H
ATOM   1040  HE1 PHE A  85      13.689  13.498   9.579  1.00  0.00
H
ATOM   1041  HE2 PHE A  85      16.115  14.334   6.177  1.00  0.00
H
ATOM   1042  HZ  PHE A  85      13.961  13.630   7.138  1.00  0.00
H
ATOM   1043  N   LYS A  86      20.436  13.100   9.000  1.00 34.79
N
ANISOU 1043  N   LYS A  86     4457   3872   4891    887    582   -116
ATOM   1044  CA  LYS A  86      21.064  12.361   7.889  1.00 34.66
C
ANISOU 1044  CA  LYS A  86     4401   3815   4954    855    640    -60
ATOM   1045  C   LYS A  86      21.569  10.991   8.344  1.00 37.91
C
ANISOU 1045  C   LYS A  86     4730   4288   5387    749    459   -106
ATOM   1046  O   LYS A  86      21.419  10.014   7.606  1.00 35.64
O
ANISOU 1046  O   LYS A  86     4430   4084   5026    739    463    -36
ATOM   1047  CB  LYS A  86      22.227  13.166   7.289  1.00 37.71
C
ANISOU 1047  CB  LYS A  86     4773   3930   5625    829    834   -100
ATOM   1048  CG  LYS A  86      21.784  14.355   6.442  1.00 39.53
C
ANISOU 1048  CG  LYS A  86     5158   4058   5802    950   1074     -6
ATOM   1049  CD  LYS A  86      22.956  15.293   6.106  1.00 44.88
C
ANISOU 1049  CD  LYS A  86     5828   4414   6812    902   1327    -71
ATOM   1050  CE  LYS A  86      23.987  14.693   5.173  1.00 53.74
C
ANISOU 1050  CE  LYS A  86     6923   5360   8136    825   1524    -68
ATOM   1051  NZ  LYS A  86      25.022  15.696   4.798  1.00 63.83
N1+
ANISOU 1051  NZ  LYS A  86     8189   6277   9788    774   1860   -136
ATOM   1052  H   LYS A  86      20.839  14.001   9.213  1.00  0.00
H
ATOM   1053  HA  LYS A  86      20.315  12.209   7.112  1.00  0.00
H
ATOM   1054  HB3 LYS A  86      22.851  12.506   6.687  1.00  0.00
H
ATOM   1055  HB2 LYS A  86      22.876  13.516   8.092  1.00  0.00
H
ATOM   1056  HG3 LYS A  86      21.014  14.913   6.975  1.00  0.00
H
ATOM   1057  HG2 LYS A  86      21.332  13.994   5.518  1.00  0.00
H
ATOM   1058  HD3 LYS A  86      23.443  15.607   7.029  1.00  0.00
H
ATOM   1059  HD2 LYS A  86      22.568  16.214   5.671  1.00  0.00
H
ATOM   1060  HE3 LYS A  86      23.493  14.328   4.273  1.00  0.00
H
ATOM   1061  HE2 LYS A  86      24.465  13.843   5.659  1.00  0.00
H
```

```
ATOM   1062  HZ1 LYS A  86      24.651  16.316   4.093  1.00  0.00
H
ATOM   1063  HZ2 LYS A  86      25.283  16.232   5.613  1.00  0.00
H
ATOM   1064  HZ3 LYS A  86      25.835  15.221   4.433  1.00  0.00
H
ATOM   1065  N   ALA A  87      22.150  10.916   9.566  1.00 36.22
N
ANISOU 1065  N   ALA A  87     4494   4012   5256    687    275   -230
ATOM   1066  CA  ALA A  87      22.655   9.664  10.140  1.00 36.18
C
ANISOU 1066  CA  ALA A  87     4460   4026   5260    614     53   -281
ATOM   1067  C   ALA A  87      21.526   8.637  10.356  1.00 37.72
C
ANISOU 1067  C   ALA A  87     4738   4441   5152    618     13   -189
ATOM   1068  O   ALA A  87      21.720   7.456  10.058  1.00 34.81
O
ANISOU 1068  O   ALA A  87     4330   4121   4777    572    -52   -158
ATOM   1069  CB  ALA A  87      23.378   9.936  11.451  1.00 38.69
C
ANISOU 1069  CB  ALA A  87     4821   4203   5674    592   -189   -440
ATOM   1070  H   ALA A  87      22.252  11.749  10.129  1.00  0.00
H
ATOM   1071  HA  ALA A  87      23.373   9.236   9.440  1.00  0.00
H
ATOM   1072  HB1 ALA A  87      23.747   8.997  11.865  1.00  0.00
H
ATOM   1073  HB2 ALA A  87      24.217  10.608  11.271  1.00  0.00
H
ATOM   1074  HB3 ALA A  87      22.689  10.398  12.158  1.00  0.00
H
ATOM   1075  N   ASP A  88      20.348   9.082  10.859  1.00 34.36
N
ANISOU 1075  N   ASP A  88     4409   4122   4523    669     83   -159
ATOM   1076  CA  ASP A  88      19.201   8.179  11.063  1.00 33.62
C
ANISOU 1076  CA  ASP A  88     4352   4196   4227    661    111    -96
ATOM   1077  C   ASP A  88      18.671   7.689   9.714  1.00 36.02
C
ANISOU 1077  C   ASP A  88     4531   4611   4543    685    187     -8
ATOM   1078  O   ASP A  88      18.346   6.510   9.561  1.00 33.81
O
ANISOU 1078  O   ASP A  88     4220   4422   4206    636    148     22
ATOM   1079  CB  ASP A  88      18.085   8.875  11.860  1.00 36.47
C
ANISOU 1079  CB  ASP A  88     4805   4594   4457    709    229   -112
ATOM   1080  CG  ASP A  88      18.196   8.771  13.368  1.00 43.87
C
ANISOU 1080  CG  ASP A  88     5980   5450   5240    678    165   -185
ATOM   1081  OD1 ASP A  88      19.186   8.186  13.856  1.00 44.01
O
ANISOU 1081  OD1 ASP A  88     6096   5374   5251    636    -42   -232
ATOM   1082  OD2 ASP A  88      17.278   9.250  14.060  1.00 47.07
O1-
ANISOU 1082  OD2 ASP A  88     6496   5860   5529    708    319   -202
ATOM   1083  H   ASP A  88      20.239  10.056  11.105  1.00  0.00
H
```

```
ATOM   1084  HA  ASP A  88      19.542   7.314  11.633  1.00  0.00
H
ATOM   1085  HB3 ASP A  88      17.118   8.484  11.542  1.00  0.00
H
ATOM   1086  HB2 ASP A  88      18.040   9.926  11.574  1.00  0.00
H
ATOM   1087  N   PHE A  89      18.617   8.597   8.729  1.00 33.11
N
ANISOU 1087  N   PHE A  89     4132   4209   4239    769    282     28
ATOM   1088  CA  PHE A  89      18.176   8.294   7.372  1.00 32.54
C
ANISOU 1088  CA  PHE A  89     4032   4196   4133    831    314    103
ATOM   1089  C   PHE A  89      19.084   7.232   6.729  1.00 33.12
C
ANISOU 1089  C   PHE A  89     4094   4235   4255    755    282    116
ATOM   1090  O   PHE A  89      18.582   6.247   6.172  1.00 31.92
O
ANISOU 1090  O   PHE A  89     3918   4186   4024    747    229    145
ATOM   1091  CB  PHE A  89      18.158   9.579   6.531  1.00 35.25
C
ANISOU 1091  CB  PHE A  89     4454   4440   4499    956    427    144
ATOM   1092  CG  PHE A  89      17.878   9.328   5.075  1.00 37.34
C
ANISOU 1092  CG  PHE A  89     4806   4704   4677   1047    437    220
ATOM   1093  CD1 PHE A  89      16.631   8.874   4.660  1.00 41.45
C
ANISOU 1093  CD1 PHE A  89     5294   5364   5092   1130    308    234
ATOM   1094  CD2 PHE A  89      18.863   9.528   4.118  1.00 39.27
C
ANISOU 1094  CD2 PHE A  89     5186   4775   4960   1052    581    258
ATOM   1095  CE1 PHE A  89      16.375   8.642   3.314  1.00 43.04
C
ANISOU 1095  CE1 PHE A  89     5638   5542   5174   1238    252    285
ATOM   1096  CE2 PHE A  89      18.608   9.284   2.774  1.00 43.11
C
ANISOU 1096  CE2 PHE A  89     5856   5227   5296   1152    601    329
ATOM   1097  CZ  PHE A  89      17.371   8.837   2.381  1.00 41.72
C
ANISOU 1097  CZ  PHE A  89     5688   5201   4962   1254    402    342
ATOM   1098  H   PHE A  89      18.891   9.553   8.907  1.00  0.00
H
ATOM   1099  HA  PHE A  89      17.162   7.898   7.419  1.00  0.00
H
ATOM   1100  HB3 PHE A  89      19.115  10.092   6.633  1.00  0.00
H
ATOM   1101  HB2 PHE A  89      17.409  10.263   6.931  1.00  0.00
H
ATOM   1102  HD1 PHE A  89      15.851   8.699   5.386  1.00  0.00
H
ATOM   1103  HD2 PHE A  89      19.841   9.877   4.415  1.00  0.00
H
ATOM   1104  HE1 PHE A  89      15.397   8.309   3.000  1.00  0.00
H
ATOM   1105  HE2 PHE A  89      19.383   9.446   2.039  1.00  0.00
H
ATOM   1106  HZ  PHE A  89      17.179   8.638   1.337  1.00  0.00
H
```

```
ATOM   1107  N   LYS A  90      20.417   7.438   6.813  1.00 29.30
N
ANISOU 1107  N   LYS A  90     3603   3586   3945    699    316     73
ATOM   1108  CA  LYS A  90      21.418   6.513   6.284  1.00 29.39
C
ANISOU 1108  CA  LYS A  90     3570   3514   4082    625    320     61
ATOM   1109  C   LYS A  90      21.298   5.153   6.978  1.00 33.49
C
ANISOU 1109  C   LYS A  90     4044   4139   4541    546    145     43
ATOM   1110  O   LYS A  90      21.371   4.128   6.305  1.00 31.89
O
ANISOU 1110  O   LYS A  90     3826   3969   4321    515    145     72
ATOM   1111  CB  LYS A  90      22.836   7.075   6.449  1.00 32.15
C
ANISOU 1111  CB  LYS A  90     3847   3627   4740    577    383    -27
ATOM   1112  CG  LYS A  90      23.912   6.179   5.807  1.00 34.44
C
ANISOU 1112  CG  LYS A  90     4054   3785   5246    508    445    -57
ATOM   1113  CD  LYS A  90      25.292   6.818   5.847  1.00 39.01
C
ANISOU 1113  CD  LYS A  90     4494   4085   6244    459    541   -178
ATOM   1114  CE  LYS A  90      26.341   5.936   5.214  1.00 44.68
C
ANISOU 1114  CE  LYS A  90     5092   4639   7247    392    649   -227
ATOM   1115  NZ  LYS A  90      26.598   4.710   6.026  1.00 43.53
N1+
ANISOU 1115  NZ  LYS A  90     4841   4567   7133    347    344   -279
ATOM   1116  H   LYS A  90      20.772   8.270   7.262  1.00  0.00
H
ATOM   1117  HA  LYS A  90      21.227   6.373   5.220  1.00  0.00
H
ATOM   1118  HB3 LYS A  90      23.055   7.200   7.509  1.00  0.00
H
ATOM   1119  HB2 LYS A  90      22.884   8.071   6.009  1.00  0.00
H
ATOM   1120  HG3 LYS A  90      23.639   5.970   4.773  1.00  0.00
H
ATOM   1121  HG2 LYS A  90      23.941   5.220   6.325  1.00  0.00
H
ATOM   1122  HD3 LYS A  90      25.567   7.023   6.882  1.00  0.00
H
ATOM   1123  HD2 LYS A  90      25.263   7.776   5.328  1.00  0.00
H
ATOM   1124  HE3 LYS A  90      27.268   6.498   5.103  1.00  0.00
H
ATOM   1125  HE2 LYS A  90      26.017   5.647   4.214  1.00  0.00
H
ATOM   1126  HZ1 LYS A  90      27.401   4.223   5.656  1.00  0.00
H
ATOM   1127  HZ2 LYS A  90      26.777   4.972   6.985  1.00  0.00
H
ATOM   1128  HZ3 LYS A  90      25.790   4.105   5.988  1.00  0.00
H
ATOM   1129  N   LEU A  91      21.078   5.151   8.309  1.00 31.02
N
ANISOU 1129  N   LEU A  91     3760   3857   4170    523     16     -1
```

```
ATOM   1130  CA  LEU A  91      20.904   3.929   9.097  1.00 30.24
C
ANISOU 1130  CA  LEU A  91     3704   3818   3970    461   -119     -5
ATOM   1131  C   LEU A  91      19.716   3.105   8.589  1.00 32.65
C
ANISOU 1131  C   LEU A  91     3988   4287   4128    452    -55     61
ATOM   1132  O   LEU A  91      19.849   1.893   8.410  1.00 31.22
O
ANISOU 1132  O   LEU A  91     3791   4126   3944    393   -105     75
ATOM   1133  CB  LEU A  91      20.763   4.266  10.592  1.00 30.99
C
ANISOU 1133  CB  LEU A  91     3945   3874   3954    465   -218    -56
ATOM   1134  CG  LEU A  91      20.362   3.122  11.559  1.00 34.73
C
ANISOU 1134  CG  LEU A  91     4581   4372   4243    421   -298    -41
ATOM   1135  CD1 LEU A  91      21.446   2.034  11.632  1.00 34.70
C
ANISOU 1135  CD1 LEU A  91     4578   4269   4337    385   -496    -63
ATOM   1136  CD2 LEU A  91      20.068   3.681  12.941  1.00 37.31
C
ANISOU 1136  CD2 LEU A  91     5153   4630   4394    451   -330    -86
ATOM   1137  H   LEU A  91      21.026   6.024   8.814  1.00  0.00
H
ATOM   1138  HA  LEU A  91      21.803   3.325   8.976  1.00  0.00
H
ATOM   1139  HB3 LEU A  91      20.063   5.093  10.707  1.00  0.00
H
ATOM   1140  HB2 LEU A  91      21.688   4.723  10.944  1.00  0.00
H
ATOM   1141  HG  LEU A  91      19.449   2.664  11.179  1.00  0.00
H
ATOM   1142 HD11 LEU A  91      21.129   1.250  12.319  1.00  0.00
H
ATOM   1143 HD12 LEU A  91      21.600   1.607  10.641  1.00  0.00
H
ATOM   1144 HD13 LEU A  91      22.378   2.473  11.987  1.00  0.00
H
ATOM   1145 HD21 LEU A  91      19.788   2.868  13.610  1.00  0.00
H
ATOM   1146 HD22 LEU A  91      20.956   4.180  13.328  1.00  0.00
H
ATOM   1147 HD23 LEU A  91      19.249   4.397  12.877  1.00  0.00
H
ATOM   1148  N   MET A  92      18.579   3.768   8.326  1.00 29.04
N
ANISOU 1148  N   MET A  92     3510   3925   3598    515     40     84
ATOM   1149  CA  MET A  92      17.371   3.123   7.801  1.00 28.72
C
ANISOU 1149  CA  MET A  92     3392   4014   3505    521     63    103
ATOM   1150  C   MET A  92      17.677   2.411   6.466  1.00 31.69
C
ANISOU 1150  C   MET A  92     3740   4402   3900    524     22    126
ATOM   1151  O   MET A  92      17.341   1.237   6.317  1.00 30.48
O
ANISOU 1151  O   MET A  92     3542   4304   3735    461    -20    119
ATOM   1152  CB  MET A  92      16.253   4.169   7.638  1.00 31.27
C
```

```
ANISOU 1152  CB  MET A  92      3662   4391   3827    625    126     95
ATOM   1153  CG  MET A  92       14.941   3.598   7.132  1.00 34.87
C
ANISOU 1153  CG  MET A  92      3977   4950   4321    646    102     70
ATOM   1154  SD  MET A  92       13.801   4.909   6.645  1.00 39.77
S
ANISOU 1154  SD  MET A  92      4509   5594   5009    820     92     45
ATOM   1155  CE  MET A  92       14.537   5.416   5.089  1.00 36.35
C
ANISOU 1155  CE  MET A  92      4231   5103   4476    946    -15    109
ATOM   1156  H   MET A  92       18.526   4.763   8.489  1.00  0.00
H
ATOM   1157  HA  MET A  92       17.040   2.375   8.521  1.00  0.00
H
ATOM   1158  HB3 MET A  92       16.589   4.955   6.962  1.00  0.00
H
ATOM   1159  HB2 MET A  92       16.085   4.670   8.591  1.00  0.00
H
ATOM   1160  HG3 MET A  92       14.484   2.991   7.914  1.00  0.00
H
ATOM   1161  HG2 MET A  92       15.133   2.949   6.278  1.00  0.00
H
ATOM   1162  HE1 MET A  92       13.949   6.225   4.655  1.00  0.00
H
ATOM   1163  HE2 MET A  92       15.556   5.762   5.264  1.00  0.00
H
ATOM   1164  HE3 MET A  92       14.554   4.570   4.402  1.00  0.00
H
ATOM   1165  N   CYS A  93       18.353   3.109   5.532  1.00 28.54
N
ANISOU 1165  N   CYS A  93      3401   3917   3526    592     66    150
ATOM   1166  CA  CYS A  93       18.736   2.568   4.216  1.00 28.93
C
ANISOU 1166  CA  CYS A  93      3514   3925   3552    610     83    172
ATOM   1167  C   CYS A  93       19.769   1.454   4.343  1.00 32.06
C
ANISOU 1167  C   CYS A  93      3881   4256   4044    498     78    154
ATOM   1168  O   CYS A  93       19.616   0.416   3.691  1.00 30.83
O
ANISOU 1168  O   CYS A  93      3735   4134   3843    471     45    154
ATOM   1169  CB  CYS A  93       19.231   3.683   3.298  1.00 29.46
C
ANISOU 1169  CB  CYS A  93      3725   3859   3610    712    215    208
ATOM   1170  SG  CYS A  93       17.993   4.955   2.950  1.00 34.01
S
ANISOU 1170  SG  CYS A  93      4379   4485   4060    888    179    238
ATOM   1171  H   CYS A  93       18.628   4.063   5.715  1.00  0.00
H
ATOM   1172  HA  CYS A  93       17.842   2.139   3.762  1.00  0.00
H
ATOM   1173  HB3 CYS A  93       19.579   3.251   2.360  1.00  0.00
H
ATOM   1174  HB2 CYS A  93       20.113   4.148   3.738  1.00  0.00
H
ATOM   1175  HG  CYS A  93       17.609   5.529   4.094  1.00  0.00
H
```

```
ATOM   1176  N   ASP A  94      20.810   1.651   5.197  1.00 28.64
 N
ANISOU 1176  N   ASP A  94     3406   3713   3764    444     76    122
ATOM   1177  CA  ASP A  94      21.841   0.644   5.445  1.00 28.81
 C
ANISOU 1177  CA  ASP A  94     3373   3640   3935    361     22     87
ATOM   1178  C   ASP A  94      21.232  -0.643   6.012  1.00 31.76
 C
ANISOU 1178  C   ASP A  94     3744   4120   4205    298    -99     97
ATOM   1179  O   ASP A  94      21.659  -1.722   5.613  1.00 30.42
 O
ANISOU 1179  O   ASP A  94     3554   3912   4092    249   -115     91
ATOM   1180  CB  ASP A  94      22.941   1.170   6.389  1.00 31.29
 C
ANISOU 1180  CB  ASP A  94     3629   3799   4463    344    -50     16
ATOM   1181  CG  ASP A  94      23.882   2.196   5.781  1.00 38.33
 C
ANISOU 1181  CG  ASP A  94     4470   4506   5586    368    115    -24
ATOM   1182  OD1 ASP A  94      23.926   2.305   4.526  1.00 38.45
 O
ANISOU 1182  OD1 ASP A  94     4546   4476   5588    394    328     18
ATOM   1183  OD2 ASP A  94      24.592   2.873   6.552  1.00 39.31
 O1-
ANISOU 1183  OD2 ASP A  94     4523   4503   5911    364     41   -107
ATOM   1184  H   ASP A  94      20.896   2.526   5.695  1.00  0.00
 H
ATOM   1185  HA  ASP A  94      22.308   0.401   4.490  1.00  0.00
 H
ATOM   1186  HB3 ASP A  94      23.522   0.330   6.769  1.00  0.00
 H
ATOM   1187  HB2 ASP A  94      22.479   1.590   7.282  1.00  0.00
 H
ATOM   1188  N   ASN A  95      20.218  -0.533   6.898  1.00 28.75
 N
ANISOU 1188  N   ASN A  95     3394   3843   3688    296   -137    108
ATOM   1189  CA  ASN A  95      19.547  -1.703   7.495  1.00 28.50
 C
ANISOU 1189  CA  ASN A  95     3387   3874   3569    226   -172    118
ATOM   1190  C   ASN A  95      18.859  -2.533   6.421  1.00 30.85
 C
ANISOU 1190  C   ASN A  95     3614   4257   3852    203   -143    120
ATOM   1191  O   ASN A  95      19.038  -3.752   6.380  1.00 28.97
 O
ANISOU 1191  O   ASN A  95     3377   3998   3632    132   -172    119
ATOM   1192  CB  ASN A  95      18.536  -1.267   8.559  1.00 27.93
 C
ANISOU 1192  CB  ASN A  95     3375   3852   3386    229   -117    119
ATOM   1193  CG  ASN A  95      19.157  -0.765   9.838  1.00 34.90
 C
ANISOU 1193  CG  ASN A  95     4417   4626   4217    244   -185    104
ATOM   1194  OD1 ASN A  95      20.310  -1.059  10.158  1.00 28.27
 O
ANISOU 1194  OD1 ASN A  95     3632   3667   3443    243   -336     83
ATOM   1195  ND2 ASN A  95      18.370  -0.069  10.642  1.00 29.75
 N
ANISOU 1195  ND2 ASN A  95     3853   3992   3460    265    -91     98
```

```
ATOM   1196  H   ASN A  95      19.889   0.380   7.178  1.00  0.00
H
ATOM   1197  HA  ASN A  95      20.304  -2.324   7.975  1.00  0.00
H
ATOM   1198  HB3 ASN A  95      17.868  -2.098   8.784  1.00  0.00
H
ATOM   1199  HB2 ASN A  95      17.887  -0.495   8.146  1.00  0.00
H
ATOM   1200 HD22 ASN A  95      18.731   0.293  11.513  1.00  0.00
H
ATOM   1201 HD21 ASN A  95      17.408   0.100  10.385  1.00  0.00
H
ATOM   1202  N   ALA A  96      18.118  -1.860   5.521  1.00 26.93
N
ANISOU 1202  N   ALA A  96     3075   3835   3322    278   -116    113
ATOM   1203  CA  ALA A  96      17.422  -2.523   4.425  1.00 27.50
C
ANISOU 1203  CA  ALA A  96     3108   3970   3372    288   -161     84
ATOM   1204  C   ALA A  96      18.404  -3.185   3.462  1.00 30.12
C
ANISOU 1204  C   ALA A  96     3520   4219   3706    276   -154     89
ATOM   1205  O   ALA A  96      18.094  -4.242   2.931  1.00 29.61
O
ANISOU 1205  O   ALA A  96     3444   4176   3631    233   -202     56
ATOM   1206  CB  ALA A  96      16.531  -1.536   3.693  1.00 29.00
C
ANISOU 1206  CB  ALA A  96     3287   4216   3515    415   -203     66
ATOM   1207  H   ALA A  96      18.028  -0.856   5.590  1.00  0.00
H
ATOM   1208  HA  ALA A  96      16.788  -3.301   4.849  1.00  0.00
H
ATOM   1209  HB1 ALA A  96      16.018  -2.045   2.877  1.00  0.00
H
ATOM   1210  HB2 ALA A  96      15.795  -1.127   4.386  1.00  0.00
H
ATOM   1211  HB3 ALA A  96      17.139  -0.726   3.290  1.00  0.00
H
ATOM   1212  N   MET A  97      19.601  -2.589   3.267  1.00 26.92
N
ANISOU 1212  N   MET A  97     3183   3691   3354    304    -69    114
ATOM   1213  CA  MET A  97      20.617  -3.168   2.374  1.00 26.68
C
ANISOU 1213  CA  MET A  97     3221   3534   3382    287     16    107
ATOM   1214  C   MET A  97      21.410  -4.297   3.046  1.00 30.02
C
ANISOU 1214  C   MET A  97     3561   3890   3955    186    -21     89
ATOM   1215  O   MET A  97      21.990  -5.133   2.353  1.00 28.98
O
ANISOU 1215  O   MET A  97     3455   3673   3882    156     36     68
ATOM   1216  CB  MET A  97      21.532  -2.083   1.783  1.00 29.95
C
ANISOU 1216  CB  MET A  97     3724   3793   3862    352    192    122
ATOM   1217  CG  MET A  97      20.785  -1.106   0.871  1.00 34.57
C
ANISOU 1217  CG  MET A  97     4485   4400   4249    480    231    154
ATOM   1218  SD  MET A  97      21.865   0.099   0.076  1.00 40.87
S
```

```
ANISOU 1218  SD  MET A  97      5470   4952   5107    550    534    185
ATOM   1219  CE  MET A  97       22.137   1.218   1.401  1.00 38.16
C
ANISOU 1219  CE  MET A  97      4923   4593   4983    517    522    170
ATOM   1220  H   MET A  97       19.817  -1.723   3.740  1.00  0.00
H
ATOM   1221  HA  MET A  97       20.081  -3.616   1.537  1.00  0.00
H
ATOM   1222  HB3 MET A  97       22.339  -2.555   1.224  1.00  0.00
H
ATOM   1223  HB2 MET A  97       22.008  -1.530   2.593  1.00  0.00
H
ATOM   1224  HG3 MET A  97       20.022  -0.583   1.448  1.00  0.00
H
ATOM   1225  HG2 MET A  97       20.243  -1.665   0.108  1.00  0.00
H
ATOM   1226  HE1 MET A  97       22.790   2.025   1.068  1.00  0.00
H
ATOM   1227  HE2 MET A  97       21.184   1.634   1.728  1.00  0.00
H
ATOM   1228  HE3 MET A  97       22.607   0.691   2.232  1.00  0.00
H
ATOM   1229  N   THR A  98       21.438  -4.325   4.385  1.00 26.29
N
ANISOU 1229  N   THR A  98      3031   3431   3528    148   -120     94
ATOM   1230  CA  THR A  98       22.106  -5.390   5.141  1.00 26.59
C
ANISOU 1230  CA  THR A  98      3049   3384   3670     86   -214     83
ATOM   1231  C   THR A  98       21.176  -6.621   5.234  1.00 30.26
C
ANISOU 1231  C   THR A  98      3538   3935   4026     16   -241     97
ATOM   1232  O   THR A  98       21.630  -7.754   5.042  1.00 29.16
O
ANISOU 1232  O   THR A  98      3398   3722   3959    -30   -259     87
ATOM   1233  CB  THR A  98       22.541  -4.873   6.532  1.00 32.40
C
ANISOU 1233  CB  THR A  98      3810   4053   4448    105   -342     77
ATOM   1234  OG1 THR A  98       23.505  -3.840   6.354  1.00 34.00
O
ANISOU 1234  OG1 THR A  98      3942   4141   4835    153   -317     32
ATOM   1235  CG2 THR A  98       23.141  -5.973   7.417  1.00 33.33
C
ANISOU 1235  CG2 THR A  98      3981   4056   4625     79   -506     70
ATOM   1236  H   THR A  98       20.987  -3.593   4.915  1.00  0.00
H
ATOM   1237  HA  THR A  98       23.002  -5.683   4.593  1.00  0.00
H
ATOM   1238  HB  THR A  98       21.670  -4.455   7.037  1.00  0.00
H
ATOM   1239  HG1 THR A  98       23.061  -3.029   6.096  1.00  0.00
H
ATOM   1240 HG21 THR A  98       23.427  -5.550   8.380  1.00  0.00
H
ATOM   1241 HG22 THR A  98       22.402  -6.759   7.572  1.00  0.00
H
ATOM   1242 HG23 THR A  98       24.021  -6.393   6.929  1.00  0.00
H
```

```
ATOM   1243  N   TYR A  99      19.890  -6.403   5.561  1.00 27.09
N
ANISOU 1243  N   TYR A  99     3137   3659   3498      5   -224    106
ATOM   1244  CA  TYR A  99      18.965  -7.528   5.739  1.00 27.77
C
ANISOU 1244  CA  TYR A  99     3209   3789   3553    -79   -205     96
ATOM   1245  C   TYR A  99      18.535  -8.185   4.417  1.00 31.81
C
ANISOU 1245  C   TYR A  99     3658   4346   4082    -95   -210     41
ATOM   1246  O   TYR A  99      18.454  -9.418   4.348  1.00 30.88
O
ANISOU 1246  O   TYR A  99     3537   4190   4005   -175   -209     21
ATOM   1247  CB  TYR A  99      17.746  -7.120   6.592  1.00 29.22
C
ANISOU 1247  CB  TYR A  99     3379   4041   3683    -97   -131     95
ATOM   1248  CG  TYR A  99      16.704  -8.208   6.729  1.00 31.50
C
ANISOU 1248  CG  TYR A  99     3610   4338   4022   -200    -47     60
ATOM   1249  CD1 TYR A  99      16.810  -9.183   7.718  1.00 34.36
C
ANISOU 1249  CD1 TYR A  99     4111   4588   4356   -282     31    101
ATOM   1250  CD2 TYR A  99      15.616  -8.270   5.865  1.00 32.50
C
ANISOU 1250  CD2 TYR A  99     3556   4551   4243   -208    -55    -29
ATOM   1251  CE1 TYR A  99      15.857 -10.200   7.836  1.00 35.17
C
ANISOU 1251  CE1 TYR A  99     4160   4658   4547   -394    169     63
ATOM   1252  CE2 TYR A  99      14.679  -9.294   5.953  1.00 34.67
C
ANISOU 1252  CE2 TYR A  99     3723   4800   4650   -317     26    -96
ATOM   1253  CZ  TYR A  99      14.796 -10.252   6.945  1.00 40.75
C
ANISOU 1253  CZ  TYR A  99     4618   5452   5413   -423    175    -47
ATOM   1254  OH  TYR A  99      13.846 -11.243   7.017  1.00 38.82
O
ANISOU 1254  OH  TYR A  99     4263   5148   5339   -546    313   -121
ATOM   1255  H   TYR A  99      19.554  -5.459   5.686  1.00  0.00
H
ATOM   1256  HA  TYR A  99      19.503  -8.288   6.306  1.00  0.00
H
ATOM   1257  HB3 TYR A  99      17.285  -6.230   6.162  1.00  0.00
H
ATOM   1258  HB2 TYR A  99      18.084  -6.816   7.583  1.00  0.00
H
ATOM   1259  HD1 TYR A  99      17.639  -9.162   8.410  1.00  0.00
H
ATOM   1260  HD2 TYR A  99      15.485  -7.513   5.106  1.00  0.00
H
ATOM   1261  HE1 TYR A  99      15.950 -10.939   8.618  1.00  0.00
H
ATOM   1262  HE2 TYR A  99      13.862  -9.341   5.248  1.00  0.00
H
ATOM   1263  HH  TYR A  99      13.982 -11.760   7.814  1.00  0.00
H
ATOM   1264  N   ASN A 100      18.205  -7.368   3.408  1.00 27.74
N
ANISOU 1264  N   ASN A 100     3133   3891   3515     -9   -231     12
```

```
ATOM   1265  CA  ASN A 100      17.700  -7.811   2.106  1.00 27.72
C
ANISOU 1265  CA  ASN A 100     3146   3914   3471     15   -296    -58
ATOM   1266  C   ASN A 100      18.795  -7.971   1.070  1.00 30.05
C
ANISOU 1266  C   ASN A 100     3592   4102   3724     53   -241    -51
ATOM   1267  O   ASN A 100      19.711  -7.154   1.029  1.00 28.99
O
ANISOU 1267  O   ASN A 100     3523   3891   3601    107   -144     -4
ATOM   1268  CB  ASN A 100      16.659  -6.827   1.591  1.00 30.73
C
ANISOU 1268  CB  ASN A 100     3501   4384   3792    122   -389   -100
ATOM   1269  CG  ASN A 100      15.541  -6.572   2.569  1.00 39.65
C
ANISOU 1269  CG  ASN A 100     4449   5590   5027     88   -387   -127
ATOM   1270  OD1 ASN A 100      14.656  -7.400   2.735  1.00 31.96
O
ANISOU 1270  OD1 ASN A 100     3332   4634   4178      7   -408   -207
ATOM   1271  ND2 ASN A 100      15.581  -5.438   3.269  1.00 28.95
N
ANISOU 1271  ND2 ASN A 100     3095   4252   3652    141   -323    -73
ATOM   1272  H   ASN A 100      18.298  -6.368   3.517  1.00  0.00
H
ATOM   1273  HA  ASN A 100      17.216  -8.778   2.241  1.00  0.00
H
ATOM   1274  HB3 ASN A 100      16.243  -7.198   0.654  1.00  0.00
H
ATOM   1275  HB2 ASN A 100      17.145  -5.883   1.343  1.00  0.00
H
ATOM   1276 HD22 ASN A 100      14.851  -5.229   3.935  1.00  0.00
H
ATOM   1277 HD21 ASN A 100      16.342  -4.787   3.133  1.00  0.00
H
ATOM   1278  N   ARG A 101      18.673  -8.994   0.191  1.00 27.68
N
ANISOU 1278  N   ARG A 101     3352   3771   3395     25   -277   -115
ATOM   1279  CA  ARG A 101      19.653  -9.254  -0.871  1.00 27.48
C
ANISOU 1279  CA  ARG A 101     3512   3611   3319     57   -169   -123
ATOM   1280  C   ARG A 101      19.583  -8.211  -1.982  1.00 31.53
C
ANISOU 1280  C   ARG A 101     4259   4087   3635    202   -143   -122
ATOM   1281  O   ARG A 101      18.542  -7.569  -2.107  1.00 31.09
O
ANISOU 1281  O   ARG A 101     4205   4133   3475    283   -304   -143
ATOM   1282  CB  ARG A 101      19.539 -10.689  -1.402  1.00 27.45
C
ANISOU 1282  CB  ARG A 101     3536   3568   3326    -17   -208   -199
ATOM   1283  CG  ARG A 101      20.231 -11.664  -0.467  1.00 31.09
C
ANISOU 1283  CG  ARG A 101     3869   3963   3980   -131   -144   -168
ATOM   1284  CD  ARG A 101      20.126 -13.089  -0.950  1.00 33.18
C
ANISOU 1284  CD  ARG A 101     4161   4172   4274   -209   -161   -241
ATOM   1285  NE  ARG A 101      20.898 -13.990  -0.093  1.00 33.84
N
ANISOU 1285  NE  ARG A 101     4162   4159   4538   -290   -105   -199
```

```
ATOM   1286  CZ  ARG A 101      21.265 -15.221  -0.434  1.00 39.06
C
ANISOU 1286  CZ  ARG A 101     4852   4713   5274   -350    -67   -242
ATOM   1287  NH1 ARG A 101      20.927 -15.717  -1.618  1.00 30.71
N
ANISOU 1287  NH1 ARG A 101     3913   3637   4116   -351    -71   -339
ATOM   1288  NH2 ARG A 101      21.985 -15.961   0.400  1.00 32.66
N1+
ANISOU 1288  NH2 ARG A 101     3984   3795   4632   -394    -49   -196
ATOM   1289  H   ARG A 101      17.881  -9.618   0.249  1.00  0.00
H
ATOM   1290  HA  ARG A 101      20.640  -9.164  -0.417  1.00  0.00
H
ATOM   1291  HB3 ARG A 101      19.995 -10.748  -2.390  1.00  0.00
H
ATOM   1292  HB2 ARG A 101      18.487 -10.959  -1.495  1.00  0.00
H
ATOM   1293  HG3 ARG A 101      19.793 -11.585   0.528  1.00  0.00
H
ATOM   1294  HG2 ARG A 101      21.282 -11.390  -0.371  1.00  0.00
H
ATOM   1295  HD3 ARG A 101      20.498 -13.154  -1.973  1.00  0.00
H
ATOM   1296  HD2 ARG A 101      19.080 -13.396  -0.950  1.00  0.00
H
ATOM   1297  HE  ARG A 101      21.152 -13.620   0.812  1.00  0.00
H
ATOM   1298 HH12 ARG A 101      21.208 -16.653  -1.872  1.00  0.00
H
ATOM   1299 HH11 ARG A 101      20.388 -15.158  -2.264  1.00  0.00
H
ATOM   1300 HH22 ARG A 101      22.261 -16.896   0.136  1.00  0.00
H
ATOM   1301 HH21 ARG A 101      22.257 -15.589   1.299  1.00  0.00
H
ATOM   1302  N   PRO A 102      20.660  -8.004  -2.792  1.00 29.85
N
ANISOU 1302  N   PRO A 102     4261   3698   3382    245     79   -100
ATOM   1303  CA  PRO A 102      20.636  -6.914  -3.788  1.00 30.92
C
ANISOU 1303  CA  PRO A 102     4705   3751   3293    394    162    -75
ATOM   1304  C   PRO A 102      19.560  -6.968  -4.872  1.00 36.10
C
ANISOU 1304  C   PRO A 102     5631   4440   3646    520    -67   -140
ATOM   1305  O   PRO A 102      19.207  -5.911  -5.402  1.00 36.59
O
ANISOU 1305  O   PRO A 102     5920   4474   3509    671   -107   -108
ATOM   1306  CB  PRO A 102      22.037  -6.968  -4.404  1.00 33.21
C
ANISOU 1306  CB  PRO A 102     5172   3794   3654    381    522    -59
ATOM   1307  CG  PRO A 102      22.885  -7.583  -3.333  1.00 35.13
C
ANISOU 1307  CG  PRO A 102     5075   4019   4255    244    582    -62
ATOM   1308  CD  PRO A 102      21.994  -8.643  -2.757  1.00 30.55
C
ANISOU 1308  CD  PRO A 102     4319   3618   3671    168    303    -98
```

```
ATOM   1309  HA  PRO A 102      20.535  -5.969  -3.255  1.00  0.00
H
ATOM   1310  HB3 PRO A 102      22.384  -5.954  -4.601  1.00  0.00
H
ATOM   1311  HB2 PRO A 102      22.023  -7.624  -5.274  1.00  0.00
H
ATOM   1312  HG3 PRO A 102      23.096  -6.836  -2.568  1.00  0.00
H
ATOM   1313  HG2 PRO A 102      23.756  -8.054  -3.789  1.00  0.00
H
ATOM   1314  HD2 PRO A 102      21.994  -9.510  -3.417  1.00  0.00
H
ATOM   1315  HD3 PRO A 102      22.277  -8.827  -1.721  1.00  0.00
H
ATOM   1316  N   ASP A 103      19.029  -8.163  -5.193  1.00 32.86
N
ANISOU 1316  N   ASP A 103     5207   4070   3210    471   -246   -240
ATOM   1317  CA  ASP A 103      18.009  -8.302  -6.239  1.00 34.36
C
ANISOU 1317  CA  ASP A 103     5645   4266   3144    600   -544   -346
ATOM   1318  C   ASP A 103      16.569  -8.151  -5.714  1.00 39.45
C
ANISOU 1318  C   ASP A 103     5991   5096   3904    621   -911   -427
ATOM   1319  O   ASP A 103      15.620  -8.480  -6.428  1.00 42.09
O
ANISOU 1319  O   ASP A 103     6411   5439   4143    707  -1241   -564
ATOM   1320  CB  ASP A 103      18.202  -9.622  -7.020  1.00 37.20
C
ANISOU 1320  CB  ASP A 103     6177   4529   3428    549   -555   -448
ATOM   1321  CG  ASP A 103      18.134 -10.900  -6.192  1.00 42.34
C
ANISOU 1321  CG  ASP A 103     6450   5265   4374    353   -583   -502
ATOM   1322  OD1 ASP A 103      18.128 -10.803  -4.932  1.00 39.32
O
ANISOU 1322  OD1 ASP A 103     5706   4990   4243    247   -533   -437
ATOM   1323  OD2 ASP A 103      18.146 -11.997  -6.795  1.00 44.64
O1-
ANISOU 1323  OD2 ASP A 103     6852   5486   4624    310   -632   -604
ATOM   1324  H   ASP A 103      19.331  -8.996  -4.709  1.00  0.00
H
ATOM   1325  HA  ASP A 103      18.172  -7.490  -6.948  1.00  0.00
H
ATOM   1326  HB3 ASP A 103      19.152  -9.589  -7.553  1.00  0.00
H
ATOM   1327  HB2 ASP A 103      17.468  -9.675  -7.824  1.00  0.00
H
ATOM   1328  N   THR A 104      16.407  -7.615  -4.494  1.00 34.66
N
ANISOU 1328  N   THR A 104     5043   4608   3518    552   -853   -361
ATOM   1329  CA  THR A 104      15.098  -7.422  -3.856  1.00 34.45
C
ANISOU 1329  CA  THR A 104     4694   4723   3673    552  -1094   -437
ATOM   1330  C   THR A 104      14.571  -5.993  -3.980  1.00 39.14
C
ANISOU 1330  C   THR A 104     5354   5340   4177    732  -1207   -405
ATOM   1331  O   THR A 104      15.353  -5.044  -4.091  1.00 36.52
O
```

```
ANISOU 1331  O   THR A 104     5237   4939   3701    805  -1011   -281
ATOM   1332  CB  THR A 104      15.140  -7.847  -2.372  1.00 36.59
C
ANISOU 1332  CB  THR A 104     4599   5078   4227    361   -929   -396
ATOM   1333  OG1 THR A 104      15.932  -6.909  -1.634  1.00 33.42
O
ANISOU 1333  OG1 THR A 104     4208   4671   3819    364   -712   -257
ATOM   1334  CG2 THR A 104      15.640  -9.286  -2.164  1.00 33.53
C
ANISOU 1334  CG2 THR A 104     4154   4648   3939    195   -829   -418
ATOM   1335  H   THR A 104      17.212  -7.319  -3.962  1.00  0.00
H
ATOM   1336  HA  THR A 104      14.389  -8.076  -4.363  1.00  0.00
H
ATOM   1337  HB  THR A 104      14.122  -7.794  -1.986  1.00  0.00
H
ATOM   1338  HG1 THR A 104      16.863  -7.100  -1.768  1.00  0.00
H
ATOM   1339 HG21 THR A 104      15.644  -9.519  -1.099  1.00  0.00
H
ATOM   1340 HG22 THR A 104      14.980  -9.979  -2.685  1.00  0.00
H
ATOM   1341 HG23 THR A 104      16.651  -9.381  -2.560  1.00  0.00
H
ATOM   1342  N   VAL A 105      13.236  -5.843  -3.903  1.00 38.76
N
ANISOU 1342  N   VAL A 105     5083   5367   4275    797  -1509   -530
ATOM   1343  CA  VAL A 105      12.538  -4.553  -3.961  1.00 39.94
C
ANISOU 1343  CA  VAL A 105     5231   5536   4410    981  -1674   -529
ATOM   1344  C   VAL A 105      13.032  -3.619  -2.834  1.00 40.98
C
ANISOU 1344  C   VAL A 105     5236   5714   4621    925  -1369   -381
ATOM   1345  O   VAL A 105      13.307  -2.451  -3.090  1.00 40.18
O
ANISOU 1345  O   VAL A 105     5351   5560   4356   1070  -1324   -290
ATOM   1346  CB  VAL A 105      10.997  -4.769  -3.944  1.00 46.75
C
ANISOU 1346  CB  VAL A 105     5748   6451   5563   1025  -2040   -734
ATOM   1347  CG1 VAL A 105      10.238  -3.497  -3.568  1.00 48.03
C
ANISOU 1347  CG1 VAL A 105     5776   6638   5834   1183  -2158   -738
ATOM   1348  CG2 VAL A 105      10.507  -5.301  -5.291  1.00 49.70
C
ANISOU 1348  CG2 VAL A 105     6331   6742   5811   1153  -2450   -905
ATOM   1349  H   VAL A 105      12.642  -6.653  -3.798  1.00  0.00
H
ATOM   1350  HA  VAL A 105      12.793  -4.084  -4.911  1.00  0.00
H
ATOM   1351  HB  VAL A 105      10.776  -5.523  -3.189  1.00  0.00
H
ATOM   1352 HG11 VAL A 105       9.167  -3.698  -3.570  1.00  0.00
H
ATOM   1353 HG12 VAL A 105      10.544  -3.171  -2.574  1.00  0.00
H
ATOM   1354 HG13 VAL A 105      10.461  -2.713  -4.292  1.00  0.00
H
```

```
ATOM   1355 HG21 VAL A 105       9.427  -5.444  -5.255  1.00  0.00
H
ATOM   1356 HG22 VAL A 105      10.752  -4.585  -6.076  1.00  0.00
H
ATOM   1357 HG23 VAL A 105      10.992  -6.254  -5.504  1.00  0.00
H
ATOM   1358  N   TYR A 106      13.197  -4.156  -1.611  1.00 35.84
N
ANISOU 1358  N   TYR A 106     4291   5132   4194    722  -1160   -358
ATOM   1359  CA  TYR A 106      13.620  -3.380  -0.443  1.00 34.02
C
ANISOU 1359  CA  TYR A 106     3960   4932   4034    665   -916   -247
ATOM   1360  C   TYR A 106      15.037  -2.841  -0.559  1.00 36.30
C
ANISOU 1360  C   TYR A 106     4505   5134   4155    676   -697   -111
ATOM   1361  O   TYR A 106      15.257  -1.671  -0.240  1.00 35.63
O
ANISOU 1361  O   TYR A 106     4460   5031   4047    745   -604    -42
ATOM   1362  CB  TYR A 106      13.426  -4.172   0.858  1.00 34.09
C
ANISOU 1362  CB  TYR A 106     3697   4991   4265    468   -768   -263
ATOM   1363  CG  TYR A 106      12.054  -4.795   0.970  1.00 37.00
C
ANISOU 1363  CG  TYR A 106     3776   5395   4886    424   -898   -417
ATOM   1364  CD1 TYR A 106      10.915  -4.006   1.089  1.00 40.59
C
ANISOU 1364  CD1 TYR A 106     4027   5877   5519    519   -997   -501
ATOM   1365  CD2 TYR A 106      11.890  -6.177   0.919  1.00 37.88
C
ANISOU 1365  CD2 TYR A 106     3792   5488   5114    287   -912   -499
ATOM   1366  CE1 TYR A 106       9.643  -4.573   1.152  1.00 43.73
C
ANISOU 1366  CE1 TYR A 106     4094   6268   6253    474  -1101   -680
ATOM   1367  CE2 TYR A 106      10.624  -6.756   0.988  1.00 40.85
C
ANISOU 1367  CE2 TYR A 106     3863   5860   5800    231  -1006   -670
ATOM   1368  CZ  TYR A 106       9.504  -5.950   1.107  1.00 49.10
C
ANISOU 1368  CZ  TYR A 106     4669   6918   7068    322  -1097   -769
ATOM   1369  OH  TYR A 106       8.257  -6.514   1.207  1.00 51.67
O
ANISOU 1369  OH  TYR A 106     4628   7202   7803    257  -1165   -969
ATOM   1370  H   TYR A 106      13.027  -5.141  -1.465  1.00  0.00
H
ATOM   1371  HA  TYR A 106      12.959  -2.515  -0.385  1.00  0.00
H
ATOM   1372  HB3 TYR A 106      13.596  -3.515   1.711  1.00  0.00
H
ATOM   1373  HB2 TYR A 106      14.184  -4.952   0.923  1.00  0.00
H
ATOM   1374  HD1 TYR A 106      11.005  -2.931   1.134  1.00  0.00
H
ATOM   1375  HD2 TYR A 106      12.753  -6.820   0.824  1.00  0.00
H
ATOM   1376  HE1 TYR A 106       8.773  -3.939   1.235  1.00  0.00
H
```

```
ATOM   1377  HE2 TYR A 106      10.519  -7.830   0.949  1.00  0.00
H
ATOM   1378  HH  TYR A 106       8.343  -7.449   1.407  1.00  0.00
H
ATOM   1379  N   TYR A 107      15.994  -3.676  -1.020  1.00 31.14
N
ANISOU 1379  N   TYR A 107     4000   4402   3431    607   -598    -91
ATOM   1380  CA  TYR A 107      17.387  -3.250  -1.201  1.00 29.19
C
ANISOU 1380  CA  TYR A 107     3944   4024   3124    605   -355      3
ATOM   1381  C   TYR A 107      17.452  -2.153  -2.285  1.00 33.55
C
ANISOU 1381  C   TYR A 107     4822   4467   3460    789   -328     44
ATOM   1382  O   TYR A 107      18.067  -1.114  -2.062  1.00 32.15
O
ANISOU 1382  O   TYR A 107     4707   4210   3300    822   -143    118
ATOM   1383  CB  TYR A 107      18.269  -4.449  -1.593  1.00 29.40
C
ANISOU 1383  CB  TYR A 107     4040   3962   3169    504   -251    -13
ATOM   1384  CG  TYR A 107      19.743  -4.140  -1.764  1.00 29.99
C
ANISOU 1384  CG  TYR A 107     4237   3861   3298    484     32     47
ATOM   1385  CD1 TYR A 107      20.208  -3.452  -2.883  1.00 32.99
C
ANISOU 1385  CD1 TYR A 107     4944   4076   3516    597    202     78
ATOM   1386  CD2 TYR A 107      20.684  -4.617  -0.856  1.00 29.03
C
ANISOU 1386  CD2 TYR A 107     3920   3695   3413    358    133     56
ATOM   1387  CE1 TYR A 107      21.569  -3.199  -3.065  1.00 34.43
C
ANISOU 1387  CE1 TYR A 107     5199   4049   3834    561    531    108
ATOM   1388  CE2 TYR A 107      22.048  -4.391  -1.040  1.00 29.33
C
ANISOU 1388  CE2 TYR A 107     3997   3538   3610    338    377     69
ATOM   1389  CZ  TYR A 107      22.484  -3.667  -2.137  1.00 37.40
C
ANISOU 1389  CZ  TYR A 107     5288   4392   4531    426    608     90
ATOM   1390  OH  TYR A 107      23.828  -3.455  -2.331  1.00 39.08
O
ANISOU 1390  OH  TYR A 107     5501   4367   4980    390    916     81
ATOM   1391  H   TYR A 107      15.761  -4.631  -1.254  1.00  0.00
H
ATOM   1392  HA  TYR A 107      17.753  -2.837  -0.261  1.00  0.00
H
ATOM   1393  HB3 TYR A 107      17.885  -4.894  -2.511  1.00  0.00
H
ATOM   1394  HB2 TYR A 107      18.150  -5.241  -0.853  1.00  0.00
H
ATOM   1395  HD1 TYR A 107      19.511  -3.103  -3.631  1.00  0.00
H
ATOM   1396  HD2 TYR A 107      20.363  -5.174   0.012  1.00  0.00
H
ATOM   1397  HE1 TYR A 107      21.904  -2.641  -3.927  1.00  0.00
H
ATOM   1398  HE2 TYR A 107      22.761  -4.780  -0.328  1.00  0.00
H
```

```
ATOM   1399  HH  TYR A 107      24.321  -3.839  -1.603  1.00  0.00
H
ATOM   1400  N   LYS A 108      16.825  -2.398  -3.453  1.00 33.16
N
ANISOU 1400  N   LYS A 108     5015   4385   3199    917   -520    -12
ATOM   1401  CA  LYS A 108      16.812  -1.456  -4.576  1.00 35.05
C
ANISOU 1401  CA  LYS A 108     5684   4482   3153   1126   -526     31
ATOM   1402  C   LYS A 108      16.199  -0.115  -4.179  1.00 39.89
C
ANISOU 1402  C   LYS A 108     6240   5135   3783   1252   -605     73
ATOM   1403  O   LYS A 108      16.771   0.929  -4.497  1.00 39.29
O
ANISOU 1403  O   LYS A 108     6428   4912   3588   1345   -396    169
ATOM   1404  CB  LYS A 108      16.089  -2.051  -5.789  1.00 39.73
C
ANISOU 1404  CB  LYS A 108     6561   5035   3498   1262   -832    -67
ATOM   1405  CG  LYS A 108      16.830  -3.222  -6.434  1.00 43.69
C
ANISOU 1405  CG  LYS A 108     7267   5431   3902   1176   -692    -97
ATOM   1406  CD  LYS A 108      16.033  -3.775  -7.590  1.00 47.74
C
ANISOU 1406  CD  LYS A 108     8090   5897   4151   1323  -1050   -219
ATOM   1407  CE  LYS A 108      16.638  -5.029  -8.147  1.00 53.52
C
ANISOU 1407  CE  LYS A 108     8993   6537   4807   1224   -929   -274
ATOM   1408  NZ  LYS A 108      15.804  -5.578  -9.251  1.00 63.44
N1+
ANISOU 1408  NZ  LYS A 108    10574   7735   5795   1376  -1333   -421
ATOM   1409  H   LYS A 108      16.331  -3.268  -3.588  1.00  0.00
H
ATOM   1410  HA  LYS A 108      17.847  -1.273  -4.865  1.00  0.00
H
ATOM   1411  HB3 LYS A 108      15.929  -1.271  -6.533  1.00  0.00
H
ATOM   1412  HB2 LYS A 108      15.091  -2.374  -5.493  1.00  0.00
H
ATOM   1413  HG3 LYS A 108      16.986  -4.006  -5.693  1.00  0.00
H
ATOM   1414  HG2 LYS A 108      17.802  -2.883  -6.792  1.00  0.00
H
ATOM   1415  HD3 LYS A 108      15.967  -3.024  -8.377  1.00  0.00
H
ATOM   1416  HD2 LYS A 108      15.013  -3.979  -7.263  1.00  0.00
H
ATOM   1417  HE3 LYS A 108      16.726  -5.772  -7.355  1.00  0.00
H
ATOM   1418  HE2 LYS A 108      17.639  -4.814  -8.521  1.00  0.00
H
ATOM   1419  HZ1 LYS A 108      15.946  -6.576  -9.317  1.00  0.00
H
ATOM   1420  HZ2 LYS A 108      14.830  -5.389  -9.064  1.00  0.00
H
ATOM   1421  HZ3 LYS A 108      16.069  -5.142 -10.123  1.00  0.00
H
ATOM   1422  N   LEU A 109      15.064  -0.142  -3.454  1.00 37.78
N
```

```
ANISOU 1422  N   LEU A 109     5617   5037   3701   1245   -855     -5
ATOM   1423  CA  LEU A 109      14.399   1.084  -2.998  1.00 38.77
C
ANISOU 1423  CA  LEU A 109     5637   5199   3894   1361   -928     15
ATOM   1424  C   LEU A 109      15.255   1.831  -1.960  1.00 40.23
C
ANISOU 1424  C   LEU A 109     5707   5373   4205   1253   -602    114
ATOM   1425  O   LEU A 109      15.374   3.050  -2.055  1.00 41.39
O
ANISOU 1425  O   LEU A 109     6000   5438   4287   1372   -522    181
ATOM   1426  CB  LEU A 109      12.976   0.795  -2.472  1.00 39.97
C
ANISOU 1426  CB  LEU A 109     5396   5499   4292   1360  -1215   -120
ATOM   1427  CG  LEU A 109      12.116   2.011  -2.050  1.00 46.00
C
ANISOU 1427  CG  LEU A 109     6026   6284   5170   1509  -1332   -136
ATOM   1428  CD1 LEU A 109      11.880   2.969  -3.214  1.00 49.04
C
ANISOU 1428  CD1 LEU A 109     6797   6538   5299   1800  -1576   -119
ATOM   1429  CD2 LEU A 109      10.791   1.564  -1.486  1.00 50.61
C
ANISOU 1429  CD2 LEU A 109     6154   6976   6100   1461  -1516   -294
ATOM   1430  H   LEU A 109      14.643  -1.026  -3.207  1.00  0.00
H
ATOM   1431  HA  LEU A 109      14.297   1.738  -3.864  1.00  0.00
H
ATOM   1432  HB3 LEU A 109      13.037   0.094  -1.640  1.00  0.00
H
ATOM   1433  HB2 LEU A 109      12.431   0.212  -3.214  1.00  0.00
H
ATOM   1434  HG  LEU A 109      12.652   2.551  -1.270  1.00  0.00
H
ATOM   1435 HD11 LEU A 109      11.273   3.809  -2.877  1.00  0.00
H
ATOM   1436 HD12 LEU A 109      12.838   3.338  -3.581  1.00  0.00
H
ATOM   1437 HD13 LEU A 109      11.361   2.445  -4.017  1.00  0.00
H
ATOM   1438 HD21 LEU A 109      10.206   2.437  -1.197  1.00  0.00
H
ATOM   1439 HD22 LEU A 109      10.247   0.996  -2.241  1.00  0.00
H
ATOM   1440 HD23 LEU A 109      10.961   0.936  -0.612  1.00  0.00
H
ATOM   1441  N   ALA A 110      15.902   1.105  -1.022  1.00 34.46
N
ANISOU 1441  N   ALA A 110     4755   4694   3645   1043   -438    115
ATOM   1442  CA  ALA A 110      16.785   1.716  -0.019  1.00 32.00
C
ANISOU 1442  CA  ALA A 110     4346   4347   3464    947   -203    174
ATOM   1443  C   ALA A 110      17.978   2.424  -0.675  1.00 36.34
C
ANISOU 1443  C   ALA A 110     5165   4696   3946    990     46    248
ATOM   1444  O   ALA A 110      18.338   3.530  -0.258  1.00 34.69
O
ANISOU 1444  O   ALA A 110     4956   4419   3805   1014    181    287
```

```
ATOM   1445  CB  ALA A 110      17.282   0.670   0.962  1.00 30.67
C
ANISOU 1445  CB  ALA A 110     3966   4233   3455    752   -150    148
ATOM   1446  H   ALA A 110      15.788   0.102  -0.992  1.00  0.00
H
ATOM   1447  HA  ALA A 110      16.210   2.458   0.535  1.00  0.00
H
ATOM   1448  HB1 ALA A 110      17.934   1.141   1.697  1.00  0.00
H
ATOM   1449  HB2 ALA A 110      16.432   0.215   1.470  1.00  0.00
H
ATOM   1450  HB3 ALA A 110      17.837  -0.099   0.424  1.00  0.00
H
ATOM   1451  N   LYS A 111      18.571   1.805  -1.709  1.00 35.37
N
ANISOU 1451  N   LYS A 111     5280   4452   3708    996    140    257
ATOM   1452  CA  LYS A 111      19.700   2.396  -2.434  1.00 37.41
C
ANISOU 1452  CA  LYS A 111     5823   4463   3930   1026    467    316
ATOM   1453  C   LYS A 111      19.256   3.662  -3.188  1.00 44.66
C
ANISOU 1453  C   LYS A 111     7080   5268   4621   1233    490    384
ATOM   1454  O   LYS A 111      19.981   4.660  -3.179  1.00 45.34
O
ANISOU 1454  O   LYS A 111     7276   5174   4776   1246    775    438
ATOM   1455  CB  LYS A 111      20.345   1.360  -3.373  1.00 40.89
C
ANISOU 1455  CB  LYS A 111     6474   4781   4283    990    593    298
ATOM   1456  CG  LYS A 111      21.690   1.787  -3.951  1.00 52.13
C
ANISOU 1456  CG  LYS A 111     8074   5913   5821    948   1040    330
ATOM   1457  CD  LYS A 111      22.401   0.605  -4.595  1.00 61.71
C
ANISOU 1457  CD  LYS A 111     9367   7018   7061    864   1191    286
ATOM   1458  CE  LYS A 111      23.544   1.018  -5.492  1.00 76.92
C
ANISOU 1458  CE  LYS A 111    11681   8597   8947    900   1668    319
ATOM   1459  NZ  LYS A 111      24.763   1.376  -4.719  1.00 86.35
N1+
ANISOU 1459  NZ  LYS A 111    12621   9594  10594    777   2025    295
ATOM   1460  H   LYS A 111      18.241   0.900  -2.012  1.00  0.00
H
ATOM   1461  HA  LYS A 111      20.449   2.692  -1.700  1.00  0.00
H
ATOM   1462  HB3 LYS A 111      19.657   1.134  -4.188  1.00  0.00
H
ATOM   1463  HB2 LYS A 111      20.464   0.416  -2.842  1.00  0.00
H
ATOM   1464  HG3 LYS A 111      22.313   2.196  -3.156  1.00  0.00
H
ATOM   1465  HG2 LYS A 111      21.534   2.567  -4.697  1.00  0.00
H
ATOM   1466  HD3 LYS A 111      21.683   0.020  -5.170  1.00  0.00
H
ATOM   1467  HD2 LYS A 111      22.774  -0.061  -3.817  1.00  0.00
H
```

```
ATOM   1468  HE3 LYS A 111      23.238   1.869  -6.101  1.00  0.00
H
ATOM   1469  HE2 LYS A 111      23.777   0.205  -6.180  1.00  0.00
H
ATOM   1470  HZ1 LYS A 111      24.974   0.639  -4.062  1.00  0.00
H
ATOM   1471  HZ2 LYS A 111      25.541   1.495  -5.352  1.00  0.00
H
ATOM   1472  HZ3 LYS A 111      24.603   2.238  -4.217  1.00  0.00
H
ATOM   1473  N   LYS A 112      18.042   3.634  -3.778  1.00 43.39
N
ANISOU 1473  N   LYS A 112     7067   5195   4224   1400    167    366
ATOM   1474  CA  LYS A 112      17.447   4.762  -4.499  1.00 46.33
C
ANISOU 1474  CA  LYS A 112     7790   5457   4355   1641     86    424
ATOM   1475  C   LYS A 112      17.170   5.913  -3.533  1.00 50.86
C
ANISOU 1475  C   LYS A 112     8123   6094   5108   1658     92    447
ATOM   1476  O   LYS A 112      17.561   7.049  -3.814  1.00 52.34
O
ANISOU 1476  O   LYS A 112     8571   6099   5215   1766    286    530
ATOM   1477  CB  LYS A 112      16.148   4.324  -5.202  1.00 51.17
C
ANISOU 1477  CB  LYS A 112     8544   6151   4749   1823   -369    353
ATOM   1478  CG  LYS A 112      15.608   5.324  -6.226  1.00 67.58
C
ANISOU 1478  CG  LYS A 112    11088   8067   6522   2124   -526    408
ATOM   1479  CD  LYS A 112      14.091   5.231  -6.386  0.00 79.43
C
ANISOU 1479  CD  LYS A 112    12433   9710   8037   2297  -1088    291
ATOM   1480  CE  LYS A 112      13.648   4.205  -7.404  0.00 93.23
C
ANISOU 1480  CE  LYS A 112    14522  11395   9505   2443  -1441    203
ATOM   1481  NZ  LYS A 112      12.167   4.083  -7.445  0.00103.12
N1+
ANISOU 1481  NZ  LYS A 112    15369  12826  10984   2497  -1972     20
ATOM   1482  H   LYS A 112      17.481   2.795  -3.739  1.00  0.00
H
ATOM   1483  HA  LYS A 112      18.154   5.104  -5.255  1.00  0.00
H
ATOM   1484  HB3 LYS A 112      15.382   4.125  -4.453  1.00  0.00
H
ATOM   1485  HB2 LYS A 112      16.307   3.361  -5.687  1.00  0.00
H
ATOM   1486  HG3 LYS A 112      16.085   5.149  -7.190  1.00  0.00
H
ATOM   1487  HG2 LYS A 112      15.879   6.335  -5.923  1.00  0.00
H
ATOM   1488  HD3 LYS A 112      13.696   6.209  -6.662  1.00  0.00
H
ATOM   1489  HD2 LYS A 112      13.639   5.001  -5.421  1.00  0.00
H
ATOM   1490  HE3 LYS A 112      14.085   3.237  -7.158  1.00  0.00
H
ATOM   1491  HE2 LYS A 112      14.016   4.490  -8.390  1.00  0.00
H
```

```
ATOM   1492  HZ1 LYS A 112      11.762   4.974  -7.693  1.00  0.00
H
ATOM   1493  HZ2 LYS A 112      11.905   3.392  -8.133  1.00  0.00
H
ATOM   1494  HZ3 LYS A 112      11.827   3.800  -6.537  1.00  0.00
H
ATOM   1495  N   ILE A 113      16.496   5.611  -2.399  1.00 46.00
N
ANISOU 1495  N   ILE A 113     7046   5707   4726   1553    -89    372
ATOM   1496  CA  ILE A 113      16.134   6.563  -1.350  1.00 45.14
C
ANISOU 1496  CA  ILE A 113     6697   5664   4790   1555    -80    372
ATOM   1497  C   ILE A 113      17.403   7.213  -0.759  1.00 45.92
C
ANISOU 1497  C   ILE A 113     6780   5632   5034   1433    269    419
ATOM   1498  O   ILE A 113      17.439   8.439  -0.640  1.00 45.66
O
ANISOU 1498  O   ILE A 113     6830   5501   5019   1517    378    462
ATOM   1499  CB  ILE A 113      15.184   5.885  -0.308  1.00 47.70
C
ANISOU 1499  CB  ILE A 113     6596   6217   5312   1461   -287    272
ATOM   1500  CG1 ILE A 113      13.745   5.768  -0.870  1.00 50.61
C
ANISOU 1500  CG1 ILE A 113     6932   6653   5644   1637   -643    195
ATOM   1501  CG2 ILE A 113      15.170   6.583   1.035  1.00 47.16
C
ANISOU 1501  CG2 ILE A 113     6289   6197   5432   1385   -167    264
ATOM   1502  CD1 ILE A 113      12.738   4.987   0.033  1.00 58.58
C
ANISOU 1502  CD1 ILE A 113     7508   7838   6913   1531   -794     69
ATOM   1503  H   ILE A 113      16.201   4.661  -2.225  1.00  0.00
H
ATOM   1504  HA  ILE A 113      15.563   7.359  -1.828  1.00  0.00
H
ATOM   1505  HB  ILE A 113      15.552   4.872  -0.144  1.00  0.00
H
ATOM   1506 HG13 ILE A 113      13.780   5.304  -1.856  1.00  0.00
H
ATOM   1507 HG12 ILE A 113      13.353   6.766  -1.067  1.00  0.00
H
ATOM   1508 HG21 ILE A 113      14.491   6.061   1.709  1.00  0.00
H
ATOM   1509 HG22 ILE A 113      16.175   6.579   1.457  1.00  0.00
H
ATOM   1510 HG23 ILE A 113      14.834   7.612   0.907  1.00  0.00
H
ATOM   1511 HD11 ILE A 113      11.761   4.962  -0.449  1.00  0.00
H
ATOM   1512 HD12 ILE A 113      13.096   3.968   0.181  1.00  0.00
H
ATOM   1513 HD13 ILE A 113      12.653   5.485   0.999  1.00  0.00
H
ATOM   1514  N   LEU A 114      18.461   6.407  -0.481  1.00 39.95
N
ANISOU 1514  N   LEU A 114     5924   4845   4412   1249    429    397
ATOM   1515  CA  LEU A 114      19.746   6.888   0.050  1.00 38.81
C
```

```
ANISOU 1515  CA  LEU A 114     5706   4545   4494   1128    708    395
ATOM   1516  C   LEU A 114      20.386   7.925  -0.879  1.00 46.26
C
ANISOU 1516  C   LEU A 114     6980   5209   5386   1221   1017    465
ATOM   1517  O   LEU A 114      20.796   8.996  -0.416  1.00 45.22
O
ANISOU 1517  O   LEU A 114     6804   4959   5419   1208   1181    465
ATOM   1518  CB  LEU A 114      20.732   5.725   0.323  1.00 37.53
C
ANISOU 1518  CB  LEU A 114     5379   4367   4512    949    767    339
ATOM   1519  CG  LEU A 114      22.094   6.107   0.939  1.00 42.01
C
ANISOU 1519  CG  LEU A 114     5788   4758   5415    824    971    286
ATOM   1520  CD1 LEU A 114      21.940   6.658   2.359  1.00 41.14
C
ANISOU 1520  CD1 LEU A 114     5448   4745   5437    780    818    232
ATOM   1521  CD2 LEU A 114      23.047   4.911   0.954  1.00 43.92
C
ANISOU 1521  CD2 LEU A 114     5898   4941   5847    693   1011    228
ATOM   1522  H   LEU A 114      18.392   5.412  -0.638  1.00  0.00
H
ATOM   1523  HA  LEU A 114      19.545   7.379   1.002  1.00  0.00
H
ATOM   1524  HB3 LEU A 114      20.899   5.171  -0.601  1.00  0.00
H
ATOM   1525  HB2 LEU A 114      20.246   4.984   0.958  1.00  0.00
H
ATOM   1526  HG  LEU A 114      22.539   6.886   0.320  1.00  0.00
H
ATOM   1527 HD11 LEU A 114      22.921   6.915   2.758  1.00  0.00
H
ATOM   1528 HD12 LEU A 114      21.312   7.549   2.338  1.00  0.00
H
ATOM   1529 HD13 LEU A 114      21.477   5.903   2.994  1.00  0.00
H
ATOM   1530 HD21 LEU A 114      23.999   5.210   1.393  1.00  0.00
H
ATOM   1531 HD22 LEU A 114      22.611   4.106   1.545  1.00  0.00
H
ATOM   1532 HD23 LEU A 114      23.211   4.564  -0.066  1.00  0.00
H
ATOM   1533  N   HIS A 115      20.428   7.618  -2.187  1.00 46.62
N
ANISOU 1533  N   HIS A 115     7397   5124   5192   1319   1111    520
ATOM   1534  CA  HIS A 115      20.985   8.507  -3.204  1.00 50.79
C
ANISOU 1534  CA  HIS A 115     8357   5335   5606   1423   1467    604
ATOM   1535  C   HIS A 115      20.212   9.823  -3.299  1.00 55.61
C
ANISOU 1535  C   HIS A 115     9159   5909   6063   1614   1397    674
ATOM   1536  O   HIS A 115      20.837  10.879  -3.357  1.00 56.80
O
ANISOU 1536  O   HIS A 115     9419   5830   6333   1616   1717    712
ATOM   1537  CB  HIS A 115      21.062   7.799  -4.561  1.00 54.82
C
ANISOU 1537  CB  HIS A 115     9314   5711   5805   1511   1550    646
```

```
ATOM   1538  CG  HIS A 115      22.091   8.380  -5.477  0.00 61.92
C
ANISOU 1538  CG  HIS A 115    10622   6215   6690   1522   2091    709
ATOM   1539  ND1 HIS A 115      23.381   8.017  -5.667  0.00 67.32
N
ANISOU 1539  ND1 HIS A 115    11880   6661   7036   1738   2242    823
ATOM   1540  CD2 HIS A 115      21.800   9.456  -6.304  0.00 64.81
C
ANISOU 1540  CD2 HIS A 115    10898   6356   7370   1347   2529    664
ATOM   1541  CE1 HIS A 115      23.894   8.863  -6.629  0.00 69.14
C
ANISOU 1541  CE1 HIS A 115    12384   6521   7363   1674   2822    854
ATOM   1542  NE2 HIS A 115      22.914   9.704  -6.977  0.00 68.10
N
ANISOU 1542  NE2 HIS A 115    11833   6384   7660   1434   3019    749
ATOM   1543  H   HIS A 115      20.062   6.734  -2.510  1.00  0.00
H
ATOM   1544  HA  HIS A 115      22.005   8.748  -2.903  1.00  0.00
H
ATOM   1545  HB3 HIS A 115      20.086   7.836  -5.044  1.00  0.00
H
ATOM   1546  HB2 HIS A 115      21.272   6.741  -4.406  1.00  0.00
H
ATOM   1547  HD1 HIS A 115      23.800   7.198  -5.101  1.00  0.00
H
ATOM   1548  HD2 HIS A 115      20.878   9.869  -6.290  1.00  0.00
H
ATOM   1549  HE1 HIS A 115      24.853   8.762  -6.931  1.00  0.00
H
ATOM   1550  N   ALA A 116      18.862   9.761  -3.242  1.00 51.94
N
ANISOU 1550  N   ALA A 116     8681   5653   5399   1768    977    670
ATOM   1551  CA  ALA A 116      17.976  10.927  -3.295  1.00 53.05
C
ANISOU 1551  CA  ALA A 116     8959   5776   5422   1979    829    719
ATOM   1552  C   ALA A 116      18.132  11.835  -2.069  1.00 54.60
C
ANISOU 1552  C   ALA A 116     8819   6011   5914   1887    927    690
ATOM   1553  O   ALA A 116      18.114  13.058  -2.204  1.00 55.57
O
ANISOU 1553  O   ALA A 116     9137   5970   6007   2011   1057    752
ATOM   1554  CB  ALA A 116      16.531  10.479  -3.435  1.00 54.45
C
ANISOU 1554  CB  ALA A 116     9060   6168   5459   2133    331    667
ATOM   1555  H   ALA A 116      18.401   8.867  -3.157  1.00  0.00
H
ATOM   1556  HA  ALA A 116      18.236  11.508  -4.180  1.00  0.00
H
ATOM   1557  HB1 ALA A 116      15.881  11.353  -3.473  1.00  0.00
H
ATOM   1558  HB2 ALA A 116      16.416   9.901  -4.352  1.00  0.00
H
ATOM   1559  HB3 ALA A 116      16.257   9.861  -2.580  1.00  0.00
H
ATOM   1560  N   GLY A 117      18.300  11.229  -0.897  1.00 47.65
N
ANISOU 1560  N   GLY A 117     7491   5322   5292   1683    866    595
```

```
ATOM   1561  CA  GLY A 117      18.483  11.951   0.356  1.00 45.74
C
ANISOU 1561  CA  GLY A 117     6963   5116   5301   1587    924    542
ATOM   1562  C   GLY A 117      19.819  12.649   0.475  1.00 48.97
C
ANISOU 1562  C   GLY A 117     7407   5271   5929   1479   1285    536
ATOM   1563  O   GLY A 117      19.881  13.774   0.973  1.00 48.70
O
ANISOU 1563  O   GLY A 117     7341   5149   6015   1499   1381    526
ATOM   1564  H   GLY A 117      18.305  10.220  -0.846  1.00  0.00
H
ATOM   1565  HA3 GLY A 117      18.360  11.262   1.191  1.00  0.00
H
ATOM   1566  HA2 GLY A 117      17.682  12.681   0.473  1.00  0.00
H
ATOM   1567  N   PHE A 118      20.898  11.999   0.011  1.00 44.73
N
ANISOU 1567  N   PHE A 118     6912   4592   5493   1360   1500    524
ATOM   1568  CA  PHE A 118      22.237  12.568   0.113  1.00 45.60
C
ANISOU 1568  CA  PHE A 118     6979   4418   5927   1236   1863    478
ATOM   1569  C   PHE A 118      22.528  13.621  -0.969  1.00 54.88
C
ANISOU 1569  C   PHE A 118     8577   5260   7016   1355   2250    580
ATOM   1570  O   PHE A 118      23.468  14.398  -0.803  1.00 56.83
O
ANISOU 1570  O   PHE A 118     8772   5241   7581   1264   2581    532
ATOM   1571  CB  PHE A 118      23.316  11.470   0.193  1.00 46.75
C
ANISOU 1571  CB  PHE A 118     6921   4515   6326   1054   1949    391
ATOM   1572  CG  PHE A 118      23.573  11.083   1.634  1.00 46.47
C
ANISOU 1572  CG  PHE A 118     6465   4638   6555    910   1691    257
ATOM   1573  CD1 PHE A 118      22.676  10.273   2.326  1.00 47.60
C
ANISOU 1573  CD1 PHE A 118     6472   5093   6522    909   1327    249
ATOM   1574  CD2 PHE A 118      24.656  11.609   2.329  1.00 49.39
C
ANISOU 1574  CD2 PHE A 118     6608   4812   7346    791   1806    130
ATOM   1575  CE1 PHE A 118      22.875   9.974   3.678  1.00 47.35
C
ANISOU 1575  CE1 PHE A 118     6166   5166   6659    803   1106    141
ATOM   1576  CE2 PHE A 118      24.859  11.299   3.679  1.00 51.08
C
ANISOU 1576  CE2 PHE A 118     6521   5145   7743    696   1504      1
ATOM   1577  CZ  PHE A 118      23.970  10.482   4.343  1.00 47.41
C
ANISOU 1577  CZ  PHE A 118     6009   4982   7025    709   1167     20
ATOM   1578  H   PHE A 118      20.794  11.092  -0.422  1.00  0.00
H
ATOM   1579  HA  PHE A 118      22.270  13.096   1.066  1.00  0.00
H
ATOM   1580  HB3 PHE A 118      24.240  11.836  -0.255  1.00  0.00
H
ATOM   1581  HB2 PHE A 118      22.985  10.594  -0.365  1.00  0.00
H
```

```
ATOM   1582  HD1 PHE A 118      21.813   9.867   1.819  1.00  0.00
H
ATOM   1583  HD2 PHE A 118      25.352  12.265   1.828  1.00  0.00
H
ATOM   1584  HE1 PHE A 118      22.170   9.345   4.201  1.00  0.00
H
ATOM   1585  HE2 PHE A 118      25.714  11.700   4.203  1.00  0.00
H
ATOM   1586  HZ  PHE A 118      24.130  10.240   5.383  1.00  0.00
H
ATOM   1587  N   LYS A 119      21.699  13.710  -2.023  1.00 53.74
N
ANISOU 1587  N   LYS A 119     8863   5101   6454   1567   2195    707
ATOM   1588  CA  LYS A 119      21.871  14.766  -3.030  1.00 57.68
C
ANISOU 1588  CA  LYS A 119     9876   5256   6783   1721   2545    828
ATOM   1589  C   LYS A 119      21.117  16.006  -2.544  1.00 62.75
C
ANISOU 1589  C   LYS A 119    10519   5923   7402   1856   2419    860
ATOM   1590  O   LYS A 119      21.600  17.133  -2.702  1.00 64.70
O
ANISOU 1590  O   LYS A 119    10954   5872   7756   1878   2770    901
ATOM   1591  CB  LYS A 119      21.374  14.330  -4.425  1.00 62.96
C
ANISOU 1591  CB  LYS A 119    11105   5854   6962   1928   2494    946
ATOM   1592  CG  LYS A 119      22.191  13.210  -5.084  0.00 82.40
C
ANISOU 1592  CG  LYS A 119    13676   8224   9409   1815   2704    924
ATOM   1593  CD  LYS A 119      21.669  12.777  -6.452  0.00 96.93
C
ANISOU 1593  CD  LYS A 119    15767   9616  11446   1716   3383    945
ATOM   1594  CE  LYS A 119      22.531  11.695  -7.049  0.00106.83
C
ANISOU 1594  CE  LYS A 119    16656  10860  13075   1471   3563    821
ATOM   1595  NZ  LYS A 119      22.019  11.248  -8.370  0.00119.45
N1+
ANISOU 1595  NZ  LYS A 119    18741  12140  14507   1487   4013    872
ATOM   1596  H   LYS A 119      20.946  13.046  -2.131  1.00  0.00
H
ATOM   1597  HA  LYS A 119      22.931  15.010  -3.100  1.00  0.00
H
ATOM   1598  HB3 LYS A 119      21.350  15.196  -5.086  1.00  0.00
H
ATOM   1599  HB2 LYS A 119      20.331  14.022  -4.356  1.00  0.00
H
ATOM   1600  HG3 LYS A 119      22.222  12.347  -4.419  1.00  0.00
H
ATOM   1601  HG2 LYS A 119      23.230  13.528  -5.178  1.00  0.00
H
ATOM   1602  HD3 LYS A 119      21.650  13.637  -7.122  1.00  0.00
H
ATOM   1603  HD2 LYS A 119      20.647  12.412  -6.351  1.00  0.00
H
ATOM   1604  HE3 LYS A 119      22.569  10.845  -6.368  1.00  0.00
H
ATOM   1605  HE2 LYS A 119      23.550  12.064  -7.161  1.00  0.00
H
```

```
ATOM   1606  HZ1 LYS A 119      22.005  12.030  -9.009  1.00  0.00
H
ATOM   1607  HZ2 LYS A 119      22.621  10.525  -8.738  1.00  0.00
H
ATOM   1608  HZ3 LYS A 119      21.083  10.884  -8.263  1.00  0.00
H
ATOM   1609  N   MET A 120      19.944  15.779  -1.919  1.00 57.26
N
ANISOU 1609  N   MET A 120     9589   5560   6606   1935   1947    828
ATOM   1610  CA  MET A 120      19.053  16.794  -1.358  1.00 56.77
C
ANISOU 1610  CA  MET A 120     9457   5568   6544   2068   1771    835
ATOM   1611  C   MET A 120      19.656  17.444  -0.099  1.00 55.99
C
ANISOU 1611  C   MET A 120     8996   5451   6828   1885   1917    728
ATOM   1612  O   MET A 120      19.515  18.652   0.093  1.00 56.43
O
ANISOU 1612  O   MET A 120     9138   5359   6943   1966   2037    750
ATOM   1613  CB  MET A 120      17.694  16.142  -1.054  1.00 58.41
C
ANISOU 1613  CB  MET A 120     9458   6113   6623   2169   1277    794
ATOM   1614  CG  MET A 120      16.674  17.055  -0.409  1.00 62.75
C
ANISOU 1614  CG  MET A 120     9853   6751   7237   2295   1092    770
ATOM   1615  SD  MET A 120      15.225  16.128   0.145  1.00 66.51
S
ANISOU 1615  SD  MET A 120     9950   7589   7730   2337    613    669
ATOM   1616  CE  MET A 120      15.871  15.313   1.568  1.00 59.90
C
ANISOU 1616  CE  MET A 120     8670   6941   7147   2008    697    549
ATOM   1617  H   MET A 120      19.608  14.834  -1.804  1.00  0.00
H
ATOM   1618  HA  MET A 120      18.902  17.571  -2.107  1.00  0.00
H
ATOM   1619  HB3 MET A 120      17.847  15.268  -0.421  1.00  0.00
H
ATOM   1620  HB2 MET A 120      17.278  15.730  -1.973  1.00  0.00
H
ATOM   1621  HG3 MET A 120      16.365  17.817  -1.125  1.00  0.00
H
ATOM   1622  HG2 MET A 120      17.128  17.562   0.443  1.00  0.00
H
ATOM   1623  HE1 MET A 120      15.092  14.700   2.021  1.00  0.00
H
ATOM   1624  HE2 MET A 120      16.708  14.678   1.275  1.00  0.00
H
ATOM   1625  HE3 MET A 120      16.214  16.056   2.288  1.00  0.00
H
ATOM   1626  N   MET A 121      20.296  16.639   0.759  1.00 48.67
N
ANISOU 1626  N   MET A 121     7690   4654   6147   1656   1872    604
ATOM   1627  CA  MET A 121      20.918  17.107   2.000  1.00 46.57
C
ANISOU 1627  CA  MET A 121     7102   4365   6228   1489   1920    470
ATOM   1628  C   MET A 121      22.450  17.110   1.879  1.00 52.14
C
ANISOU 1628  C   MET A 121     7738   4789   7282   1309   2252    393
```

```
ATOM   1629  O   MET A 121      23.156  16.922   2.874  1.00 50.22
O
ANISOU 1629  O   MET A 121     7167   4558   7356   1140   2177    239
ATOM   1630  CB  MET A 121      20.452  16.253   3.191  1.00 45.54
C
ANISOU 1630  CB  MET A 121     6630   4554   6120   1393   1572    367
ATOM   1631  CG  MET A 121      18.957  16.259   3.380  1.00 47.16
C
ANISOU 1631  CG  MET A 121     6827   4992   6099   1545   1311    405
ATOM   1632  SD  MET A 121      18.422  15.267   4.780  1.00 47.56
S
ANISOU 1632  SD  MET A 121     6545   5344   6181   1418   1032    290
ATOM   1633  CE  MET A 121      18.711  13.569   4.149  1.00 43.56
C
ANISOU 1633  CE  MET A 121     6012   4963   5575   1343    919    317
ATOM   1634  H   MET A 121      20.369  15.650   0.565  1.00  0.00
H
ATOM   1635  HA  MET A 121      20.592  18.133   2.174  1.00  0.00
H
ATOM   1636  HB3 MET A 121      20.933  16.609   4.102  1.00  0.00
H
ATOM   1637  HB2 MET A 121      20.796  15.227   3.057  1.00  0.00
H
ATOM   1638  HG3 MET A 121      18.476  15.894   2.473  1.00  0.00
H
ATOM   1639  HG2 MET A 121      18.613  17.285   3.511  1.00  0.00
H
ATOM   1640  HE1 MET A 121      18.424  12.844   4.911  1.00  0.00
H
ATOM   1641  HE2 MET A 121      18.114  13.408   3.251  1.00  0.00
H
ATOM   1642  HE3 MET A 121      19.767  13.445   3.909  1.00  0.00
H
ATOM   1643  N   SER A 122      22.957  17.358   0.657  1.00 52.66
N
ANISOU 1643  N   SER A 122     8136   4565   7309   1357   2625    488
ATOM   1644  CA  SER A 122      24.391  17.402   0.370  1.00 55.49
C
ANISOU 1644  CA  SER A 122     8431   4586   8065   1190   3040    409
ATOM   1645  C   SER A 122      25.055  18.608   1.023  1.00 61.78
C
ANISOU 1645  C   SER A 122     9057   5141   9276   1101   3238    291
ATOM   1646  O   SER A 122      24.379  19.594   1.325  1.00 60.49
O
ANISOU 1646  O   SER A 122     9002   4994   8986   1216   3178    335
ATOM   1647  CB  SER A 122      24.644  17.402  -1.136  1.00 61.65
C
ANISOU 1647  CB  SER A 122     9696   5077   8650   1279   3463    551
ATOM   1648  OG  SER A 122      24.261  18.616  -1.759  1.00 72.10
O
ANISOU 1648  OG  SER A 122    11448   6161   9785   1448   3713    681
ATOM   1649  H   SER A 122      22.337  17.526  -0.122  1.00  0.00
H
ATOM   1650  HA  SER A 122      24.844  16.503   0.787  1.00  0.00
H
ATOM   1651  HB3 SER A 122      24.103  16.574  -1.594  1.00  0.00
H
```

```
ATOM   1652  HB2 SER A 122      25.701  17.215  -1.326  1.00  0.00
H
ATOM   1653  HG  SER A 122      23.408  18.503  -2.185  1.00  0.00
H
ATOM   1654  N   LYS A 123      26.383  18.516   1.243  1.00 61.64
N
ANISOU 1654  N   LYS A 123     8748   4878   9794    900   3459    122
ATOM   1655  CA  LYS A 123      27.199  19.575   1.842  1.00 63.77
C
ANISOU 1655  CA  LYS A 123     8796   4860  10574    785   3648    -45
ATOM   1656  C   LYS A 123      27.060  20.895   1.081  1.00 69.01
C
ANISOU 1656  C   LYS A 123     9852   5203  11167    891   4102     81
ATOM   1657  O   LYS A 123      27.036  21.951   1.709  1.00 68.03
O
ANISOU 1657  O   LYS A 123     9652   4980  11215    887   4107      9
ATOM   1658  CB  LYS A 123      28.666  19.133   1.941  1.00 69.06
C
ANISOU 1658  CB  LYS A 123     9098   5253  11889    571   3851   -256
ATOM   1659  CG  LYS A 123      29.132  18.893   3.374  1.00 88.88
C
ANISOU 1659  CG  LYS A 123    11112   7873  14784    446   3393   -513
ATOM   1660  CD  LYS A 123      30.169  19.936   3.802  1.00105.11
C
ANISOU 1660  CD  LYS A 123    12885   9517  17536    306   3620   -751
ATOM   1661  CE  LYS A 123      30.703  19.715   5.199  1.00118.19
C
ANISOU 1661  CE  LYS A 123    14090  11236  19579    206   3099  -1037
ATOM   1662  NZ  LYS A 123      29.772  20.223   6.243  1.00125.73
N1+
ANISOU 1662  NZ  LYS A 123    15127  12440  20204    291   2687  -1047
ATOM   1663  H   LYS A 123      26.880  17.674   0.989  1.00  0.00
H
ATOM   1664  HA  LYS A 123      26.835  19.739   2.856  1.00  0.00
H
ATOM   1665  HB3 LYS A 123      29.303  19.885   1.475  1.00  0.00
H
ATOM   1666  HB2 LYS A 123      28.810  18.224   1.357  1.00  0.00
H
ATOM   1667  HG3 LYS A 123      29.566  17.896   3.452  1.00  0.00
H
ATOM   1668  HG2 LYS A 123      28.275  18.936   4.046  1.00  0.00
H
ATOM   1669  HD3 LYS A 123      29.730  20.931   3.736  1.00  0.00
H
ATOM   1670  HD2 LYS A 123      30.997  19.934   3.094  1.00  0.00
H
ATOM   1671  HE3 LYS A 123      31.668  20.212   5.301  1.00  0.00
H
ATOM   1672  HE2 LYS A 123      30.878  18.651   5.356  1.00  0.00
H
ATOM   1673  HZ1 LYS A 123      29.921  21.213   6.374  1.00  0.00
H
ATOM   1674  HZ2 LYS A 123      28.818  20.062   5.952  1.00  0.00
H
ATOM   1675  HZ3 LYS A 123      29.944  19.739   7.113  1.00  0.00
H
```

```
ATOM   1676  N   GLU A 124      26.905  20.819  -0.258  1.00 67.15
N
ANISOU 1676  N   GLU A 124    10089   4792  10631   1001   4470    274
ATOM   1677  CA  GLU A 124      26.704  21.949  -1.170  1.00 69.75
C
ANISOU 1677  CA  GLU A 124    10946   4784  10770   1147   4919    442
ATOM   1678  C   GLU A 124      25.354  22.639  -0.912  1.00 70.48
C
ANISOU 1678  C   GLU A 124    11252   5112  10413   1377   4565    570
ATOM   1679  O   GLU A 124      25.297  23.868  -0.834  1.00 70.32
O
ANISOU 1679  O   GLU A 124    11357   4871  10490   1427   4763    585
ATOM   1680  CB  GLU A 124      26.802  21.457  -2.626  1.00 73.36
C
ANISOU 1680  CB  GLU A 124    11939   5034  10898   1240   5302    618
ATOM   1681  CG  GLU A 124      26.717  22.549  -3.676  1.00 89.45
C
ANISOU 1681  CG  GLU A 124    14658   6662  12668   1416   5802    813
ATOM   1682  CD  GLU A 124      25.563  22.376  -4.644  1.00114.26
C
ANISOU 1682  CD  GLU A 124    18434   9944  15037   1730   5559   1055
ATOM   1683  OE1 GLU A 124      24.394  22.466  -4.202  1.00106.71
O
ANISOU 1683  OE1 GLU A 124    17353   9397  13795   1873   4947   1079
ATOM   1684  OE2 GLU A 124      25.827  22.155  -5.848  1.00113.21
O1-
ANISOU 1684  OE2 GLU A 124    18942   9474  14597   1843   5987   1208
ATOM   1685  H   GLU A 124      26.922  19.920  -0.718  1.00  0.00
H
ATOM   1686  HA  GLU A 124      27.499  22.675  -0.998  1.00  0.00
H
ATOM   1687  HB3 GLU A 124      26.022  20.718  -2.810  1.00  0.00
H
ATOM   1688  HB2 GLU A 124      27.731  20.902  -2.757  1.00  0.00
H
ATOM   1689  HG3 GLU A 124      27.653  22.589  -4.233  1.00  0.00
H
ATOM   1690  HG2 GLU A 124      26.634  23.518  -3.184  1.00  0.00
H
ATOM   1691  N   ARG A 125      24.280  21.839  -0.785  1.00 63.75
N
ANISOU 1691  N   ARG A 125    10410   4686   9127   1510   4054    643
ATOM   1692  CA  ARG A 125      22.917  22.307  -0.526  1.00 62.29
C
ANISOU 1692  CA  ARG A 125    10346   4743   8578   1732   3675    735
ATOM   1693  C   ARG A 125      22.828  22.922   0.876  1.00 62.44
C
ANISOU 1693  C   ARG A 125     9953   4880   8892   1640   3480    576
ATOM   1694  O   ARG A 125      22.141  23.930   1.059  1.00 62.53
O
ANISOU 1694  O   ARG A 125    10101   4854   8803   1787   3448    628
ATOM   1695  CB  ARG A 125      21.916  21.146  -0.674  1.00 62.13
C
ANISOU 1695  CB  ARG A 125    10314   5122   8171   1844   3197    790
ATOM   1696  CG  ARG A 125      21.803  20.589  -2.099  1.00 75.44
C
ANISOU 1696  CG  ARG A 125    12497   6697   9470   1989   3304    946
```

```
ATOM   1697  CD  ARG A 125      20.555  21.041  -2.834  1.00 87.12
C
ANISOU 1697  CD  ARG A 125    14441   8188  10471   2320   3075   1108
ATOM   1698  NE  ARG A 125      19.334  20.535  -2.200  1.00 92.29
N
ANISOU 1698  NE  ARG A 125    14798   9260  11008   2411   2494   1060
ATOM   1699  CZ  ARG A 125      18.339  21.306  -1.775  1.00101.05
C
ANISOU 1699  CZ  ARG A 125    15856  10457  12080   2580   2250   1069
ATOM   1700  NH1 ARG A 125      18.393  22.623  -1.932  1.00 86.72
N
ANISOU 1700  NH1 ARG A 125    14299   8358  10291   2691   2497   1138
ATOM   1701  NH2 ARG A 125      17.272  20.763  -1.201  1.00 81.97
N1+
ANISOU 1701  NH2 ARG A 125    13128   8384   9630   2639   1788   1001
ATOM   1702  H   ARG A 125      24.383  20.838  -0.868  1.00  0.00
H
ATOM   1703  HA  ARG A 125      22.669  23.075  -1.258  1.00  0.00
H
ATOM   1704  HB3 ARG A 125      20.933  21.471  -0.334  1.00  0.00
H
ATOM   1705  HB2 ARG A 125      22.193  20.341   0.007  1.00  0.00
H
ATOM   1706  HG3 ARG A 125      21.828  19.500  -2.065  1.00  0.00
H
ATOM   1707  HG2 ARG A 125      22.684  20.878  -2.671  1.00  0.00
H
ATOM   1708  HD3 ARG A 125      20.597  20.695  -3.867  1.00  0.00
H
ATOM   1709  HD2 ARG A 125      20.525  22.130  -2.861  1.00  0.00
H
ATOM   1710  HE  ARG A 125      19.276  19.532  -2.094  1.00  0.00
H
ATOM   1711 HH12 ARG A 125      17.632  23.202  -1.606  1.00  0.00
H
ATOM   1712 HH11 ARG A 125      19.195  23.046  -2.377  1.00  0.00
H
ATOM   1713 HH22 ARG A 125      16.517  21.352  -0.879  1.00  0.00
H
ATOM   1714 HH21 ARG A 125      17.215  19.761  -1.086  1.00  0.00
H
ATOM   1715  N   LEU A 126      23.556  22.337   1.851  1.00 55.61
N
ANISOU 1715  N   LEU A 126     8621   4125   8385   1411   3347    377
ATOM   1716  CA  LEU A 126      23.599  22.827   3.226  1.00 54.00
C
ANISOU 1716  CA  LEU A 126     8072   4002   8443   1312   3140    196
ATOM   1717  C   LEU A 126      24.474  24.073   3.363  1.00 58.79
C
ANISOU 1717  C   LEU A 126     8662   4201   9473   1223   3515     94
ATOM   1718  O   LEU A 126      24.145  24.947   4.168  1.00 57.92
O
ANISOU 1718  O   LEU A 126     8462   4094   9450   1237   3407      6
ATOM   1719  CB  LEU A 126      24.019  21.731   4.219  1.00 51.81
C
ANISOU 1719  CB  LEU A 126     7390   3953   8342   1136   2807     19
```

```
ATOM   1720  CG  LEU A 126      23.067  20.525   4.361  1.00 54.14
C
ANISOU 1720  CG  LEU A 126     7653   4659   8259   1202   2419      90
ATOM   1721  CD1 LEU A 126      23.730  19.397   5.133  1.00 53.19
C
ANISOU 1721  CD1 LEU A 126     7219   4668   8325   1028   2183     -59
ATOM   1722  CD2 LEU A 126      21.740  20.914   5.018  1.00 54.82
C
ANISOU 1722  CD2 LEU A 126     7758   4986   8086   1336   2159     117
ATOM   1723  H   LEU A 126      24.108  21.517   1.646  1.00  0.00
H
ATOM   1724  HA  LEU A 126      22.583  23.122   3.490  1.00  0.00
H
ATOM   1725  HB3 LEU A 126      24.181  22.178   5.200  1.00  0.00
H
ATOM   1726  HB2 LEU A 126      25.015  21.373   3.958  1.00  0.00
H
ATOM   1727  HG  LEU A 126      22.846  20.157   3.359  1.00  0.00
H
ATOM   1728 HD11 LEU A 126      23.038  18.559   5.219  1.00  0.00
H
ATOM   1729 HD12 LEU A 126      24.628  19.074   4.606  1.00  0.00
H
ATOM   1730 HD13 LEU A 126      24.001  19.748   6.129  1.00  0.00
H
ATOM   1731 HD21 LEU A 126      21.102  20.034   5.097  1.00  0.00
H
ATOM   1732 HD22 LEU A 126      21.931  21.315   6.013  1.00  0.00
H
ATOM   1733 HD23 LEU A 126      21.242  21.670   4.412  1.00  0.00
H
ATOM   1734  N   LEU A 127      25.574  24.171   2.576  1.00 56.60
N
ANISOU 1734  N   LEU A 127     8479   3545   9481   1129   3990      95
ATOM   1735  CA  LEU A 127      26.436  25.361   2.580  1.00 59.39
C
ANISOU 1735  CA  LEU A 127     8818   3445  10301   1031   4432      -7
ATOM   1736  C   LEU A 127      25.658  26.554   2.013  1.00 63.04
C
ANISOU 1736  C   LEU A 127     9740   3752  10459   1244   4649     185
ATOM   1737  O   LEU A 127      25.764  27.658   2.557  1.00 62.73
O
ANISOU 1737  O   LEU A 127     9633   3526  10675   1216   4753      89
ATOM   1738  CB  LEU A 127      27.760  25.134   1.803  1.00 62.43
C
ANISOU 1738  CB  LEU A 127     9180   3421  11119    868   4964     -62
ATOM   1739  CG  LEU A 127      28.674  26.362   1.590  1.00 71.48
C
ANISOU 1739  CG  LEU A 127    10293   4024  12842    742   5521    -184
ATOM   1740  CD1 LEU A 127      29.178  26.946   2.914  1.00 72.19
C
ANISOU 1740  CD1 LEU A 127     9803   4072  13554    545   5261    -524
ATOM   1741  CD2 LEU A 127      29.831  26.037   0.678  1.00 76.28
C
ANISOU 1741  CD2 LEU A 127    11044   4194  13744    639   6180    -160
ATOM   1742  H   LEU A 127      25.824  23.410   1.961  1.00  0.00
H
```

```
ATOM   1743  HA  LEU A 127      26.690  25.588   3.616  1.00  0.00
H
ATOM   1744  HB3 LEU A 127      27.535  24.683   0.837  1.00  0.00
H
ATOM   1745  HB2 LEU A 127      28.330  24.343   2.291  1.00  0.00
H
ATOM   1746  HG  LEU A 127      28.078  27.132   1.100  1.00  0.00
H
ATOM   1747 HD11 LEU A 127      29.816  27.807   2.713  1.00  0.00
H
ATOM   1748 HD12 LEU A 127      28.328  27.258   3.521  1.00  0.00
H
ATOM   1749 HD13 LEU A 127      29.749  26.189   3.452  1.00  0.00
H
ATOM   1750 HD21 LEU A 127      30.453  26.923   0.550  1.00  0.00
H
ATOM   1751 HD22 LEU A 127      30.426  25.236   1.116  1.00  0.00
H
ATOM   1752 HD23 LEU A 127      29.451  25.717  -0.292  1.00  0.00
H
ATOM   1753  N   ALA A 128      24.854  26.326   0.947  1.00 59.94
N
ANISOU 1753  N   ALA A 128     9822   3431   9520   1473   4669    443
ATOM   1754  CA  ALA A 128      24.014  27.373   0.347  1.00 62.76
C
ANISOU 1754  CA  ALA A 128    10670   3650   9527   1733   4782    643
ATOM   1755  C   ALA A 128      22.949  27.847   1.347  1.00 65.05
C
ANISOU 1755  C   ALA A 128    10770   4250   9697   1843   4323    598
ATOM   1756  O   ALA A 128      22.639  29.033   1.382  1.00 66.03
O
ANISOU 1756  O   ALA A 128    11091   4189   9806   1970   4448    649
ATOM   1757  CB  ALA A 128      23.361  26.867  -0.931  1.00 64.57
C
ANISOU 1757  CB  ALA A 128    11434   3906   9193   1971   4769    889
ATOM   1758  H   ALA A 128      24.815  25.406   0.532  1.00  0.00
H
ATOM   1759  HA  ALA A 128      24.650  28.221   0.095  1.00  0.00
H
ATOM   1760  HB1 ALA A 128      22.743  27.656  -1.361  1.00  0.00
H
ATOM   1761  HB2 ALA A 128      24.133  26.581  -1.645  1.00  0.00
H
ATOM   1762  HB3 ALA A 128      22.738  26.002  -0.704  1.00  0.00
H
ATOM   1763  N   LEU A 129      22.443  26.927   2.199  1.00 59.30
N
ANISOU 1763  N   LEU A 129     9666   3957   8907   1787   3837    495
ATOM   1764  CA  LEU A 129      21.476  27.228   3.255  1.00 57.53
C
ANISOU 1764  CA  LEU A 129     9226   4025   8607   1853   3447    420
ATOM   1765  C   LEU A 129      22.139  28.080   4.345  1.00 60.49
C
ANISOU 1765  C   LEU A 129     9343   4230   9409   1683   3547    206
ATOM   1766  O   LEU A 129      21.563  29.085   4.768  1.00 59.23
O
ANISOU 1766  O   LEU A 129     9245   4027   9234   1789   3532    197
```

```
ATOM   1767  CB  LEU A 129      20.938  25.933   3.879  1.00 54.93
C
ANISOU 1767  CB  LEU A 129     8596   4127   8147   1790   3012    352
ATOM   1768  CG  LEU A 129      19.420  25.760   4.009  1.00 59.36
C
ANISOU 1768  CG  LEU A 129     9172   5017   8366   1991   2648    431
ATOM   1769  CD1 LEU A 129      19.101  24.514   4.811  1.00 57.67
C
ANISOU 1769  CD1 LEU A 129     8674   5158   8081   1882   2324    356
ATOM   1770  CD2 LEU A 129      18.737  26.968   4.649  1.00 62.36
C
ANISOU 1770  CD2 LEU A 129     9484   5407   8804   2073   2583    365
ATOM   1771  H   LEU A 129      22.731  25.961   2.128  1.00  0.00
H
ATOM   1772  HA  LEU A 129      20.644  27.787   2.825  1.00  0.00
H
ATOM   1773  HB3 LEU A 129      21.398  25.791   4.857  1.00  0.00
H
ATOM   1774  HB2 LEU A 129      21.349  25.080   3.339  1.00  0.00
H
ATOM   1775  HG  LEU A 129      19.008  25.631   3.008  1.00  0.00
H
ATOM   1776 HD11 LEU A 129      18.020  24.403   4.896  1.00  0.00
H
ATOM   1777 HD12 LEU A 129      19.517  23.641   4.308  1.00  0.00
H
ATOM   1778 HD13 LEU A 129      19.536  24.601   5.807  1.00  0.00
H
ATOM   1779 HD21 LEU A 129      17.664  26.787   4.714  1.00  0.00
H
ATOM   1780 HD22 LEU A 129      19.140  27.126   5.649  1.00  0.00
H
ATOM   1781 HD23 LEU A 129      18.919  27.854   4.040  1.00  0.00
H
ATOM   1782  N   LYS A 130      23.345  27.668   4.795  1.00 57.33
N
ANISOU 1782  N   LYS A 130     8652   3717   9415   1431   3624     16
ATOM   1783  CA  LYS A 130      24.164  28.354   5.799  1.00 58.26
C
ANISOU 1783  CA  LYS A 130     8495   3633  10009   1251   3664   -240
ATOM   1784  C   LYS A 130      24.432  29.788   5.353  1.00 64.60
C
ANISOU 1784  C   LYS A 130     9520   4013  11014   1292   4108   -208
ATOM   1785  O   LYS A 130      24.208  30.721   6.129  1.00 64.72
O
ANISOU 1785  O   LYS A 130     9477   3970  11143   1300   4046   -323
ATOM   1786  CB  LYS A 130      25.499  27.604   5.985  1.00 61.17
C
ANISOU 1786  CB  LYS A 130     8533   3875  10833   1006   3691   -438
ATOM   1787  CG  LYS A 130      25.614  26.800   7.266  0.00 73.78
C
ANISOU 1787  CG  LYS A 130     9800   5777  12457    904   3173   -631
ATOM   1788  CD  LYS A 130      26.989  26.151   7.360  0.00 85.19
C
ANISOU 1788  CD  LYS A 130    10901   7024  14443    688   3176   -856
ATOM   1789  CE  LYS A 130      27.142  25.274   8.575  0.00 94.87
C
```

```
ANISOU 1789  CE  LYS A 130    11867   8505  15674    615   2624  -1046
ATOM   1790  NZ  LYS A 130      28.530  24.761   8.709  0.00105.80
N1+
ANISOU 1790  NZ  LYS A 130    12887   9645  17668    429   2571  -1302
ATOM   1791  H   LYS A 130      23.755  26.818   4.435  1.00  0.00
H
ATOM   1792  HA  LYS A 130      23.628  28.369   6.748  1.00  0.00
H
ATOM   1793  HB3 LYS A 130      26.323  28.316   5.931  1.00  0.00
H
ATOM   1794  HB2 LYS A 130      25.665  26.947   5.131  1.00  0.00
H
ATOM   1795  HG3 LYS A 130      24.846  26.027   7.279  1.00  0.00
H
ATOM   1796  HG2 LYS A 130      25.464  27.458   8.122  1.00  0.00
H
ATOM   1797  HD3 LYS A 130      27.755  26.926   7.374  1.00  0.00
H
ATOM   1798  HD2 LYS A 130      27.173  25.561   6.462  1.00  0.00
H
ATOM   1799  HE3 LYS A 130      26.450  24.435   8.506  1.00  0.00
H
ATOM   1800  HE2 LYS A 130      26.877  25.841   9.467  1.00  0.00
H
ATOM   1801  HZ1 LYS A 130      28.622  23.898   8.192  1.00  0.00
H
ATOM   1802  HZ2 LYS A 130      29.178  25.443   8.342  1.00  0.00
H
ATOM   1803  HZ3 LYS A 130      28.735  24.593   9.684  1.00  0.00
H
ATOM   1804  N   ARG A 131      24.852  29.965   4.079  1.00 62.76
N
ANISOU 1804  N   ARG A 131     9594   3465  10788   1330   4579    -43
ATOM   1805  CA  ARG A 131      25.133  31.279   3.488  1.00 66.10
C
ANISOU 1805  CA  ARG A 131    10322   3421  11374   1378   5094     27
ATOM   1806  C   ARG A 131      23.894  32.183   3.452  1.00 70.23
C
ANISOU 1806  C   ARG A 131    11171   4002  11510   1647   5000    193
ATOM   1807  O   ARG A 131      24.009  33.375   3.735  1.00 70.86
O
ANISOU 1807  O   ARG A 131    11317   3792  11814   1648   5235    139
ATOM   1808  CB  ARG A 131      25.753  31.134   2.092  1.00 69.10
C
ANISOU 1808  CB  ARG A 131    11049   3440  11764   1376   5647    187
ATOM   1809  CG  ARG A 131      27.224  30.745   2.150  1.00 81.95
C
ANISOU 1809  CG  ARG A 131    12308   4781  14048   1073   5956    -44
ATOM   1810  CD  ARG A 131      27.796  30.458   0.782  1.00 96.67
C
ANISOU 1810  CD  ARG A 131    14537   6286  15908   1066   6557    113
ATOM   1811  NE  ARG A 131      29.227  30.159   0.857  1.00108.02
N
ANISOU 1811  NE  ARG A 131    15555   7416  18073    771   6888   -136
ATOM   1812  CZ  ARG A 131      29.930  29.590  -0.117  1.00124.84
C
ANISOU 1812  CZ  ARG A 131    17831   9301  20301    701   7346    -76
```

```
ATOM   1813  NH1 ARG A 131      29.339  29.235  -1.252  1.00112.20
N
ANISOU 1813  NH1 ARG A 131    16834   7743  18053    909   7490    227
ATOM   1814  NH2 ARG A 131      31.226  29.359   0.039  1.00114.14
N1+
ANISOU 1814  NH2 ARG A 131    16014   7644  19711    431   7639   -339
ATOM   1815  H   ARG A 131      24.989  29.166   3.477  1.00  0.00
H
ATOM   1816  HA  ARG A 131      25.873  31.768   4.122  1.00  0.00
H
ATOM   1817  HB3 ARG A 131      25.648  32.073   1.549  1.00  0.00
H
ATOM   1818  HB2 ARG A 131      25.201  30.383   1.527  1.00  0.00
H
ATOM   1819  HG3 ARG A 131      27.342  29.866   2.784  1.00  0.00
H
ATOM   1820  HG2 ARG A 131      27.793  31.547   2.620  1.00  0.00
H
ATOM   1821  HD3 ARG A 131      27.639  31.321   0.135  1.00  0.00
H
ATOM   1822  HD2 ARG A 131      27.271  29.611   0.340  1.00  0.00
H
ATOM   1823  HE  ARG A 131      29.680  30.416   1.723  1.00  0.00
H
ATOM   1824 HH12 ARG A 131      29.878  28.803  -1.989  1.00  0.00
H
ATOM   1825 HH11 ARG A 131      28.350  29.397  -1.379  1.00  0.00
H
ATOM   1826 HH22 ARG A 131      31.755  28.926  -0.705  1.00  0.00
H
ATOM   1827 HH21 ARG A 131      31.684  29.616   0.902  1.00  0.00
H
ATOM   1828  N   SER A 132      22.712  31.610   3.148  1.00 65.99
N
ANISOU 1828  N   SER A 132    10797   3829  10446   1873   4638    368
ATOM   1829  CA  SER A 132      21.449  32.360   3.102  1.00 66.48
C
ANISOU 1829  CA  SER A 132    11112   3965  10184   2156   4478    507
ATOM   1830  C   SER A 132      20.957  32.781   4.492  1.00 69.64
C
ANISOU 1830  C   SER A 132    11167   4577  10716   2117   4175    315
ATOM   1831  O   SER A 132      20.095  33.648   4.586  1.00 69.62
O
ANISOU 1831  O   SER A 132    11317   4556  10579   2320   4122    381
ATOM   1832  CB  SER A 132      20.370  31.554   2.385  1.00 69.72
C
ANISOU 1832  CB  SER A 132    11752   4655  10082   2405   4166    711
ATOM   1833  OG  SER A 132      19.867  30.506   3.198  1.00 77.03
O
ANISOU 1833  OG  SER A 132    12293   6037  10938   2326   3723    607
ATOM   1834  H   SER A 132      22.668  30.623   2.939  1.00  0.00
H
ATOM   1835  HA  SER A 132      21.624  33.267   2.524  1.00  0.00
H
ATOM   1836  HB3 SER A 132      20.781  31.135   1.466  1.00  0.00
H
```

```
ATOM   1837  HB2 SER A 132      19.552  32.216   2.100  1.00  0.00
H
ATOM   1838  HG  SER A 132      20.582  29.910   3.432  1.00  0.00
H
ATOM   1839  N   MET A 133      21.497  32.172   5.561  1.00 65.75
N
ANISOU 1839  N   MET A 133    10251   4254  10477   1873   3977     74
ATOM   1840  CA  MET A 133      21.126  32.486   6.943  1.00 65.48
C
ANISOU 1840  CA  MET A 133     9965   4387  10528   1823   3706   -126
ATOM   1841  C   MET A 133      21.851  33.720   7.490  1.00 73.77
C
ANISOU 1841  C   MET A 133    10963   5072  11994   1701   3949   -314
ATOM   1842  O   MET A 133      21.546  34.163   8.598  1.00 73.81
O
ANISOU 1842  O   MET A 133    10841   5150  12053   1679   3765   -486
ATOM   1843  CB  MET A 133      21.307  31.262   7.855  1.00 65.58
C
ANISOU 1843  CB  MET A 133     9650   4735  10532   1660   3331   -287
ATOM   1844  CG  MET A 133      20.213  30.231   7.693  1.00 67.24
C
ANISOU 1844  CG  MET A 133     9869   5347  10333   1802   3031   -151
ATOM   1845  SD  MET A 133      20.640  28.667   8.489  1.00 69.49
S
ANISOU 1845  SD  MET A 133     9858   5939  10605   1602   2697   -285
ATOM   1846  CE  MET A 133      19.128  27.785   8.292  1.00 64.49
C
ANISOU 1846  CE  MET A 133     9247   5713   9544   1787   2423   -141
ATOM   1847  H   MET A 133      22.198  31.456   5.434  1.00  0.00
H
ATOM   1848  HA  MET A 133      20.062  32.721   6.938  1.00  0.00
H
ATOM   1849  HB3 MET A 133      21.346  31.588   8.894  1.00  0.00
H
ATOM   1850  HB2 MET A 133      22.273  30.800   7.653  1.00  0.00
H
ATOM   1851  HG3 MET A 133      20.030  30.060   6.632  1.00  0.00
H
ATOM   1852  HG2 MET A 133      19.287  30.615   8.121  1.00  0.00
H
ATOM   1853  HE1 MET A 133      19.225  26.793   8.733  1.00  0.00
H
ATOM   1854  HE2 MET A 133      18.324  28.326   8.790  1.00  0.00
H
ATOM   1855  HE3 MET A 133      18.898  27.689   7.231  1.00  0.00
H
ATOM   1856  N   SER A 134      22.767  34.305   6.696  1.00 73.77
N
ANISOU 1856  N   SER A 134    11088   4652  12291   1624   4392   -289
ATOM   1857  CA  SER A 134      23.511  35.506   7.076  1.00102.68
C
ANISOU 1857  CA  SER A 134    14690   7899  16426   1491   4686   -475
ATOM   1858  C   SER A 134      22.649  36.771   6.965  1.00118.30
C
ANISOU 1858  C   SER A 134    16962   9723  18264   1702   4837   -365
ATOM   1859  O   SER A 134      21.603  36.770   6.312  1.00 68.62
O
```

```
ANISOU 1859  O   SER A 134    10981   3537  11553   1969   4816   -105
ATOM   1860  CB  SER A 134      24.774  35.647   6.233  1.00108.94
C
ANISOU 1860  CB  SER A 134    15519   8253  17621   1336   5189   -477
ATOM   1861  OG  SER A 134      24.471  35.811   4.857  1.00119.02
O
ANISOU 1861  OG  SER A 134    17273   9366  18583   1530   5548   -156
ATOM   1862  H   SER A 134      22.970  33.915   5.787  1.00  0.00
H
ATOM   1863  HA  SER A 134      23.814  35.397   8.117  1.00  0.00
H
ATOM   1864  HB3 SER A 134      25.398  34.763   6.364  1.00  0.00
H
ATOM   1865  HB2 SER A 134      25.349  36.504   6.582  1.00  0.00
H
ATOM   1866  HG  SER A 134      24.308  34.952   4.460  1.00  0.00
H
ATOM   1867  N   NMA A 134A     23.059  37.921   7.609  1.00  0.00
N
ATOM   1868  CA  NMA A 134A     22.257  39.132   7.517  1.00  0.00
C
ATOM   1869  H   NMA A 134A     23.915  37.921   8.143  1.00  0.00
H
ATOM   1870 1HA  NMA A 134A     21.363  38.923   6.889  1.00  0.00
H
ATOM   1871 2HA  NMA A 134A     21.936  39.449   8.535  1.00  0.00
H
ATOM   1872 3HA  NMA A 134A     22.859  39.947   7.057  1.00  0.00
H
CONECT    1    4    5    6
CONECT    4    1
CONECT    5    1
CONECT    6    1
ENDMDL
END
```

9

```
REMARK   4      COMPLIES WITH FORMAT V. 3.0, 1-DEC-2006
REMARK 888
REMARK 888 WRITTEN BY MAESTRO (A PRODUCT OF SCHRODINGER, LLC)
TITLE     BI-7273
MODEL        1
HETATM    1  C1  CP10  900       6.295  -2.788   5.722  1.00  0.00
C
HETATM    2  C2  CP10  900       5.365  -3.516   4.963  1.00  0.00
C
HETATM    3  C3  CP10  900       5.806  -4.486   4.047  1.00  0.00
C
HETATM    4  C4  CP10  900       7.184  -4.702   3.897  1.00  0.00
C
HETATM    5  C5  CP10  900       8.119  -4.035   4.689  1.00  0.00
C
HETATM    6  C6  CP10  900       7.656  -3.075   5.584  1.00  0.00
C
```

```
HETATM    7  O1  CP10  900        5.827  -1.808   6.573  1.00  0.00
O
HETATM    8  C7  CP10  900        6.737  -1.190   7.489  1.00  0.00
C
HETATM    9  O2  CP10  900        4.846  -5.161   3.312  1.00  0.00
O
HETATM   10  C8  CP10  900        5.243  -6.144   2.363  1.00  0.00
C
HETATM   11  C9  CP10  900        3.867  -3.265   5.144  1.00  0.00
C
HETATM   12  N1  CP10  900        3.096  -3.019   3.828  1.00  0.00
N1+
HETATM   13  C10 CP10  900        1.568  -3.046   4.032  1.00  0.00
C
HETATM   14  C11 CP10  900        3.539  -1.734   3.103  1.00  0.00
C
HETATM   15  C12 CP10  900        9.577  -4.285   4.584  1.00  0.00
C
HETATM   16  C13 CP10  900       10.435  -3.242   4.574  1.00  0.00
C
HETATM   17  N2  CP10  900       11.804  -3.277   4.584  1.00  0.00
N
HETATM   18  C14 CP10  900       12.482  -4.463   4.744  1.00  0.00
C
HETATM   19  C15 CP10  900       11.609  -5.708   4.625  1.00  0.00
C
HETATM   20  C16 CP10  900       10.199  -5.633   4.593  1.00  0.00
C
HETATM   21  C17 CP10  900       12.218  -6.965   4.569  1.00  0.00
C
HETATM   22  N3  CP10  900       11.510  -8.120   4.490  1.00  0.00
N
HETATM   23  C18 CP10  900       10.157  -8.043   4.511  1.00  0.00
C
HETATM   24  C19 CP10  900        9.471  -6.833   4.572  1.00  0.00
C
HETATM   25  O3  CP10  900       13.694  -4.523   4.972  1.00  0.00
O
HETATM   26  C20 CP10  900       12.588  -2.036   4.568  1.00  0.00
C
HETATM   27  H1  CP10  900        7.545  -5.394   3.158  1.00  0.00
H
HETATM   28  H2  CP10  900        8.384  -2.539   6.173  1.00  0.00
H
HETATM   29  H3  CP10  900        6.219  -0.528   8.171  1.00  0.00
H
HETATM   30  H4  CP10  900        7.466  -0.571   6.971  1.00  0.00
H
HETATM   31  H5  CP10  900        7.255  -1.924   8.108  1.00  0.00
H
HETATM   32  H6  CP10  900        5.823  -6.945   2.824  1.00  0.00
H
HETATM   33  H7  CP10  900        5.819  -5.706   1.548  1.00  0.00
H
HETATM   34  H8  CP10  900        4.355  -6.598   1.927  1.00  0.00
H
```

```
HETATM   35  H9  CP10  900       3.638  -2.400   5.772  1.00  0.00
H
HETATM   36  H10 CP10  900       3.405  -4.148   5.589  1.00  0.00
H
HETATM   37  H11 CP10  900       1.311  -4.013   4.469  1.00  0.00
H
HETATM   38  H12 CP10  900       1.103  -2.931   3.051  1.00  0.00
H
HETATM   39  H13 CP10  900       1.300  -2.222   4.695  1.00  0.00
H
HETATM   40  H14 CP10  900       4.619  -1.771   2.968  1.00  0.00
H
HETATM   41  H15 CP10  900       3.253  -0.878   3.719  1.00  0.00
H
HETATM   42  H16 CP10  900       3.033  -1.722   2.136  1.00  0.00
H
HETATM   43  H17 CP10  900      10.087  -2.217   4.575  1.00  0.00
H
HETATM   44  H18 CP10  900      13.293  -7.075   4.573  1.00  0.00
H
HETATM   45  H19 CP10  900       9.620  -8.980   4.495  1.00  0.00
H
HETATM   46  H20 CP10  900       8.394  -6.857   4.604  1.00  0.00
H
HETATM   47  H21 CP10  900      13.659  -2.185   4.413  1.00  0.00
H
HETATM   48  H22 CP10  900      12.459  -1.504   5.510  1.00  0.00
H
HETATM   49  H23 CP10  900      12.236  -1.383   3.768  1.00  0.00
H
HETATM   50  H24 CP10  900       3.347  -3.801   3.233  1.00  0.00
H
CONECT    1    2    6    7
CONECT    2    1    3   11
CONECT    3    2    4    9
CONECT    4    3    5   27
CONECT    5    4    6   15
CONECT    6    1    5   28
CONECT    7    1    8
CONECT    8    7   29   30   31
CONECT    9    3   10
CONECT   10    9   32   33   34
CONECT   11    2   12   35   36
CONECT   12   11   13   14   50
CONECT   13   12   37   38   39
CONECT   14   12   40   41   42
CONECT   15    5   20   16
CONECT   16   15   17   43
CONECT   17   16   18   26
CONECT   18   17   19   25
CONECT   19   18   20   21
CONECT   20   15   19   24
CONECT   21   19   22   44
CONECT   22   21   23
CONECT   23   22   24   45
CONECT   24   20   23   46
CONECT   25   18
```

```
CONECT   26   17   47   48   49
CONECT   27    4
CONECT   28    6
CONECT   29    8
CONECT   30    8
CONECT   31    8
CONECT   32   10
CONECT   33   10
CONECT   34   10
CONECT   35   11
CONECT   36   11
CONECT   37   13
CONECT   38   13
CONECT   39   13
CONECT   40   14
CONECT   41   14
CONECT   42   14
CONECT   43   16
CONECT   44   21
CONECT   45   23
CONECT   46   24
CONECT   47   26
CONECT   48   26
CONECT   49   26
CONECT   50   12
ENDMDL
END
```

10a

```
REMARK   4      COMPLIES WITH FORMAT V. 3.0, 1-DEC-2006
REMARK 888
REMARK 888 WRITTEN BY MAESTRO (A PRODUCT OF SCHRODINGER, LLC)
TITLE     LP-99
MODEL        1
HETATM    1  C1  CP11  900       8.219  -3.313   6.941  1.00  0.00
C
HETATM    2  C2  CP11  900       9.079  -4.318   6.171  1.00  0.00
C
HETATM    3  C3  CP11  900       8.872  -4.227   4.663  1.00  0.00
C
HETATM    4  C4  CP11  900       7.355  -4.323   4.276  1.00  0.00
C
HETATM    5  N1  CP11  900       6.434  -3.641   5.254  1.00  0.00
N
HETATM    6  C5  CP11  900       6.746  -3.292   6.541  1.00  0.00
C
HETATM    7  C6  CP11  900       5.083  -3.485   4.868  1.00  0.00
C
HETATM    8  C7  CP11  900       6.924  -5.732   3.809  1.00  0.00
C
HETATM    9  N2  CP11  900       9.395  -2.933   4.171  1.00  0.00
N
HETATM   10  C8  CP11  900       5.812  -5.863   2.968  1.00  0.00
C
```

```
HETATM   11  C9  CP11  900       5.370  -7.120   2.562  1.00  0.00
C
HETATM   12  C10 CP11  900       6.056  -8.259   2.973  1.00  0.00
C
HETATM   13  C11 CP11  900       7.190  -8.148   3.771  1.00  0.00
C
HETATM   14  C12 CP11  900       7.631  -6.890   4.171  1.00  0.00
C
HETATM   15 CL1  CP11  900       5.509  -9.816   2.474  1.00  0.00
Cl
HETATM   16  S1  CP11  900      11.078  -2.457   4.433  1.00  0.00
S
HETATM   17  C13 CP11  900      12.082  -4.050   4.390  1.00  0.00
C
HETATM   18  C14 CP11  900      13.570  -3.872   4.788  1.00  0.00
C
HETATM   19  C15 CP11  900      13.955  -2.633   5.622  1.00  0.00
C
HETATM   20  C16 CP11  900      14.009  -5.154   5.517  1.00  0.00
C
HETATM   21  O1  CP11  900      11.154  -1.877   5.771  1.00  0.00
O
HETATM   22  O2  CP11  900      11.490  -1.646   3.288  1.00  0.00
O
HETATM   23  O3  CP11  900       5.917  -2.933   7.371  1.00  0.00
O
HETATM   24  C17 CP11  900       4.134  -4.457   5.203  1.00  0.00
C
HETATM   25  C18 CP11  900       2.785  -4.304   4.839  1.00  0.00
C
HETATM   26  C19 CP11  900       2.397  -3.137   4.144  1.00  0.00
C
HETATM   27  C20 CP11  900       3.348  -2.157   3.835  1.00  0.00
C
HETATM   28  C21 CP11  900       4.679  -2.330   4.199  1.00  0.00
C
HETATM   29  N3  CP11  900       1.792  -5.289   5.146  1.00  0.00
N
HETATM   30  C22 CP11  900       0.463  -5.203   4.816  1.00  0.00
C
HETATM   31  C23 CP11  900       0.105  -3.988   4.107  1.00  0.00
C
HETATM   32  C24 CP11  900       0.966  -3.002   3.773  1.00  0.00
C
HETATM   33  O4  CP11  900      -0.396  -6.050   5.071  1.00  0.00
O
HETATM   34  C25 CP11  900       0.508  -1.755   3.027  1.00  0.00
C
HETATM   35  C26 CP11  900       2.226  -6.496   5.876  1.00  0.00
C
HETATM   36  H1  CP11  900       8.259  -3.523   8.011  1.00  0.00
H
HETATM   37  H2  CP11  900       8.590  -2.295   6.813  1.00  0.00
H
HETATM   38  H3  CP11  900      10.112  -4.149   6.452  1.00  0.00
H
```

```
HETATM   39  H4  CP11  900       8.875  -5.330   6.503  1.00  0.00
H
HETATM   40  H5  CP11  900       9.418  -5.009   4.135  1.00  0.00
H
HETATM   41  H6  CP11  900       7.261  -3.750   3.358  1.00  0.00
H
HETATM   42  H7  CP11  900       8.797  -2.189   4.547  1.00  0.00
H
HETATM   43  H8  CP11  900       5.273  -4.990   2.632  1.00  0.00
H
HETATM   44  H9  CP11  900       4.500  -7.208   1.927  1.00  0.00
H
HETATM   45  H10 CP11  900       7.720  -9.034   4.091  1.00  0.00
H
HETATM   46  H11 CP11  900       8.499  -6.847   4.809  1.00  0.00
H
HETATM   47  H12 CP11  900      11.584  -4.746   5.057  1.00  0.00
H
HETATM   48  H13 CP11  900      11.979  -4.466   3.388  1.00  0.00
H
HETATM   49  H14 CP11  900      14.128  -3.733   3.862  1.00  0.00
H
HETATM   50  H15 CP11  900      13.751  -1.708   5.082  1.00  0.00
H
HETATM   51  H16 CP11  900      15.019  -2.627   5.853  1.00  0.00
H
HETATM   52  H17 CP11  900      13.428  -2.584   6.571  1.00  0.00
H
HETATM   53  H18 CP11  900      13.819  -6.052   4.929  1.00  0.00
H
HETATM   54  H19 CP11  900      13.436  -5.272   6.441  1.00  0.00
H
HETATM   55  H20 CP11  900      15.070  -5.134   5.771  1.00  0.00
H
HETATM   56  H21 CP11  900       4.474  -5.327   5.743  1.00  0.00
H
HETATM   57  H22 CP11  900       3.073  -1.249   3.319  1.00  0.00
H
HETATM   58  H23 CP11  900       5.396  -1.557   3.973  1.00  0.00
H
HETATM   59  H24 CP11  900      -0.936  -3.887   3.836  1.00  0.00
H
HETATM   60  H25 CP11  900      -0.561  -1.782   2.811  1.00  0.00
H
HETATM   61  H26 CP11  900       1.034  -1.662   2.077  1.00  0.00
H
HETATM   62  H27 CP11  900       0.702  -0.862   3.622  1.00  0.00
H
HETATM   63  H28 CP11  900       1.428  -7.219   6.058  1.00  0.00
H
HETATM   64  H29 CP11  900       2.631  -6.219   6.849  1.00  0.00
H
HETATM   65  H30 CP11  900       3.002  -7.019   5.315  1.00  0.00
H
CONECT    1    2    6   36   37
CONECT    2    1    3   38   39
CONECT    3    2    4    9   40
```

```
CONECT    4    3    5    8   41
CONECT    5    4    6    7
CONECT    6    1    5   23
CONECT    7    5   28   24
CONECT    8    4   14   10
CONECT    9    3   16   42
CONECT   10    8   11   43
CONECT   11   10   12   44
CONECT   12   11   13   15
CONECT   13   12   14   45
CONECT   14    8   13   46
CONECT   15   12
CONECT   16    9   17   21   22
CONECT   17   16   18   47   48
CONECT   18   17   19   20   49
CONECT   19   18   50   51   52
CONECT   20   18   53   54   55
CONECT   21   16
CONECT   22   16
CONECT   23    6
CONECT   24    7   25   56
CONECT   25   24   26   29
CONECT   26   25   27   32
CONECT   27   26   28   57
CONECT   28    7   27   58
CONECT   29   25   30   35
CONECT   30   29   31   33
CONECT   31   30   32   59
CONECT   32   26   31   34
CONECT   33   30
CONECT   34   32   60   61   62
CONECT   35   29   63   64   65
CONECT   36    1
CONECT   37    1
CONECT   38    2
CONECT   39    2
CONECT   40    3
CONECT   41    4
CONECT   42    9
CONECT   43   10
CONECT   44   11
CONECT   45   13
CONECT   46   14
CONECT   47   17
CONECT   48   17
CONECT   49   18
CONECT   50   19
CONECT   51   19
CONECT   52   19
CONECT   53   20
CONECT   54   20
CONECT   55   20
CONECT   56   24
CONECT   57   27
CONECT   58   28
CONECT   59   31
CONECT   60   34
```

```
CONECT   61   34
CONECT   62   34
CONECT   63   35
CONECT   64   35
CONECT   65   35
ENDMDL
END
```

10b

```
REMARK   4      COMPLIES WITH FORMAT V. 3.0, 1-DEC-2006
REMARK 888
REMARK 888 WRITTEN BY MAESTRO (A PRODUCT OF SCHRODINGER, LLC)
TITLE     LP-99
MODEL        1
MODEL        1
HETATM    1  CAK 6B2 A 201      -5.259 -16.888  -1.587  1.00  9.63
C
HETATM    2  CAJ 6B2 A 201      -5.798 -15.463  -1.540  1.00  9.39
C
HETATM    3  CAI 6B2 A 201      -5.013 -14.427  -1.176  1.00  9.13
C
HETATM    4  CAH 6B2 A 201      -5.428 -13.038  -1.140  1.00  9.23
C
HETATM    5  OAL 6B2 A 201      -4.628 -12.167  -0.798  1.00  7.98
O
HETATM    6  NAG 6B2 A 201      -6.729 -12.832  -1.528  1.00  9.53
N
HETATM    7  CAM 6B2 A 201      -7.199 -11.434  -1.544  1.00  9.19
C
HETATM    8  CAD 6B2 A 201      -7.647 -13.855  -1.940  1.00  9.54
C
HETATM    9  CAC 6B2 A 201      -8.966 -13.574  -2.347  1.00  9.59
C
HETATM   10  CAE 6B2 A 201      -7.206 -15.199  -1.940  1.00  9.78
C
HETATM   11  CAF 6B2 A 201      -8.086 -16.226  -2.306  1.00 10.55
C
HETATM   12  CAA 6B2 A 201      -9.381 -15.927  -2.715  1.00 10.76
C
HETATM   13  CAB 6B2 A 201      -9.824 -14.598  -2.767  1.00 10.31
C
HETATM   14  NAN 6B2 A 201     -11.105 -14.280  -3.290  1.00 12.38
N
HETATM   15  CAO 6B2 A 201     -11.387 -14.610  -4.600  1.00 11.80
C
HETATM   16  OAT 6B2 A 201     -10.566 -15.115  -5.367  1.00 13.49
O
HETATM   17  CAP 6B2 A 201     -12.795 -14.280  -5.142  1.00 12.33
C
HETATM   18  CAQ 6B2 A 201     -13.861 -14.008  -4.077  1.00 12.23
C
HETATM   19  CAR 6B2 A 201     -13.338 -13.094  -2.965  1.00 13.52
C
HETATM   20  NBA 6B2 A 201     -12.927 -11.779  -3.523  1.00 16.22
N
```

```
HETATM   21  SBC 6B2 A 201      -14.122 -10.663  -4.178  1.00 22.12
S
HETATM   22  OBE 6B2 A 201      -15.436 -11.312  -4.150  1.00 23.44
O
HETATM   23  OBF 6B2 A 201      -13.567 -10.141  -5.431  1.00 23.38
O
HETATM   24  CBD 6B2 A 201      -14.135  -9.275  -2.895  1.00 25.55
C
HETATM   25  CBG 6B2 A 201      -14.956  -8.048  -3.357  1.00 30.90
C
HETATM   26  CBI 6B2 A 201      -16.460  -8.339  -3.508  1.00 33.24
C
HETATM   27  CBH 6B2 A 201      -14.723  -6.862  -2.405  1.00 31.10
C
HETATM   28  CAS 6B2 A 201      -12.099 -13.750  -2.296  1.00 12.49
C
HETATM   29  CAU 6B2 A 201      -12.565 -14.703  -1.186  1.00 11.81
C
HETATM   30  CAV 6B2 A 201      -12.535 -16.093  -1.347  1.00 12.60
C
HETATM   31  CAW 6B2 A 201      -12.971 -16.927  -0.322  1.00 12.63
C
HETATM   32  CAX 6B2 A 201      -13.455 -16.373   0.860  1.00 13.25
C
HETATM   33 CL   6B2 A 201      -13.988 -17.404   2.135  1.00 18.86
Cl
HETATM   34  CAY 6B2 A 201      -13.502 -14.991   1.026  1.00 13.51
C
HETATM   35  CAZ 6B2 A 201      -13.040 -14.158   0.013  1.00 12.52
C
HETATM   36 HAK1 6B2 A 201       -4.193 -16.922  -1.363  1.00  0.00
H
HETATM   37 HAK2 6B2 A 201       -5.397 -17.319  -2.578  1.00  0.00
H
HETATM   38 HAK3 6B2 A 201       -5.766 -17.517  -0.856  1.00  0.00
H
HETATM   39  HAI 6B2 A 201       -3.986 -14.619  -0.899  1.00  0.00
H
HETATM   40 HAM1 6B2 A 201       -6.500 -10.719  -1.105  1.00  0.00
H
HETATM   41 HAM2 6B2 A 201       -8.131 -11.333  -0.985  1.00  0.00
H
HETATM   42 HAM3 6B2 A 201       -7.367 -11.118  -2.572  1.00  0.00
H
HETATM   43  HAC 6B2 A 201       -9.327 -12.559  -2.393  1.00  0.00
H
HETATM   44  HAF 6B2 A 201       -7.774 -17.260  -2.287  1.00  0.00
H
HETATM   45  HAA 6B2 A 201      -10.027 -16.733  -3.030  1.00  0.00
H
HETATM   46 HAP1 6B2 A 201      -13.112 -15.101  -5.785  1.00  0.00
H
HETATM   47 HAP2 6B2 A 201      -12.682 -13.411  -5.791  1.00  0.00
H
HETATM   48 HAQ1 6B2 A 201      -14.757 -13.612  -4.551  1.00  0.00
H
```

```
HETATM   49 HAQ2 6B2 A 201      -14.173 -14.958  -3.638  1.00  0.00
H
HETATM   50  HAR 6B2 A 201      -14.109 -12.922  -2.211  1.00  0.00
H
HETATM   51 HNBA 6B2 A 201      -12.049 -11.802  -4.051  1.00  0.00
H
HETATM   52 HBD1 6B2 A 201      -14.506  -9.695  -1.961  1.00  0.00
H
HETATM   53 HBD2 6B2 A 201      -13.089  -9.009  -2.739  1.00  0.00
H
HETATM   54  HBG 6B2 A 201      -14.568  -7.749  -4.334  1.00  0.00
H
HETATM   55 HBI1 6B2 A 201      -16.655  -9.066  -4.295  1.00  0.00
H
HETATM   56 HBI2 6B2 A 201      -17.010  -7.436  -3.775  1.00  0.00
H
HETATM   57 HBI3 6B2 A 201      -16.888  -8.726  -2.583  1.00  0.00
H
HETATM   58 HBH1 6B2 A 201      -13.662  -6.615  -2.331  1.00  0.00
H
HETATM   59 HBH2 6B2 A 201      -15.083  -7.081  -1.398  1.00  0.00
H
HETATM   60 HBH3 6B2 A 201      -15.239  -5.968  -2.753  1.00  0.00
H
HETATM   61  HAS 6B2 A 201      -11.577 -12.964  -1.748  1.00  0.00
H
HETATM   62  HAV 6B2 A 201      -12.183 -16.534  -2.267  1.00  0.00
H
HETATM   63  HAW 6B2 A 201      -12.940 -18.000  -0.446  1.00  0.00
H
HETATM   64  HAY 6B2 A 201      -13.874 -14.568   1.948  1.00  0.00
H
HETATM   65  HAZ 6B2 A 201      -13.067 -13.088   0.154  1.00  0.00
H
CONECT    1    2   36   37   38
CONECT    2    1    3   10
CONECT    3    2    4   39
CONECT    4    3    5    6
CONECT    5    4
CONECT    6    4    7    8
CONECT    7    6   40   41   42
CONECT    8    6    9   10
CONECT    9    8   13   43
CONECT   10    2    8   11
CONECT   11   10   12   44
CONECT   12   11   13   45
CONECT   13    9   12   14
CONECT   14   13   15   28
CONECT   15   14   16   17
CONECT   16   15
CONECT   17   15   18   46   47
CONECT   18   17   19   48   49
CONECT   19   18   20   28   50
CONECT   20   19   21   51
CONECT   21   20   22   23   24
CONECT   22   21
CONECT   23   21
```

```
CONECT   24   21   25   52   53
CONECT   25   24   26   27   54
CONECT   26   25   55   56   57
CONECT   27   25   58   59   60
CONECT   28   14   19   29   61
CONECT   29   28   30   35
CONECT   30   29   31   62
CONECT   31   30   32   63
CONECT   32   31   33   34
CONECT   33   32
CONECT   34   32   35   64
CONECT   35   29   34   65
CONECT   36    1
CONECT   37    1
CONECT   38    1
CONECT   39    3
CONECT   40    7
CONECT   41    7
CONECT   42    7
CONECT   43    9
CONECT   44   11
CONECT   45   12
CONECT   46   17
CONECT   47   17
CONECT   48   18
CONECT   49   18
CONECT   50   19
CONECT   51   20
CONECT   52   24
CONECT   53   24
CONECT   54   25
CONECT   55   26
CONECT   56   26
CONECT   57   26
CONECT   58   27
CONECT   59   27
CONECT   60   27
CONECT   61   28
CONECT   62   30
CONECT   63   31
CONECT   64   34
CONECT   65   35
ENDMDL
END
```

11

```
REMARK   4      COMPLIES WITH FORMAT V. 3.0, 1-DEC-2006
REMARK 888
REMARK 888 WRITTEN BY MAESTRO (A PRODUCT OF SCHRODINGER, LLC)
TITLE     compund_64
MODEL        1
HETATM    1  C1  CP12  900       8.973  -9.508   3.241  1.00  0.00
C
HETATM    2  C2  CP12  900       9.816  -9.785   4.489  1.00  0.00
C
```

```
HETATM    3  C3  CP12  900      11.310  -9.613   4.207  1.00  0.00
C
HETATM    4  C4  CP12  900      11.671 -10.642   3.095  1.00  0.00
C
HETATM    5  N1  CP12  900      10.837 -10.456   1.860  1.00  0.00
N
HETATM    6  C5  CP12  900       9.495 -10.134   1.927  1.00  0.00
C
HETATM    7  C6  CP12  900      11.405 -10.883   0.613  1.00  0.00
C
HETATM    8  C7  CP12  900      11.803 -12.061   3.667  1.00  0.00
C
HETATM    9  N2  CP12  900      11.818  -8.247   3.956  1.00  0.00
N
HETATM   10  C8  CP12  900      10.732 -12.963   3.644  1.00  0.00
C
HETATM   11  C9  CP12  900      10.885 -14.243   4.171  1.00  0.00
C
HETATM   12  C10 CP12  900      12.112 -14.629   4.705  1.00  0.00
C
HETATM   13  C11 CP12  900      13.189 -13.746   4.711  1.00  0.00
C
HETATM   14  C12 CP12  900      13.037 -12.468   4.180  1.00  0.00
C
HETATM   15 CL1  CP12  900      12.298 -16.209   5.371  1.00  0.00
Cl
HETATM   16  O1  CP12  900       8.720 -10.246   0.975  1.00  0.00
O
HETATM   17  C13 CP12  900      11.247 -10.100  -0.542  1.00  0.00
C
HETATM   18  C14 CP12  900      11.804 -10.458  -1.780  1.00  0.00
C
HETATM   19  C15 CP12  900      12.528 -11.663  -1.868  1.00  0.00
C
HETATM   20  C16 CP12  900      12.694 -12.455  -0.727  1.00  0.00
C
HETATM   21  C17 CP12  900      12.148 -12.068   0.495  1.00  0.00
C
HETATM   22  N3  CP12  900      11.662  -9.644  -2.951  1.00  0.00
N
HETATM   23  C18 CP12  900      12.219  -9.916  -4.175  1.00  0.00
C
HETATM   24  C19 CP12  900      12.921 -11.186  -4.236  1.00  0.00
C
HETATM   25  C20 CP12  900      13.092 -12.029  -3.193  1.00  0.00
C
HETATM   26  O2  CP12  900      12.167  -9.176  -5.160  1.00  0.00
O
HETATM   27  C21 CP12  900      13.854 -13.340  -3.339  1.00  0.00
C
HETATM   28  C22 CP12  900      10.886  -8.392  -2.831  1.00  0.00
C
HETATM   29  C23 CP12  900      11.407  -7.092   4.518  1.00  0.00
C
HETATM   30  N4  CP12  900      12.396  -6.183   4.545  1.00  0.00
N
```

```
HETATM   31  C24 CP12  900      12.282  -4.798   4.777  1.00  0.00
C
HETATM   32  C25 CP12  900      11.112  -4.071   4.510  1.00  0.00
C
HETATM   33  C26 CP12  900      11.098  -2.689   4.668  1.00  0.00
C
HETATM   34  C27 CP12  900      12.249  -2.030   5.087  1.00  0.00
C
HETATM   35  C28 CP12  900      13.411  -2.743   5.371  1.00  0.00
C
HETATM   36  C29 CP12  900      13.421  -4.128   5.227  1.00  0.00
C
HETATM   37  O3  CP12  900      10.276  -6.893   4.962  1.00  0.00
O
HETATM   38  H1  CP12  900       7.949  -9.848   3.407  1.00  0.00
H
HETATM   39  H2  CP12  900       8.912  -8.434   3.074  1.00  0.00
H
HETATM   40  H3  CP12  900       9.494  -9.142   5.308  1.00  0.00
H
HETATM   41  H4  CP12  900       9.608 -10.790   4.859  1.00  0.00
H
HETATM   42  H5  CP12  900      11.820  -9.901   5.127  1.00  0.00
H
HETATM   43  H6  CP12  900      12.687 -10.397   2.779  1.00  0.00
H
HETATM   44  H7  CP12  900      12.704  -8.210   3.469  1.00  0.00
H
HETATM   45  H8  CP12  900       9.777 -12.674   3.233  1.00  0.00
H
HETATM   46  H9  CP12  900      10.054 -14.933   4.163  1.00  0.00
H
HETATM   47  H10 CP12  900      14.137 -14.048   5.131  1.00  0.00
H
HETATM   48  H11 CP12  900      13.874 -11.786   4.204  1.00  0.00
H
HETATM   49  H12 CP12  900      10.669  -9.197  -0.470  1.00  0.00
H
HETATM   50  H13 CP12  900      13.247 -13.383  -0.770  1.00  0.00
H
HETATM   51  H14 CP12  900      12.301 -12.730   1.329  1.00  0.00
H
HETATM   52  H15 CP12  900      13.342 -11.449  -5.196  1.00  0.00
H
HETATM   53  H16 CP12  900      14.209 -13.491  -4.359  1.00  0.00
H
HETATM   54  H17 CP12  900      13.211 -14.184  -3.087  1.00  0.00
H
HETATM   55  H18 CP12  900      14.726 -13.355  -2.682  1.00  0.00
H
HETATM   56  H19 CP12  900      10.798  -7.825  -3.759  1.00  0.00
H
HETATM   57  H20 CP12  900      11.343  -7.733  -2.093  1.00  0.00
H
HETATM   58  H21 CP12  900       9.868  -8.614  -2.507  1.00  0.00
H
```

```
HETATM   59  H22 CP12  900       13.316  -6.522   4.307  1.00  0.00
H
HETATM   60  H23 CP12  900       10.218  -4.557   4.147  1.00  0.00
H
HETATM   61  H24 CP12  900       10.198  -2.132   4.460  1.00  0.00
H
HETATM   62  H25 CP12  900       12.231  -0.955   5.208  1.00  0.00
H
HETATM   63  H26 CP12  900       14.291  -2.222   5.714  1.00  0.00
H
HETATM   64  H27 CP12  900       14.322  -4.673   5.462  1.00  0.00
H
CONECT    1    2    6   38   39
CONECT    2    1    3   40   41
CONECT    3    2    4    9   42
CONECT    4    3    5    8   43
CONECT    5    4    6    7
CONECT    6    1    5   16
CONECT    7    5   21   17
CONECT    8    4   14   10
CONECT    9    3   29   44
CONECT   10    8   11   45
CONECT   11   10   12   46
CONECT   12   11   13   15
CONECT   13   12   14   47
CONECT   14    8   13   48
CONECT   15   12
CONECT   16    6
CONECT   17    7   18   49
CONECT   18   17   19   22
CONECT   19   18   20   25
CONECT   20   19   21   50
CONECT   21    7   20   51
CONECT   22   18   23   28
CONECT   23   22   24   26
CONECT   24   23   25   52
CONECT   25   19   24   27
CONECT   26   23
CONECT   27   25   53   54   55
CONECT   28   22   56   57   58
CONECT   29    9   30   37
CONECT   30   29   31   59
CONECT   31   30   36   32
CONECT   32   31   33   60
CONECT   33   32   34   61
CONECT   34   33   35   62
CONECT   35   34   36   63
CONECT   36   31   35   64
CONECT   37   29
CONECT   38    1
CONECT   39    1
CONECT   40    2
CONECT   41    2
CONECT   42    3
CONECT   43    4
CONECT   44    9
CONECT   45   10
```

```
CONECT   46   11
CONECT   47   13
CONECT   48   14
CONECT   49   17
CONECT   50   20
CONECT   51   21
CONECT   52   24
CONECT   53   27
CONECT   54   27
CONECT   55   27
CONECT   56   28
CONECT   57   28
CONECT   58   28
CONECT   59   30
CONECT   60   32
CONECT   61   33
CONECT   62   34
CONECT   63   35
CONECT   64   36
ENDMDL
END
```

12

```
REMARK   4      COMPLIES WITH FORMAT V. 3.0, 1-DEC-2006
REMARK 888
REMARK 888 WRITTEN BY MAESTRO (A PRODUCT OF SCHRODINGER, LLC)
TITLE     PF-47736
MODEL        1
HETATM    1  N1  CP13  900      10.946  -2.921   4.966  1.00  0.00
N
HETATM    2  N2  CP13  900      12.098  -3.675   4.943  1.00  0.00
N
HETATM    3  C1  CP13  900      12.408  -4.996   5.046  1.00  0.00
C
HETATM    4  C2  CP13  900      11.377  -6.056   4.708  1.00  0.00
C
HETATM    5  C3  CP13  900      10.003  -5.837   4.562  1.00  0.00
C
HETATM    6  C4  CP13  900       9.218  -4.658   4.639  1.00  0.00
C
HETATM    7  C5  CP13  900       9.743  -3.332   4.856  1.00  0.00
C
HETATM    8  O1  CP13  900      13.550  -5.326   5.372  1.00  0.00
O
HETATM    9  C6  CP13  900      11.832  -7.371   4.534  1.00  0.00
C
HETATM   10  C7  CP13  900      10.967  -8.424   4.232  1.00  0.00
C
HETATM   11  C8  CP13  900       9.589  -8.226   4.096  1.00  0.00
C
HETATM   12  C9  CP13  900       9.130  -6.917   4.256  1.00  0.00
C
HETATM   13  C10 CP13  900       7.889  -5.027   4.442  1.00  0.00
C
```

```
HETATM   14  N3  CP13  900       7.867  -6.376   4.172  1.00  0.00
N
HETATM   15  C11 CP13  900       6.643  -4.269   4.452  1.00  0.00
C
HETATM   16  C12 CP13  900       5.461  -4.533   3.740  1.00  0.00
C
HETATM   17  N4  CP13  900       4.524  -3.633   3.932  1.00  0.00
N
HETATM   18  N5  CP13  900       5.069  -2.760   4.784  1.00  0.00
N
HETATM   19  C13 CP13  900       6.336  -3.116   5.162  1.00  0.00
C
HETATM   20  C14 CP13  900       4.312  -1.592   5.186  1.00  0.00
C
HETATM   21  N6  CP13  900      11.518  -9.704   4.172  1.00  0.00
N
HETATM   22  C15 CP13  900      11.435 -10.553   3.128  1.00  0.00
C
HETATM   23  C16 CP13  900      12.195 -11.900   3.237  1.00  0.00
C
HETATM   24  O2  CP13  900      10.826 -10.318   2.084  1.00  0.00
O
HETATM   25  C17 CP13  900      13.740 -11.852   3.014  1.00  0.00
C
HETATM   26  C18 CP13  900      14.299 -13.274   2.732  1.00  0.00
C
HETATM   27  C19 CP13  900      15.831 -13.298   2.552  1.00  0.00
C
HETATM   28  C20 CP13  900      16.291 -12.306   1.469  1.00  0.00
C
HETATM   29  C21 CP13  900      15.763 -10.892   1.761  1.00  0.00
C
HETATM   30  C22 CP13  900      14.230 -10.863   1.920  1.00  0.00
C
HETATM   31  N7  CP13  900      11.822 -12.489   4.571  1.00  0.00
N1+
HETATM   32  H1  CP13  900      12.878  -3.046   5.056  1.00  0.00
H
HETATM   33  H2  CP13  900       9.005  -2.539   4.917  1.00  0.00
H
HETATM   34  H3  CP13  900      12.887  -7.586   4.629  1.00  0.00
H
HETATM   35  H4  CP13  900       8.918  -9.047   3.890  1.00  0.00
H
HETATM   36  H5  CP13  900       7.006  -6.863   3.975  1.00  0.00
H
HETATM   37  H6  CP13  900       5.300  -5.360   3.063  1.00  0.00
H
HETATM   38  H7  CP13  900       6.898  -2.507   5.856  1.00  0.00
H
HETATM   39  H8  CP13  900       4.941  -0.933   5.784  1.00  0.00
H
HETATM   40  H9  CP13  900       3.447  -1.905   5.771  1.00  0.00
H
HETATM   41  H10 CP13  900       3.981  -1.049   4.300  1.00  0.00
H
```

```
HETATM   42  H11 CP13  900      12.130  -9.944   4.934  1.00  0.00
H
HETATM   43  H12 CP13  900      11.747 -12.611   2.537  1.00  0.00
H
HETATM   44  H13 CP13  900      14.175 -11.510   3.956  1.00  0.00
H
HETATM   45  H14 CP13  900      14.029 -13.958   3.538  1.00  0.00
H
HETATM   46  H15 CP13  900      13.834 -13.674   1.830  1.00  0.00
H
HETATM   47  H16 CP13  900      16.162 -14.306   2.297  1.00  0.00
H
HETATM   48  H17 CP13  900      16.312 -13.051   3.500  1.00  0.00
H
HETATM   49  H18 CP13  900      17.381 -12.294   1.413  1.00  0.00
H
HETATM   50  H19 CP13  900      15.937 -12.636   0.490  1.00  0.00
H
HETATM   51  H20 CP13  900      16.068 -10.204   0.971  1.00  0.00
H
HETATM   52  H21 CP13  900      16.223 -10.532   2.684  1.00  0.00
H
HETATM   53  H22 CP13  900      13.928  -9.842   2.158  1.00  0.00
H
HETATM   54  H23 CP13  900      13.758 -11.104   0.966  1.00  0.00
H
HETATM   55  H24 CP13  900      10.857 -12.268   4.776  1.00  0.00
H
HETATM   56  H25 CP13  900      11.927 -13.493   4.524  1.00  0.00
H
HETATM   57  H26 CP13  900      12.434 -12.128   5.293  1.00  0.00
H
CONECT    1    2    7
CONECT    2    1    3   32
CONECT    3    2    4    8
CONECT    4    3    5    9
CONECT    5    4    6   12
CONECT    6    5    7   13
CONECT    7    1    6   33
CONECT    8    3
CONECT    9    4   10   34
CONECT   10    9   11   21
CONECT   11   10   12   35
CONECT   12    5   11   14
CONECT   13    6   14   15
CONECT   14   12   13   36
CONECT   15   13   19   16
CONECT   16   15   17   37
CONECT   17   16   18
CONECT   18   17   19   20
CONECT   19   15   18   38
CONECT   20   18   39   40   41
CONECT   21   10   22   42
CONECT   22   21   23   24
CONECT   23   22   25   31   43
CONECT   24   22
CONECT   25   23   30   26   44
```

```
CONECT   26   25   27   45   46
CONECT   27   26   28   47   48
CONECT   28   27   29   49   50
CONECT   29   28   30   51   52
CONECT   30   25   29   53   54
CONECT   31   23   55   56   57
CONECT   32    2
CONECT   33    7
CONECT   34    9
CONECT   35   11
CONECT   36   14
CONECT   37   16
CONECT   38   19
CONECT   39   20
CONECT   40   20
CONECT   41   20
CONECT   42   21
CONECT   43   23
CONECT   44   25
CONECT   45   26
CONECT   46   26
CONECT   47   27
CONECT   48   27
CONECT   49   28
CONECT   50   28
CONECT   51   29
CONECT   52   29
CONECT   53   30
CONECT   54   30
CONECT   55   31
CONECT   56   31
CONECT   57   31
ENDMDL
END
```

13

```
REMARK   4      COMPLIES WITH FORMAT V. 3.0, 1-DEC-2006
REMARK 888
REMARK 888 WRITTEN BY MAESTRO (A PRODUCT OF SCHRODINGER, LLC)
TITLE     compound_6A
MODEL        1
HETATM    1  N1  CP14  900       5.514  -3.883   4.571  1.00  0.00
N
HETATM    2  C1  CP14  900       6.339  -3.401   5.501  1.00  0.00
C
HETATM    3  C2  CP14  900       7.708  -3.732   5.668  1.00  0.00
C
HETATM    4  C3  CP14  900       8.245  -4.639   4.726  1.00  0.00
C
HETATM    5  N2  CP14  900       7.424  -5.154   3.771  1.00  0.00
N
HETATM    6  C4  CP14  900       6.131  -4.766   3.753  1.00  0.00
C
HETATM    7  N3  CP14  900       8.219  -3.092   6.767  1.00  0.00
N
```

```
HETATM    8  C5  CP14  900       7.234  -2.356   7.217  1.00  0.00
C
HETATM    9  N4  CP14  900       6.073  -2.496   6.496  1.00  0.00
N
HETATM   10  N5  CP14  900       5.360  -5.332   2.762  1.00  0.00
N
HETATM   11  C6  CP14  900       9.674  -5.104   4.689  1.00  0.00
C
HETATM   12  C7  CP14  900       9.981  -6.482   4.605  1.00  0.00
C
HETATM   13  C8  CP14  900      11.322  -6.894   4.572  1.00  0.00
C
HETATM   14  C9  CP14  900      12.352  -5.970   4.638  1.00  0.00
C
HETATM   15  C10 CP14  900      12.060  -4.616   4.694  1.00  0.00
C
HETATM   16  C11 CP14  900      10.729  -4.166   4.694  1.00  0.00
C
HETATM   17  O1  CP14  900       8.938  -7.392   4.572  1.00  0.00
O
HETATM   18  C12 CP14  900       9.200  -8.762   4.284  1.00  0.00
C
HETATM   19  O2  CP14  900      10.408  -2.821   4.650  1.00  0.00
O
HETATM   20  C13 CP14  900      11.444  -1.849   4.524  1.00  0.00
C
HETATM   21  H1  CP14  900       7.271  -1.686   8.067  1.00  0.00
H
HETATM   22  H2  CP14  900       5.214  -2.006   6.695  1.00  0.00
H
HETATM   23  H3  CP14  900       4.395  -5.058   2.689  1.00  0.00
H
HETATM   24  H4  CP14  900       5.826  -5.941   2.108  1.00  0.00
H
HETATM   25  H5  CP14  900      11.593  -7.937   4.516  1.00  0.00
H
HETATM   26  H6  CP14  900      13.375  -6.314   4.627  1.00  0.00
H
HETATM   27  H7  CP14  900      12.886  -3.924   4.719  1.00  0.00
H
HETATM   28  H8  CP14  900       9.689  -8.878   3.315  1.00  0.00
H
HETATM   29  H9  CP14  900       8.256  -9.306   4.240  1.00  0.00
H
HETATM   30  H10 CP14  900       9.817  -9.229   5.054  1.00  0.00
H
HETATM   31  H11 CP14  900      12.059  -2.029   3.641  1.00  0.00
H
HETATM   32  H12 CP14  900      12.081  -1.831   5.409  1.00  0.00
H
HETATM   33  H13 CP14  900      11.019  -0.854   4.410  1.00  0.00
H
CONECT    1    2    6
CONECT    2    1    3    9
CONECT    3    2    4    7
CONECT    4    3    5   11
CONECT    5    4    6
```

```
CONECT    6    1    5   10
CONECT    7    3    8
CONECT    8    7    9   21
CONECT    9    2    8   22
CONECT   10    6   23   24
CONECT   11    4   16   12
CONECT   12   11   13   17
CONECT   13   12   14   25
CONECT   14   13   15   26
CONECT   15   14   16   27
CONECT   16   11   15   19
CONECT   17   12   18
CONECT   18   17   28   29   30
CONECT   19   16   20
CONECT   20   19   31   32   33
CONECT   21    8
CONECT   22    9
CONECT   23   10
CONECT   24   10
CONECT   25   13
CONECT   26   14
CONECT   27   15
CONECT   28   18
CONECT   29   18
CONECT   30   18
CONECT   31   20
CONECT   32   20
CONECT   33   20
ENDMDL
END
```

14

```
REMARK   4      COMPLIES WITH FORMAT V. 3.0, 1-DEC-2006
REMARK 888
REMARK 888 WRITTEN BY MAESTRO (A PRODUCT OF SCHRODINGER, LLC)
TITLE     compound_16
MODEL        1
HETATM    1  C1  CP15  900      12.146  -6.301   4.477  1.00  0.00
C
HETATM    2  N1  CP15  900      11.244  -5.276   4.650  1.00  0.00
N
HETATM    3  C2  CP15  900       9.841  -5.526   4.628  1.00  0.00
C
HETATM    4  C3  CP15  900       9.419  -6.858   4.427  1.00  0.00
C
HETATM    5  C4  CP15  900      10.339  -7.886   4.232  1.00  0.00
C
HETATM    6  C5  CP15  900      11.704  -7.595   4.252  1.00  0.00
C
HETATM    7  C6  CP15  900       9.173  -4.283   4.845  1.00  0.00
C
HETATM    8  C7  CP15  900      10.180  -3.337   4.972  1.00  0.00
C
HETATM    9  C8  CP15  900      11.438  -3.916   4.864  1.00  0.00
C
```

```
HETATM   10  C9  CP15  900       12.738  -3.248   4.972  1.00  0.00
C
HETATM   11  C10 CP15  900       12.731  -1.733   5.143  1.00  0.00
C
HETATM   12  O1  CP15  900       13.819  -3.833   4.971  1.00  0.00
O
HETATM   13  O2  CP15  900        9.818  -9.150   4.038  1.00  0.00
O
HETATM   14  C11 CP15  900        7.707  -4.019   4.949  1.00  0.00
C
HETATM   15  N2  CP15  900        6.886  -4.912   4.335  1.00  0.00
N
HETATM   16  C12 CP15  900        5.545  -4.725   4.416  1.00  0.00
C
HETATM   17  C13 CP15  900        4.980  -3.655   5.114  1.00  0.00
C
HETATM   18  C14 CP15  900        5.826  -2.748   5.753  1.00  0.00
C
HETATM   19  C15 CP15  900        7.206  -2.932   5.675  1.00  0.00
C
HETATM   20 CL1  CP15  900        4.509  -5.858   3.629  1.00  0.00
Cl
HETATM   21  C16 CP15  900       10.662 -10.293   4.171  1.00  0.00
C
HETATM   22  C17 CP15  900       11.329 -10.649   2.830  1.00  0.00
C
HETATM   23  C18 CP15  900       12.326 -11.809   2.953  1.00  0.00
C
HETATM   24  H1  CP15  900       13.196  -6.072   4.496  1.00  0.00
H
HETATM   25  H2  CP15  900        8.366  -7.097   4.416  1.00  0.00
H
HETATM   26  H3  CP15  900       12.444  -8.362   4.093  1.00  0.00
H
HETATM   27  H4  CP15  900       10.008  -2.285   5.146  1.00  0.00
H
HETATM   28  H5  CP15  900       13.746  -1.348   5.230  1.00  0.00
H
HETATM   29  H6  CP15  900       12.182  -1.456   6.044  1.00  0.00
H
HETATM   30  H7  CP15  900       12.251  -1.258   4.288  1.00  0.00
H
HETATM   31  H8  CP15  900        3.908  -3.532   5.160  1.00  0.00
H
HETATM   32  H9  CP15  900        5.420  -1.911   6.301  1.00  0.00
H
HETATM   33  H10 CP15  900        7.868  -2.237   6.171  1.00  0.00
H
HETATM   34  H11 CP15  900       11.393 -10.169   4.972  1.00  0.00
H
HETATM   35  H12 CP15  900       10.028 -11.124   4.484  1.00  0.00
H
HETATM   36  H13 CP15  900       10.556 -10.907   2.105  1.00  0.00
H
HETATM   37  H14 CP15  900       11.844  -9.781   2.419  1.00  0.00
H
```

```
HETATM   38  H15 CP15  900       12.777 -12.041   1.987  1.00  0.00
H
HETATM   39  H16 CP15  900       11.838 -12.714   3.316  1.00  0.00
H
HETATM   40  H17 CP15  900       13.133 -11.565   3.644  1.00  0.00
H
CONECT    1    2    6   24
CONECT    2    1    3    9
CONECT    3    2    4    7
CONECT    4    3    5   25
CONECT    5    4    6   13
CONECT    6    1    5   26
CONECT    7    3    8   14
CONECT    8    7    9   27
CONECT    9    2    8   10
CONECT   10    9   11   12
CONECT   11   10   28   29   30
CONECT   12   10
CONECT   13    5   21
CONECT   14    7   19   15
CONECT   15   14   16
CONECT   16   15   17   20
CONECT   17   16   18   31
CONECT   18   17   19   32
CONECT   19   14   18   33
CONECT   20   16
CONECT   21   13   22   34   35
CONECT   22   21   23   36   37
CONECT   23   22   38   39   40
CONECT   24    1
CONECT   25    4
CONECT   26    6
CONECT   27    8
CONECT   28   11
CONECT   29   11
CONECT   30   11
CONECT   31   17
CONECT   32   18
CONECT   33   19
CONECT   34   21
CONECT   35   21
CONECT   36   22
CONECT   37   22
CONECT   38   23
CONECT   39   23
CONECT   40   23
ENDMDL
END
```

Ensemble docking

Table S1. Mean docking score and standard deviation of ensemble docking results.

Compound	Mean docking score	St. Dev.
**1**	-2.54	1.23
**2**	-3.85	0.91
**3**	-2.73	1.22
**4**	-3.12	0.64
**5**	-2.50	0.81
**6**	-3.77	1.00
**7**	-2.96	0.62
**8**	-3.53	1.20

Additional MD for 4UIW (simulation time: 1 μs)

The calculated MM-GBSA ΔG_{bind} value is -74.27 kcal/mol

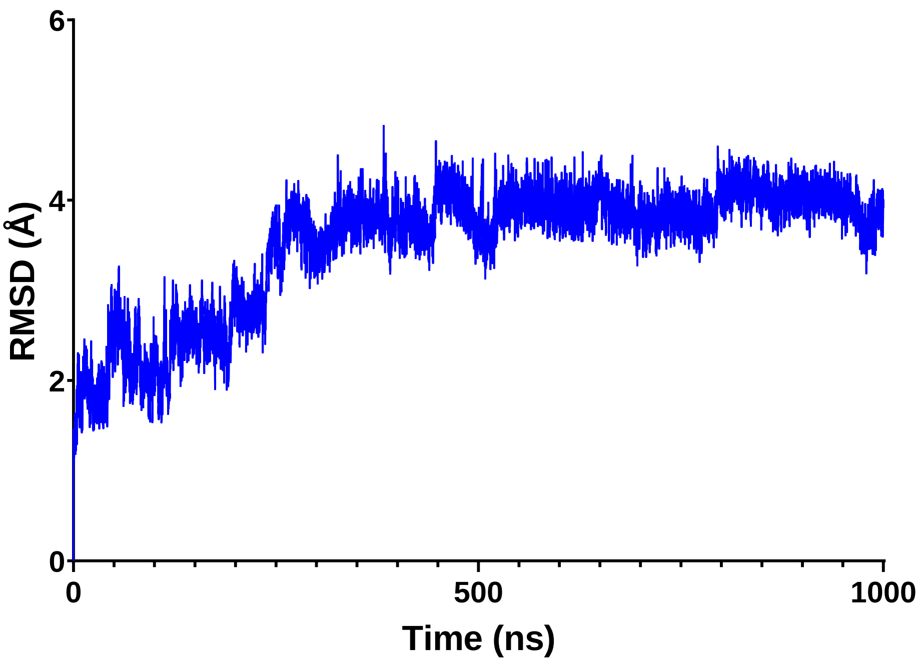

Figure S1. RMSD of the backbone atoms of 4UIW.

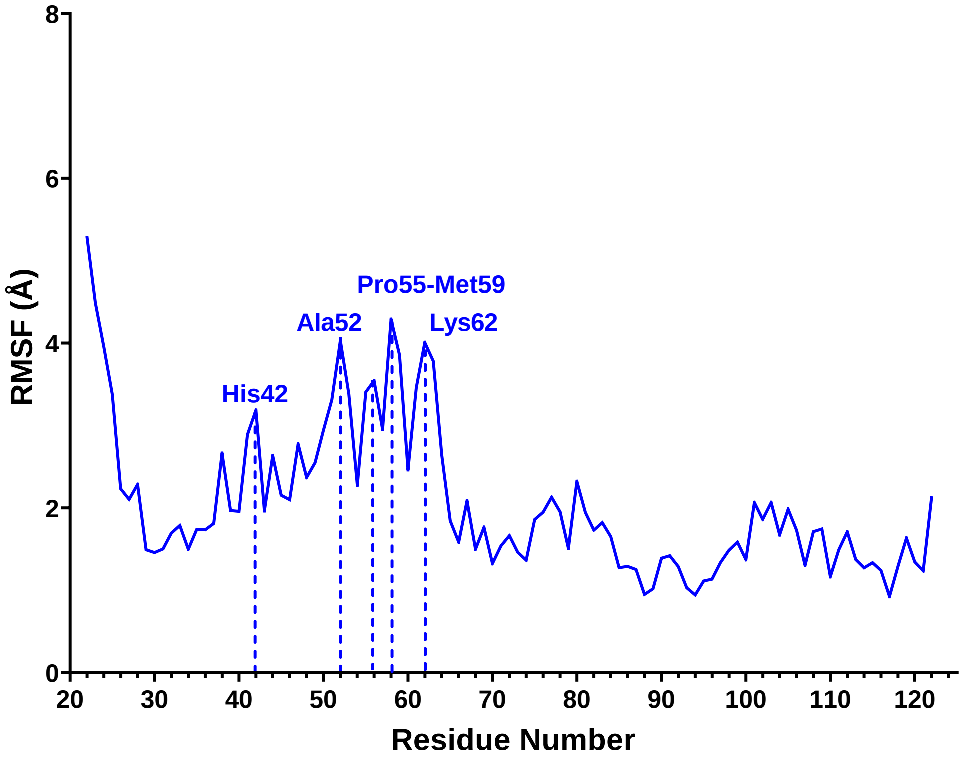

Figure S2. RMSF of 4UIW. The data reported are the mean of the backbone and side chains values and residues with an RMSF above 3.0 Å are labeled.

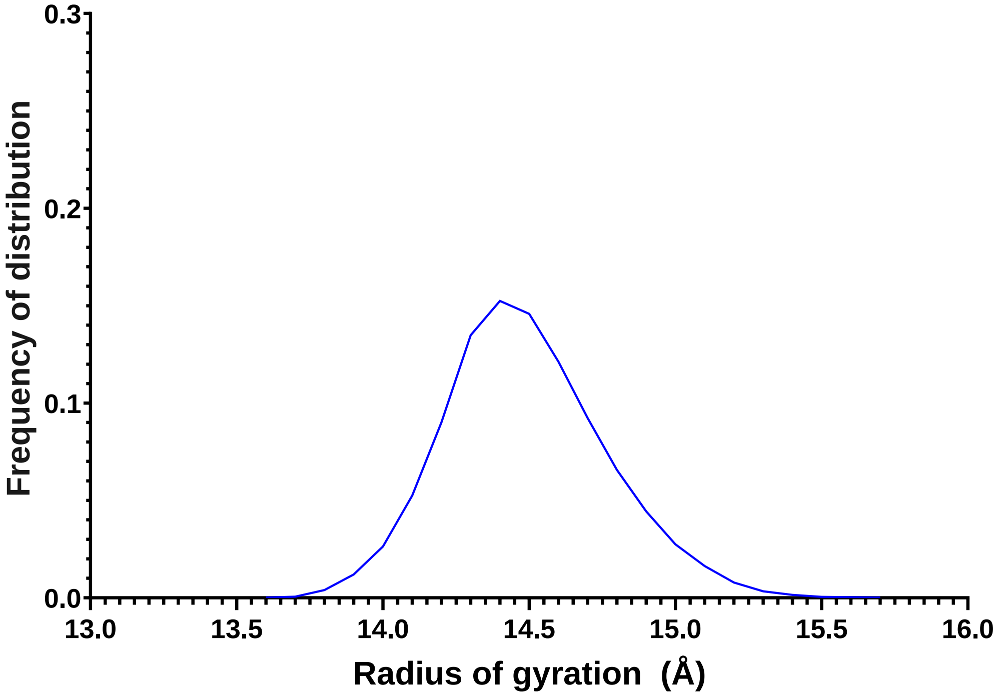

Figure S3. The radius of gyration of 4UIW plotted as frequency of distribution.

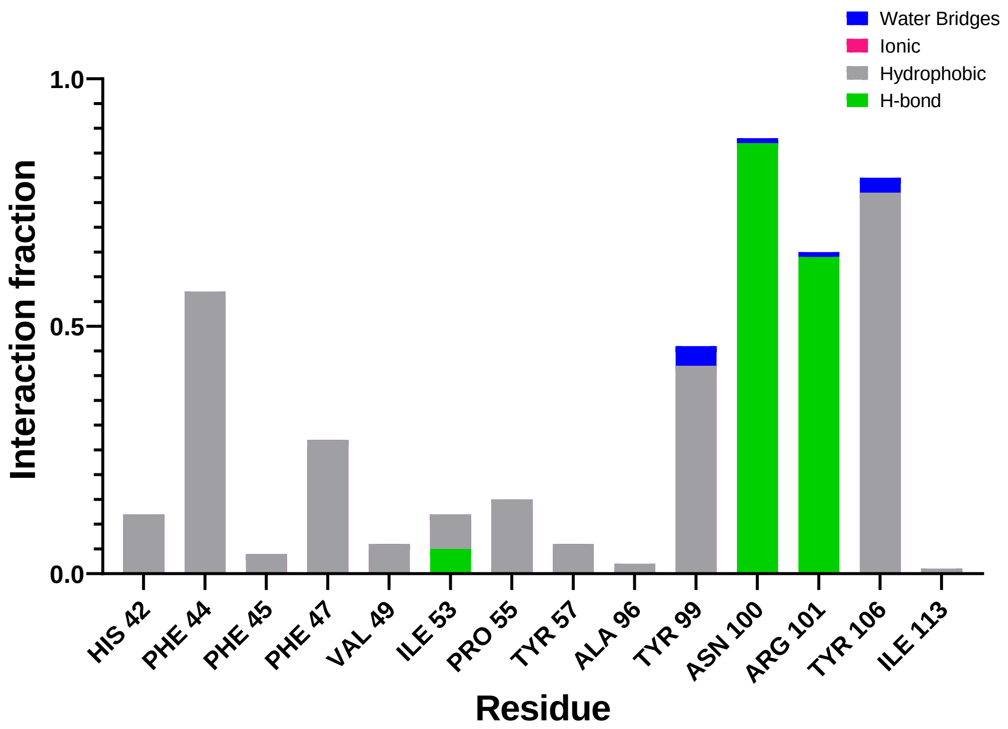

Figure S4. Protein-ligand interactions of 4UIW. Values reported are expressed as the fraction of total time.

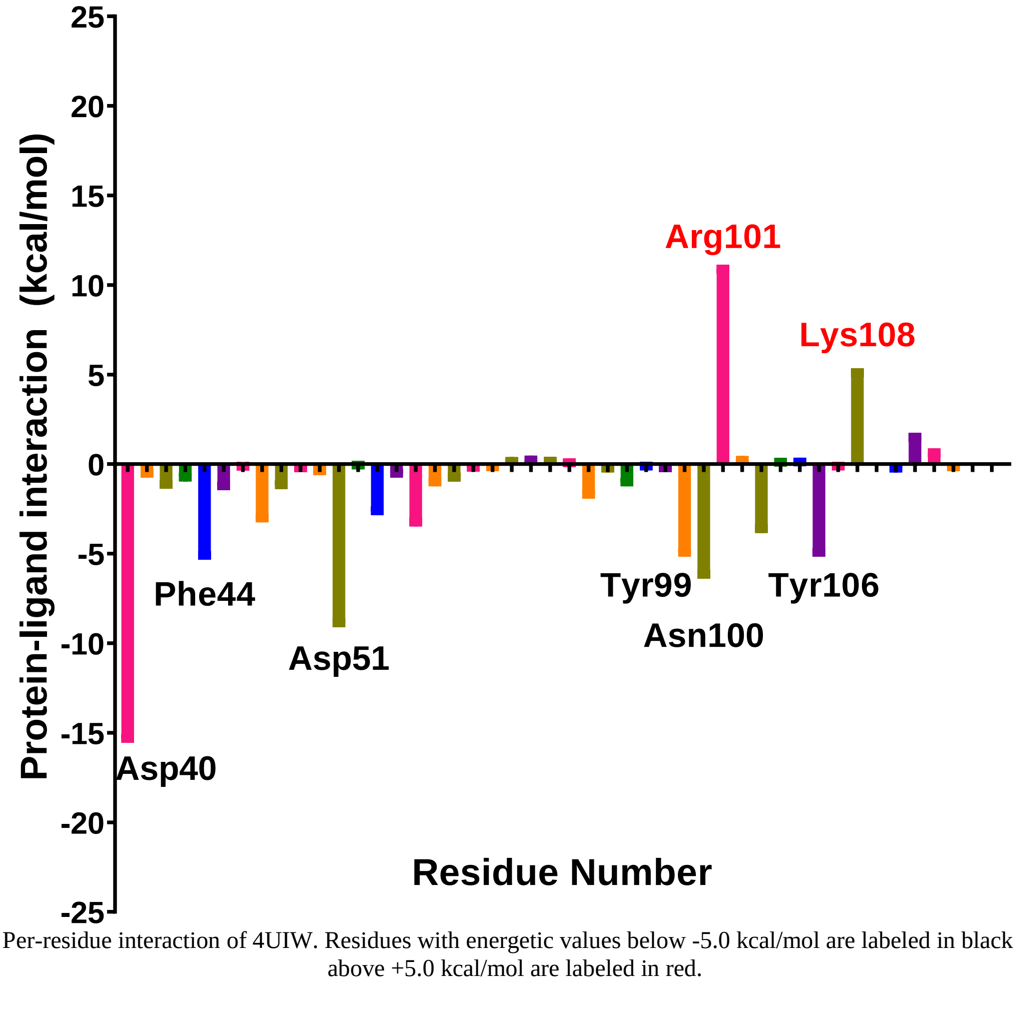

Figure S5. Per-residue interaction of 4UIW. Residues with energetic values below -5.0 kcal/mol are labeled in black and residues above +5.0 kcal/mol are labeled in red.
